# Trends in Nurses’ Sleep: A Meta‐Regression Involving 219,245 Nurses From 45 Countries

**DOI:** 10.1155/jonm/9051680

**Published:** 2026-08-27

**Authors:** Leanna G. Santostefano, Kurt Lushington, Siobhan Banks, Grant R. Tomkinson, Justin J. Lang, Kristin Graham, Hayley Ruf, Kiriaki Stewart, Kirstie Howland, Michelle Headland, Michael Musker, Lisa Matricciani

**Affiliations:** ^1^ Behaviour-Brain-Body Research Centre, Adelaide University, Adelaide, South Australia, Australia, adelaide.edu.au; ^2^ Alliance for Research in Exercise, Nutrition and Activity (ARENA), School of Allied Health and Human Performance, Adelaide University, Adelaide, South Australia, Australia, adelaide.edu.au; ^3^ Centre for Surveillance and Applied Research, Public Health Agency of Canada, Ottawa, Ontario, Canada, phac-aspc.gc.ca; ^4^ School of Epidemiology and Public Health, Faculty of Medicine, University of Ottawa, Ottawa, Ontario, Canada, uottawa.ca; ^5^ College of Health, Adelaide University, Adelaide, South Australia, Australia, adelaide.edu.au; ^6^ IIMPACT in Health research, Adelaide University, Adelaide, South Australia, Australia, adelaide.edu.au; ^7^ Mental Health and Suicide Prevention Research and Education, Adelaide University, Adelaide, South Australia, Australia, adelaide.edu.au; ^8^ Rosemary Bryant AO Research Centre, Adelaide University, Adelaide, South Australia, Australia, adelaide.edu.au

**Keywords:** humans, nurse, sleep, sleep duration, sleep quality, temporal trends

## Abstract

**Aim:**

To examine temporal trends in nurses’ sleep duration and quality.

**Background:**

In recent decades, the nursing workplace demands have intensified. This is thought to have contributed to a decline in nurses’ sleep duration and quality. Despite concerns, it remains unclear if nurses’ sleep has changed.

**Method:**

A systematic database search was used to identify studies reporting nurses’ total sleep time or sleep quality (Pittsburgh Sleep Quality Index [PSQI]). Trends in mean sleep duration and global PSQI scores were analysed using generalised linear regression models adjusted for age, sex, geographic region, shift type and measurement method (self‐report vs. actigraphy).

**Results:**

Data were extracted from 280 studies representing 219,245 nurses. A significant decline of 28.7 min was observed in sleep duration (95% CI: 26.2, 33.1, standardised effect size [ES]: 0.43) between 1985 and 2022, while a 1.6‐point increase in global PSQI scores was observed between 1990 and 2023, indicative of poorer sleep quality (95% CI: 1.47, 1.77, ES: 0.49).

**Conclusion:**

These findings suggest a decline in nurses’ sleep duration and quality over recent decades. These trends suggest that targeted interventions may be needed to improve nurses’ sleep health.

**Implications for Nursing Management:**

Temporal declines in nurses’ sleep duration and quality may reflect an increased risk of fatigue, impaired clinical performance, staff burnout and work‐related accidents. This study provides important insights into workforce well‐being and highlights the need for nurse managers to recognise staff susceptibility to poor sleep and proactively develop and implement strategies that promote healthy sleep.

## 1. Introduction

Nurses represent the largest professional group within the global healthcare system, which has become increasingly demanding both physically and mentally [1–3]. Progressive staff shortages have resulted in many nurses having to work longer hours in a complex healthcare system characterised by growing workplace violence and an increasing number of patients with complex health needs—particularly older adults with chronic health conditions [1, 2, 4]. These factors are thought to negatively impact nurses’ well‐being, including sleep [5, 6], with reviews [5, 6], cross‐sectional studies [7, 8] and meta‐analyses [9] indicating that many nurses sleep less than recommended and report poor sleep quality. Despite growing concerns, it remains unclear whether there has been a decline in nurses’ well‐being, particularly sleep health.

While it is often believed that sleep duration and quality have declined in the general adult population, empirical evidence remains mixed and inconclusive [10–12]. Mixed findings have been attributed to measurement limitations (e.g., time‐use methods vs. sleep diaries), restricted timeframes and the differences in the types of days assessed (e.g., weekends vs. weekdays) [10, 11]. A further consideration is that population‐based studies do not account for person‐level factors (such as occupation), which are known to influence sleep [10, 13]. Given the unique pressures and factors influencing nurses’ sleep, it is possible that temporal trends in sleep may differ within this occupational group.

Research on nurses’ sleep is particularly important. There is a large body of evidence indicating that nurses who experience poor sleep are more likely to face job dissatisfaction, occupational stress and intentions to leave the profession [14, 15]. Beyond workforce sustainability, sleep is also critical for both nurses’ well‐being and patient safety. Poor sleep has been negatively associated with nurses’ physical and mental well‐being, increasing their risk of burnout, chronic health conditions and fatigue [16, 17]. Sleep‐deprived nurses are also more likely to make medication errors and less likely to detect patient deterioration, which directly compromises patient safety [17, 18].

Given the importance of sleep for nurses’ well‐being, patient safety and workforce sustainability, it is of interest to determine whether nurses’ sleep has changed over time. Such insight may help identify emerging risks and inform workforce management and planning strategies. If sleep has worsened, it may signal growing pressures that threaten both workforce stability and care quality, highlighting the need for more targeted interventions to support healthy sleep among nurses. Alternatively, stable or improving trends might suggest that either poor sleep is less prevalent than commonly believed or that sufficient policies and strategies are in place to manage nurses’ sleep health. However, sleep is not typically the focus of well‐being interventions [19, 20], and few resources are available to promote and support healthy sleep [21]. Thus, assessing temporal trends may help determine whether targeted strategies should be developed or integrated into practice.

Therefore, the aim of this study was to examine temporal trends in nurses’ sleep duration and quality by reviewing all published studies that have reported on nurses’ sleep duration or sleep quality.

## 2. Methods

To identify studies that report on nurses’ sleep duration or quality, a systematic literature search was conducted and reported in accordance with the Preferred Reporting Items for Systematic Reviews and Meta‐Analyses (PRISMA) 2020 statement [22]. The protocol was registered in the International Prospective Register of Systematic Reviews (PROSPERO) database (ID: CRD42024540892).

### 2.1. Eligibility Criteria

The following inclusion criteria were applied:

i. Only studies where ‘nurse’ was the primary occupation.
ii. For analytical purposes, we required that mean values were based on a minimum of 30 participants. This was to meet assumptions of the central limit theorem where the *n* = 30 threshold helps ensure that the sampling distribution of the mean approximates normality, thereby supporting the validity of trend analyses that rely on parametric assumptions [23].
iii. We selected only full‐text, peer‐reviewed, primary observational studies (e.g., case–control studies, cross‐sectional studies, intervention studies [only baseline data were extracted] and longitudinal studies [only baseline data were extracted]).
iv. Studies had to be published in English as we did not have the resources available to undertake translation.
v. Studies reported mean total sleep time derived from either self‐report (e.g., sleep diary or single‐item questionnaire) or actigraphy.
vi. Studies reported mean global scores for the Pittsburgh Sleep Quality Index (PSQI) (range 0–21 points, with higher scores indicating worse sleep quality). The PSQI is widely used to assess sleep quality and has high internal and external validity [24]. Studies were excluded if they only provided categorical data (e.g., sleep duration < 7 h, 7–9 h, > 9 h; ‘good’ or ‘poor’ sleep and PSQI > 5 or < 5).

### 2.2. Information Sources

A search of five electronic databases (MEDLINE, Embase, Emcare, PsycINFO [via OVID] and Scopus) was undertaken on 22 April 2024. The reference lists of relevant topical reviews were examined to identify additional eligible studies.

### 2.3. Search Strategy

The systematic search strategy was developed in consultation with an academic librarian. The first set of search terms identified the measure (sleep duration or quality), and the second identified the population (nurses). Relevant subject headings, related MeSH headings and keywords were used. No year limit was applied given that this study was focused on determining temporal trends. The search strategies used are described in Supporting File 1.

### 2.4. Selection Process

All identified records were uploaded into EndNote (v21.0, Clarivate Analytics, Philadelphia, PA, USA), where duplicates were automatically removed. Dissertations, conference abstracts, supplements and additional duplicates were manually removed before records were exported into Covidence (Veritas Health Innovation, Melbourne, VIC, Australia), where remaining duplicates were removed. The screening process was independently completed by two authors, who screened all titles and abstracts and then all full‐text articles. One author (L.S.) reviewed all titles/abstracts and full texts, with one of the following eight authors (L.M., K.L., M.M., K.G., K.S., H.R., K.H. and M.H.) providing the independent second review. All conflicts were resolved through discussion.

### 2.5. Data Collection Process

Data extraction was undertaken using a protocol developed by the research team (Supporting File 2). For each included study, data were independently extracted by two reviewers. The extracted datasets were then cross‐checked for consistency, and any discrepancies were resolved through discussion. Studies were also screened to identify duplicate datasets or subsets of a larger study. When two articles referred to the same dataset, the study providing the most detailed descriptive data was included.

### 2.6. Data Items

Variables for data extraction included both study‐level and participant‐level variables. Key study‐level variables included study sample size and the year of measurement. When studies reported a range of years, the year of measurement was expressed as the midpoint of the reported measurement period (e.g., 2000 was taken as the midpoint measurement year for data collected between 1999 and 2001). When missing, attempts were not made to contact the original author, and an estimate based on reported data was imputed—a methodology that has been used in other peer‐reviewed articles examining historical trends [25]. Given that the aim of this study was to examine temporal trends, attempts to contact authors for missing data were considered inappropriate due to the potential for differential response by publication year, which may have introduced systematic bias into the trend estimates. Key participant‐level variables included both sleep duration and quality, where measures of central tendency (mean) and dispersion (standard deviation [SD]) were extracted. The global PSQI score was used to assess sleep quality, and total sleep time was used to assess sleep duration, derived from either self‐report or actigraphy.

The following covariates were included in the analyses because of their known association with sleep: geographic region, sex, age and shift type [26–29]. The measurement method (i.e., self‐report or actigraphy) was also included in the analyses of studies that reported sleep duration. The country where the study was conducted was recorded, and the geographic region was operationalised according to the classifications created by the World Health Organization [30] (African Region [AFR], Region of the Americas [AMR], Eastern Mediterranean Region [EMR], European Region [EUR], South‐East Asia Region [SEAR] and Western Pacific Region [WPR]). Sex was recorded as the percentage of females within each study sample. Age was expressed as the mean age of the sample. Shift type was operationalised as either ‘worked night hours’ (i.e., participants worked night‐time hours, whether on a fixed or rotating schedule) or ‘worked non‐night hours’ (i.e., only morning, daytime or evening shifts).

### 2.7. Risk of Bias Assessment

Given the exploratory and descriptive aim of this review, a formal risk of bias assessment was not undertaken. The included studies were highly heterogeneous in design and methodology, and applying a single critical appraisal tool across all studies would not have been appropriate. While we did not use a critical appraisal tool to evaluate the methodological quality of studies, we only included studies with at least 30 participants to ensure that samples were sufficiently large to improve our confidence in the extracted mean sleep estimates.

### 2.8. Synthesis Methods and Data Analysis

Analyses were conducted in SAS EG 7.1 (Cary, NC, USA) using a method informed by Tomkinson [31]. Sample‐weighted general linear models (proc GLM) were used to assess temporal trends in nurses’ sleep duration and quality, with year of measurement as the independent variable and either mean total sleep time (Model 1 [sleep duration]) or mean global PSQI scores (Model 2 [sleep quality]) as the dependent variables. This approach was selected to examine population‐level temporal trends across published datasets rather than to estimate pooled intervention effects or between‐study heterogeneity, as would typically be performed in a conventional meta‐analysis. Within‐study clustering was considered minimal because most included studies contributed a single data point. All models were adjusted for the following covariates: geographic region, sex, age and shift type. These covariates were included in the analyses because of their known association with sleep [26–29]. Models analysing sleep duration were additionally adjusted for the measurement method (e.g., subjective or objective).

For all analyses, the year of measurement was modelled both as a continuous variable and as a categorical variable (grouped by decade: 1985–1999, 2000–2009, 2010–2019 and 2020–2022 for sleep duration; and 1990–1999, 2000–2009, 2010–2019 and 2020–2023 for sleep quality, with the earliest decade as the reference category). Modelling year as both a continuous and categorical variable allowed assessment of overall linear temporal trends and potential nonlinear or period‐specific differences, including during the COVID‐19 pandemic, which has been reported to be particularly detrimental to nurses’ sleep [32, 33].

Trends were expressed as mean rates of change (i.e., the regression coefficient) and as standardised (Cohen’s) effect sizes (ES) (i.e., the regression coefficient divided by the pooled SD). Mean rates of change were expressed for the period by multiplying the yearly rate of change by the number of years between the first and last data point. For sleep duration, negative trends indicated less sleep over time, and positive trends indicated more sleep over time. Since a higher PSQI score represents poorer sleep quality, positive trends indicated poorer sleep quality over time, while negative trends indicated better sleep quality over time. The magnitudes of the ES were interpreted as small (ES = 0.20–0.49), moderate (ES = 0.50–0.79) and large (ES ≥ 0.80) [34]. Alpha was set at 0.05 for all analyses.

## 3. Results

### 3.1. Study Selection

**FIGURE 1:**
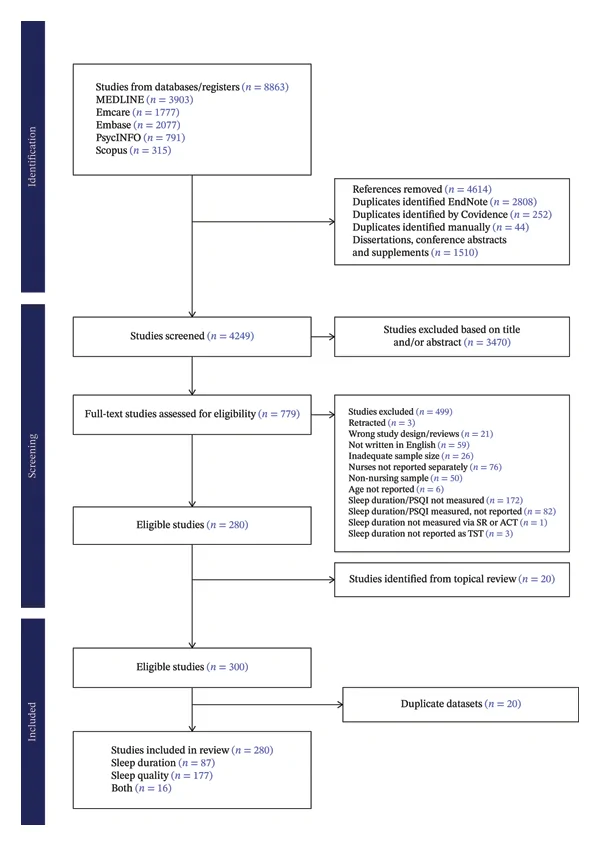
PRISMA flow diagram. Note. PSQI = Pittsburgh Sleep Quality Index; SR = self‐report; ACT = actigraphy and TST = total sleep time.

The systematic search identified 8863 unique articles from the online database search. After removing duplicates, dissertations, conference abstracts and supplements, 4249 records were screened at the title/abstract level, which was reduced to 779 papers for full‐text screening. Of these, 499 papers were excluded as they did not meet the inclusion criteria or used a duplicate dataset. The reasons for study exclusion are outlined in Supporting File 3 as per the PRISMA 2020 guidelines. A total of 280 unique studies were included in this review (Supporting File 4). Figure 1 provides the PRISMA flow diagram of the screening process.

### 3.2. Studies Characteristics

A total of 280 studies were included, with 87 focused on sleep duration, 177 focused on sleep quality and 16 focused on both sleep duration and quality. Sleep duration was mostly assessed by self‐report (80.5%, 83/103 studies, *n* = 118,390), and the remainder was assessed by actigraphy (19.5%, 20/103 studies, *n* = 3675).

As presented in Table 1, data were collected on 219,245 nurses across six geographical regions. Most nurses were female (94.6%) with a mean age of 36 years (range: 23–61 years), and most worked either as part of a rotating or fixed shift schedule that included night hours (88.6%). The full dataset is available in Supporting File 5.

**TABLE 1:** Participant demographic statistics.

	Number of studies	Sample size	Percentage of sample (%)
Female	280	207,339	94.6
Geographical region			
Western Pacific Region	87	164,811	75.1
European Region	70	22,403	10.2
Region of the Americas	46	12,949	5.9
South‐East Asian Region	37	8,922	4.1
Eastern Mediterranean Region	38	9,550	4.4
African Region	2	610	0.3
Type of Shifts Worked			
Worked night hours	207	194,180	88.6
Worked non‐night hours	37	8,326	3.8
Not reported	36	16,739	7.6
Age Range			
20–29 years	72	34,931	15.9
30–39 years	157	102,203	46.6
40–49 years	48	81,766	37.3
50–60 years	3	345	0.2

### 3.3. Temporal Trends in Sleep Duration

**FIGURE 2:**
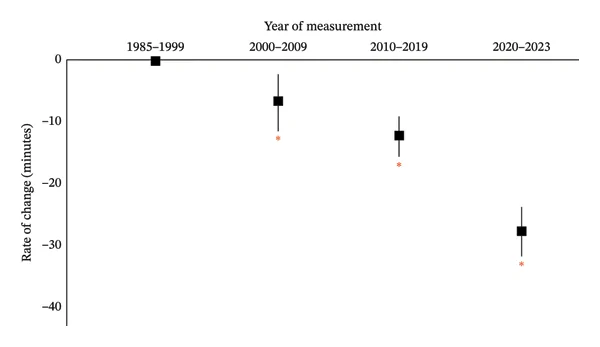
Decade‐by‐decade rate of change in nurses’ sleep duration relative to the 1985–1999 baseline period. Note. ^∗^Denotes statistically significant change compared with baseline period.

A total of 103 studies representing 125,172 nurses provided estimates for sleep duration between 1985 and 2022. Over this 37‐year period, there was a small but significant decline of 28.7 min in mean total sleep time (95% CI: 26.2, 33.1, ES: 0.43). Adjusted models (including age, sex, shift type, measurement method and geographic region) are documented in Supporting File 5. As presented in Figure 2, significant temporal declines were also observed across each of the examined decades. Compared to the 1985–1999 period, nurse mean total sleep time decreased by 7.0 min (95% CI: 3.2, 10.9, ES: 0.10) between 2000 and 2009, 12.4 min (95% CI: 9.8, 14.9, ES: 0.19) between 2010 and 2019 and 27.2 min (95% CI: 24.1, 30.4, ES: 0.40) between 2020 and 2022.

### 3.4. Temporal Trends in Sleep Quality

**FIGURE 3:**
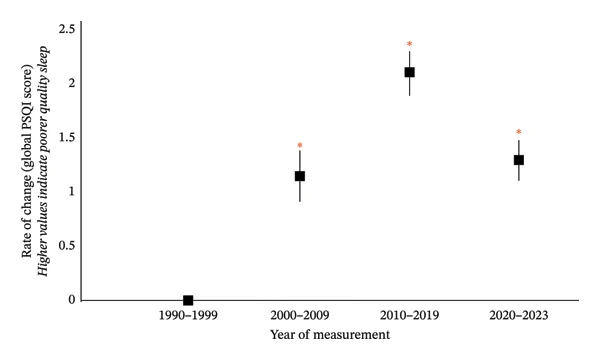
Decade‐by‐decade rate of change in nurses’ sleep quality relative to the 1990–1999 baseline period. Note. ^∗^Denotes statistically significant change compared with baseline period; PSQI = Pittsburgh Sleep Quality Index. Higher scores indicate poorer sleep quality.

A total of 193 studies representing 97,232 nurses provided estimates of sleep quality between 1990 and 2023. Over this 33‐year period, there was a small but significant increase of 1.6 points in mean global PSQI scores (95% CI: 1.5, 1.8, *p* < 0.0001; ES: 0.49), which suggests a decline in sleep quality. Adjusted models (including age, sex, shift type and geographic region) are presented in Table 2. As illustrated, significant temporal declines were observed across all decades (Figure 3). Compared to the period 1990–1999, nurse mean PSQI scores increased (indicating poorer sleep quality) by 1.1 points (95% CI: 0.9, 1.3, ES: 0.33) between 2000 and 2009, by 2.0 points (95% CI: 1.9, 2.2, ES: 0.62) between 2010 and 2019 and by 1.3 points (95% CI: 1.1, 1.4, ES: 0.38) between 2020 and 2023.

**TABLE 2:** General linear models assessing temporal trends in nurses’ sleep duration and quality.

	Estimate	SE	CI (95%)	*p* value
Sleep duration				
Intercept	29.877	1.477	26.983, 32.771	
Year of measurement	−0.013	0.001	−0.014, 0.015	
Age	0.009	0.001	0.007, 0.013	< 0.0001
Gender	0.024	0.001	0.024, 0.026	< 0.0001
Shift type				
Works night hours	−0.166	0.002	−0.199, −0.133	< 0.0001
Works non‐night hours	REF			
Method				
Actigraphy	0.753	0.020	0.713, 0.792	< 0.0001
Self‐report	REF			
Country				
African Region	0.241	0.049	0.144, 0.338	< 0.0001
South‐East Asia Region	0.057	0.022	0.013, 0.101	< 0.0117
Western Pacific Region	1.668	0.033	1.604, 1.734	< 0.0001
European Region	0.854	0.020	0.814, 0.893	< 0.0001
Region of the Americas	0.145	0.020	0.105, 0.185	< 0.0001
Eastern Mediterranean Region	REF			
Sleep quality				
Intercept	−87.830	4.720	−97.082, 78.578	< 0.0001
Year of measurement	0.049	0.002	0.044, 0.0535	< 0.0001
Age	−0.101	0.003	−0.106, −0.095	< 0.0001
Gender	0.035	0.001	0.0337, 0.0371	< 0.0001
Shift type				
Works night hours	0.023	0.045	0.609, −0.0645	0.609
Works non‐night hours	REF			
Country				
African Region	0.883	0.089	0.703, 1.051	< 0.0001
South‐East Asia Region	−4.537	0.074	−4.681, −4.392	< 0.0001
Western Pacific Region	−2.949	0.100	−3.145, −2.751	< 0.0001
European Region	−2.645	0.077	−2.795, −2.495	< 0.0001
Region of the Americas	−2.048	0.078	−2.201, −1.897	< 0.0001
Eastern Mediterranean Region	REF			

Abbreviations: CI = confidence interval, SE = standard error.

## 4. Discussion

The aim of this study was to determine temporal trends in nurses’ sleep duration and quality. Through a comprehensive systematic search that identified 280 unique studies, the inspection of sleep values revealed a significant decline of 28.7 min in sleep duration and a 1.6‐point reduction in sleep quality. These estimates seem to suggest a modest [35, 36] but concerning trend of a decline in both sleep duration and quality.

Our findings align with some, but not all, population‐based studies that examine temporal trends in adult sleep duration. In a systematic review of 12 repeated cross‐sectional studies describing secular trends in adult sleep duration from 15 developed countries over the past 40 years (1960s–2000s), Bin et al. [11] found that sleep duration had increased in seven countries, decreased in six and was inconclusive in two. For studies that reported a decline, trend estimates varied between approximately 4 and 24 min over a 14–33‐year time period [11], with our finding (28.7 min over a 37‐year period) slightly higher than this. Bin et al. [11] reported no consistent pattern to the changes in sleep duration across countries and suggested a range of factors might be responsible for the observed differences between countries, including work and occupational groups.

Although this study did not examine causal mechanisms, it is conceivable that the declines observed in the current study might reflect broader population trends and changing occupational factors. Indeed, a growing ageing population amidst a shrinking nursing workforce has created a global nursing shortage [37–39], resulting in extended work hours and heavier workloads [40, 41] for those who continue to work clinically. Both work hours [41, 42] and clinical demands [1, 3, 5, 6, 43–46] are known to negatively impact sleep, with time‐use studies suggesting that each additional hour worked is associated with an average reduction of 14 min of sleep [47, 48]. These issues may be particularly pronounced among nurses in specialised units, who are often expected to take on additional shifts beyond their regular schedules. In severely understaffed settings like emergency departments, 24‐h shifts are becoming increasingly common [41]. Despite clear evidence of the negative effects of excessive work hours and occupational fatigue, there is a lack of international regulation regarding nurse work hours [41]. This contrasts with other safety‐sensitive industries such as trucking, rail and nuclear energy, where strict legislative standards govern maximum working hours [41].

The temporal decline in nurses’ sleep might also reflect growing demands on the healthcare system, which has seen a growing need for mental and behavioural health services, rising incidents of patient violence against healthcare workers and an increasing number of older adults presenting with multiple complex and chronic health conditions [1, 3]. These factors contribute significantly to the psychological strain experienced by nurses. Nurses often struggle to psychologically detach after work, frequently engaging in worry, rumination and repetitive thoughts related to job responsibilities—such as concerns about specific patients or potential errors [44–46, 49]. Collectively, these factors are likely to contribute to nurse stress [5, 6] and, consequently, poor sleep [43].

### 4.1. Strengths and Limitations

This study conducted a comprehensive systematic search that identified 280 studies representing 219,245 nurses across 45 countries, alongside statistical adjustment for potential covariates. Despite the strengths of this study, there are several limitations. Trend estimates were derived from aggregate study‐level data presented in primary research written in English involving voluntary participants who provided self‐report estimates of sleep duration and/or quality, reported as a continuous measure. As such, the results of this study reflect population‐level trends that might not be representative of the general nursing population and are subject to sources of bias (e.g., language, publication and self‐report bias) and the possibility of ecological fallacy. While the inclusion of only English‐language studies may have excluded relevant studies published in other languages, evidence suggests that restricting systematic reviews to English‐language studies has minimal impact on overall effect estimates and conclusions [50, 51]. Also, since most studies reported on nurses from the WPR, results may be limited in terms of global generalisability and the wider international workforce.

Further, our analyses were designed to assess population‐level temporal trends rather than pooled ESs or between‐study heterogeneity. Accordingly, fixed‐effects models were used instead of random‐effects meta‐regression approaches, although residual between‐study variability and unmeasured clustering may still have influenced the trend estimates. Additionally, modelling year as continuous and decade‐based variables may not have fully captured complex nonlinear temporal patterns.

Due to the exploratory and descriptive aim of this review, with 280 included studies that varied in study design, a formal risk of bias assessment was not undertaken as a single critical appraisal tool would not have been appropriate. This is an important methodological consideration and limitation of this review.

It is also important to recognise that although sleep duration and quality were treated as distinct measures, they may overlap with each other and possibly reflect other sleep‐related constructs. For example, measures of sleep duration may reflect sleep satisfaction [11, 52], while measures of sleep quality using the PSQI are derived from a questionnaire that includes a subscale related to sleep duration. Further, although the PSQI is a widely used measure of sleep quality, other measures of sleep quality exist, and different assessment measures may yield different trend estimates. While we used a consistent validated tool to assess sleep quality, we relied on the peer‐review process for cross‐cultural validation and did not independently screen each study for measurement invariance or validation procedures. Accordingly, temporal trend estimates may, in part, reflect differences in measurement approaches. It is also important to recognise that although temporal trends may be unique for different nursing specialities, we were unable to explore this factor as many studies did not clearly report this information. Similarly, since definitions for night work varied widely across studies, we are limited in our confidence with which ‘night work’ can be interpreted as a contributing factor. We also interpolated estimates when key data (e.g., year of measurement) were missing, which may have induced bias. Finally, this study examined rates of temporal change in sleep outcomes rather than absolute sleep levels; therefore, we cannot determine whether nurses consistently slept less than recommended by advisory bodies. Lastly, it is important to recognise that data examined in this study were retrieved from a systematic search last conducted in 2024. As such, ongoing surveillance may be needed, including future updates to this review.

### 4.2. Implications for Practise and Research

Temporal declines identified in both sleep duration and quality suggest that nurse managers should consider sleep as a potential factor affecting workforce well‐being. The findings of this study also suggest a need for strategies, evidence‐based recommendations and interventions to promote and support healthy sleep among nurses. These may include, but are not limited to, emphasising the importance of healthy sleep within fatigue risk management strategies, designing rosters that consider sleep preferences, limiting quick‐return shifts which restrict opportunities for sleep and monitoring overtime. In addition to these strategies, it may also be important to integrate sleep education within workforce well‐being programmes and provide staff with sleep‐health education.

Efforts to better understand the unique perceptions, behaviours and experiences of sleep among nurses may also help inform the mechanisms driving the observed decline and support the development of targeted interventions that effectively promote healthy sleep. This information may be best utilised through collaborative approaches involving individual nurses, their institutions and possibly within specific nursing subspecialities. A recent systematic review identified a range of interventions designed to improve nurses’ sleep, including light therapy, dietary supplements, aromatherapy, education and strategic napping, although definitive conclusions regarding effectiveness could not be drawn [53]. While the interventions examined in this review were predominantly person‐centred approaches, the authors acknowledged the importance of workplace contexts, including administrative support and workplace environments, noting that feasibility, facilitators and barriers to implementation need to be considered to achieve sustainable and effective workplace interventions [53]. Attention to organisational factors may also help improve sleep. This has been identified by previous authors, including setting mandatory limits on overtime and ensuring roster schedules to allow enough time for not only sleep but also for travel home and presleep recovery activities [54, 55]. The effectiveness of these approaches remains uncertain in nurse populations, and future research is needed to explore the mechanisms driving these trends to ensure that interventions are targeted, evidence‐based and effective.

## 5. Conclusion

This study identified that over the past 3 decades, there have been significant declines in nurses’ sleep duration and quality. The observed temporal findings highlight the importance of continued monitoring and organisational strategies that support nurses’ sleep health and workplace well‐being.

## Author Contributions

Leanna G. Santostefano: methodology, investigation, data curation, validation, project administration, writing–original draft, writing–review and editing and visualization.

Kurt Lushington: methodology, investigation, writing–review and editing, visualization and supervision.

Siobhan Banks: methodology, writing–review and editing and supervision.

Grant R. Tomkinson: methodology, formal analysis, visualization, writing–review and editing and supervision.

Justin J. Lang: methodology, software, formal analysis, visualization, writing–review and editing and supervision.

Kristin Graham: methodology, investigation, data curation and writing–review and editing.

Hayley Ruf, Kiriaki Stewart, Kirstie Howland, Michelle Headland and Michael Musker: methodology, investigation, data curation and writing–review and editing.

Lisa Matricciani: conceptualization, methodology, investigation, data curation, writing–original draft, writing–review and editing, visualization and supervision.

## Funding

This research did not receive any funding. Open access publishing facilitated by Adelaide University, as part of the Wiley ‐ Adelaide University agreement via the Council of Australasian University Librarians.

## Conflicts of Interest

The authors declare no conflicts of interest.

## Supporting Information

Additional supporting information can be found online in the Supporting Information section.

## Supporting information


**Supporting Information 1** Supporting File 1: The search strategies formulated for each database.

**Supporting Information 2** Supporting File 2: Data extraction protocol.

**Supporting Information 3** Supporting File 3: Excluded studies and reasons.

**Supporting Information 4** Supporting File 4: Reference list of all included studies.

**Supporting Information 5** Supporting File 5: Extracted data of all included studies.

## Data Availability

Data are available in supporting files.
