# Supplementary material for: Trends in Nurses’ Sleep: A Meta‐Regression Involving 219,245 Nurses From 45 Countries
Source: J Nurs Manag. 2026 Aug 27;2026:9051680. doi: 10.1155/jonm/9051680 (PMC13522381; doi:10.1155/jonm/9051680)
Supplement: Supplementary file 1 [file JONM-2026-9051680-s001.docx]

**Supplementary File 4: Reference list of all included studies**

1. Abasi, S., Akbari, H., & Sabery, M. (2022). The effects of acupressure on sleep quality and compassion fatigue among emergency and critical care nurses during the coronavirus disease 2019 pandemic: A clinical trial. *Nursing and Midwifery Studies*, *11*(2), 79-84. [https://doi.org/https://dx.doi.org/10.4103/nms.nms_97_21](https://doi.org/https:/dx.doi.org/10.4103/nms.nms_97_21)
2. Akbarzadeh, H., Khezri, H. D., Mahmudi, G., Jahani, M. A., Tahmasebi, H., & Zeydi, A. E. (2014). Sleep quality among Iranian nurses working in intensive care units versus general wards: A cross-sectional study. *International Journal of Caring Sciences*, *7*(3), 930-935.
3. Al Maqbali, M. (2021). Sleep disturbance among frontline nurses during the COVID-19 pandemic. *Sleep and Biological Rhythms*, *19*(4), 467-473. [https://doi.org/https://dx.doi.org/10.1007/s41105-021-00337-6](https://doi.org/https:/dx.doi.org/10.1007/s41105-021-00337-6)
4. Al-Hammouri, M. M., & Rababah, J. A. (2024). A brief mindfulness-based intervention, sleep quality, sleep duration, and fatigue among nurses: A randomized controlled trial. *Journal of clinical psychology*. [https://doi.org/https://dx.doi.org/10.1002/jclp.23677](https://doi.org/https:/dx.doi.org/10.1002/jclp.23677)
5. Alameri, R. A., Almulla, H. A., Al Swyan, A. H., & Hammad, S. S. (2024). Sleep quality and fatigue among nurses working in high-acuity clinical settings in Saudi Arabia: a cross-sectional study. *BMC Nursing*, *23*(1), 51. [https://doi.org/https://dx.doi.org/10.1186/s12912-023-01693-z](https://doi.org/https:/dx.doi.org/10.1186/s12912-023-01693-z)
6. Alaradi, F. A., Priya, G., & Al-Darazi, F. (2023). Shift Changes' Effects on Sleep Quality and Work Performance among Nurses Working in Bahrain's Intensive Care Units. *Journal of the Dow University of Health Sciences*, *17*(1), 3-10.<https://doi.org/10.36570/jduhs.2023.1.1723>
7. Aldemir, S., & Tasdemir Yigitoglu, G. (2024). The Relation Between Sleep Quality and Anger Expression That Occur in Nurses: A Cross-Sectional Study. *Journal of Radiology Nursing*, *43*(1), 60-67. [https://doi.org/https://dx.doi.org/10.1016/j.jradnu.2023.10.003](https://doi.org/https:/dx.doi.org/10.1016/j.jradnu.2023.10.003)
8. Alharbi, W. I., Mahrus, A., Elsayed, S. A., & Alolayan, A. B. (2021). Sleep quality among nurses during the first year of coronavirus disease 2019. *Open Access Macedonian Journal of Medical Sciences*, *9*, 128-134. [https://doi.org/https://dx.doi.org/10.3889/oamjms.2021.6923](https://doi.org/https:/dx.doi.org/10.3889/oamjms.2021.6923)
9. Alimoglu, M. K., & Donmez, L. (2005). Daylight exposure and the other predictors of burnout among nurses in a University Hospital. *International Journal of Nursing Studies*, *42*(5), 549-555.<https://doi.org/10.1016/j.ijnurstu.2004.09.001>
10. Aliyu, I., Ibrahim, Z. F., Teslim, L. O., Okhiwu, H., Peter, I. D., & Michael, G. C. (2017). Sleep quality among nurses in a tertiary hospital in North-West Nigeria. *The Nigerian postgraduate medical journal*, *24*(3), 168-173. [https://doi.org/https://dx.doi.org/10.4103/npmj.npmj_79_17](https://doi.org/https:/dx.doi.org/10.4103/npmj.npmj_79_17)
11. Almhdawi, K. A., Alrabbaie, H., Kanaan, S. F., Oteir, A. O., Jaber, A. F., Ismael, N. T., & Obaidat, D. S. (2020). Predictors and prevalence of lower quadrant work-related musculoskeletal disorders among hospital-based nurses: A cross-sectional study. *Journal of Back and Musculoskeletal Rehabilitation*, *33*(6), 885-896. [https://doi.org/https://dx.doi.org/10.3233/BMR-191815](https://doi.org/https:/dx.doi.org/10.3233/BMR-191815)
12. Alward, R. R., & Monk, T. H. (1990). A comparison of rotating-shift and permanent night nurses. *International Journal of Nursing Studies, 27*(3), 297-302.<https://doi.org/10.1016/0020-7489(90)90044-j>
13. Andreassen, C. S., Pallesen, S., Moen, B. E., Bjorvatn, B., Waage, S., & Schaufeli, W. B. (2018). Workaholism and negative work-related incidents among nurses. *Industrial health*, *56*(5), 373-381. [https://doi.org/https://dx.doi.org/10.2486/indhealth.2017-0223](https://doi.org/https:/dx.doi.org/10.2486/indhealth.2017-0223)
14. Apellido, R. (2018). Night shift work and weight gain among female Filipino nurses.*The Sciences and Engineering*, *79*(3).
15. Arimura, M., Imai, M., Okawa, M., Fujimura, T., & Yamada, N. (2010). Sleep, Mental Health Status, and Medical Errors among Hospital Nurses in Japan. *Industrial health*, *48*(6), 811-817.<https://doi.org/10.2486/indhealth.MS1093>
16. Assadi, T., Sadeghi, F., Noyani, A., SeidAbadi, A. M., & Yekesadat, S. M. (2019). Occupational Burnout and Its Related Factors Among Iranian Nurses: A Cross-Sectional Study in Shahroud, Northeast of Iran. *Open Access Macedonian Journal of Medical Sciences*, *7*(17), 2902-2907.<https://doi.org/10.3889/oamjms.2019.744>
17. Aydin Sayilan, A., Kulakac, N., & Uzun, S. (2021). Burnout levels and sleep quality of COVID-19 heroes. *Perspectives in psychiatric care*, *57*(3), 1231-1236. [https://doi.org/https://dx.doi.org/10.1111/ppc.12678](https://doi.org/https:/dx.doi.org/10.1111/ppc.12678)
18. Baek, J., Han, K., & Choi-Kwon, S. (2020). Sleep diary- and actigraphy-derived sleep parameters of 8-hour fast-rotating shift work nurses: A prospective descriptive study. *International Journal of Nursing Studies*, *112*, 103719. [https://doi.org/https://dx.doi.org/10.1016/j.ijnurstu.2020.103719](https://doi.org/https:/dx.doi.org/10.1016/j.ijnurstu.2020.103719)
19. Balamadhuwanthi, S., Latha, R., Vedapriya, D. R., Tamilselvan, K., & Mathangi, D. C. (2023). Effect of chair yoga on heart rate variability, perceived stress, and sleep quality among nursing professionals from a tertiary care hospital. *Biomedicine (India)*, *43*(1), 499-503. [https://doi.org/https://dx.doi.org/10.51248/.V43I01.2292](https://doi.org/https:/dx.doi.org/10.51248/.V43I01.2292)
20. Bamonti, P., Conti, E., Cavanagh, C., Gerolimatos, L., Gregg, J., Goulet, C., Pifer, M., & Edelstein, B. (2019). Coping, Cognitive Emotion Regulation, and Burnout in Long-Term Care Nursing Staff: A Preliminary Study. *Journal of Applied Gerontology*, *38*(1), 92-111. [https://doi.org/https://dx.doi.org/10.1177/0733464817716970](https://doi.org/https:/dx.doi.org/10.1177/0733464817716970)
21. Barak, Y., Achiron, A., Lampl, Y., Gilad, R., Ring, A., Elizur, A., & Sarova-Pinhas, I. (1995). Sleep disturbances among female nurses: Comparing shift to day work. *Chronobiology international*, *12*(5), 345-350.<https://doi.org/10.3109/07420529509057283>
22. Bayoumi, M., Ahmed, S., & Abdelgayed, A. (2020). Impact of Nurses' Poor Sleep Hygiene on Patient's Care at ICUS. *Pakistan Journal of Medical and Health Sciences*, *14*(2), 972-976.
23. Bazrafshan, M., Moravveji, F., Soleymaninejad, N., Zare, F., Rahimpoor, R., & Zolghadr, R. (2018). Prevalence and effects of sleep disorders among shift-working nurse. *Annals of Tropical Medicine and Public Health*, *11*(1), 13-18.
24. Beebe, D., Chang, J. J., Kress, K., & Mattfeldt-Beman, M. (2017). Diet quality and sleep quality among day and night shift nurses. *Journal of Nursing Management*, *25*(7), 549-557. [https://doi.org/https://dx.doi.org/10.1111/jonm.12492](https://doi.org/https:/dx.doi.org/10.1111/jonm.12492)
25. Berkman, L. F., Liu, S. Y., Hammer, L., Moen, P., Klein, L. C., Kelly, E., Fay, M., Davis, K., Durham, M., Karuntzos, G., & Buxton, O. M. (2015). Work-family conflict, cardiometabolic risk, and sleep duration in nursing employees. *Journal of Occupational Health Psychology*, *20*(4), 420-433. [https://doi.org/https://dx.doi.org/10.1037/a0039143](https://doi.org/https:/dx.doi.org/10.1037/a0039143)
26. Bharadwaj, A. (2023). A Correlation between Sleep Deprivation and Cognitive Performance in Nurses Working in Shifts in SRG and SHKBM (Janana) Hospital, Jhalawar Medical College. *International Journal of Current Pharmaceutical Review and Research*, *15*(6), 311-318.
27. Bilgic, S., Celikkalp, U., & Misirli, C. (2021). Stress level and sleep quality of nurses during the COVID-19 pandemic. *Work (Reading, Mass.)*, *70*(4), 1021-1029. [https://doi.org/https://dx.doi.org/10.3233/WOR-210538](https://doi.org/https:/dx.doi.org/10.3233/WOR-210538)
28. Bjorvatn, B., Dale, S., Hogstad-Erikstein, R., Fiske, E., Pallesen, S., & Waage, S. (2012). Self-reported sleep and health among Norwegian hospital nurses in intensive care units. *Nursing in Critical Care*, *17*(4), 180-188. [https://doi.org/https://dx.doi.org/10.1111/j.1478-5153.2012.00504.x](https://doi.org/https:/dx.doi.org/10.1111/j.1478-5153.2012.00504.x)
29. Boned-Galán, Á., López-Ibort, N., & Gascón-Catalán, A. (2022). Sleep disturbances in nurse managers during the early and late stages of the COVID-19 pandemic. *Frontiers in Psychology*, *13*.<https://doi.org/10.3389/fpsyg.2022.1070355>
30. Botti, S., Cannici, C., Liptrott, S. J., de Cecco, V., Rostagno, E., Gargiulo, G., Orlando, L., Caime, A., Samarani, E., Galgano, L., Cioce, M., Mordini, N., Mandelli, N. E., Tombari, L., Errichiello, S., Celon, N., Lupo, R., Rea, T., & Serra, N. (2022). Nurses' Physical and Psychological Symptoms during the first COVID-19 Lockdown in Italy: A Nationwide Cross-Sectional Study in Stem Cell Transplantation Setting. *Mediterranean Journal of Hematology and Infectious Diseases*, *14*(1), 2022010. [https://doi.org/https://dx.doi.org/10.4084/MJHID.2022.010](https://doi.org/https:/dx.doi.org/10.4084/MJHID.2022.010)
31. Bracci, M., Manzella, N., Copertaro, A., Staffolani, S., Strafella, E., Barbaresi, M., Copertaro, B., Rapisarda, V., Valentino, M., & Santarelli, L. (2014). Rotating-Shift Nurses after a Day off: Peripheral Clock Gene Expression, Urinary Melatonin, and Serum 17-β-Estradiol Levels. *Scandinavian Journal of Work, Environment ＆ Health*, *40*(3), 295-304.<https://doi.org/10.5271/sjweh.3414>
32. Celik, A. S., & Yarali, S. (2023). The Effect of Laughter Yoga on the Psychological Resilience and Sleep Quality of Nurses During the Pandemic: A Randomized Controlled Trial. *Alternative Therapies in Health and Medicine*, *29*(5), 146-152.
33. Chaiard, J., Deeluea, J., Suksatit, B., Songkham, W., & Inta, N. (2018). Short sleep duration among Thai nurses: Influences on fatigue, daytime sleepiness, and occupational errors. *Journal of Occupational Health*, *60*(5), 348-355. [https://doi.org/https://dx.doi.org/10.1539/joh.2017-0258-OA](https://doi.org/https:/dx.doi.org/10.1539/joh.2017-0258-OA)
34. Chang, S. J., & Jang, S. J. (2019). Social jetlag and quality of life among nursing students: A cross‐sectional study. *Journal of Advanced Nursing*, *75*(7), 1418-1426.<https://doi.org/10.1111/jan.13857>
35. Chang, W.-P., & Chang, Y.-P. (2019). Relationship between job satisfaction and sleep quality of female shift-working nurses: using shift type as moderator variable. *Industrial health*, *57*(6), 732-740. [https://doi.org/https://dx.doi.org/10.2486/indhealth.2018-0258](https://doi.org/https:/dx.doi.org/10.2486/indhealth.2018-0258)
36. Chang, W.-P., & Li, H.-B. (2019). Differences in workday sleep fragmentation, rest-activity cycle, sleep quality, and activity level among nurses working different shifts. *Chronobiology international*, *36*(12), 1761-1771. [https://doi.org/https://dx.doi.org/10.1080/07420528.2019.1681441](https://doi.org/https:/dx.doi.org/10.1080/07420528.2019.1681441)
37. Chang, W.-P., & Yang, C.-M. (2020). Influence of sleep-wake cycle on body mass index in female shift-working nurses with sleep quality as mediating variable. *Industrial health*, *58*(2), 161-169. [https://doi.org/https://dx.doi.org/10.2486/indhealth.2019-0066](https://doi.org/https:/dx.doi.org/10.2486/indhealth.2019-0066)
38. Chang, Y.-S., Wu, Y.-H., Lu, M. R., Hsu, C.-Y., Liu, C.-K., & Hsu, C. (2015). Did a brief nap break have positive benefits on information processing among nurses working on the first 8-h night shift? *Applied Ergonomics*, *48*, 104-108. [https://doi.org/https://dx.doi.org/10.1016/j.apergo.2014.11.005](https://doi.org/https:/dx.doi.org/10.1016/j.apergo.2014.11.005)
39. Chang, Y.-Y., Lin, C.-L., & Chang, L.-Y. (2017). The Effects of Aromatherapy Massage on Sleep Quality of Nurses on Monthly Rotating Night Shifts. *Evidence-based complementary and alternative medicine: eCAM*, *2017*, 3861273. [https://doi.org/https://dx.doi.org/10.1155/2017/3861273](https://doi.org/https:/dx.doi.org/10.1155/2017/3861273)
40. Chappel, S. E., Aisbett, B., Considine, J., & Ridgers, N. D. (2020). Emergency nurses' activity levels across rotating shifts. *Australasian Emergency Care, 23*(3), 203-210.<https://doi.org/10.1016/j.auec.2020.03.001>
41. Chatha, M. H., Khan, S. A., Nazir Ahmed, W., Khan, A., Abu Bakar, M., & Asif, M. (2022). A Mental Health Survey of Medical Professionals Working During the COVID-19 Pandemic at Shaukat Khanum Cancer Hospital, Lahore. *Cureus*, *14*(9), 28869. [https://doi.org/https://dx.doi.org/10.7759/cureus.28869](https://doi.org/https:/dx.doi.org/10.7759/cureus.28869)
42. Chen, R., Chou, K.-R., Huang, Y.-J., Wang, T.-S., Liu, S.-Y., & Ho, L.-Y. (2006). Effects of a SARS prevention programme in Taiwan on nursing staff's anxiety, depression and sleep quality: A longitudinal survey. *International Journal of Nursing Studies*, *43*(2), 215-225. [https://doi.org/https://dx.doi.org/10.1016/j.ijnurstu.2005.03.006](https://doi.org/https:/dx.doi.org/10.1016/j.ijnurstu.2005.03.006)
43. Chen, R., Fang, P., Tan, L., Li, J., & Yu, L. (2022). Factors associated with sleep quality among nurse managers during regular prevention and control of the COVID-19 in China: a cross-sectional study. *BMC Nursing*, *21*(1), 363-363.<https://doi.org/10.1186/s12912-022-01149-w>
44. Chiang, S.-L., Chiang, L.-C., Tzeng, W.-C., Lee, M.-S., Fang, C.-C., Lin, C.-H., & Lin, C.-H. (2022). Impact of Rotating Shifts on Lifestyle Patterns and Perceived Stress among Nurses: A Cross-Sectional Study. *International journal of environmental research and public health*, *19*(9). [https://doi.org/https://dx.doi.org/10.3390/ijerph19095235](https://doi.org/https:/dx.doi.org/10.3390/ijerph19095235)
45. Chien, P.-L., Su, H.-F., Hsieh, P.-C., Siao, R.-Y., Ling, P.-Y., & Jou, H.-J. (2013). Sleep Quality among Female Hospital Staff Nurses. *Sleep disorders*, *2013*, 283490. [https://doi.org/https://dx.doi.org/10.1155/2013/283490](https://doi.org/https:/dx.doi.org/10.1155/2013/283490)
46. Chin, W., Guo, Y. L., Hung, Y. J., Yang, C. Y., & Shiao, J. S. C. (2015). Short sleep duration is dose-dependently related to job strain and burnout in nurses: a cross sectional survey. *International Journal of Nursing Studies*, *52*(1), 297-306. [https://doi.org/https://dx.doi.org/10.1016/j.ijnurstu.2014.09.003](https://doi.org/https:/dx.doi.org/10.1016/j.ijnurstu.2014.09.003)
47. Chin, W.-S., Chen, Y.-C., Lin, T.-T., Guo, Y.-L. L., & Shiao, J. S. C. (2021). Short sleep and chronic neck and shoulder discomfort in nurses. *Journal of Occupational Health*, *63*(1), 12236. [https://doi.org/https://dx.doi.org/10.1002/1348-9585.12236](https://doi.org/https:/dx.doi.org/10.1002/1348-9585.12236)
48. Cho, O.-H., Yoon, J., & Kim, M. (2023). Influencing factors of fatigue among public health nurses during the COVID-19 pandemic: A cross-sectional study. *Public Health Nursing*, *40*(1), 80-89. [https://doi.org/https://dx.doi.org/10.1111/phn.13131](https://doi.org/https:/dx.doi.org/10.1111/phn.13131)
49. Cho, S., Park, W.-J., Kang, W., Lim, H.-M., Ahn, J.-S., Lim, D.-Y., & Moon, J.-D. (2019). The association between serum dehydroepiandrosterone sulfate (DHEAS) levels and job-related stress among female nurses. *Annals of occupational and environmental medicine*, *31*, 18. [https://doi.org/https://dx.doi.org/10.35371/aoem.2019.31.e18](https://doi.org/https:/dx.doi.org/10.35371/aoem.2019.31.e18)
50. Christina, S., & Konjengbam, S. (2019). Sleep quality among nurses in a tertiary hospital in Manipur. *Journal of Medical Society*, *33*(3), 146-151. [https://doi.org/https://dx.doi.org/10.4103/jms.jms_80_20](https://doi.org/https:/dx.doi.org/10.4103/jms.jms_80_20)
51. Chueh, K.-H., Chen, K.-R., & Lin, Y.-H. (2021). Psychological distress and sleep disturbance among female nurses: Anxiety or depression? *Journal of Transcultural Nursing*, *32*(1), 14-20. [https://doi.org/https://dx.doi.org/10.1177/1043659619881491](https://doi.org/https:/dx.doi.org/10.1177/1043659619881491)
    a. Colak, M., & Esin, M. N. (2024). Factors affecting the psychomotor vigilance of nurses working night shift. *International nursing review*, *71*(1), 84-93.
52. Cox, S. D., Benoit, J. S., Brohard, C. L., & McIntyre, T. M. (2021). Evaluation of sleep quality among nursing faculty: Application of the Pittsburgh Sleep Quality Index-A descriptive correlational study. *Nursing open, 9*(1), 339-348. [https://doi.org/https://dx.doi.org/10.1002/nop2.1067](https://doi.org/https:/dx.doi.org/10.1002/nop2.1067)
53. Cyr, M., Artenie, D. Z., Al Bikaii, A., Lee, V., Raz, A., & Olson, J. A. (2023). An evening light intervention reduces fatigue and errors during night shifts: A randomized controlled trial. *Sleep health*, *9*(3), 373-380. [https://doi.org/https://dx.doi.org/10.1016/j.sleh.2023.02.004](https://doi.org/https:/dx.doi.org/10.1016/j.sleh.2023.02.004)
54. da Silva, K. K. M., De Martino, M. M. F., Viana, M. C. O., Bezerra, C. M. B., & Miranda, F. A. N. (2017). Relationship between work shifts and quality of sleep of nurses: A descriptive study. *Online Brazilian Journal of Nursing*, *16*(1), 57-63. [https://doi.org/https://dx.doi.org/10.17665/1676-4285.20175577](https://doi.org/https:/dx.doi.org/10.17665/1676-4285.20175577)
55. Dagcan, N., & Arslan, S. (2021). SLEEP QUALITY AND BODY MASS INDEX OF NURSES: A CROSS-SECTIONAL STUDY. *Central European Journal of Nursing and Midwifery*, *12*(4), 530-536. [https://doi.org/https://dx.doi.org/10.15452/CEJNM.2021.12.0030](https://doi.org/https:/dx.doi.org/10.15452/CEJNM.2021.12.0030)
56. Dai, C., Hu, P., Yan, F., He, X., Cheng, W., Yu, L., Fang, A., Meng, X., Lou, M., Chen, Y., Chi, D., Zhou, H., Chen, Q., Fang, Z., Ni, S., & Huang, Q. (2023). The mediating role of sleep quality in the relationship between dispositional mindfulness and fatigue in Chinese nurses during the COVID-19 pandemic. *BMC Nursing*, *22*(1), 471. [https://doi.org/https://dx.doi.org/10.1186/s12912-023-01642-w](https://doi.org/https:/dx.doi.org/10.1186/s12912-023-01642-w)
57. Dai, C., Qiu, H., Huang, Q., Hu, P., Hong, X., Tu, J., Xie, Q., Li, H., Ren, W., Ni, S., & Chen, F. (2019). The effect of night shift on sleep quality and depressive symptoms among Chinese nurses. *Neuropsychiatric Disease and Treatment*, *15*, 435-440. [https://doi.org/https://dx.doi.org/10.2147/NDT.S190689](https://doi.org/https:/dx.doi.org/10.2147/NDT.S190689)
58. De Martino, M. M. F., Abreu, A. C. B., Barbosa, M. F. d. S., & Teixeira, J. E. M. (2013). The relationship between shift work and sleep patterns in nurses. *Ciencia & saude coletiva*, *18*(3), 763-768. [https://doi.org/https://dx.doi.org/10.1590/s1413-81232013000300022](https://doi.org/https:/dx.doi.org/10.1590/s1413-81232013000300022)
59. Debbia, F., Medina-Valverde, M. J., Garcia-Arcos, A., Garrido-Gutierrez, A., Rodnguez-Borrego, M. A., & Lopez-Soto, P. J. (2021). Chronotype, general health and quality of sleep in a population of Spanish nurses. *Revista da Escola de Enfermagem*, *55*, 1-8. [https://doi.org/https://dx.doi.org/10.1590/S1980-220X2020010903752](https://doi.org/https:/dx.doi.org/10.1590/S1980-220X2020010903752)
60. Demir, G., & Karadag, G. (2024). The Relationship Between Nurses' Sleep Quality and Their Tendency to Commit Medical Errors. *Sleep Science*, *17*(1), 7-15. [https://doi.org/https://dx.doi.org/10.1055/s-0043-1776753](https://doi.org/https:/dx.doi.org/10.1055/s-0043-1776753)
61. Deng, X., Fang, R., & Cai, Y. (2021). Evaluation of the correlation between effort-reward imbalance and sleep quality among community health workers. *BMC health services research*, *21*(1), 490. [https://doi.org/https://dx.doi.org/10.1186/s12913-021-06526-w](https://doi.org/https:/dx.doi.org/10.1186/s12913-021-06526-w)
62. Di Muzio, M., Diella, G., Di Simone, E., Novelli, L., Alfonsi, V., Scarpelli, S., Annarumma, L., Salfi, F., Pazzaglia, M., Giannini, A. M., & De Gennaro, L. (2020). Nurses and Night Shifts: Poor Sleep Quality Exacerbates Psychomotor Performance. *Frontiers in Neuroscience*, *14*, 579938. [https://doi.org/https://dx.doi.org/10.3389/fnins.2020.579938](https://doi.org/https:/dx.doi.org/10.3389/fnins.2020.579938)
63. Di Muzio, M., Diella, G., Di Simone, E., Pazzaglia, M., Alfonsi, V., Novelli, L., Cianciulli, A., Scarpelli, S., Gorgoni, M., Giannini, A., Ferrara, M., Lucidi, F., & De Gennaro, L. (2021). Comparison of Sleep and Attention Metrics Among Nurses Working Shifts on a Forward- vs Backward-Rotating Schedule. *JAMA network open*, *4*(10), e2129906. [https://doi.org/https://dx.doi.org/10.1001/jamanetworkopen.2021.29906](https://doi.org/https:/dx.doi.org/10.1001/jamanetworkopen.2021.29906)
64. Ding, C., Li, L., Li, G., Li, X., Xie, L., & Duan, Z. (2023). Impact of workplace violence against psychological health among nurse staff from Yunnan-Myanmar Chinese border region: propensity score matching analysis. *BMC Nursing*, *22*(1), 242. [https://doi.org/https://dx.doi.org/10.1186/s12912-023-01402-w](https://doi.org/https:/dx.doi.org/10.1186/s12912-023-01402-w)
65. Dong, H., Zhang, Q., Sun, Z., Sang, F., & Xu, Y. (2017). Sleep problems among Chinese clinical nurses working in general hospitals. *Occupational medicine (Oxford, England)*, *67*(7), 534-539. [https://doi.org/https://dx.doi.org/10.1093/occmed/kqx124](https://doi.org/https:/dx.doi.org/10.1093/occmed/kqx124)
66. Dong, H., Zhang, Q., Zhang, Z., & Zhu, C. (2022). Association of sleep disturbance with shift work, occupational stress, and musculoskeletal pain in Chinese public hospital emergency workers: A multicentre cross-sectional study. *Chronobiology international*, *39*(6), 886-894. [https://doi.org/https://dx.doi.org/10.1080/07420528.2022.2048662](https://doi.org/https:/dx.doi.org/10.1080/07420528.2022.2048662)
67. Dong, H., Zhang, Q., Zhu, C., & Lv, Q. (2020). Sleep quality of nurses in the emergency department of public hospitals in China and its influencing factors: A cross-sectional study. *Health and Quality of Life Outcomes*, *18*(1), 116. [https://doi.org/https://dx.doi.org/10.1186/s12955-020-01374-4](https://doi.org/https:/dx.doi.org/10.1186/s12955-020-01374-4)
68. Donmezdil, S., & Arac, S. (2021). Effect of shift work in intensive care on attention disorder in nurses. *International Journal of Clinical Practice*, *75*(4), 13774. [https://doi.org/https://dx.doi.org/10.1111/ijcp.13774](https://doi.org/https:/dx.doi.org/10.1111/ijcp.13774)
69. Dorrian, J., Paterson, J., Dawson, D., Pincombe, J., Grech, C., & Rogers, A. E. (2011). Sleep, stress and compensatory behaviors in Australian nurses and midwives. *Revista de saude publica*, *45*(5), 922-930. [https://doi.org/https://dx.doi.org/10.1590/s0034-89102011005000059](https://doi.org/https:/dx.doi.org/10.1590/s0034-89102011005000059)
70. Duan, X., Wu, Q., Zhu, X., & Shi, Y. (2017). Improving sleep quality relieves occupational stress in nurses of cardiac surgical intensive care unit. *Biomedical Research (India)*, *28*(9), 3934-3940.
71. Ebrahimian, A., Fakhr-Movahedi, A., & Hashemi-Amrei, S.-H. (2023). The relationship between the emergency nurses' sleep quality and the sleep quality of their spouses: A cross-sectional descriptive-analytical study. *Health science reports*, *6*(1), 965. [https://doi.org/https://dx.doi.org/10.1002/hsr2.965](https://doi.org/https:/dx.doi.org/10.1002/hsr2.965)
72. Fang, J., Kunaviktikul, W., Olson, K., Chontawan, R., & Kaewthummanukul, T. (2008). Factors influencing fatigue in Chinese nurses. *Nursing and Health Sciences*, *10*(4), 291-299. [https://doi.org/https://dx.doi.org/10.1111/j.1442-2018.2008.00407.x](https://doi.org/https:/dx.doi.org/10.1111/j.1442-2018.2008.00407.x)
73. Fang, R., & Li, X. (2015). A regular yoga intervention for staff nurse sleep quality and work stress: a randomised controlled trial. *Journal of clinical nursing*, *24*(23), 3374-3379. [https://doi.org/https://dx.doi.org/10.1111/jocn.12983](https://doi.org/https:/dx.doi.org/10.1111/jocn.12983)
74. Fang, Y., Kang, X., Feng, X., Zhao, D., Song, D., & Li, P. (2019). Conditional effects of mindfulness on sleep quality among clinical nurses: the moderating roles of extraversion and neuroticism. *Psychology, health & medicine*, *24*(4), 481-492. [https://doi.org/https://dx.doi.org/10.1080/13548506.2018.1492731](https://doi.org/https:/dx.doi.org/10.1080/13548506.2018.1492731)
75. Farag, A., Moon, C., & Xiao, Q. (2021). Work and Personal Characteristics Associated with Sleep Behavior Among Acute Care Nurses. *Journal of Nursing Regulation*, *12*(1), 40-51. [https://doi.org/https://dx.doi.org/10.1016/S2155-8256%2821%2900019-3](https://doi.org/https:/dx.doi.org/10.1016/S2155-8256%2821%2900019-3)
76. Faraut, B., Cordina-Duverger, E., Aristizabal, G., Drogou, C., Gauriau, C., Sauvet, F., Levi, F., Leger, D., & Guenel, P. (2022). Immune disruptions and night shift work in hospital healthcare professionals: The intricate effects of social jetlag and sleep debt. *Frontiers in Immunology*, *13*(1*)*, 939829. [https://doi.org/https://dx.doi.org/10.3389/fimmu.2022.939829](https://doi.org/https:/dx.doi.org/10.3389/fimmu.2022.939829)
77. Fard, Z., Azadi, A., Veisani, Y., & Jamshidbeigi, A. (2020). The association between nurses' moral distress and sleep quality and their influencing factor in private and public hospitals in Iran. *Journal of Education and Health Promotion*, *9*(1).<https://doi.org/10.4103/jehp.jehp_291_20>
78. Farmer, H. R., Slavish, D. C., Ruiz, J., Dietch, J. R., Ruggero, C. J., Messman, B. A., Kelly, K., Kohut, M., & Taylor, D. J. (2022). Racial/ethnic variations in inflammatory markers: exploring the role of sleep duration and sleep efficiency. *Journal of behavioural medicine*, *45*(6), 855-867. [https://doi.org/https://dx.doi.org/10.1007/s10865-022-00357-8](https://doi.org/https:/dx.doi.org/10.1007/s10865-022-00357-8)
79. Feng, H.-l., Qi, X.-x., Xia, C.-L., Xiao, S.-q., & Fan, L. (2021). Association between night shift and sleep quality and health among Chinese nurses: A cross-sectional study. *Journal of Nursing Management*, *29*(7), 2123-2131. [https://doi.org/https://dx.doi.org/10.1111/jonm.13351](https://doi.org/https:/dx.doi.org/10.1111/jonm.13351)
80. Feng, T., Booth, B. M., Baldwin-Rodriguez, B., Osorno, F., & Narayanan, S. (2021). A multimodal analysis of physical activity, sleep, and work shift in nurses with wearable sensor data. *Scientific reports*, *11*(1), 8693. [https://doi.org/https://dx.doi.org/10.1038/s41598-021-87029-w](https://doi.org/https:/dx.doi.org/10.1038/s41598-021-87029-w)
81. Garcia-Tudela, A., Simonelli-Munoz, A. J., Jimenez-Rodriguez, D., Martinez Franco, P., Rivera-Caravaca, J. M., & Gallego-Gomez, J. I. (2023). Sleep analysis of hospital and out-of-hospital emergency professionals. *International emergency nursing*, *71*, 101352. [https://doi.org/https://dx.doi.org/10.1016/j.ienj.2023.101352](https://doi.org/https:/dx.doi.org/10.1016/j.ienj.2023.101352)
82. Garrido, A. L. F., Duarte, A. D. S., Santana, P. T., Rodrigues, G. H., Pellegrino, P., Nogueira, L. F. R., Cipolla-Neto, J., Moreno, C. R. D. C., & Marqueze, E. C. (2021). Eating habits, sleep, and a proxy for circadian disruption are correlated with dyslipidemia in overweight night workers. *Nutrition*, *83*(1), 111084. [https://doi.org/https://dx.doi.org/10.1016/j.nut.2020.111084](https://doi.org/https:/dx.doi.org/10.1016/j.nut.2020.111084)
83. Geiger-Brown, J., Rogers, V. E., Trinkoff, A. M., Kane, R. L., Bausell, R. B., & Scharf, S. M. (2012). Sleep, sleepiness, fatigue, and performance of 12-hour-shift nurses. *Chronobiology international*, *29*(2), 211-219. [https://doi.org/https://dx.doi.org/10.3109/07420528.2011.645752](https://doi.org/https:/dx.doi.org/10.3109/07420528.2011.645752)
84. Ghorbani, A., Momeni, M., Yekefallah, L., & Shahrokhi, A. (2023). The association between chronotype, sleep quality and medication errors among critical care nurses. *Chronobiology international*, *40*(11), 1480-1486. [https://doi.org/https://dx.doi.org/10.1080/07420528.2023.2256862](https://doi.org/https:/dx.doi.org/10.1080/07420528.2023.2256862)
85. Gomez-Garcia, T., Ruzafa-Martinez, M., Fuentelsaz-Gallego, C., Madrid, J. A., Rol, M. A., Martinez-Madrid, M. J., & Moreno-Casbas, T. (2016). Nurses' sleep quality, work environment and quality of care in the Spanish National Health System: Observational study among different shifts. *BMJ Open*, *6*(8), 012073. [https://doi.org/https://dx.doi.org/10.1136/bmjopen-2016-012073](https://doi.org/https:/dx.doi.org/10.1136/bmjopen-2016-012073)
86. Grundy, A., Sanchez, M., Richardson, H., Tranmer, J., Borugian, M., Graham, C. H., & Aronson, K. J. (2009). Light intensity exposure, sleep duration, physical activity, and biomarkers of melatonin among rotating shift nurses. *Chronobiology international*, *26*(7), 1443-1461. [https://doi.org/https://dx.doi.org/10.3109/07420520903399987](https://doi.org/https:/dx.doi.org/10.3109/07420520903399987)
87. Guardiano, M., Boy, P., Shapirshteyn, G., Dobrozdravic, L., Chen, L., Yang, H., Robbins, W., & Li, J. (2022). Working Conditions and Wellbeing among Prison Nurses during the COVID-19 Pandemic in Comparison to Community Nurses. *International journal of environmental research and public health*, *19*(17), 10955. [https://doi.org/https://dx.doi.org/10.3390/ijerph191710955](https://doi.org/https:/dx.doi.org/10.3390/ijerph191710955)
88. Güneysu, İ., Aydın, S., Aslan, E. A., Batmaz, S., & Güneysu, S. (2024). A comparison of sleep quality, quality of life, and psychological complaints of nurses working shift work and not working shift work in a university hospital. *Journal of Psychiatric Nursing*, *15*(1), 1-8.<https://doi.org/10.14744/phd.2023.98975>
89. Guofang, W., Zhang, W., & Zhu, Z. (2024). Effects of Psychological Intervention Based on the Self-Transcendence Theory on the Sleep Quality of Chinese Nurses. *Iranian Journal of Public Health*, *53*(4), 818-827.
90. Ha, Y., Lee, S.-H., Lee, D.-H., Kang, Y.-H., Choi, W., & An, J. (2022). Effectiveness of a Mobile Wellness Program for Nurses with Rotating Shifts during COVID-19 Pandemic: A Pilot Cluster-Randomized Trial. *International journal of environmental research and public health*, *19*(2). [https://doi.org/https://dx.doi.org/10.3390/ijerph19021014](https://doi.org/https:/dx.doi.org/10.3390/ijerph19021014)
91. Hajaghazadeh, M., Zamanzadeh, V., Ghofrani, M., & Orujlu, S. (2019). Morningness-eveningness chronotypes, sleep quality, and sleepiness among nurses. *Open Public Health Journal*, *12*(1), 414-419. [https://doi.org/https://dx.doi.org/10.2174/1874944501912010414](https://doi.org/https:/dx.doi.org/10.2174/1874944501912010414)
92. Hajo, S., Reed, J. L., Hans, H., Tulloch, H. E., Reid, R. D., & Prince, S. A. (2020). Physical activity, sedentary time and sleep and associations with mood states, shift work disorder and absenteeism among nurses: An analysis of the cross-sectional Champlain Nurses' Study. *PeerJ*, *2020*(3), 8464. [https://doi.org/https://dx.doi.org/10.7717/peerj.8464](https://doi.org/https:/dx.doi.org/10.7717/peerj.8464)
93. Haldar, P., & Sahu, S. (2015). Occupational stress and work efficiency of nursing staff engaged in rotating shift work. *Biological Rhythm Research*, *46*(4), 511-522. [https://doi.org/https://dx.doi.org/10.1080/09291016.2015.1021153](https://doi.org/https:/dx.doi.org/10.1080/09291016.2015.1021153)
94. Han, K., Kim, Y.-H., Lee, H. Y., Cho, H., & Jung, Y. S. (2019). Changes in health behaviours and health status of novice nurses during the first 2 years of work. *Journal of Advanced Nursing*, *75*(8), 1648-1656. [https://doi.org/https://dx.doi.org/10.1111/jan.13947](https://doi.org/https:/dx.doi.org/10.1111/jan.13947)
95. Han, Y., Yuan, Y., Zhang, L., & Fu, Y. (2016). Sleep disorder status of nurses in general hospitals and its influencing factors. *Psychiatria Danubina*, *28*(2), 176-183.
96. Hansen, A. M., Garde, A. H., & Hansen, J. (2006). Diurnal urinary 6-sulfatoxymelatonin levels among healthy Danish nurses during work and leisure time. *Chronobiology international*, *23*(6), 1203-1215. [https://doi.org/https://dx.doi.org/10.1080/07420520601100955](https://doi.org/https:/dx.doi.org/10.1080/07420520601100955)
97. Haseli, A., Egdampur, F., Qaderi, K., Kaffashian, M. R., & Delpisheh, A. (2023). Nurses and midwives' sleep quality and its associated factors during the early COVID-19 pandemic in Iran. *Heliyon*, *9*(4), 15068. [https://doi.org/https://dx.doi.org/10.1016/j.heliyon.2023.e15068](https://doi.org/https:/dx.doi.org/10.1016/j.heliyon.2023.e15068)
98. Hausswirth, C., Nesi, X., Dubois, A., Duforez, F., Rougier, Y., & Slattery, K. (2022). Four Weeks of a Neuro-Meditation Program Improves Sleep Quality and Reduces Hypertension in Nursing Staff During the COVID-19 Pandemic: A Parallel Randomized Controlled Trial. *Frontiers in Psychology*, *13*.<https://doi.org/10.3389/fpsyg.2022.854474>
99. Hayajneh, A. A., Al-Younis, M. O., & Rababa, M. (2024). The effect of a mindfulness intervention (MI) on sleep disturbance (SD) among nurses. *Scientific reports*, *14*(1), 5084. [https://doi.org/https://dx.doi.org/10.1038/s41598-024-55748-5](https://doi.org/https:/dx.doi.org/10.1038/s41598-024-55748-5)
100. Helvaci, G., Nur Aslan Cin, N., Canbulat, S., & Yardimci, H. (2020). Evaluating Diet and Sleep Quality of Shift and Non-Shift Nurses Using Three-Factor Pittsburgh Sleep Quality Index and Healthy Eating Index-2015. Sleep Medicine Research, 11(2), 94-101. [https://doi.org/https://dx.doi.org/10.17241/SMR.2020.00682](https://doi.org/https:/dx.doi.org/10.17241/SMR.2020.00682)
101. Hemmati-Maslakpak, M., Mollazadeh, F., & Jamshidi, H. (2021). The predictive power of sleep quality by morning-evening chronotypes, job satisfaction, and shift schedule in nurses: A cross-sectional study. Iranian Journal of Nursing and Midwifery Research, 26(2), 127-132. [https://doi.org/https://dx.doi.org/10.4103/ijnmr.IJNMR-301-19](https://doi.org/https:/dx.doi.org/10.4103/ijnmr.IJNMR-301-19)
102. Hesselbacher, S., Guntupalli, B., Surani, S., & Subramanian, S. (2015). Sleep Quality and Vigilance Differ Among Inpatient Nurses Based on the Unit Setting and Shift Worked. Journal of patient safety, 11(4), 215-220. [https://doi.org/https://dx.doi.org/10.1097/PTS.0000000000000089](https://doi.org/https:/dx.doi.org/10.1097/PTS.0000000000000089)
103. Hittle, B. M., Wardlaw, C., Lambert, J., & Bankston, K. (2023). A Cross-Sectional Study of the Social Work Environment and Black Registered Nurses' Sleep. Journal of racial and ethnic health disparities. [https://doi.org/https://dx.doi.org/10.1007/s40615-023-01717-z](https://doi.org/https:/dx.doi.org/10.1007/s40615-023-01717-z)
104. Holanda, N. C. P., de Castro Moreno, C. R., & Marqueze, E. C. (2018). Metabolic syndrome components: Is there a difference according to exposure to night work? Chronobiology international, 35(6), 801-810. [https://doi.org/https://dx.doi.org/10.1080/07420528.2018.1450267](https://doi.org/https:/dx.doi.org/10.1080/07420528.2018.1450267)
105. Hong, K. J., & Lee, Y. (2020). The moderating effect of nursing practice environment on the relationship between clinical nurses' sleep quality and wellness. International journal of environmental research and public health, 17(19), 1-11. [https://doi.org/https://dx.doi.org/10.3390/ijerph17197068](https://doi.org/https:/dx.doi.org/10.3390/ijerph17197068)
106. Hsieh, H.-F., Liu, Y., Hsu, H.-T., Ma, S.-C., Wang, H.-H., & Ko, C.-H. (2021). Relations between Stress and Depressive Symptoms in Psychiatric Nurses: The Mediating Effects of Sleep Quality and Occupational Burnout. International journal of environmental research and public health, 18(14). [https://doi.org/https://dx.doi.org/10.3390/ijerph18147327](https://doi.org/https:/dx.doi.org/10.3390/ijerph18147327)
107. Hsieh, M.-L., Li, Y.-M., Chang, E.-T., Lai, H.-L., Wang, W.-H., & Wang, S.-C. (2011). Sleep disorder in Taiwanese nurses: A random sample survey. Nursing & Health Sciences, 13(4), 468-474. [https://doi.org/https://dx.doi.org/10.1111/j.1442-2018.2011.00641.x](https://doi.org/https:/dx.doi.org/10.1111/j.1442-2018.2011.00641.x)
108. Hsu, H.-C., Lee, H.-F., & Lin, M.-H. (2021). Exploring the Association between Sleep Quality and Heart Rate Variability among Female Nurses. International journal of environmental research and public health, 18(11). [https://doi.org/https://dx.doi.org/10.3390/ijerph18115551](https://doi.org/https:/dx.doi.org/10.3390/ijerph18115551)
109. Huang, L., Lei, W., Liu, H., Hang, R., Tao, X., & Zhan, Y. (2022). Nurses' Sleep Quality of "Fangcang" Hospital in China during the COVID-19 Pandemic. International Journal of Mental Health and Addiction, 20(2), 789-799. [https://doi.org/https://dx.doi.org/10.1007/s11469-020-00404-y](https://doi.org/https:/dx.doi.org/10.1007/s11469-020-00404-y)
110. Huang, T., Poole, E. M., Vetter, C., Rexrode, K. M., Kubzansky, L. D., Schernhammer, E., Rohleder, N., Hu, F. B., Redline, S., & Tworoger, S. S. (2017). Habitual sleep quality and diurnal rhythms of salivary cortisol and dehydroepiandrosterone in postmenopausal women. *Psychoneuroendocrinology*, *84*(1), 172-180. [https://doi.org/https://dx.doi.org/10.1016/j.psyneuen.2017.07.484](https://doi.org/https:/dx.doi.org/10.1016/j.psyneuen.2017.07.484)
111. Hui-Ren, Z., Li-Li, M., Qin, L., Wei-Ying, Z., Hai-Ping, Y., & Wei, Z. (2023). Evaluation of the correlation between sleep quality and work engagement among nurses in Shanghai during the post-epidemic era. *Nursing open*, *10*(7), 4838-4848. [https://doi.org/https://dx.doi.org/10.1002/nop2.1735](https://doi.org/https:/dx.doi.org/10.1002/nop2.1735)
112. Huth, J. J., Eliades, A., Handwork, C., Englehart, J. L., & Messenger, J. (2013). Shift worked, quality of sleep, and elevated body mass index in pediatric nurses. *Journal of Pediatric Nursing*, *28*(6), 64-73. [https://doi.org/https://dx.doi.org/10.1016/j.pedn.2013.02.032](https://doi.org/https:/dx.doi.org/10.1016/j.pedn.2013.02.032)
113. Hwang, S., & Lee, J. (2023). The influence of COVID-19-related resilience on depression, job stress, sleep quality, and burnout among intensive care unit nurses. *Frontiers in Psychology*, *14*(1).<https://doi.org/10.3389/fpsyg.2023.1168243>
114. Hwang, S.-K., Lee, Y.-J., Cho, M.-E., Kim, B.-K., & Yoon, Y.-I. (2022). Factors Associated with Gastrointestinal Symptoms among Rotating Shift Nurses in South Korea: A Cross-Sectional Study. *International journal of environmental research and public health*, *19*(16), 9795. https://doi.org/https://dx.doi.org/10.3390/ijerph19169795
115. Ichiba, T., Gorlova, S., Nagashima, Y., Aitake, M., Hori, E., & Nishijo, H. (2018). Association Between Sleep Quality and Subjective Fatigue in Night-Shift Nurses with Good and Poor Sleep. *Sleep and Vigilance*, *2*(1), 63-69. [https://doi.org/https://dx.doi.org/10.1007/s41782-018-0036-y](https://doi.org/https:/dx.doi.org/10.1007/s41782-018-0036-y)
116. James, L., James, S. M., Wilson, M., Brown, N., Dotson, E. J., Dan Edwards, C., & Butterfield, P. (2020). Sleep health and predicted cognitive effectiveness of nurses working 12-hour shifts: An observational study [Professional Personnel Attitudes & Characteristics 3430]. *International Journal of Nursing Studies*, *112*(1), 103667. [https://doi.org/https://dx.doi.org/10.1016/j.ijnurstu.2020.103667](https://doi.org/https:/dx.doi.org/10.1016/j.ijnurstu.2020.103667)
117. JÄRvelin-Pasanen, S., Ropponen, A., Tarvainen, M. P., Karjalainen, P. A., & Louhevaara, V. (2013). Differences in Heart Rate Variability of Female Nurses between and within Normal and Extended Work Shifts. *Industrial health*, *51*(2), 154-164.<https://doi.org/10.2486/indhealth.MS1368>
118. Johnson, A. L., Jung, L., Brown, K. C., Weaver, M. T., & Richards, K. C. (2014). Sleep deprivation and error in nurses who work the night shift. *Journal of Nursing Administration*, *44*(1), 17-22. [https://doi.org/https://dx.doi.org/10.1097/NNA.0000000000000016](https://doi.org/https:/dx.doi.org/10.1097/NNA.0000000000000016)
119. Juan-Garcia, C., Plaza-Carmona, M., & Fernandez-Martinez, N. (2021). Sleep analysis in emergency nurses' department. *Revista da Associacao Medica Brasileira*, *67*(6), 862-867. [https://doi.org/https://dx.doi.org/10.1590/1806-9282.20210183](https://doi.org/https:/dx.doi.org/10.1590/1806-9282.20210183)
120. Jung, G., & Oh, J. (2020). The relationship between childhood trauma, eating disorders, and sleep quality among registered hospital nurses in south korea. *Healthcare (Switzerland)*, *8*(4).<https://doi.org/10.3390/healthcare8040490>
121. Kagamiyama, H., Sumi, N., Yoshida, Y., Sugimura, N., Nemoto, F., & Yano, R. (2019). Association between sleep and fatigue in nurses who are engaged in 16 h night shifts in Japan: Assessment using actigraphy. *Japan journal of nursing science: JJNS*, *16*(4), 373-384.<https://doi.org/10.1111/jjns.12246>
122. Kang, H., Lee, M., & Jang, S. J. (2020). The Impact of Social Jetlag on Sleep Quality among Nurses: A Cross-Sectional Survey. *International journal of environmental research and public health*, *18*(1). [https://doi.org/https://dx.doi.org/10.3390/ijerph18010047](https://doi.org/https:/dx.doi.org/10.3390/ijerph18010047)
123. Kang, J.-H., Miao, N.-F., Tseng, I.-J., Sithole, T., & Chung, M.-H. (2015). Circadian Activity Rhythms and Sleep in Nurses Working Fixed 8-hr Shifts. *Biological Research for Nursing*, *17*(3), 348-355. [https://doi.org/https://dx.doi.org/10.1177/1099800414549454](https://doi.org/https:/dx.doi.org/10.1177/1099800414549454)
124. Karagozoglu, S., & Bingol, N. (2008). Sleep quality and job satisfaction of Turkish nurses. *Nursing Outlook*, *56*(6), 298-307. [https://doi.org/https://dx.doi.org/10.1016/j.outlook.2008.03.009](https://doi.org/https:/dx.doi.org/10.1016/j.outlook.2008.03.009)
125. Karahan, A., Abbasoglu, A., Ugurlu, Z., Isik, S. A., Kilic, G., & Elbas, N. O. (2020). Determination of sleep quality, fatigue, and concentration in nurses according to their shifts and chronotype. *Journal of Psychiatric Nursing*, *11*(2), 98-105. [https://doi.org/https://dx.doi.org/10.14744/phd.2019.90277](https://doi.org/https:/dx.doi.org/10.14744/phd.2019.90277)
126. Kato, C., Shimada, J., & Hayashi, K. (2012). Sleepiness during shift work in Japanese nurses: A comparison study using JESS, SSS, and actigraphy. *Sleep and Biological Rhythms*, *10*(2), 109-117. [https://doi.org/https://dx.doi.org/10.1111/j.1479-8425.2011.00528.x](https://doi.org/https:/dx.doi.org/10.1111/j.1479-8425.2011.00528.x)
127. Katsifaraki, M., Nilsen, K. B., Christensen, J. O., Waersted, M., Knardahl, S., Bjorvatn, B., Harma, M., & Matre, D. (2019). Sleep duration mediates abdominal and lower-extremity pain after night work in nurses. *International archives of occupational and environmental health*, *92*(3), 415-422. https://doi.org/https://dx.doi.org/10.1007/s00420-018-1373-9
128. Katsifaraki, M., Nilsen, K. B., Christensen, J. O., Waersted, M., Knardahl, S., Bjorvatn, B., Harma, M., & Matre, D. (2020). Pain complaints after consecutive nights and quick returns in Norwegian nurses working three-shift rotation: An observational study. *BMJ Open*, *10*(9), 35533. [https://doi.org/https://dx.doi.org/10.1136/bmjopen-2019-035533](https://doi.org/https:/dx.doi.org/10.1136/bmjopen-2019-035533)
129. Kayaroganam, R., Sarkar, S., Satheesh, S., Tamilmani, S., Sivanantham, P., & Kar, S. S. (2022). Prevalence and correlates of psychological distress among nurses in a teaching institute in South India. *Journal of family medicine and primary care*, *11*(11), 6765-6771. [https://doi.org/https://dx.doi.org/10.4103/jfmpc.jfmpc_2313_21](https://doi.org/https:/dx.doi.org/10.4103/jfmpc.jfmpc_2313_21)
130. Khade, Y., Behera, S., Parvathi, R., & Korradi, S. (2018). Study of insomnia, daytime sleepiness and sleep quality among South Indian nurses. *Journal of Clinical and Diagnostic Research*, *12*(4), 9-12. [https://doi.org/https://dx.doi.org/10.7860/JCDR/2018/32602.11392](https://doi.org/https:/dx.doi.org/10.7860/JCDR/2018/32602.11392)
131. Khastar, H., Mirrezaie, S. M., Chashmi, N. A., & Jahanfar, S. (2020). Sleep Improvement Effect on Sexual Life Quality Among Rotating Female Shift Workers: A Randomized Controlled Trial. *Journal of Sexual Medicine*, *17*(8), 1467-1475. [https://doi.org/https://dx.doi.org/10.1016/j.jsxm.2020.03.003](https://doi.org/https:/dx.doi.org/10.1016/j.jsxm.2020.03.003)
132. Khatony, A., Zakiei, A., Khazaie, H., Rezaei, M., & Janatolmakan, M. (2020). International Nursing: A Study of Sleep Quality Among Nurses and Its Correlation With Cognitive Factors. *Nursing administration quarterly*, *44*(1), 1-10. [https://doi.org/https://dx.doi.org/10.1097/NAQ.0000000000000397](https://doi.org/https:/dx.doi.org/10.1097/NAQ.0000000000000397)
133. Kikuchi, Y., Ishii, N., & Kodama, H. (2018). Effects of night-time on-call work on heart rate variability before bed and sleep quality in visiting nurses. *International archives of occupational and environmental health*, *91*(6), 695-704. [https://doi.org/https://dx.doi.org/10.1007/s00420-018-1317-4](https://doi.org/https:/dx.doi.org/10.1007/s00420-018-1317-4)
134. Kim, H. I., Jung, S.-A., Choi, J. Y., Kim, S.-E., Jung, H.-K., Shim, K.-N., & Yoo, K. (2013). Impact of shiftwork on irritable bowel syndrome and functional dyspepsia. *Journal of Korean medical science*, *28*(3), 431-437. [https://doi.org/https://dx.doi.org/10.3346/jkms.2013.28.3.431](https://doi.org/https:/dx.doi.org/10.3346/jkms.2013.28.3.431)
135. Kim, K., & Lee, Y. (2022). Influence of Sleep Characteristic Changes on Nurses' Quality of Life during Their Transition to Practice: A Prospective Cohort Study. *International journal of environmental research and public health*, *19*(1), 573. [https://doi.org/https://dx.doi.org/10.3390/ijerph19010573](https://doi.org/https:/dx.doi.org/10.3390/ijerph19010573)
136. Kim, T., & Jang, S. J. (2021). Shift nurses' social jetlag, anxiety, and premenstrual symptoms: A cross-sectional study. *Collegian, 29*(4), 477-483. [https://doi.org/https://dx.doi.org/10.1016/j.colegn.2021.11.001](https://doi.org/https:/dx.doi.org/10.1016/j.colegn.2021.11.001)
137. Kim-Godwin, Y., Lee, M., Logan, J. G., & Liu, X. (2021). Factors influencing sleep quality among female staff nurses during the early covid-19 pandemic in the United States. *International journal of environmental research and public health*, *18*(9), 4827. [https://doi.org/https://dx.doi.org/10.3390/ijerph18094827](https://doi.org/https:/dx.doi.org/10.3390/ijerph18094827)
138. Kishore, K., & Shekhar, S. (2023). A Cross Sectional Observational Assessment of Sleep Quality among Residents, Interns, Staff Nurses and Medical Students. *International Journal of Current Pharmaceutical Review and Research*, *15*(5), 198-204.
139. Koh, S.-J., Kim, M., Oh, D. Y., Kim, B. G., Lee, K. L., & Kim, J. W. (2014). Psychosocial stress in nurses with shift work schedule is associated with functional gastrointestinal disorders. *Journal of neurogastroenterology and motility*, *20*(4), 516-522. [https://doi.org/https://dx.doi.org/10.5056/jnm14034](https://doi.org/https:/dx.doi.org/10.5056/jnm14034)
140. Korkmaz, S., Kazgan, A., Cekic, S., Tartar, A. S., Balci, H. N., & Atmaca, M. (2020). The anxiety levels, quality of sleep and life and problem-solving skills in healthcare workers employed in COVID-19 services. *Journal of Clinical Neuroscience*, *80*(1), 131-136. [https://doi.org/https://dx.doi.org/10.1016/j.jocn.2020.07.073](https://doi.org/https:/dx.doi.org/10.1016/j.jocn.2020.07.073)
141. Kumar, S., Azmi, S. A., Gaur, R. K., & Akhouri, D. (2022). Observational Study to Assess the Sleep Quality Among Residents, Interns, Staff Nurses and Medical Students Using Pittsburgh Sleep Quality Index Scale Questionnaire (PQSI Score). *International Journal of Toxicological and Pharmacological Research*, *12*(1), 77-83.
142. Kunert, K., King, M. L., & Kolkhorst, F. W. (2007). Fatigue and sleep quality in nurses: Tiredness can negatively affect hospital nurses' quality of life and patient care. *Journal of Psychosocial Nursing and Mental Health Services*, *45*(8), 31-37.
143. Kurebayashi, Y. (2020). Effects of self-compassion and self-focus on sleep disturbances among psychiatric nurses. *Perspectives in psychiatric care*, *56*(2), 474-480. [https://doi.org/https://dx.doi.org/10.1111/ppc.12458](https://doi.org/https:/dx.doi.org/10.1111/ppc.12458)
144. Kwak, S.-K., Ahn, J.-S., & Kim, Y.-H. (2024). The Association of Job Stress, Quality of Sleep, and the Experience of Near-Miss Errors among Nurses in General Hospitals. *Healthcare (Basel, Switzerland)*, *12*(6). [https://doi.org/https://dx.doi.org/10.3390/healthcare12060699](https://doi.org/https:/dx.doi.org/10.3390/healthcare12060699)
145. Labyak, S., Lava, S., Turek, F., & Zee, P. (2002). Effects of shiftwork on sleep and menstrual function in nurses. *Health Care for Women International*, *23*(7), 703-714. http://doi.org/ 10.1080/07399330290107449
146. Lee, C.-Y., Chen, H.-C., Meg Tseng, M.-C., Lee, H.-C., & Huang, L.-H. (2015). The Relationships Among Sleep Quality and Chronotype, Emotional Disturbance, and Insomnia Vulnerability in Shift Nurses. *The journal of nursing research: JNR*, *23*(3), 225-235. [https://doi.org/https://dx.doi.org/10.1097/jnr.0000000000000095](https://doi.org/https:/dx.doi.org/10.1097/jnr.0000000000000095)
147. Lee, H., & Jang, S. J. (2022). Social jetlag and depression in female rotating-shift nurses: A secondary analysis. *Perspectives in psychiatric care*, *58*(4), 2246-2254. [https://doi.org/https://dx.doi.org/10.1111/ppc.13054](https://doi.org/https:/dx.doi.org/10.1111/ppc.13054)
148. Lee, K. A. (1992). Self-reported sleep disturbances in employed women. *Sleep*, *15*(6), 493-498. [https://doi.org/http://dx.doi.org/10.1093/sleep/15.6.493](https://doi.org/http:/dx.doi.org/10.1093/sleep/15.6.493)
149. Lee, S., Mu, C., Gonzalez, B. D., Vinci, C. E., & Small, B. J. (2021). Sleep health is associated with next-day mindful attention in healthcare workers. *Sleep health*, *7*(1), 105-112. https://doi.org/https://dx.doi.org/10.1016/j.sleh.2020.07.005
150. Li, J. N., Chen, X. Q., Jiang, X. M., Zheng, Q. X., Pan, Y. Q., Zhu, Y., Huang, L., & Liu, R. L. (2023). Exploring the Associations between Chronotype, Night Shift Work Schedule, Quality of Work Life, and Sleep Quality among Maternal and Child Health Nurses: A Multicentre Cross-Sectional Study. *Journal of Nursing Management*, *2023*, 1811732. [https://doi.org/https://dx.doi.org/10.1155/2023/1811732](https://doi.org/https:/dx.doi.org/10.1155/2023/1811732)
151. Li, N., Zhu, P., Li, H., Chen, X., Chen, L., & Chen, M. (2022). Association Between Role Overload and Sleep Quality Among Chinese First-Line Nurses During the COVID-19 Outbreak: The Moderating Effect of Mindfulness. *The journal of nursing research : JNR*, *30*(6), e242. [https://doi.org/https://dx.doi.org/10.1097/jnr.0000000000000531](https://doi.org/https:/dx.doi.org/10.1097/jnr.0000000000000531)
152. Lin, L. C., Huang, Y. C., Carter, P., & Zuniga, J. (2021). Resilience among nurses in long term care and rehabilitation settings. *Applied Nursing Research*, *62(1)*, 151518. [https://doi.org/https://dx.doi.org/10.1016/j.apnr.2021.151518](https://doi.org/https:/dx.doi.org/10.1016/j.apnr.2021.151518)
153. Lin, P.-C., Chen, C.-H., Pan, S.-M., Pan, C.-H., Chen, C.-J., Chen, Y.-M., Hung, H.-C., & Wu, M.-T. (2012). Atypical work schedules are associated with poor sleep quality and mental health in Taiwan female nurses. *International archives of occupational and environmental health*, *85*(8), 877-884. [https://doi.org/https://dx.doi.org/10.1007/s00420-011-0730-8](https://doi.org/https:/dx.doi.org/10.1007/s00420-011-0730-8)
154. Lin, R. T., Lin, Y. T., Hsia, Y. F., & Kuo, C. C. (2021). Long working hours and burnout in health care workers: Non‐linear dose‐response relationship and the effect mediated by sleeping hours—A cross‐sectional study. *Journal of Occupational Health*, *63*(1), 12228.<https://doi.org/10.1002/1348-9585.12228>
155. Lin, S.-H., Liao, W.-C., Chen, M.-Y., & Fan, J.-Y. (2014). The impact of shift work on nurses' job stress, sleep quality and self-perceived health status. *Journal of Nursing Management*, *22*(5), 604-612. [https://doi.org/https://dx.doi.org/10.1111/jonm.12020](https://doi.org/https:/dx.doi.org/10.1111/jonm.12020)
156. Lin, Y.-E., Lin, C.-T., Hu, M.-L., Tzeng, S., & Chien, L.-Y. (2023). The relationships among perceived stress, resilience, sleep quality and first-month retention of newly employed nurses: A cross-sectional survey. *Nursing open*, *10*(6), 4004-4012. [https://doi.org/https://dx.doi.org/10.1002/nop2.1659](https://doi.org/https:/dx.doi.org/10.1002/nop2.1659)
157. Liu, D., Wu, Y., Jiang, F., Liu, Y., & Tang, Y.-L. (2021). The Prevalence and Associated Factors of Short Sleep Duration Among Nurses in Tertiary Public Hospitals in China: Findings from a National Survey. *Risk management and healthcare policy*, *14*(1), 2547-2552. [https://doi.org/https://dx.doi.org/10.2147/RMHP.S315262](https://doi.org/https:/dx.doi.org/10.2147/RMHP.S315262)
158. Liu, K., Yin, T., & Shen, Q. (2020). Relationships between sleep quality, mindfulness and work-family conflict in Chinese nurses: A cross-sectional study. *Applied nursing research: ANR*, *55*(1), 151250. [https://doi.org/https://dx.doi.org/10.1016/j.apnr.2020.151250](https://doi.org/https:/dx.doi.org/10.1016/j.apnr.2020.151250)
159. Liu, X., Xu, Y., Xu, H., Jiang, L., Wang, T., Chen, C., Lee, A., & Zhu, P. (2022). Anxiety and sleep quality among front-line nurses treating first wave COVID-19 in China: The mediating role of mindfulness. *Archives of psychiatric nursing*, *41*(1), 341-347. [https://doi.org/https://dx.doi.org/10.1016/j.apnu.2022.09.007](https://doi.org/https:/dx.doi.org/10.1016/j.apnu.2022.09.007)
160. Liu, Y., Xian, J. S., Wang, R., Ma, K., Li, F., Wang, F. L., Yang, X., Mu, N., Xu, K., Quan, Y. L., Wang, S., Lai, Y., Yang, C. Y., Li, T., Zhang, Y., Tan, B., Feng, H., Chen, T. N., & Wang, L. H. (2022). Factoring and correlation in sleep, fatigue, and mental workload of clinical first-line nurses in the post-pandemic era of COVID-19: A multi-center cross-sectional study. *Frontiers in Psychiatry*, *13*(1), 963419. [https://doi.org/https://dx.doi.org/10.3389/fpsyt.2022.963419](https://doi.org/https:/dx.doi.org/10.3389/fpsyt.2022.963419)
161. Lyu, X., Li, K., Liu, Q., Wang, X., Yang, Z., Yang, Y., Yang, Q., Wang, H., Yuan, N., Ji, C., Kong, F., Li, W., Yin, M., Li, Z., Zou, S., Zhao, X., Fang, X., Zhang, C., & Du, X. (2022). Sleep status of psychiatric nurses: A survey from China. *Nursing open*, *9*(6), 2720-2728. [https://doi.org/https://dx.doi.org/10.1002/nop2.972](https://doi.org/https:/dx.doi.org/10.1002/nop2.972)
162. Magnavita, N., Mele, L., Meraglia, I., Merella, M., Vacca, M. E., Cerrina, A., Gabriele, M., Labella, M., Soro, M. T., Ursino, S., & Matera, C. (2022). The Impact of Workplace Violence on Headache and Sleep Problems in Nurses. *International journal of environmental research and public health*, *19*(20), 13423. [https://doi.org/https://dx.doi.org/10.3390/ijerph192013423](https://doi.org/https:/dx.doi.org/10.3390/ijerph192013423)
163. Majumdar, P., Adan, A., & Sahu, S. (2023). Implication of Sleep and Food Intake Pattern on the Depressive Status of the Female Shift Workers Engaged in Two-Shift System. *Sleep and Vigilance*, *7*(2), 195-205. [https://doi.org/https://dx.doi.org/10.1007/s41782-023-00246-3](https://doi.org/https:/dx.doi.org/10.1007/s41782-023-00246-3)
164. Makowiec-Dąbrowska, T., Krawczyk-Adamus, P., Sprusińska, E., & Jóźwiak, Z. W. (2000). Can Nurses Be Employed in 12-Hour Shift Systems? *International journal of occupational safety and ergonomics*, *6*(3), 393-403.<https://doi.org/10.1080/10803548.2000.11076463>
165. Malak, M. Z., Al-Amer, R. M., & Abu Adas, M. H. (2023). The influence of shift-work on perceived stress, sleep quality, and body mass index among emergency nurses. *Journal of Human Behavior in the Social Environment*, *33*(6), 779-792. [https://doi.org/https://dx.doi.org/10.1080/10911359.2023.2177226](https://doi.org/https:/dx.doi.org/10.1080/10911359.2023.2177226)
166. Matre, D., Knardahl, S., & Nilsen, K. B. (2017). Night-shift work is associated with increased pain perception [Article]. *Scandinavian Journal of Work, Environment and Health*, *43*(3), 260-268.<https://doi.org/10.5271/sjweh.3627>
167. McDowall, K., Murphy, E., & Anderson, K. (2017). The impact of shift work on sleep quality among nurses. *Occupational medicine (Oxford, England)*, *67*(8), 621-625. [https://doi.org/https://dx.doi.org/10.1093/occmed/kqx152](https://doi.org/https:/dx.doi.org/10.1093/occmed/kqx152)
168. Miguez-Torres, N., Martínez-Rodríguez, A., Martínez-Olcina, M., Miralles-Amorós, L., & Reche-García, C. (2021). Relationship between emotional intelligence, sleep quality and body mass index in emergency nurses. *Healthcare (Switzerland)*, *9*(5), 607.<https://doi.org/10.3390/healthcare9050607>
169. Min, A., Hong, H. C., Son, S., & Lee, T. (2021). Sleep, fatigue and alertness during working hours among rotating-shift nurses in Korea: An observational study. *Journal of Nursing Management*, *29*(8), 2647-2657. [https://doi.org/https://dx.doi.org/10.1111/jonm.13446](https://doi.org/https:/dx.doi.org/10.1111/jonm.13446)
170. Minelli, A., Di Palma, M., Rocchi, M. B. L., Ponzio, E., Barbadoro, P., Bracci, M., Pelusi, G., & Prospero, E. (2021). Cortisol, chronotype, and coping styles as determinants of tolerance of nursing staff to rotating shift work. *Chronobiology international*, *38*(5), 666-680. [https://doi.org/https://dx.doi.org/10.1080/07420528.2021.1887883](https://doi.org/https:/dx.doi.org/10.1080/07420528.2021.1887883)
171. Mohamed, B. E. S., Ghaith, R. F. A. H., & Ahmed, H. A. A. E. (2022). Relationship between work–family conflict, sleep quality, and depressive symptoms among mental health nurses. *Middle East Current Psychiatry*, *29*(1).<https://doi.org/10.1186/s43045-022-00183-3>
172. Mokros, L., Koprowicz, J., Nowakowska-Domagala, K., Rodak, J., & Pietras, T. (2018). Eveningness and its possibility of predicting burnout symptoms among physicians and nurses - Preliminary results. *Current Problems of Psychiatry*, *19*(1), 23-29. [https://doi.org/https://dx.doi.org/10.1515/cpp-2018-0003](https://doi.org/https:/dx.doi.org/10.1515/cpp-2018-0003)
173. Molu, B., Tuna, P. T., Keskin, A. Y., & Tuna, H. I. (2023). Determination of Chronotype, Quality of Life, Sleep Quality, and Social Jetlag in Shift Working Nurses. *Journal of Turkish Sleep Medicine*, *10*(1), 48-53. [https://doi.org/https://dx.doi.org/10.4274/jtsm.galenos.2022.29484](https://doi.org/https:/dx.doi.org/10.4274/jtsm.galenos.2022.29484)
174. Molzof, H. E., Prapanjaroensin, A., Patel, V. H., Mokashi, M. V., Gamble, K. L., & Patrician, P. A. (2019). Misaligned core body temperature rhythms impact cognitive performance of hospital shift work nurses. *Neurobiology of Learning and Memory*, *160*(1), 151-159. [https://doi.org/https://dx.doi.org/10.1016/j.nlm.2019.01.002](https://doi.org/https:/dx.doi.org/10.1016/j.nlm.2019.01.002)
175. Morimoto, H., Tanaka, H., Ohkubo, R., Mimura, M., Ooe, N., Ichikawa, A., & Yukitoshi, H. (2016). Self-help therapy for sleep problems in hospital nurses in Japan: A controlled pilot study. *Sleep and Biological Rhythms*, *14*(2), 177-185. [https://doi.org/https://dx.doi.org/10.1007/s41105-015-0037-3](https://doi.org/https:/dx.doi.org/10.1007/s41105-015-0037-3)
176. Mousavi, S., Mohammadi, V., & Foroughi, Z. (2020). Effect of coenzyme Q10 supplementation on work-related fatigue in nurses: a double-blind, randomized placebo-controlled study. *Fatigue: Biomedicine, Health and Behavior*, *8*(1), 1-10. [https://doi.org/https://dx.doi.org/10.1080/21641846.2019.1704374](https://doi.org/https:/dx.doi.org/10.1080/21641846.2019.1704374)
177. Mulhall, M. D., Sletten, T. L., Magee, M., Stone, J. E., Ganesan, S., Collins, A., Anderson, C., Lockley, S. W., Howard, M. E., & Rajaratnam, S. M. W. (2019). Sleepiness and driving events in shift workers: The impact of circadian and homeostatic factors. *Sleep*, *42*(6), zsz074. [https://doi.org/https://dx.doi.org/10.1093/sleep/zsz074](https://doi.org/https:/dx.doi.org/10.1093/sleep/zsz074)
178. Nagurney, J. T. (2005). The Accuracy and Completeness of Data Collected by Prospective and Retrospective Methods. *Academic Emergency Medicine*, *12*(9), 884–895. <https://doi.org/10.1197/j.aem.2005.04.021>
179. Nascimento, J. O. V., dos Santos, J., Meira, K. C., Pierin, A. M. G., & Souza-Talarico, J. N. (2019). Shift work of nursing professionals and blood pressure, burnout, and common mental disorders. *Revista da Escola de Enfermagem*, *53*(1), 03443. [https://doi.org/https://dx.doi.org/10.1590/S1980-220X2018002103443](https://doi.org/https:/dx.doi.org/10.1590/S1980-220X2018002103443)
180. Nishimura, Y., Ikeda, H., Matsumoto, S., Izawa, S., Kawakami, S., Tamaki, M., Masuda, S., & Kubo, T. (2023). Impaired self-monitoring ability on reaction times of psychomotor vigilance task of nurses after a night shift. *Chronobiology international*, *40*(5), 603-611. [https://doi.org/https://dx.doi.org/10.1080/07420528.2023.2193270](https://doi.org/https:/dx.doi.org/10.1080/07420528.2023.2193270)
181. Niu, S.-F., Miao, N.-F., Liao, Y.-M., Chi, M.-J., Chung, M.-H., & Chou, K.-R. (2017). Sleep Quality Associated with Different Work Schedules: A Longitudinal Study of Nursing Staff. *Biological Research for Nursing*, *19*(4), 375-381. [https://doi.org/https://dx.doi.org/10.1177/1099800417695483](https://doi.org/https:/dx.doi.org/10.1177/1099800417695483)
182. Norful, A. A., Haghighi, F., & Shechter, A. (2023). Assessing sleep health dimensions in frontline registered nurses during the COVID-19 pandemic: implications for psychological health and wellbeing. *SLEEP Advances*, *4*(1).<https://doi.org/10.1093/sleepadvances/zpac046>
183. Nourian, M., Nikfarid, L., Khavari, A. M., Barati, M., & Allahgholipour, A. R. (2021). The Impact of an Online Mindfulness-Based Stress Reduction Program on Sleep Quality of Nurses Working in COVID-19 Care Units: A Clinical Trial. *Holistic nursing practice*, *35*(5), 257-263. [https://doi.org/https://dx.doi.org/10.1097/HNP.0000000000000466](https://doi.org/https:/dx.doi.org/10.1097/HNP.0000000000000466)
184. Olson, J. A., Artenie, D. Z., Cyr, M., Raz, A., & Lee, V. (2020). Developing a light-based intervention to reduce fatigue and improve sleep in rapidly rotating shift workers [Physiological Processes 2540]. *Chronobiology international*, *37*(4), 573-591. [https://doi.org/https://dx.doi.org/10.1080/07420528.2019.1698591](https://doi.org/https:/dx.doi.org/10.1080/07420528.2019.1698591)
185. Panwar, A., Bagla, R. K., Mohan, M., & Rathore, B. B. (2024). The impact of shift work on sleep quality and circadian patterns of heart rate variability among nurses working in a tertiary care hospital. *Journal of Cardiovascular Disease Research*, *15*(2), 900-905. [https://doi.org/https://dx.doi.org/10.4807/jcdr.2024.15.02.102](https://doi.org/https:/dx.doi.org/10.4807/jcdr.2024.15.02.102)
186. Parajuli, N., Pradhan, B., & Jat, M. (2021). Effect of four weeks of integrated yoga intervention on perceived stress and sleep quality among female nursing professionals working at a tertiary care hospital: A pilot study. *Industrial psychiatry journal*, *30*(1), 136-140.<https://doi.org/10.4103/ipj.ipj_11_21>
187. Park, C. H., Bang, M., Ahn, K. J., Kim, W. J., & Shin, N. Y. (2020). Sleep disturbance-related depressive symptom and brain volume reduction in shift-working nurses. *Scientific reports*, *10*(1).<https://doi.org/10.1038/s41598-020-66066-x>
188. Park, E., Lee, H. Y., & Park, C. S.-Y. (2018). Association between sleep quality and nurse productivity among Korean clinical nurses. *Journal of Nursing Management*, *26*(8), 1051-1058. [https://doi.org/https://dx.doi.org/10.1111/jonm.12634](https://doi.org/https:/dx.doi.org/10.1111/jonm.12634)
189. Pelin, M., Sert, H., Horzum, M. B., & Randler, C. (2023). Relationship between burnout levels of nurses and their circadian preference, sleep quality, and personality traits. *Chronobiology international*, *40*(7), 885-895. [https://doi.org/https://dx.doi.org/10.1080/07420528.2023.2222814](https://doi.org/https:/dx.doi.org/10.1080/07420528.2023.2222814)
190. Peplonska, B., Bukowska, A., Gromadzinska, J., Sobala, W., Reszka, E., Lie, J.-A., Kjuus, H., & Wasowicz, W. (2012). Night shift work characteristics and 6-sulfatoxymelatonin (MT6s) in rotating night shift nurses and midwives. *Occupational and environmental medicine (London, England)*, *69*(5), 339-346.<https://doi.org/10.1136/oemed-2011-100273>
191. Petrosino, F., Bartoli, D., Trotta, F., Di Nome, S., Di Sarli, M. G., Frammartino, R., & Giancane, F. (2024). Nurses quality of life, sleep disturbance, and intention to leave critical care units: A cross-sectional moderated mediation analysis. *Intensive & critical care nursing*, *81*(1), 103602. [https://doi.org/https://dx.doi.org/10.1016/j.iccn.2023.103602](https://doi.org/https:/dx.doi.org/10.1016/j.iccn.2023.103602)
192. Polydoropoulou, E., Intas, G., Platis, C., Stergiannis, P., & Panoutsopoulos, G. (2023). Investigating Physical Activity Habits and Sleep Disorders in the Nursing Staff of Greece During the COVID-19 Pandemic: A Multicenter Cross-Sectional Correlational Study. *Advances in Experimental Medicine and Biology*, *1425*, 535-543. [https://doi.org/https://dx.doi.org/10.1007/978-3-031-31986-0_52](https://doi.org/https:/dx.doi.org/10.1007/978-3-031-31986-0_52)
193. Qiao, H., Beibei, Z., Chong, T., Tieying, Z., Yuzhi, G., Jing, M., & Davidson, P. M. (2020). Both frequency and duration of rotating night shifts are associated with metabolic parameters: a cross-sectional study. *Sleep Medicine*, *71*, 89-96. [https://doi.org/https://dx.doi.org/10.1016/j.sleep.2020.02.016](https://doi.org/https:/dx.doi.org/10.1016/j.sleep.2020.02.016)
194. Quera-Salva, M. A., Guilleminault, C., Claustrat, B., Defrance, R., Gajdos, P., McCann, C. C., & De Lattre, J. (1997). Rapid shift in peak melatonin secretion associated with improved performance in short shift work schedule. *Sleep*, *20*(12), 1145-1150.<http://ovidsp.ovid.com/ovidweb.cgi?T=JS&PAGE=reference&D=emed6&NEWS=N&AN=28082307>
195. Rahman, H. A., Hatsanee, A., Menjeni, N. A., Salleh, Z. A., Hamid, R. A., & Ali, M. (2022). Perceived sleep quality: a comparison between hospital nurses and student nurses. *British journal of nursing (Mark Allen Publishing)*, *31*(11), 578-588. [https://doi.org/https://dx.doi.org/10.12968/bjon.2022.31.11.578](https://doi.org/https:/dx.doi.org/10.12968/bjon.2022.31.11.578)
196. Rajcani, J., Vytykacova, S., Solarikova, P., & Brezina, I. (2021). Stress and hair cortisol concentrations in nurses during the first wave of the COVID-19 pandemic. *Psychoneuroendocrinology*, *129*, 105245. [https://doi.org/https://dx.doi.org/10.1016/j.psyneuen.2021.105245](https://doi.org/https:/dx.doi.org/10.1016/j.psyneuen.2021.105245)
197. Ramadan, M. Z., & Al-Saleh, K. S. (2014). The association of sleep deprivation on the occurrence of errors by nurses who work the night shift. *Current health sciences journal*, *40*(2), 97-103. [https://doi.org/https://dx.doi.org/10.12865/CHSJ.40.02.03](https://doi.org/https:/dx.doi.org/10.12865/CHSJ.40.02.03)
198. Roman, P., Perez-Cayuela, I., Gil-Hernandez, E., Rodriguez-Arrastia, M., Aparicio-Mota, A., Ropero-Padilla, C., & Rueda-Ruzafa, L. (2023). Influence of Shift Work on The Health of Nursing Professionals. *Journal of Personalized Medicine*, *13*(4), 627. [https://doi.org/https://dx.doi.org/10.3390/jpm13040627](https://doi.org/https:/dx.doi.org/10.3390/jpm13040627)
199. Roveda, E., Castelli, L., Galasso, L., Mule, A., Ce, E., Condemi, V., Banfi, G., Montaruli, A., & Esposito, F. (2021). Differences in Daytime Activity Levels and Daytime Sleep Between Night and Day Duty: An Observational Study in Italian Orthopedic Nurses. *Frontiers in Physiology*, *12*, 628231. [https://doi.org/https://dx.doi.org/10.3389/fphys.2021.628231](https://doi.org/https:/dx.doi.org/10.3389/fphys.2021.628231)
200. Ruggiero, J. S. (2003). Correlates of fatigue in critical care nurses. *Research in nursing & health*, *26*(6), 434-444. http://doi.org/ 10.1002/nur.10106
201. Ruggiero, J. S. (2005). Health, Work Variables, and Job Satisfaction Among Nurses. *The Journal of nursing administration*, *35*(5), 254-263.<https://doi.org/10.1097/00005110-200505000-00009>
202. Ruggiero, J. S., & Avi-Itzhak, T. (2016). Sleep Patterns of Emergency Department Nurses on Workdays and Days Off. *The journal of nursing research: JNR*, *24*(2), 173-180. [https://doi.org/https://dx.doi.org/10.1097/jnr.0000000000000121](https://doi.org/https:/dx.doi.org/10.1097/jnr.0000000000000121)
203. Saadat, S., Karbakhsh, M., Saremi, M., Alimohammadi, I., Ashayeri, H., Fayaz, M., Sadeghian, F., & Rostami, R. (2018). A prospective study of psychomotor performance of driving among two kinds of shift work in Iran. *Electronic physician*, *10*(2), 6417-6425.<https://doi.org/10.19082/6417>
204. Sajedi, S., Jafarjalal, E., & Haghani, S. (2022). Relationship between Psychological Capital and Sleep Quality in Intensive Care Unit Nurses. *Journal of Client-Centered Nursing Care*, *8*(2), 139-145.<https://doi.org/10.32598/JCCNC.8.2.418.1>
205. Saleh, A. M., Awadalla, N. J., El-masri, Y. M., & Sleem, W. F. (2014). Impacts of nurses' circadian rhythm sleep disorders, fatigue, and depression on medication administration errors. *Egyptian Journal of Chest Diseases and Tuberculosis*, *63*(1), 145-153. [https://doi.org/https://dx.doi.org/10.1016/j.ejcdt.2013.10.001](https://doi.org/https:/dx.doi.org/10.1016/j.ejcdt.2013.10.001)
206. Samaha, E., Lal, S., Samaha, N., & Wyndham, J. (2007). Psychological, lifestyle and coping contributors to chronic fatigue in shift-worker nurses. *Journal of Advanced Nursing*, *59*(3), 221-232. [https://doi.org/https://dx.doi.org/10.1111/j.1365-2648.2007.04338.x](https://doi.org/https:/dx.doi.org/10.1111/j.1365-2648.2007.04338.x)
207. Sampaio, F., Gaspar, S., Fonseca, C., Lopes, M. J., Paiva, T., & Guedes de Pinho, L. (2023). Sleep Quality between Nurses and the General Population during the COVID-19 Pandemic in Portugal: What Are the Differences? *International journal of environmental research and public health*, *20*(8), 5531. [https://doi.org/https://dx.doi.org/10.3390/ijerph20085531](https://doi.org/https:/dx.doi.org/10.3390/ijerph20085531)
208. Saracoglu, K. T., Simsek, T., Kahraman, S., Bombaci, E., Sezen, O., Saracoglu, A., & Demirhan, R. (2020). The Psychological Impact of COVID-19 Disease is more Severe on Intensive Care Unit Healthcare Providers: A Cross-sectional Study. *Clinical Psychopharmacology and Neuroscience*, *18*(4), 607-615. [https://doi.org/https://dx.doi.org/10.9758/CPN.2020.18.4.607](https://doi.org/https:/dx.doi.org/10.9758/CPN.2020.18.4.607)
209. Sayik, D., Acikgoz, A., & Kaya, S. (2022). Anxiety and Sleep Quality Levels of Emergency Medical Personnel and Factors Affecting Them During the Coronavirus Disease-2019 Pandemic. *Journal of Turkish Sleep Medicine*, *9*(1), 27-36. [https://doi.org/https://dx.doi.org/10.4274/jtsm.galenos.2021.20982](https://doi.org/https:/dx.doi.org/10.4274/jtsm.galenos.2021.20982)
210. Scott, L. D., Hofmeister, N., Rogness, N., & Rogers, A. E. (2010). An interventional approach for patient and nurse safety: A fatigue countermeasures feasibility study. *Nursing Research*, *59*(4), 250-258. [https://doi.org/https://dx.doi.org/10.1097/NNR.0b013e3181de9116](https://doi.org/https:/dx.doi.org/10.1097/NNR.0b013e3181de9116)
211. Scott, L. D., Hwang, W.-T., & Rogers, A. E. (2006). The impact of multiple care giving roles on fatigue, stress, and work performance among hospital staff nurses. *The Journal of nursing administration*, *36*(2), 86-95. [https://doi.org/https://dx.doi.org/10.1097/00005110-200602000-00007](https://doi.org/https:/dx.doi.org/10.1097/00005110-200602000-00007)
212. Seabra, P. R. C., Lopes, J. M. O., Calado, M. E., & Capelas, M. L. (2019). A national survey of the nurses' mental health - The case of Portugal. *Nurse Forum*, *54*(3), 425-433.<https://doi.org/10.1111/nuf.12350>
213. Segon, T., Kerebih, H., Gashawu, F., Tesfaye, B., Nakie, G., & Anbesaw, T. (2022). Sleep quality and associated factors among nurses working at comprehensive specialized hospitals in Northwest, Ethiopia. *Frontiers in Psychiatry*, *13*(1), 931588. [https://doi.org/https://dx.doi.org/10.3389/fpsyt.2022.931588](https://doi.org/https:/dx.doi.org/10.3389/fpsyt.2022.931588)
214. Seong, J., Son, S., & Min, A. (2022). Effect of sleep on alertness at work among fixed night shift nurses: A prospective observational study. *Journal of Advanced Nursing*, *78*(10), 3197-3206.<https://doi.org/10.1111/jan.15180>
215. Setyowati, L., Chiu, H. Y., Kurnia, A. D., Aini, N., Mashfufa, E. W., & Marta, O. F. D. (2023). The impact of sleep toward executive functions among rapidly rotating shift nurses of emergency departments in Indonesia. *Healthcare in Low-Resource Settings*, *11*(2).<https://doi.org/10.4081/hls.2023.11744>
216. Shao, M., Chou, Y., Yeh, M., & Tzeng, W. C. (2010). Sleep quality and quality of life in female shift-working nurses. *Journal of Advanced Nursing*, *66*(7), 1565-1572. [https://doi.org/https://dx.doi.org/10.1111/j.1365-2648.2010.05300.x](https://doi.org/https:/dx.doi.org/10.1111/j.1365-2648.2010.05300.x)
217. Shehata, R. S. A., Mohamed Nour, Z. A., Abdelrahim Badr, A. M., & Khalifa, E. M. (2021). Serotonin variations and sleep disorders among shift workers. A cross-sectional study. *Toxicology and Industrial Health*, *37*(10), 603-609. [https://doi.org/https://dx.doi.org/10.1177/07482337211033135](https://doi.org/https:/dx.doi.org/10.1177/07482337211033135)
218. Shi, L., Liu, Y., Jiang, T., Yan, P., Cao, F., Chen, Y., Wei, H., & Liu, J. (2020). Relationship between Mental Health, the CLOCK Gene, and Sleep Quality in Surgical Nurses: A Cross-Sectional Study. *BioMed Research International*, *2020*, 4795763. [https://doi.org/https://dx.doi.org/10.1155/2020/4795763](https://doi.org/https:/dx.doi.org/10.1155/2020/4795763)
219. Shiffer, D., Minonzio, M., Dipaola, F., Bertola, M., Zamuner, A. R., Dalla Vecchia, L. A., Solbiati, M., Costantino, G., Furlan, R., & Barbic, F. (2018). Effects of Clockwise and Counterclockwise Job Shift Work Rotation on Sleep and Work-Life Balance on Hospital Nurses. *International journal of environmental research and public health*, *15*(9). [https://doi.org/https://dx.doi.org/10.3390/ijerph15092038](https://doi.org/https:/dx.doi.org/10.3390/ijerph15092038)
220. Shin, S., & Kim, S. (2021). Rotating between day and night shifts: Factors influencing sleep patterns of hospital nurses. *Journal of clinical nursing*, *30*(21-22), 3182-3193. [https://doi.org/https://dx.doi.org/10.1111/jocn.15819](https://doi.org/https:/dx.doi.org/10.1111/jocn.15819)
221. Silva, A. F., Dalri, R. C. M. B., Eckeli, A. L., De Sousa Uva, A. N. P., Mendes, A. M. O. C., & Robazzi, M. L. C. C. (2022). Sleep quality, personal and work variables, and life habits of hospital nurses. *Revista Latino-Americana de Enfermagem*, *30*, e3538. [https://doi.org/https://dx.doi.org/10.1590/1518-8345.5756.3538](https://doi.org/https:/dx.doi.org/10.1590/1518-8345.5756.3538)
222. Sirinara, P., Hanprathet, N., Pongpirul, K., & Jiamjarasrangsi, W. (2019). Impact of shift work on sleep quality among nursing staff. *Chulalongkorn Medical Journal*, *63*(4), 221-227.<https://doi.org/10.14456/clmj.2019.14>
223. Smart, D., & Wilson, M. (2013). Reported sleep health and viral respiratory illness in nurses. *Medsurg nursing: official journal of the Academy of Medical-Surgical Nurses*, *22*(4), 221-227.
224. Spelten, E., Totterdell, P., Barton, J., & Folkard, S. (1995). Effects of age and domestic commitment on the sleep and alertness of female shiftworkers. *Work and Stress*, *9*(3), 165-175. [https://doi.org/http://dx.doi.org/10.1080/02678379508256551](https://doi.org/http:/dx.doi.org/10.1080/02678379508256551)
225. Stavas, J. A., Nilsen, K. B., & Matre, D. (2024). The association between proportion of night shifts and musculoskeletal pain and headaches in nurses: a cross-sectional study. *BMC Musculoskeletal Disorders*, *25*(1), 67. [https://doi.org/https://dx.doi.org/10.1186/s12891-024-07196-5](https://doi.org/https:/dx.doi.org/10.1186/s12891-024-07196-5)
226. Stimpfel, A. W., Fatehi, F., & Kovner, C. (2020). Nurses' sleep, work hours, and patient care quality, and safety. *Sleep health*, *6*(3), 314-320. [https://doi.org/https://dx.doi.org/10.1016/j.sleh.2019.11.001](https://doi.org/https:/dx.doi.org/10.1016/j.sleh.2019.11.001)
227. Su, T. P., Lien, T. C., Yang, C. Y., Su, Y. L., Wang, J. H., Tsai, S. L., & Yin, J. C. (2007). Prevalence of psychiatric morbidity and psychological adaptation of the nurses in a structured SARS caring unit during outbreak: A prospective and periodic assessment study in Taiwan. *Journal of Psychiatric Research*, *41*(2), 119-130.<https://doi.org/10.1016/j.jpsychires.2005.12.006>
228. Suleiman, K., Al-Khateeb, T., Al Kalaldeh, M., & Sharour, L. A. (2020). The Effect of Shift Fluctuations on Sleep Quality among Nurses Working in the Emergency Rooms in Amman, Jordan. *International Journal of Occupational Safety and Health*, *10*(1), 11-17. [https://doi.org/https://dx.doi.org/10.3126/ijosh.v10i1.29877](https://doi.org/https:/dx.doi.org/10.3126/ijosh.v10i1.29877)
229. Suleiman, K., Hijazi, Z., Kalaldeh, M. A., & Sharour, L. A. (2020). Factors Associated with Sleep Quality Among Emergency Nurses in Jordan. *Sleep and Vigilance*, *4*(1), 11-16. [https://doi.org/https://dx.doi.org/10.1007/s41782-019-00082-4](https://doi.org/https:/dx.doi.org/10.1007/s41782-019-00082-4)
230. Sungur, M., Ovayolu, N., Ovayolu, O., & Disli, E. (2022). Sleep quality of hospital nurses during COVID-19 pandemic. *Journal of Nursing and Midwifery Sciences*, *9*(4), 317-323. [https://doi.org/https://dx.doi.org/10.4103/jnms.jnms_32_21](https://doi.org/https:/dx.doi.org/10.4103/jnms.jnms_32_21)
231. Sun, P., Wang, M., Song, T., Wu, Y., Luo, J., Chen, L., & Yan, L. (2021). The Psychological Impact of COVID-19 Pandemic on Health Care Workers: A Systematic Review and Meta-Analysis. *Frontiers in Psychology*, *12*. https://doi.org/10.3389/fpsyg.2021.626547
232. Sveinsdottir, H. (2006). Self-assessed quality of sleep, occupational health, working environment, illness experience and job satisfaction of female nurses working different combination of shifts. *Scandinavian Journal of Caring Sciences*, *20*(2), 229-237. [https://doi.org/https://dx.doi.org/10.1111/j.1471-6712.2006.00402.x](https://doi.org/https:/dx.doi.org/10.1111/j.1471-6712.2006.00402.x)
233. Tada, Y., Kawano, Y., Maeda, I., Yoshizaki, T., Sunami, A., Yokoyama, Y., Matsumoto, H., Hida, A., Komatsu, T., & Togo, F. (2014). Association of body mass index with lifestyle and rotating shift work in Japanese female nurses. *Obesity*, *22*(12), 2489-2493. [https://doi.org/https://dx.doi.org/10.1002/oby.20908](https://doi.org/https:/dx.doi.org/10.1002/oby.20908)
234. Tahvilian, P., Koohestani, S., Maghooli, K., Jafarnia Dabanloo, N., Sarhangi Kadijani, M., Jahantigh, S., Zangeneh Soroush, M., & Saliani, A. (2022). Effects of COVID-19-related psychological distress and anxiety on quality of sleep and life in healthcare workers in Iran and three European countries. *Frontiers in public health*, *10*, 997626. [https://doi.org/https://dx.doi.org/10.3389/fpubh.2022.997626](https://doi.org/https:/dx.doi.org/10.3389/fpubh.2022.997626)
235. Tamrakar, P., Pant, S. B., & Acharya, S. P. (2023). Anxiety and depression among nurses in COVID and non-COVID intensive care units. *Nursing in Critical Care*, *28*(2), 272-280. [https://doi.org/https://dx.doi.org/10.1111/nicc.12685](https://doi.org/https:/dx.doi.org/10.1111/nicc.12685)
236. Taylor, D. J., Dietch, J. R., Wardle-Pinkston, S., Slavish, D. C., Messman, B., Ruggero, C. J., & Kelly, K. (2022). Shift Work Disorder Index: initial validation and psychosocial associations in a sample of nurses. *Journal of Clinical Sleep Medicine*, *18*(10), 2339-2351. [https://doi.org/https://dx.doi.org/10.5664/jcsm.10108](https://doi.org/https:/dx.doi.org/10.5664/jcsm.10108)
237. Thun, E., Waage, S., Bjorvatn, B., Moen, B. E., Vedaa, O., Blytt, K. M., & Pallesen, S. (2021). Short sleep duration and high exposure to quick returns are associated with impaired everyday memory in shift workers. *Nursing Outlook*, *69*(3), 293-301. [https://doi.org/https://dx.doi.org/10.1016/j.outlook.2020.09.008](https://doi.org/https:/dx.doi.org/10.1016/j.outlook.2020.09.008)
238. Tian, Y., Yue, Y., Liao, X., Wang, J., Ye, M., Liu, Y., Li, Y., & Zhou, J. (2021). Mental health service use and its associated factors among nurses in China: A cross-sectional survey. *PeerJ*, *9*, 11181. [https://doi.org/https://dx.doi.org/10.7717/peerj.11181](https://doi.org/https:/dx.doi.org/10.7717/peerj.11181)
239. Togo, F., Yoshizaki, T., & Komatsu, T. (2017). Association between depressive symptoms and morningness-eveningness, sleep duration and rotating shift work in Japanese nurses. *Chronobiology international*, *34*(3), 349-359. [https://doi.org/https://dx.doi.org/10.1080/07420528.2016.1273942](https://doi.org/https:/dx.doi.org/10.1080/07420528.2016.1273942)
240. Topal Kilincarslan, G., Ozcan Algul, A., & Gordeles Beser, N. (2024). Sleep quality, coping, and related depression: A cross-sectional study of Turkish nurses. *International nursing review*. [https://doi.org/https://dx.doi.org/10.1111/inr.12928](https://doi.org/https:/dx.doi.org/10.1111/inr.12928)
241. Tosun, A., Tosun, H., Odul Ozkaya, B., Erdogan, Z., & Gul, A. (2022). "Sleep Quality and Depression Level in Nurses in COVID-19 Pandemic". *Omega*, 302228221123159. [https://doi.org/https://dx.doi.org/10.1177/00302228221123159](https://doi.org/https:/dx.doi.org/10.1177/00302228221123159)
242. Totterdell, P., Spelten, E., Smith, L., Barton, J., & Folkard, S. (1995). Recovery From Work Shifts: How Long Does It Take? *Journal of applied psychology*, *80*(1), 43-57.<https://doi.org/10.1037/0021-9010.80.1.43>
243. Tsai, J.-C., Chou, K.-R., Tsai, H.-T., Yen, Y.-C., & Niu, S.-F. (2019). Effects of Nocturnal Sleep Quality on Diurnal Cortisol Profiles and Attention in Nurses: A Cross-Sectional Study. *Biological Research for Nursing*, *21*(5), 510-518. [https://doi.org/https://dx.doi.org/10.1177/1099800419861695](https://doi.org/https:/dx.doi.org/10.1177/1099800419861695)
244. Tsou, M. T., Pai, T. P., Chiang, T. M., Huang, W. H., Lin, H. M., & Lee, S. C. (2021). Burnout and metabolic syndrome among different departments of medical center nurses in Taiwan-Cross-sectional study and biomarker research. *Journal of Occupational Health*, *63*(1), 12188. [https://doi.org/https://dx.doi.org/10.1002/1348-9585.12188](https://doi.org/https:/dx.doi.org/10.1002/1348-9585.12188)
245. Tu, Z.-H., He, J.-W., & Zhou, N. (2020). Sleep quality and mood symptoms in conscripted frontline nurse in Wuhan, China during COVID-19 outbreak: A cross-sectional study. *Medicine*, *99*(26), 20769. [https://doi.org/https://dx.doi.org/10.1097/MD.0000000000020769](https://doi.org/https:/dx.doi.org/10.1097/MD.0000000000020769)
246. Ulusoy, H. G., Sanlier, N., & Rakicioglu, N. (2021). Do Rotating Night Shifts Change Nurses' Nutritional Status? A Cross-Sectional Study. *Journal of the American College of Nutrition*. [https://doi.org/https://dx.doi.org/10.1080/07315724.2021.1947413](https://doi.org/https:/dx.doi.org/10.1080/07315724.2021.1947413)
247. Van de Langenberg, D., Vlaanderen, J., Berentzen, N., Kromhout, H., & Vermeulen, R. (2023). Associating night-shift work with lifetime use of sleep medication and sleep quality in a cohort of female nurses. *Annals of work exposures and health*, *67*(9), 1056-1068. [https://doi.org/https://dx.doi.org/10.1093/annweh/wxad058](https://doi.org/https:/dx.doi.org/10.1093/annweh/wxad058)
248. Vedaa, O., Morland, E., Larsen, M., Harris, A., Erevik, E., Sivertsen, B., Bjorvatn, B., Waage, S., & Pallesen, S. (2017). Sleep Detriments Associated With Quick Returns in Rotating Shift Work: A Diary Study. *Journal of Occupational and Environmental Medicine*, *59*(6), 522-527. [https://doi.org/https://dx.doi.org/10.1097/JOM.0000000000001006](https://doi.org/https:/dx.doi.org/10.1097/JOM.0000000000001006)
249. Veal, B. M., Mu, C. X., Small, B. J., & Lee, S. (2021). Subjective cognitive abilities correlate with poor sleep among day-shift and night-shift nurses. *Journal of Sleep Research*, *30*(5), 13359. [https://doi.org/https://dx.doi.org/10.1111/jsr.13359](https://doi.org/https:/dx.doi.org/10.1111/jsr.13359)
250. Verhaegen, P., Cober, R., de Smedt, M., Dirkx, J., Kerstens, J., Ryvers, D., & van Dael, P. (1987). The adaptation of night nurses to different work schedules. *Ergonomics*, *30*(9), 1301-1309.<https://doi.org/10.1080/00140138708966024>
251. Vijaykumar, N., Kiran, S., & Karne, S. L. (2018). Influence of altered circadian rhythm on quality of sleep and its association with cognition in shift nurses. *National Journal of Physiology, Pharmacyand Pharmacology*, *8*(5), 643-649. [https://doi.org/https://dx.doi.org/10.5455/njppp.2018.8.1144629112017](https://doi.org/https:/dx.doi.org/10.5455/njppp.2018.8.1144629112017)
252. Wang, D., Lin, B., Liang, H., Deng, Y., & Zhang, L. (2023). Mediating role of psychological flexibility in the effect of obsessive-compulsive symptoms on sleep quality among nurses during the COVID-19 pandemic. *Current Psychology*.<https://doi.org/10.1007/s12144-023-04546-9>
253. Wang, D., Lin, B., Xiong, F., Deng, Y., & Zhang, L. (2023). Effectiveness of Internet-delivered self-help acceptance and commitment therapy (iACT) on nurses' obsessive-compulsive symptoms and sleep quality: A randomized controlled trial with 3-month follow-up. *Journal of Affective Disorders*, *341*, 319-328. [https://doi.org/https://dx.doi.org/10.1016/j.jad.2023.08.125](https://doi.org/https:/dx.doi.org/10.1016/j.jad.2023.08.125)
254. Wang, H., Shen, J. H., Dai, J., Zhu, J. X., Tong, L., & Zhang, X. Q. (2024). Associations between sleep quality and coping style among front-line nurses: a cross-sectional study in China. *Journal of Public Health (Germany)*. [https://doi.org/https://dx.doi.org/10.1007/s10389-024-02245-8](https://doi.org/https:/dx.doi.org/10.1007/s10389-024-02245-8)
255. Wang, J., Zheng, Z., Tang, Y., Zhang, R., Lu, Q., Wang, B., & Sun, Q. (2022). Psychological distress and its influencing factors among psychiatric nurses in China: A cross-sectional study. *Frontiers in Psychiatry*, *13*, 948786. [https://doi.org/https://dx.doi.org/10.3389/fpsyt.2022.948786](https://doi.org/https:/dx.doi.org/10.3389/fpsyt.2022.948786)
256. Wang, L., Wu, Y. X., Lin, Y. Q., Zeng, Z. N., Xie, X. L., Chen, Q. Y., & Wei, S. C. (2022). Reliability and validity of the Pittsburgh Sleep Quality Index among frontline COVID-19 health care workers using classical test theory and item response theory. *Journal of Clinical Sleep Medicine*, *18*(2), 541-551.<https://doi.org/10.5664/jcsm.9658>
257. Wang, X. G., Luo, Y. Y., & Li, S. R. (2020). The impact of sleep quality on reported anxiety and fatigue among shift nurses: The mediating role of resilience. *Journal of Integrative Nursing*, *2*(4), 190-195.<https://doi.org/10.4103/jin.jin_39_20>
258. Watanabe, K., Sugimura, N., Shishido, I., Konya, I., Yamaguchi, S., & Yano, R. (2022). Effects of 90 Min Napping on Fatigue and Associated Environmental Factors among Nurses Working Long Night Shifts: A Longitudinal Observational Study. *International journal of environmental research and public health*, *19*(15), 9429. [https://doi.org/https://dx.doi.org/10.3390/ijerph19159429](https://doi.org/https:/dx.doi.org/10.3390/ijerph19159429)
259. Weaver, S. H., Dimino, K., Fleming, K., Harvey, J., Manzella, M., O'Neill, P., Paliwal, M., Phillips, M., & Wurmser, T. A. (2024). Exploring Sleep and Fatigue of Clinical Nurses and Administrative Supervisors. *Nurse Leader*, *22*(2), 203-210. [https://doi.org/https://dx.doi.org/10.1016/j.mnl.2023.11.010](https://doi.org/https:/dx.doi.org/10.1016/j.mnl.2023.11.010)
260. Winyuchakrit, I., & Taneepanichskul, N. (2017). Premenstrual syndrome and sleep quality among private hospital nurses in Bangkok, Thailand. *Journal of Health Research*, *31*, 217-224. [https://doi.org/https://dx.doi.org/10.14456/jhr.2017.87](https://doi.org/https:/dx.doi.org/10.14456/jhr.2017.87)
261. Wu, S., Wu, C., Wang, X., Fei, W., & Fu, Y. (2023). Mediating effect of circadian rhythm between work stress and sleep quality in Chinese shift-working nurses: A cross-sectional survey. *Nursing open*, *10*(2), 560-569. [https://doi.org/https://dx.doi.org/10.1002/nop2.1322](https://doi.org/https:/dx.doi.org/10.1002/nop2.1322)
262. Wu, Y., Guo, Y., Xu, N., Zhang, H., Xiu, Y., Lin, D., & Ying, W. (2023). Risk factors for post-traumatic stress disorder in nurses from the regional medical alliance during the COVID-19 epidemic: A prospective cross-sectional*and Pharmacology*, *8*(5), 643-649. [https://doi.org/https://dx.doi.org/10.5455/njppp.2018.8.1144629112017](https://doi.org/https:/dx.doi.org/10.5455/njppp.2018.8.1144629112017)
263. Xi, S., Gu, Y., Guo, H., Jin, B., Guo, F., Miao, W., & Zhang, L. (2022). Sleep quality status, anxiety, and depression status of nurses in infectious disease department. *Frontiers in Psychology*, *13,* 947948.<https://doi.org/10.3389/fpsyg.2022.947948>
264. Yamasaki, F., Schwartz, J. E., Gerber, L. M., Warren, K., & Pickering, T. G. (1998). Impact of shift work and race/ethnicity on the diurnal rhythm of blood pressure and catecholamines. *Hypertension*, *32*(3), 417-423. [https://doi.org/http://dx.doi.org/10.1161/01.HYP.32.3.417](https://doi.org/http:/dx.doi.org/10.1161/01.HYP.32.3.417)
265. Yan, L. J., & Xie, Y. J. (2022). Associations Between Sleep Quality and 10-Year Cardiovascular Disease Risk Among Female Nurses in Hong Kong: A Cross-sectional Study. *The Journal of cardiovascular nursing*, *37*(3), 22-31. [https://doi.org/https://dx.doi.org/10.1097/JCN.0000000000000857](https://doi.org/https:/dx.doi.org/10.1097/JCN.0000000000000857)
266. Yin, M., Li, W., Yang, Q., Yue, Y., Fang, X., Yang, Z., Wang, X., Liu, Q., Kong, F., Ji, C., Lv, X., Wang, H., Yuan, N., Li, Z., Zhang, C., Li, K., Yang, Y., & Du, X. (2022). The Mediating Role of Coping Style in the Relationship Between Sleep Quality and Burnout: A Cross-Sectional Study Among Psychiatric Nurses. *Frontiers in Psychiatry*, *13*, 926040. [https://doi.org/https://dx.doi.org/10.3389/fpsyt.2022.926040](https://doi.org/https:/dx.doi.org/10.3389/fpsyt.2022.926040)
267. Yoo, J., Slavish, D., Dietch, J. R., Kelly, K., Ruggero, C., & Taylor, D. J. (2023). Daily reactivity to stress and sleep disturbances: unique risk factors for insomnia. *Sleep*, *46*(2). [https://doi.org/https://dx.doi.org/10.1093/sleep/zsac256](https://doi.org/https:/dx.doi.org/10.1093/sleep/zsac256)
268. Yu, C., Zhang, X., Wang, Y., Mao, F., & Cao, F. (2024). Stress begets stress: The moderating role of childhood adversity in the relationship between job stress and sleep quality among nurses. *Journal of Affective Disorders*, *348*, 345-352. [https://doi.org/https://dx.doi.org/10.1016/j.jad.2023.12.090](https://doi.org/https:/dx.doi.org/10.1016/j.jad.2023.12.090)
269. Yu, F., Cavadino, A., Mackay, L., Ward, K., King, A., & Smith, M. (2022). A cluster analysis of physical activity profiles and resilience in intensive care nurses. *International Journal of Workplace Health Management*, *15*(2), 174-192. [https://doi.org/https://dx.doi.org/10.1108/IJWHM-04-2021-0082](https://doi.org/https:/dx.doi.org/10.1108/IJWHM-04-2021-0082)
270. Yuan, M.-Z., & Fang, Q. (2024). Latent class analysis of the sleep quality of night shift nurses and impact of shift-related factors on the occupational stress and anxiety. *Journal of Advanced Nursing*. [https://doi.org/https://dx.doi.org/10.1111/jan.16067](https://doi.org/https:/dx.doi.org/10.1111/jan.16067)
271. Zadeh, B. Q., Hadadian, F., Salari, N., Maazinezhad, S., & Paveh, B. K. (2018). The effect of transcotaneus electrical acupoint stimulation on sleep quality in nurses. *Journal of Kermanshah University of Medical Sciences*, *22*(1), 68669. [https://doi.org/https://dx.doi.org/10.5812/jkums.68669](https://doi.org/https:/dx.doi.org/10.5812/jkums.68669)
272. Zamanian, Z., Nikeghbal, K., & Khajehnasiri, F. (2016). Influence of Sleep on Quality of Life Among Hospital Nurses. *Electronic physician*, *8*(1), 1811-1816. [https://doi.org/https://dx.doi.org/10.19082/1811](https://doi.org/https:/dx.doi.org/10.19082/1811)
273. Zarei, S., & Fooladvand, K. (2022). Mediating effect of sleep disturbance and rumination on work-related burnout of nurses treating patients with coronavirus disease. *BMC psychology*, *10*(1), 197. [https://doi.org/https://dx.doi.org/10.1186/s40359-022-00905-6](https://doi.org/https:/dx.doi.org/10.1186/s40359-022-00905-6)
274. Zarzour, M., Hachem, C., Kerbage, H., Richa, S., El Choueifaty, D., Saliba, G., Haddad, E., Melki, E., Sleilaty, G., Saliby, R., Sabbagh, C., & Choucair, J. (2022). Anxiety and sleep quality in a sample of Lebanese healthcare workers during the COVID-19 outbreak. *L'Encephale: Revue de psychiatrie clinique biologique et therapeutique*, *48*(5), 496-503. [https://doi.org/https://dx.doi.org/10.1016/j.encep.2021.06.016](https://doi.org/https:/dx.doi.org/10.1016/j.encep.2021.06.016)
275. Zhang, C.-Q., Zhang, R., Lu, Y., Liu, H., Kong, S., Baker, J. S., & Zhang, H. (2021). Occupational stressors, mental health, and sleep difficulty among nurses during the COVID-19 pandemic: The mediating roles of cognitive fusion and cognitive reappraisal. *Journal of Contextual Behavioral Science*, *19*, 64-71. [https://doi.org/https://dx.doi.org/10.1016/j.jcbs.2020.12.004](https://doi.org/https:/dx.doi.org/10.1016/j.jcbs.2020.12.004)
276. Zhang, X., Dai, X., Jiao, J., & Lee, S.-Y. (2023). Impact of sleep-wake features on fatigue among female shift work nurses. *Annals of medicine*, *55*(1), 2210843. [https://doi.org/https://dx.doi.org/10.1080/07853890.2023.2210843](https://doi.org/https:/dx.doi.org/10.1080/07853890.2023.2210843)
277. Zhang, Y., Tang, M., & Zhou, Y. (2023). Holistic sleep improvement strategies for frontline nurses served during a public health emergency (COVID-19) in Wuhan, China: A quasi-experimental study. *Nursing open*, *10*(3), 1471-1481. [https://doi.org/https://dx.doi.org/10.1002/nop2.1397](https://doi.org/https:/dx.doi.org/10.1002/nop2.1397)
278. Zhou, H.-Q., Yao, M., Chen, Y.-W., Huang, J.-Y., & Chen, G.-Y. (2017). Functional Gastrointestinal Disorders Common Among Nurses With Poor Sleep Quality in Shanghai, China: A Pilot Study. *Gastroenterology nursing: the official journal of the Society of Gastroenterology Nurses and Associates*, *40*(4), 312-319. [https://doi.org/https://dx.doi.org/10.1097/SGA.0000000000000176](https://doi.org/https:/dx.doi.org/10.1097/SGA.0000000000000176)
279. Zion, N., & Shochat, T. (2018). Cognitive functioning of female nurses during the night shift: The impact of age, clock time, time awake and subjective sleepiness. *Chronobiology international*, *35*(11), 1595-1607. [https://doi.org/https://dx.doi.org/10.1080/07420528.2018.1497642](https://doi.org/https:/dx.doi.org/10.1080/07420528.2018.1497642)
280. Zion, N., & Shochat, T. (2019). Let them sleep: The effects of a scheduled nap during the night shift on sleepiness and cognition in hospital nurses. *Journal of Advanced Nursing*, *75*(11), 2603-2615. [https://doi.org/https://dx.doi.org/10.1111/jan.14031](https://doi.org/https:/dx.doi.org/10.1111/jan.14031)
