# Supplementary material for: Trends in Nurses’ Sleep: A Meta‐Regression Involving 219,245 Nurses From 45 Countries
Source: J Nurs Manag. 2026 Aug 27;2026:9051680. doi: 10.1155/jonm/9051680 (PMC13522381; doi:10.1155/jonm/9051680)
Supplement: Supplementary file 2 [file JONM-2026-9051680-s002.docx]

**Supplementary File 3: Excluded studies and reasons.**

| **Reference** | **Exclusion Reason** | |
| --- | --- | --- |
| Rashidi, M. A., Aghl, M. R. P., Ahmadi, O., Pournajaf, A., Seififard, M., Kazemi, M., & Pournajaf, M. (2020). Occupational fatigue and sleep quality: A comparison between nurses working in various wards of public hospitals. *Journal of Advances in Medical and Biomedical Research, 28*(131), 336-345. https://doi.org/10.30699/jambs.28.131.336 | | Age not reported |
| [Weaver, A. L., Stutzman, S. E., Supnet, C., & Olson, D. M. (2016). Sleep quality, but not quantity, is associated with self-perceived minor error rates among emergency department nurses. *International emergency nursing, 25,* 48-52. https://doi.org/https://dx.doi.org/10.1016/j.ienj.2015.08.003](https://doi.org/https:/dx.doi.org/10.1016/j.ienj.2015.08.003) | | Age not reported |
| [Sim, S., Lee, S., & Bang, M. (2019). Relationship among sleep quality, fatigue, resilience, and nursing performance ability in shift work nurses. *Indian Journal of Public Health Research and Development, 10*(11), 4659-4663. https://doi.org/https://dx.doi.org/10.5958/0976-5506.2019.04341.9](https://doi.org/https:/dx.doi.org/10.5958/0976-5506.2019.04341.9) | | Age not reported |
| [Seki, Y., & Yamazaki, Y. (2006). Effects of working conditions on intravenous medication errors in a Japanese hospital. *Journal of Nursing Management, 14*(2), 128-139. https://doi.org/https://dx.doi.org/10.1111/j.1365-2934.2006.00597.x](https://doi.org/https:/dx.doi.org/10.1111/j.1365-2934.2006.00597.x) | | Age not reported |
| [De Negreiros, C. T. F., da Silva, S. R. S., da Silva, R. M., Costa, A. L. S., Farias, F. C., Cangussu, D. D. D., & Kimura, C. A. (2019). Association of sleep quality and academic profile with stress of nursing students. *Revista Baiana de Enfermagem, 33*, e33482. https://doi.org/https://dx.doi.org/10.18471/rbe.v33.33482](https://doi.org/https:/dx.doi.org/10.18471/rbe.v33.33482) | | Age not reported |
| [Wu, X., Lu, Y., Xie, X., Chen, R., Zhang, N., Zhou, C., & Ye, Z. (2022). Association between circadian rhythm and sleep quality among nursing interns: A latent profile and moderation analysis. *Frontiers in Neuroscience, 16*, 995775. https://doi.org/https://dx.doi.org/10.3389/fnins.2022.995775](https://doi.org/https:/dx.doi.org/10.3389/fnins.2022.995775) | | Age not reported |
| [Sadeghniiat-Haghighi, K., Aminian, O., Pouryaghoub, G., & Yazdi, Z. (2008). Efficacy and hypnotic effects of melatonin in shift-work nurses: Double-blind, placebo-controlled crossover trial. *Journal of Circadian Rhythms, 6,* 10. https://doi.org/https://dx.doi.org/10.1186/1740-3391-6-10](https://doi.org/https:/dx.doi.org/10.1186/1740-3391-6-10) | | Clinical sample |
| [Ota, T., Tan, C., Ishii, A., & Shiotani, H. (2022). Do differences in chronotypes affect sleep and health-related quality of life of nursing students? A cross-sectional study. *Chronobiology International, 39*(11), 1435-1443. https://doi.org/https://dx.doi.org/10.1080/07420528.2022.2117049](https://doi.org/https:/dx.doi.org/10.1080/07420528.2022.2117049) | | Clinical sample |
| [Lu, Y. e., Li, Y., Huang, Y., Zhang, X., Wang, J., Wu, L., & Cao, F. (2023). Effects and mechanisms of a web- and mobile-based acceptance and commitment therapy intervention for anxiety and depression symptoms in nurses: Fully decentralized randomized controlled trial. *Journal of Medical Internet Research, 25*. https://doi.org/https://dx.doi.org/10.2196/51549](https://doi.org/https:/dx.doi.org/10.2196/51549) | | Clinical sample |
| [Hsu, H.-C., Lin, M.-H., Lee, H.-F., Wu, C.-Y., Chou, C.-T., & Lin, S.-F. (2021). Effects of aromatherapy with essential oil massage on the sleep quality of critical care nurses: A randomized controlled trial. *Complementary therapies in clinical practice, 43,* 101358. https://doi.org/https://dx.doi.org/10.1016/j.ctcp.2021.101358](https://doi.org/https:/dx.doi.org/10.1016/j.ctcp.2021.101358) | | Clinical sample |
| [Chueh, K.-H., Chang, C.-C., & Yeh, M.-L. (2018). Effects of Auricular Acupressure on Sleep Quality, Anxiety, and Depressed Mood in RN-BSN Students With Sleep Disturbance. *The journal of nursing research : JNR, 26(*1), 10-17. https://doi.org/https://dx.doi.org/10.1097/JNR.0000000000000209](https://doi.org/https:/dx.doi.org/10.1097/JNR.0000000000000209) | | Clinical sample |
| [Zhou, K., Kong, J., Wan, Y., Zhang, X., Liu, X., Qu, B., Wang, B., & Xue, R. (2022). Positive impacts of e-aid cognitive behavioural therapy on the sleep quality and mood of nurses on site during the COVID-19 pandemic. *Sleep and Breathing, 26*(4), 1947-1951. https://doi.org/https://dx.doi.org/10.1007/s11325-021-02547-1](https://doi.org/https:/dx.doi.org/10.1007/s11325-021-02547-1) | | Clinical sample |
| [Bai, J., & Cheng, C. (2022). The relationship between health-promoting behaviors, menopause syndrome, and sleep quality in Chinese middle-aged nurses: A cross-sectional study. *Japan journal of nursing science : JJNS, 19(*1), e12443. https://doi.org/https://dx.doi.org/10.1111/jjns.12443](https://doi.org/https:/dx.doi.org/10.1111/jjns.12443) | | Clinical sample |
| [Kavurmaci, M., Dayapoglu, N., & Tan, M. (2020). Effect of Music Therapy on Sleep Quality. *Alternative Therapies in Health and Medicine, 26*(4), 22-26. http://ovidsp.ovid.com/ovidweb.cgi?T=JS&PAGE=reference&D=emed21&NEWS=N&AN=628438987](http://ovidsp.ovid.com/ovidweb.cgi?T=JS&PAGE=reference&D=emed21&NEWS=N&AN=628438987) | | Clinical sample |
| [Chang, W. L., Ko, S. H., Chang, T. H., & Hung, L. C. (2010). Effect of auricular acupressure on improving sleep quality in nurses. *Journal of Nursing and Healthcare Research, 6*(4), 261-270. https://www.scopus.com/inward/record.uri?eid=2-s2.0-84865133315&partnerID=40&md5=b1116b9e9e2291b9ce46a5461ebf2938](https://www.scopus.com/inward/record.uri?eid=2-s2.0-84865133315&partnerID=40&md5=b1116b9e9e2291b9ce46a5461ebf2938) | | Clinical sample |
| [F. (2019). A comparison of early versus late nap times during the night shift regarding the psychomotor performance required for safe driving: A pilot study. *Shiraz E Medical Journal, 20*(11), e86980. https://doi.org/https://dx.doi.org/10.5812/semj.86980](https://doi.org/https:/dx.doi.org/10.5812/semj.86980) | | Inadequate sample size |
| [Harrison, E. M., Schmied, E. A., Easterling, A. P., Yablonsky, A. M., & Glickman, G. L. (2020). A hybrid effectiveness-implementation study of a multi-component lighting intervention for hospital shift workers. *International Journal of Environmental Research and Public Health, 17*(23), 1-10. https://doi.org/https://dx.doi.org/10.3390/ijerph17239141](https://doi.org/https:/dx.doi.org/10.3390/ijerph17239141) | | Inadequate sample size |
| [Rodrigues, G. H., Duarte, A. D. S., Garrido, A. L. F., Teixeira Santana, P., Pellegrino, P., Nogueira, L. F. R., Crispim, C. A., Cipolla-Neto, J., Moreno, C. R. D. C., & Marqueze, E. C. (2022). A putative association between food intake, meal timing and sleep parameters among overweight nursing professionals working night shifts. *Sleep Epidemiology, 2*, 100040. https://doi.org/https://dx.doi.org/10.1016/j.sleepe.2022.100040](https://doi.org/https:/dx.doi.org/10.1016/j.sleepe.2022.100040) | | Inadequate sample size |
| Aotaweesuk, N., Bumrongsawat, S., Ongiem, A., & Vichitvejapisal, P. (2018). A stress survey in nurse anesthetist students. *Journal of the Medical Association of Thailand*, *101*(12), 1646-1652. | | Inadequate sample size |
| [Clissold, G., Smith, P., Accutt, B., & Di Milia, L. (2002). A study of female nurses combining partner and parent roles with working a continuous three-shift roster: the impact on sleep, fatigue and stress. *Contemporary nurse : a journal for the Australian nursing profession, 12*(3), 294-302. http://ovidsp.ovid.com/ovidweb.cgi?T=JS&PAGE=reference&D=emed7&NEWS=N&AN=35632474](http://ovidsp.ovid.com/ovidweb.cgi?T=JS&PAGE=reference&D=emed7&NEWS=N&AN=35632474) | | Inadequate sample size |
| [Guleryuz, O. D., & Fidanci, I. (2020). AN analysis on sleep quality of the healthcare professionals during the COVID- 19 pandemic. *Acta Medica Mediterranea, 36*(6), 3797-3800. https://doi.org/https://dx.doi.org/10.19193/0393-6384_2020_6_601](https://doi.org/https:/dx.doi.org/10.19193/0393-6384_2020_6_601) | | Inadequate sample size |
| [Aarts, M. P. J., Hartmeyer, S. L., Morsink, K., Kort, H. S. M., & de Kort, Y. A. W. (2020). Can Special Light Glasses Reduce Sleepiness and Improve Sleep of Nightshift Workers? A Placebo-Controlled Explorative Field Study. *Clocks and Sleep, 2*(2), 225-245. https://doi.org/10.3390/clockssleep2020018](https://doi.org/10.3390/clockssleep2020018) | | Inadequate sample size |
| [Cho, H., Brzozowski, S., Arsenault Knudsen, E. N., & Steege, L. M. (2021). Changes in Fatigue Levels and Sleep Measures of Hospital Nurses During Two 12-Hour Work Shifts. *The Journal of nursing administration, 51*(3), 128-134. https://doi.org/https://dx.doi.org/10.1097/NNA.0000000000000983](https://doi.org/https:/dx.doi.org/10.1097/NNA.0000000000000983) | | Inadequate sample size |
| [Wu, X., Bai, F., Wang, Y., Zhang, L., Liu, L., Chen, Y., Li, H., & Zhang, T. (2021). Circadian Rhythm Disorders and Corresponding Functional Brain Abnormalities in Young Female Nurses: A Preliminary Study. *Frontiers in Neurology, 12,* 664610. https://doi.org/https://dx.doi.org/10.3389/fneur.2021.664610](https://doi.org/https:/dx.doi.org/10.3389/fneur.2021.664610) | | Inadequate sample size |
| [Ferreira, L. R. C., Miguel, M. A. L., Figueiredo De Martino, M. M., & Menna-Barreto, L. (2013). Circadian rhythm of wrist temperature and night shift-work. *Biological Rhythm Research, 44*(5), 737-744. https://doi.org/https://dx.doi.org/10.1080/09291016.2012.739931](https://doi.org/https:/dx.doi.org/10.1080/09291016.2012.739931) | | Inadequate sample size |
| [Jarnefelt, H., Lagerstedt, R., Kajaste, S., Sallinen, M., Savolainen, A., & Hublin, C. (2012). Cognitive behavioral therapy for shift workers with chronic insomnia. *Sleep Medicine, 13*(10), 1238-1246. https://doi.org/https://dx.doi.org/10.1016/j.sleep.2012.10.003](https://doi.org/https:/dx.doi.org/10.1016/j.sleep.2012.10.003) | | Inadequate sample size |
| [Bryden, G., & Holdstock, T. L. (1973). Effects of night duty on sleep patterns of nurses. *Psychophysiology, 10*(1), 36-42. https://doi.org/https://dx.doi.org/10.1111/j.1469-8986.1973.tb01080.x](https://doi.org/https:/dx.doi.org/10.1111/j.1469-8986.1973.tb01080.x) | | Inadequate sample size |
| [Lowson, E., Middleton, B., Arber, S., & Skene, D. J. (2013). Effects of night work on sleep, cortisol and mood of female nurses, their husbands and children. *Sleep and Biological Rhythms, 11*(1), 7-13. https://doi.org/https://dx.doi.org/10.1111/j.1479-8425.2012.00585.x](https://doi.org/https:/dx.doi.org/10.1111/j.1479-8425.2012.00585.x) | | Inadequate sample size |
| [Manacci, C., Rogers, K., Martin, G., Kovach, B., Mancuso, C., & Fallon, W. (1999). Efficacy of 24-hour shifts: prepared or impaired? A prospective study. *Air medical journal, 18*(1), 20-25. http://ovidsp.ovid.com/ovidweb.cgi?T=JS&PAGE=reference&D=emed6&NEWS=N&AN=129402890](http://ovidsp.ovid.com/ovidweb.cgi?T=JS&PAGE=reference&D=emed6&NEWS=N&AN=129402890) | | Inadequate sample size |
| [Costa, G., Ghirlanda, G., Tarondi, G., Minors, D., & Waterhouse, J. (1994). Evaluation of a rapidly rotating shift system for tolerance of nurses to nightwork. *International Archives of Occupational and Environmental Health, 65*(5), 305-311. http://ovidsp.ovid.com/ovidweb.cgi?T=JS&PAGE=reference&D=emed5&NEWS=N&AN=24083177](http://ovidsp.ovid.com/ovidweb.cgi?T=JS&PAGE=reference&D=emed5&NEWS=N&AN=24083177) | | Inadequate sample size |
| [workshop on recovery from job stress for radiation therapists and oncology nurses: A randomised trial. *Journal of Medical Imaging and Radiation Oncology, 59*(4), 491-498. https://doi.org/https://dx.doi.org/10.1111/1754-9485.12322](https://doi.org/https:/dx.doi.org/10.1111/1754-9485.12322) | | Inadequate sample size |
| [Sakai, S., Nagata, M., Nagata, T., & Mori, K. (2021). Improved Sleep Quality and Work Performance Among Shift Workers Consuming a "Foods with Function Claims" Containing Asparagus Extract. *Journal of UOEH, 43*(1), 15-23. https://doi.org/https://dx.doi.org/10.7888/juoeh.43.15](https://doi.org/https:/dx.doi.org/10.7888/juoeh.43.15) | | Inadequate sample size |
| [Boivin, D. B., Boudreau, P., James, F. O., & Kin, N. M. K. N. Y. (2012). Photic resetting in night-shift work: Impact on nurses' sleep. *Chronobiology International, 29*(5), 619-628. https://doi.org/https://dx.doi.org/10.3109/07420528.2012.675257](https://doi.org/https:/dx.doi.org/10.3109/07420528.2012.675257) | | Inadequate sample size |
| [Roskoden, F. C., Kruger, J., Vogt, L. J., Gartner, S., Hannich, H. J., Steveling, A., Lerch, M. M., & Aghdassi, A. A. (2017). Physical activity, energy expenditure, nutritional habits, quality of sleep and stress levels in shift-working health care personnel. *PLoS ONE, 12*(1), e0169983. https://doi.org/https://dx.doi.org/10.1371/journal.pone.0169983](https://doi.org/https:/dx.doi.org/10.1371/journal.pone.0169983) | | Inadequate sample size |
| [Chung, M.-H., Kuo, T. B. J., Hsu, N., Chu, H., Chou, K.-R., & Yang, C. C. H. (2012). Recovery after three-shift work: relation to sleep-related cardiac neuronal regulation in nurses. *Industrial Health, 50*(1), 24-30. https://doi.org/https://dx.doi.org/10.2486/indhealth.ms1305](https://doi.org/https:/dx.doi.org/10.2486/indhealth.ms1305) | | Inadequate sample size |
| [Wang, X., Jiang, X., Huang, Q., Wang, H., Gurarie, D., Ndeffo-Mbah, M., Fan, F., Fu, P., Horn, M. A., Mondal, A., King, C., Xu, S., Zhao, H., & Bai, Y. (2020). Risk factors of SARS-CoV-2 infection in healthcare workers: a retrospective study of a nosocomial outbreak. *Sleep Medicine, 2,* 100028. https://doi.org/https://dx.doi.org/10.1016/j.sleepx.2020.100028](https://doi.org/https:/dx.doi.org/10.1016/j.sleepx.2020.100028) | | Inadequate sample size |
| [Carter, P. A., Dyer, K. A., & Mikan, S. Q. (2013). Sleep disturbance, chronic stress, and depression in hospice nurses: Testing the feasibility of an intervention. *Oncology Nursing Forum, 40*(5), E368-E373. https://doi.org/https://dx.doi.org/10.1188/13.ONF.E368-E373](https://doi.org/https:/dx.doi.org/10.1188/13.ONF.E368-E373) | | Inadequate sample size |
| [Rheaume, A., & Mullen, J. (2018). The impact of long work hours and shift work on cognitive errors in nurses. *Journal of Nursing Management, 26*(1), 26-32. https://doi.org/https://dx.doi.org/10.1111/jonm.12513](https://doi.org/https:/dx.doi.org/10.1111/jonm.12513) | | Inadequate sample size |
| [Clissold, G., Smith, P., & Acutt, B. (2001). The impact of unwaged domestic work on the duration and timing of sleep of female nurses working full-time on rotating 3-shift rosters. *Journal of human ergology, 30*(1-2), 345-349. http://ovidsp.ovid.com/ovidweb.cgi?T=JS&PAGE=reference&D=emed7&NEWS=N&AN=137559300](http://ovidsp.ovid.com/ovidweb.cgi?T=JS&PAGE=reference&D=emed7&NEWS=N&AN=137559300) | | Inadequate sample size |
| [Borges, F. N. d. S., & Fischer, F. M. (2003). Twelve-hour night shifts of healthcare workers: a risk to the patients? *Chronobiology International, 20*(2), 351-360. https://doi.org/https://dx.doi.org/10.1081/cbi-120019341](https://doi.org/https:/dx.doi.org/10.1081/cbi-120019341) | | Inadequate sample size |
| Ruggiero, J. S., Redeker, N. S., Fiedler, N., Avi-Itzhak, T., & Fischetti, N. (2011). Sleep and Psychomotor Vigilance in Female Shiftworkers. *Biological Research for Nursing, 14*(3), 225–235. https://doi.org/10.1177/1099800411408413 | | Inadequate sample size |
| Chang, S. J., & Jang, S. J. (2019). Social jetlag and quality of life among nursing students: A cross‐sectional study. *Journal of Advanced Nursing*, *75*(7), 1418–1426. https://doi.org/10.1111/jan.13857 | | Non-nursing sample |
| Güneş, Z., & Arslantaş, H. (2017). Insomnia in nursing students and related factors: A cross-sectional study. *International Journal of Nursing Practice*, *23*(5), e12578. https://doi.org/10.1111/ijn.12578 | | Non-nursing sample |
| Blome, M. J., Johnson, M. L., Jones, M. A., Moore, M. S., & Beck, M. F. (2021). Sleep Quality and Daytime Sleepiness in Prelicensure Baccalaureate Nursing Students. *Journal of Nursing Education*, *60*(4), 196–202. https://doi.org/10.3928/01484834-20210322-03 | | Non-nursing sample |
| Dalai, C., Ghosh, T., Sarkar, D., Sarkar, K., & Ghosal, A. (2021). A study on smartphone addiction and its effects on sleep quality among nursing students in a municipality town of West Bengal. *Journal of Family Medicine and Primary Care*, *10*(1), 378. https://doi.org/10.4103/jfmpc.jfmpc_1657_20 | | Non-nursing sample |
| Fernández-Medina, I. M., Ruíz-Fernández, M. D., Hernández-Padilla, J. M., Granero-Molina, J., Fernández-Sola, C., Jiménez-Lasserrotte, M. del M., Lirola, M.-J., Cortés-Rodríguez, A. E., & López-Rodríguez, M. M. (2020). Adherence to the Mediterranean Diet and Self-efficacy as Mediators in the Mediation of Sleep Quality and Grades in Nursing Students. *Nutrients*, *12*(11), 3265. https://doi.org/10.3390/nu12113265 | | Non-nursing sample |
| Pareek, A. B. (2022). Effect of Sleep Hygiene Program on Quality of Sleep of Nursing Students. A Randomised Controlled Trial. *Neuroquantology, 20*(16). http://doi.org/ 10.48047/NQ.2022.20.16.NQ88165 | | Non-nursing sample |
| Doğru, B. V., Utli, H., & Aykar, F. Ş. (2021). Effect of therapeutic touch on daytime sleepiness, stress and fatigue among students of nursing and midwifery: A randomized sham-controlled trial. *Complementary Therapies in Clinical Practice*, 101322. https://doi.org/10.1016/j.ctcp.2021.101322 | | Non-nursing sample |
| Aktan, G. G., Baysal, E., Eser, I., Sari, D., & Yildirim, G. O. (2020). Sleep Behaviours and Burnout in Nursing Students: A Cross-Sectional Study. *International Journal of Caring Sciences, 13*(2), 1123. | | Non-nursing sample |
| [Ravi, R., & Mohamed, M. G. (2023). Wellbeing among Nursing Students: Relationship between Lifestyle Behaviours, Sleep Quality and Resilience. Africa Jounral of Nursing Midwifery, 24(3), 1219. http://doi.org/10.25159/2520-5293/12619](http://doi.org/10) | | Non-nursing sample |
| Guner, S. G., Ovayolu, O., & Ovayolu, N. (2024). Effect of Ramadan fasting on sleep quality of nursing students in Turkey. *Biological Rhythm Research, 55*(2), 170-183. https://doi.org/10.1080/09291016.2023.2301119 | | Non-nursing sample |
| Rapp, K., Buechele, G., & Weiland, S. K. (2007). Sleep duration and smoking cessation in student nurses. *Addictive Behaviors*, *32*(7), 1505–1510. https://doi.org/10.1016/j.addbeh.2006.11.005 | | Non-nursing sample |
| Park, S., Lee, Y., Yoo, M., & Jung, S. (2019). Wellness and sleep quality in Korean nursing students: A cross-sectional study. *Applied Nursing Research*, *48*, 13–18. https://doi.org/10.1016/j.apnr.2019.05.008 | | Non-nursing sample |
| Jang, S. J., Son, Y., & Lee, H. (2021). Associations among social jetlag, sleep‐related characteristics, premenstrual symptoms, depression, and body mass index of female nursing students. *Perspectives in Psychiatric Care*. https://doi.org/10.1111/ppc.12972 | | Non-nursing sample |
| Salvi, C. P. P., Mendes, S. S., & Martino, M. M. F. D. (2020). Profile of nursing students: quality of life, sleep and eating habits. *Revista Brasileira de Enfermagem*, *73*(suppl 1). https://doi.org/10.1590/0034-7167-2019-0365 | | Non-nursing sample |
| Kayaba, M., Matsushita, T., Katayama, N., Inoue, Y., & Sasai-Sakuma, T. (2021). Sleep–wake rhythm and its association with lifestyle, health-related quality of life and academic performance among Japanese nursing students: a cross-sectional study. *BMC Nursing*, *20*(1). https://doi.org/10.1186/s12912-021-00748-3 | | Non-nursing sample |
| [Gianfredi, V., Nucci, D., Tonzani, A., Amodeo, R., Benvenuti, A. L., Villarini, M., & Moretti, M. (2018). Sleep disorder, Mediterranean Diet and learning performance among nursing students: inSOMNIA, a cross-sectional study. *Annali di igiene : medicina preventiva e di comunita, 30*(6), 470-481. https://doi.org/https://dx.doi.org/10.7416/ai.2018.2258](https://doi.org/https:/dx.doi.org/10.7416/ai.2018.2258) | | Non-nursing sample |
| [Tantalanukul, S., & Wongsawat, P. (2022). Factors associated with stress among nursing students at Boromrajonani colleges of nursing in Thailand northern region under the jurisdiction of ministry of public health. *Trends in Sciences, 19(13), 4641. https://doi.org/10.48048/tis.2022.4641*](https://doi.org/10.48048/tis.2022.4641) | | Non-nursing sample |
| [Marta, O. F., Kuo, S., Bloomfield, J., Lee, H., Ruhyanudin, F., Poynor, M. Y., Brahmadhi, A., Pratiwi, I. D., Aini, N., Mashfufa, E. W., Hasan, F., & Chiu, H. (2020). Gender differences in the relationships between sleep disturbances and academic performance among nursing students: A cross-sectional study. *Nurse Education Today, 85,* 104270. https://doi.org/10.1016/j.nedt.2019.104270](https://doi.org/10.1016/j.nedt.2019.104270) | | Non-nursing sample |
| Huang, C.-Y., Liao, H.-Y., Chang, E.-T., & Lai, H.-L. (2018). Factors associated with the teaching of sleep hygiene to patients in nursing students. *Nurse Education in Practice*, *28*, 150–155. https://doi.org/10.1016/j.nepr.2017.10.029 | | Non-nursing sample |
| Fruh, S. M., Taylor, S. E., Graves, R. J., Hayes, K., McDermott, R., Hauff, C., Williams, S. G., Sittig, S., Campbell, M., Hudson, G., Hall, H., Melnyk, B. M., & Barinas, J. L. (2021). Relationships among hope, body satisfaction, wellness habits, and stress in nursing students. *Journal of Professional Nursing*, *37*(3), 640–647. https://doi.org/10.1016/j.profnurs.2021.01.009 | | Non-nursing sample |
| Şentürk, E., Varol, E., Yıldız, M., & Okyar, G. (2022). Investigation of relationship between melatonin level and religious orientation, depression level and sleep quality of students in nursing and different department. *INTERNATIONAL JOURNAL of ENDOCRINOLOGY (Ukraine)*, *18*(5), 265–272. https://doi.org/10.22141/2224-0721.18.5.2022.1184 | | Non-nursing sample |
| Kotera, Y., Cockerill, V., Chircop, J. G. E., & Forman, D. (2020). Mental health shame, self‐compassion and sleep in UK nursing students: Complete mediation of self‐compassion in sleep and mental health. *Nursing Open*, *8*(3), 1325–1335. https://doi.org/10.1002/nop2.749 | | Non-nursing sample |
| Romero-Blanco, C., Hernández-Martínez, A., Parra-Fernández, M. L., Onieva-Zafra, M. D., Prado-Laguna, M. del C., & Rodríguez-Almagro, J. (2021). Food Addiction and Lifestyle Habits among University Students. *Nutrients*, *13*(4), 1352. https://doi.org/10.3390/nu13041352 | | Non-nursing sample |
| Benavente, S. B. T., Silva, R. M. da, Higashi, A. B., Guido, L. de A., & Costa, A. L. S. (2014). Influence of stress factors and socio-demographic characteristics on the sleep quality of nursing students. *Revista Da Escola de Enfermagem Da USP*, *48*(3), 514–520. https://doi.org/10.1590/s0080-623420140000300018 | | Non-nursing sample |
| Lei, J., Jin, H., Shen, S., Li, Z., & Gu, G. (2014). Influence of clinical practice on nursing students’ mental and immune-endocrine functions. *International Journal of Nursing Practice*, *21*(4), 392–400. https://doi.org/10.1111/ijn.12272 | | Non-nursing sample |
| [Owens, H., Polivka, B., Christian, B., King, K., & Ridner, S. L. (2019). Mediating effect of sleep behaviors when predicting weight-related behaviors in nursing students. *Journal of the American Association of Nurse Practitioners, 31*(5), 309-318. https://doi.org/https://dx.doi.org/10.1097/JXX.0000000000000151](https://doi.org/https:/dx.doi.org/10.1097/JXX.0000000000000151) | | Non-nursing sample |
| Kavurmacı, M., Sarıaslan, A., & Yıldız, İ. (2021). Determination the effects of lavender oil quality of sleep and fatigue of students. *Perspectives in Psychiatric Care*, *58*(3). https://doi.org/10.1111/ppc.12892 | | Non-nursing sample |
| [Rahman, H. A., Hatsanee, A., Menjeni, N. A., Salleh, Z. A., Hamid, R. A., & Ali, M. (2022). Perceived sleep quality: a comparison between hospital nurses and student nurses. *British journal of nursing (Mark Allen Publishing), 31*(11), 578-588. https://doi.org/https://dx.doi.org/10.12968/bjon.2022.31.11.578](https://doi.org/https:/dx.doi.org/10.12968/bjon.2022.31.11.578) | | Non-nursing sample |
| [Abdali, N., Nobahar, M., & Ghorbani, R. (2019). Evaluation of emotional intelligence, sleep quality, and fatigue among Iranian medical, nursing, and paramedical students: A cross-sectional study. *Qatar Medical Journal, 2019*(3), 15. https://doi.org/https://dx.doi.org/10.5339/qmj.2019.15](https://doi.org/https:/dx.doi.org/10.5339/qmj.2019.15) | | Non-nursing sample |
| [Kondo, A., Abuliezi, R., Naruse, K., Oki, T., Niitsu, K., & Ezeonwu, M. C. (2021). Perceived Control, Preventative Health Behaviors, and the Mental Health of Nursing Students During the COVID-19 Pandemic: A Cross-Sectional Study. *Inquiry (United States), 58.* https://doi.org/https://dx.doi.org/10.1177/00469580211060279](https://doi.org/https:/dx.doi.org/10.1177/00469580211060279) | | Non-nursing sample |
| [Ahn, S. Y., & Kim, Y. J. (2015). The influence of smart phone use and stress on quality of sleep among nursing students. *Indian Journal of Science and Technology, 8(*35). https://doi.org/10.17485/ijst/2015/v8i35/IPL0930](https://doi.org/10.17485/ijst/2015/v8i35/IPL0930) | | Non-nursing sample |
| Demir, G. (2017). Daytime sleepiness and related factors in nursing students. *Nurse Education Today*, *59*, 21–25. https://doi.org/10.1016/j.nedt.2017.08.003 | | Non-nursing sample |
| [Kesgin, M. T., & Caglar, S. (2020). Evaluation of sleep quality and perceived stress of nursing students who are engaged in clinical practice based on their sleeping habits. *European Research Journal, 6*(5), 429-437. https://doi.org/https://dx.doi.org/10.18621/EURJ.508165](https://doi.org/https:/dx.doi.org/10.18621/EURJ.508165) | | Non-nursing sample |
| [Zhang, Y., Peters, A., & Chen, G. (2018). Perceived Stress Mediates the Associations between Sleep Quality and Symptoms of Anxiety and Depression among College Nursing Students. *International journal of nursing education scholarship, 15*(1). https://doi.org/https://dx.doi.org/10.1515/ijnes-2017-0020](https://doi.org/https:/dx.doi.org/10.1515/ijnes-2017-0020) | | Non-nursing sample |
| [Lee, C. T., Ting, G. K., Bellissimo, N., & Khalesi, S. (2022). The associations between lifestyle factors and mental well-being in baccalaureate nursing students: An observational study. *Nursing & Health Sciences, 24(*1), 255-264. https://doi.org/https://dx.doi.org/10.1111/nhs.12923](https://doi.org/https:/dx.doi.org/10.1111/nhs.12923) | | Non-nursing sample |
| [Romero-Blanco, C., Rodriguez-Almagro, J., Onieva-Zafra, M. D., Parra-Fernandez, M. L., Prado-Laguna, M. C., & Hernandez-Martinez, A. (2020). Sleep pattern changes in nursing students during the COVID-19 lockdown. *International Journal of Environmental Research and Public Health, 17*(14), 1-11. https://doi.org/https://dx.doi.org/10.3390/ijerph17145222](https://doi.org/https:/dx.doi.org/10.3390/ijerph17145222) | | Non-nursing sample |
| Kayaba, M., Ishitsuka, M., Ishidate, M., Ueno, K., Kajihara, Y., & Sasai-Sakuma, T. (2023). Delayed sleep-wake rhythm due to staying at home during the COVID-19 pandemic and sleep debt after returning to campus among Japanese nursing university students: A longitudinal study. *Heliyon*, *9*(4), e14994. https://doi.org/10.1016/j.heliyon.2023.e14994 | | Non-nursing sample |
| [Mulè, A., Galasso, L., Castelli, L., Ciorciari, A., Michielon, G., Esposito, F., Roveda, E., & Montaruli, A. (2022). Lifestyle of Italian University Students Attending Different Degree Courses: A Survey on Physical Activity, Sleep and Eating Behaviors during the COVID-19 Pandemic. *Sustainability (Switzerland), 14*(22). https://doi.org/10.3390/su142215340](https://doi.org/10.3390/su142215340) | | Non-nursing sample |
| Uppal, M., Gautam, S., Lakra, M. V., Betigeri, A. V., Gandhi, A., & Mondal, S. (2020). Effect of Partial Sleep Deprivation on Neurocognitive Functions in Female Nursing Students: An observational study in India. *International Journal of Current Research and Review*, *12*(14), 49–55. https://doi.org/10.31782/ijcrr.2020.121411 | | Non-nursing sample |
| [Huang, C.-F., Yang, L.-Y., Wu, L.-M., Liu, Y., & Chen, H.-M. (2014). Determinants of daytime sleepiness in first-year nursing students: a questionnaire survey. *Nurse education today, 34*(6), 1048-1053. https://doi.org/https://dx.doi.org/10.1016/j.nedt.2013.11.005](https://doi.org/https:/dx.doi.org/10.1016/j.nedt.2013.11.005) | | Non-nursing sample |
| [Yilmaz, D., Tanrikulu, F., & Dikmen, Y. (2017). Research on Sleep Quality and the Factors Affecting the Sleep Quality of the Nursing Students. *Current health sciences journal, 43*(1), 20-24. https://doi.org/https://dx.doi.org/10.12865/CHSJ.43.01.03](https://doi.org/https:/dx.doi.org/10.12865/CHSJ.43.01.03) | | Non-nursing sample |
| Amaral, K. V., Galdino, M. J. Q., & Martins, J. T. (2021). Sleep quality and work among nursing vocational students. *Revista Brasileira de Enfermagem*, *74*(6). https://doi.org/10.1590/0034-7167-2020-1285 | | Non-nursing sample |
| Mendes, S. S., & Martino, M. M. F. D. (2020). Stress factors of nursing students in their final year. *Revista Da Escola de Enfermagem Da USP*, *54*. https://doi.org/10.1590/S1980-220X2018053903593 | | Non-nursing sample |
| Sahu, M., Gandhi, S., Kumar Sharma, M., & Marimuthu, P. (2020). Social media use and health promoting lifestyle: an exploration among Indian nursing students. *Investigación Y Educación En Enfermería*, *38*(2). https://doi.org/10.17533/udea.iee.v38n2e12 | | Non-nursing sample |
| [Mikaniki, T., Assarroudi, A., Shegarf Nakhaie, M. R., Akrami, R., & Sahebkar, M. (2020). Sleep quality and correlated factors among retired nurses in the North-east of Iran. *Africa Journal of Nursing and Midwifery, 22*(2), 8032. https://doi.org/https://dx.doi.org/10.25159/2520-5293/8032](https://doi.org/https:/dx.doi.org/10.25159/2520-5293/8032) | | Non-nursing sample |
| [Mollaoǧlu, M., Şahin, S., Tuncay, F. Ö., & Fertelli, T. K. (2010). An analysis of relation between sleep quality and academic success. *SENDROM, 22*(1), 52-56. https://www.scopus.com/inward/record.uri?eid=2-s2.0-77950999834&partnerID=40&md5=6049e62f532578e74d8b58d4a9d54584](https://www.scopus.com/inward/record.uri?eid=2-s2.0-77950999834&partnerID=40&md5=6049e62f532578e74d8b58d4a9d54584) | | Non-nursing sample |
| [Pileggi, H., Nugent, R., Buscemi, D., Nugent, K., & Raj, R. (2011). Characterizing the quantity and perceived quality of sleep in a health care student population. *Journal of Investigative Medicine, 59*(2), 491. https://doi.org/https://dx.doi.org/10.231/JIM.0b013e31820bab4c](https://doi.org/https:/dx.doi.org/10.231/JIM.0b013e31820bab4c) | | Non-nursing sample |
| [Chust-Hernández, P., & Toledano-Enríquez, L. (2021). Impact of self-esteem, trait anxiety and quality of sleep on the satisfaction with life in Nursing students. *Metas de Enfermeria, 24*(7), 26-32. https://doi.org/10.35667/MetasEnf.0000.24.1003081794](https://doi.org/10.35667/MetasEnf.0000.24.1003081794) | | Non-nursing sample |
| [Huang, Y. W., Yang, C. H., Ho, S. L., & Hwu, Y. J. (2011). The relationship between practicum-related stress and sleep quality in nursing students. *Journal of Nursing and Healthcare Research, 7*(1), 14-25. https://www.scopus.com/inward/record.uri?eid=2-s2.0-84865120193&partnerID=40&md5=b6599b7edff10e5e83c751d5cb6b0456](https://www.scopus.com/inward/record.uri?eid=2-s2.0-84865120193&partnerID=40&md5=b6599b7edff10e5e83c751d5cb6b0456) | | Non-nursing sample |
| [Pengbo, Y., Dianli, H., Wenjing, S., Ying, W., Xuequn, Z., Ying, C., Guimei, D., Xiaolin, G., Yaning, L., Yuehao, S., Kun, S., Chengfei, X., Peiran, Y., Sai, L., & Xuelian, P. (2021). Investigation on sleep quality and mental health of nurses in public health emergencies. *Chinese Journal of Practical Nursing, 37(*27), 2111-2116. https://doi.org/10.3760/cma.j.cn211501-20200823-03636](https://doi.org/10.3760/cma.j.cn211501-20200823-03636) | | Not written in English |
| [Aghdam, N. F., Ameri, M., Goli, S., & Imeni, M. (2020). Relationship Between Sleep Quality and Job Stress of Nurses in Different Shifts Working. *Avicenna Journal of Nursing and Midwifery Care, 28*(2), 103-111. https://doi.org/10.30699/ajnmc.28.2.103](https://doi.org/10.30699/ajnmc.28.2.103) | | Not written in English |
| [Yang, Y., Zhao, R. Y., & Lan, M. J. (2008). [Effect of sleep quality on day cycle fatigue in ward nurses]. *Zhonghua lao dong wei sheng zhi ye bing za zhi = Zhonghua laodong weisheng zhiyebing zazhi = Chinese journal of industrial hygiene and occupational diseases, 26*(6), 360-362. https://www.scopus.com/inward/record.uri?eid=2-s2.0-84879791498&partnerID=40&md5=98deea7f615c10a779e193bbe07a2dbc](https://www.scopus.com/inward/record.uri?eid=2-s2.0-84879791498&partnerID=40&md5=98deea7f615c10a779e193bbe07a2dbc) | | Not written in English |
| [Silveira, F. B. C. A., Neto, J. C. G. L., Weiss, C., & de Araujo, M. F. M. (2021). Association between community-based and workplace violence and the sleep quality of health professionals: A cross-sectional study. *Ciencia e Saude Coletiva, 26*(5), 1647-1656. https://doi.org/https://dx.doi.org/10.1590/1413-81232021265.04522021](https://doi.org/https:/dx.doi.org/10.1590/1413-81232021265.04522021) | | Not written in English |
| [Niu, Y., Tao, S., Yang, Y., Zou, L., Li, T., Xie, Y., Zhang, D., Zhai, S., Qu, Y., Tao, F., & Wu, X. (2023). Association between sleep quality and anxiety-depression co-morbid symptoms among nursing students of medical college in Hefei City. *Chinese Journal of School Health, 44*(8), 1186-1189. https://doi.org/10.16835/j.cnki.1000-9817.2023.08.015](https://doi.org/10.16835/j.cnki.1000-9817.2023.08.015) | | Not written in English |
| [Del Angel Perez, B., Perez, R. L., Perez, P. A., De Los Angeles Fang Huerta, M., & Barrios, F. F. (2023). Association between sleep quality and quantity and body mass index in college adolescents: A cross-sectional study. *Revista Cuidarte, 14*(3). https://doi.org/10.15649/cuidarte.3032](https://doi.org/10.15649/cuidarte.3032) | | Not written in English |
| [Wang, L., Xia, C. L., & Fan, L. (2020). Associations between sleep and non-alcoholic fatty liver disease in nurses. *Journal of Environmental and Occupational Medicine, 37*(5), 497-502. https://doi.org/10.13213/j.cnki.jeom.2020.19711](https://doi.org/10.13213/j.cnki.jeom.2020.19711) | | Not written in English |
| [Kholmogorova, A. B., Petrikov, S. S., Suroyegina, A. Y., Mikita, O. Y., Rakhmanina, A. A., & Roy, A. P. (2020). Burnout and its factors in healthcare workers involved in providing health care for patients with COVID-19 at different stages of the pandemic. *Russian Sklifosovsky Journal of Emergency Medical Care, 9*(3), 321-337. https://doi.org/10.23934/2223-9022-2020-9-3-321-337](https://doi.org/10.23934/2223-9022-2020-9-3-321-337) | | Not written in English |
| [Figueiredo de Martino, M. M. (2002). Comparative study of sleep patterns in nurses working day and night shifts. *Revista Panamericana de Salud Publica/Pan American Journal of Public Health, 12*(2), 95-100. https://doi.org/10.1590/s1020-49892002000800004](https://doi.org/10.1590/s1020-49892002000800004) | | Not written in English |
| [Wu, J. H., Wang, X. D., Huang, Y. L., & Jian, D. M. (2005). Comparison of quality of life and mental health of nurses in large and medium-sized hospitals. *Chinese Journal of Clinical Rehabilitation, 9*(32), 85-87. https://www.scopus.com/inward/record.uri?eid=2-s2.0-27644478774&partnerID=40&md5=7aa6084febf14b244dadee20c5114554](https://www.scopus.com/inward/record.uri?eid=2-s2.0-27644478774&partnerID=40&md5=7aa6084febf14b244dadee20c5114554) | | Not written in English |
| [Salaree, M. M., Rahmati, F., & Parandeh, A. (2021). Comparison of sleep quality and its related factors in nurses before and at the same time with the COVID-19 epidemic. *Journal of Military Medicine, 23*(5), 395-403. https://doi.org/10.30491/JMM.23.5.395](https://doi.org/10.30491/JMM.23.5.395) | | Not written in English |
| [Ghods, A. A., Emadi Khalaf, M., Mirmohamadkhani, M., & Sotodehasl, N. (2016). Comparison of the effects of lavender essential oil and sesame oil on sleep quality of nurses. *Journal of Babol University of Medical Sciences, 18*(5), 13-19. https://www.scopus.com/inward/record.uri?eid=2-s2.0-84976493254&partnerID=40&md5=cbb0cfe81e18d4b3d652e60ffdf9eb1a](https://www.scopus.com/inward/record.uri?eid=2-s2.0-84976493254&partnerID=40&md5=cbb0cfe81e18d4b3d652e60ffdf9eb1a) | | Not written in English |
| [Kouhnavard, B., Behbahani, N. R., & Soleimani, N. (2017). Comparisons between quality of sleep and chronic fatigue syndrome in hospital radiology employees and nurses. *Iran Occupational Health, 14*(3), 26-36. https://www.scopus.com/inward/record.uri?eid=2-s2.0-85036664998&partnerID=40&md5=2d4cac37cbf3db3d649ea35c44eb8aba](https://www.scopus.com/inward/record.uri?eid=2-s2.0-85036664998&partnerID=40&md5=2d4cac37cbf3db3d649ea35c44eb8aba) | | Not written in English |
| [Liu, Q., Jiang, L., Ye, J., Ge, X., Yang, N., Yan, H., Wuyang, H., & Han, X. (2021). Correlation between shift work and sleep disorders of medical staff in tertiary hospitals. J*ournal of Environmental and Occupational Medicine, 38*(1), 76-82. https://doi.org/10.13213/j.cnki.jeom.2021.20313](https://doi.org/10.13213/j.cnki.jeom.2021.20313) | | Not written in English |
| [Chang, H., Xie, M., Chen, J., Tao, M., Zhang, Q., Liu, R., & Li, X. (2023). Current situation and potential profile analysis of self-compassion behavior of clinical nurses. *Chinese Journal of Nursing, 58*(13), 1615-1622. https://doi.org/10.3761/j.issn.0254-1769.2023.13.013](https://doi.org/10.3761/j.issn.0254-1769.2023.13.013) | | Not written in English |
| [Jung, Y. J., & Kang, S. W. (2017). Differences in sleep, fatigue, and neurocognitive function between shift nurses and non-shift nurses. *Korean Journal of Adult Nursing, 29*(2), 190-199. https://doi.org/10.7475/kjan.2017.29.2.190](https://doi.org/10.7475/kjan.2017.29.2.190) | | Not written in English |
| [Kim, H. J. (2012). Effect of environmental intervention on sleep, emotions and job satisfaction of rotating shift nurses in intensive care unit. *Korean Journal of Adult Nursing, 24*(1), 11-19. https://doi.org/10.7475/kjan.2012.24.1.11](https://doi.org/10.7475/kjan.2012.24.1.11) | | Not written in English |
| [Fei, Y. (2019). Effects of emotional resilience training on nurses' perceived stress, positive and negative emotions and sleep quality. *Revista Argentina de Clinica Psicologica, 28*(2), 199-209. https://doi.org/10.24205/03276716.2019.1106](https://doi.org/10.24205/03276716.2019.1106) | | Not written in English |
| [Seol, M. J., Lee, B. S., & Lee, S. K. (2018). Effects of labor intensity and fatigue on sleep quality of clinical nurses. *Journal of Korean Academy of Nursing Administration, 24*(4), 276-287. https://doi.org/10.11111/jkana.2018.24.4.276](https://doi.org/10.11111/jkana.2018.24.4.276) | | Not written in English |
| [Venuta, M., Barzaghi, L., Cavalieri, C., Gamberoni, T., & Guaraldi, G. P. (1999). Effects of shift work on the quality of sleep and psychological health based on a sample of professional nurses. *Giornale italiano di medicina del lavoro ed ergonomia, 21*(3), 221-225. https://www.scopus.com/inward/record.uri?eid=2-s2.0-0033156038&partnerID=40&md5=88e91a9968f077b017bebfd274d15d4e](https://www.scopus.com/inward/record.uri?eid=2-s2.0-0033156038&partnerID=40&md5=88e91a9968f077b017bebfd274d15d4e) | | Not written in English |
| [Chashemi, N. A., Mirrezaie, S. M., Nouhi, S., & Khastar, H. (2018). Evaluating the relationship between sleep disturbances and sexual quality of life among female shift working nurses. *Journal of Knowledge and Health in Basic Medical Sciences, 13*(1), 19-25. https://doi.org/10.22100/jkh.v13i1.1869](https://doi.org/10.22100/jkh.v13i1.1869) | | Not written in English |
| [Shirali, G. A., Afshari, D., & Karimpour, S. (2021). Evaluation of Human Errors among Nurses Using Predictive Analysis of Cognitive Errors and Human Event Analysis Techniques: Case study of the Specific Responsibilities of the Cardiac Intensive Care Unit. *Journal of Health and Safety at Work, 11*(2), 213-225. https://www.scopus.com/inward/record.uri?eid=2-s2.0-85123249928&partnerID=40&md5=0c00c90ada86f59adcec9de064dfc864](https://www.scopus.com/inward/record.uri?eid=2-s2.0-85123249928&partnerID=40&md5=0c00c90ada86f59adcec9de064dfc864) | | Not written in English |
| [Pavlova, A. N., Mishkich, I. A., Luchkevich, V. S., Chechura, A. N., & Zarudnaya, V. V. (2017). Evaluation of professional readiness of health care providers to prevention-oriented activities. *Gigiena i Sanitariya, 96*(4), 402-404. https://doi.org/https://dx.doi.org/10.18821/0016-9900-2017-96-4-402-404](https://doi.org/https:/dx.doi.org/10.18821/0016-9900-2017-96-4-402-404) | | Not written in English |
| [Tsai, P. C., Lin, J. H., & Hsu, H. C. (2019). Exploring sleep quality, spiritual health, and related factors in nurses in the intensive care unit. *Journal of Nursing, 66*(4), 49-59. https://doi.org/10.6224/JN.201908_66(4).07](https://doi.org/10.6224/JN.201908_66(4).07) | | Not written in English |
| [An, M., Kim, J., Heo, S., Kim, S. H., & Hwang, Y. Y. (2018). Factors affecting sleep quality of clinical nurses working in a hospital. *Korean Journal of Adult Nursing, 30*(2), 126-135. https://doi.org/10.7475/kjan.2018.30.2.126](https://doi.org/10.7475/kjan.2018.30.2.126) | | Not written in English |
| [Cândido Morais, H. C., Gomes, B. G., da Silva de Holanda, A. K., Alves Nogueira, K. E., Fonseca, R., Dinelly Pinto, A. C. M., & Mendes, I. C. (2022). Factors associated with poor sleep quality in nursing students from a private university in Ceará. *Mundo da Saude, 46,* 3-11. https://doi.org/10.15343/0104-7809.202246003011](https://doi.org/10.15343/0104-7809.202246003011) | | Not written in English |
| [Kubo, T., Takahashi, M., Sallinen, M., Kubo, Y., & Suzumura, H. (2013). [How are leisure activity and shiftwork schedule associated with recovery from fatigue in shiftwork nurses?]. *Sangyō eiseigaku zasshi = Journal of occupational health, 55*(3), 90-102. https://doi.org/10.1539/sangyoeisei.B12004](https://doi.org/10.1539/sangyoeisei.B12004) | | Not written in English |
| [Fischer, F. M., Teixeira, L. R., Borges, F. N., Goncalves, M. B., & Ferreira, R. M. (2002). How nursing staff perceive the duration and quality of sleep and levels of alertness. *Cadernos de saúde pública / Ministério da Saúde, Fundação Oswaldo Cruz, Escola Nacional de Saúde Pública, 18*(5), 1261-1269. https://doi.org/10.1590/s0102-311x2002000500018](https://doi.org/10.1590/s0102-311x2002000500018) | | Not written in English |
| [Maier, M. D. R., & Kanunfre, C. C. (2021). Impact on nursing personnel's mental health and sleep quality during the COVID-19 pandemic. *Revista Enfermagem, 29,* e61806. https://doi.org/https://dx.doi.org/10.12957/reuerj.2021.61806](https://doi.org/https:/dx.doi.org/10.12957/reuerj.2021.61806) | | Not written in English |
| [Guo, W., & Wang, W. (2019). Investigation on sleep quality of nurses in emergency departments of tertiary hospitals and analysis of influencing factors. *Chinese Journal of Industrial Hygiene and Occupational Diseases, 37*(4), 286-289. https://doi.org/10.3760/cma.j.issn.1001-9391.2019.04.011](https://doi.org/10.3760/cma.j.issn.1001-9391.2019.04.011) | | Not written in English |
| [Mauro, M. Y. C., Rebelo, A. M. S., Ferreira, A. O. M., Sper, N. P. T., Santos, M. I. S., & Gallasch, C. H. (2019). Night work and self-perceived body weight changes among nursing professionals. *Revista Enfermagem, 27,* e31273. https://doi.org/https://dx.doi.org/10.12957/reuerj.2019.31273](https://doi.org/https:/dx.doi.org/10.12957/reuerj.2019.31273) | | Not written in English |
| [Moreno-Casbas, M. T., Alonso-Poncelas, E., Gomez-Garcia, T., Martinez-Madrid, M. J., & Escobar-Aguilar, G. (2018). Perception of the quality of care, work environment and sleep characteristics of nurses working in the National Health System. *Enfermeria Clinica, 28*(4), 230-239. https://doi.org/https://dx.doi.org/10.1016/j.enfcli.2018.01.001](https://doi.org/https:/dx.doi.org/10.1016/j.enfcli.2018.01.001) | | Not written in English |
| [Pu, S. J., Wang, C. Q., Wu, H., Shen, L., & Qiu, J. N. (2022). Physical and mental condition and medicine consumption of frontline medical staff of shelter hospitals in Shanghai during the fight against the epidemic of coronavirus disease 2019. *Academic Journal of Naval Medical University, 43*(11), 1268-1273. https://doi.org/10.16781/j.CN31-2187/R.20220461](https://doi.org/10.16781/j.CN31-2187/R.20220461) | | Not written in English |
| [Sheng, X., Liu, F., Zhou, J., & Liao, R. (2020). Psychological status and sleep quality of nursing interns during the outbreak of COVID-19. *Nan Fang Yi Ke Da Xue Xue Bao / Journal of Southern Medical University, 40*(3), 346-350. https://doi.org/10.12122/j.issn.1673-4254.2020.03.07](https://doi.org/10.12122/j.issn.1673-4254.2020.03.07) | | Not written in English |
| [Scatularo, C. E., Battioni, L., Bellia, S., De Robert, S. C., Gatti, M. D. L. N., Racki, M., Soracio, G., Lescano, A., Giorgini, J., & Pereiro, S. M. (2021). Psychophysical impact of the covid-19 pandemic on healthcare workers in argentina. The imppacts-sac.20 survey. *Revista Argentina de Cardiologia, 89*(3), 196-202. https://doi.org/https://dx.doi.org/10.7775/rac.v89.i3.20231](https://doi.org/https:/dx.doi.org/10.7775/rac.v89.i3.20231) | | Not written in English |
| [Baek, J., & Choi-Kwon, S. (2020). Secondary data analysis on the factors influencing premenstrual symptoms of shift work nurses: Focused on the sleep and occupational stress. *Journal of Korean Academy of Nursing, 50*(4), 631-640. https://doi.org/https://dx.doi.org/10.4040/jkan.19230](https://doi.org/https:/dx.doi.org/10.4040/jkan.19230) | | Not written in English |
| [Mendes, S. S., & De Martino, M. M. F. (2012). Shift work: Overall health state related to sleep in nursing workers. *Revista da Escola de Enfermagem, 46*(6), 1471-1476. https://doi.org/https://dx.doi.org/10.1590/S0080-62342012000600026](https://doi.org/https:/dx.doi.org/10.1590/S0080-62342012000600026) | | Not written in English |
| [Zambrano, C. L. M., Bello, P. T. M., Soto, C. F. O., Cárdenas, C. P. P., & Rivera, L. C. V. (2023). SLEEP IN NURSING STUDENTS AT A UNIVERSITY IN SOUTHERN CHILE. *Horizonte de Enfermeria, 34*(3), 610-623. https://doi.org/10.7764/Horiz_Enferm.34.3.610-623](https://doi.org/10.7764/Horiz_Enferm.34.3.610-623) | | Not written in English |
| [Kim, H. S., Eom, M. R., & Kim, E. K. (2010). Sleep patterns and circadian types of nursing students during shift schedules. *Journal of Korean Academy of Nursing, 40*(1), 43-51. https://doi.org/10.4040/jkan.2010.40.1.43](https://doi.org/10.4040/jkan.2010.40.1.43) | | Not written in English |
| [Ferreira, L. R. C., & De Martino, M. M. F. (2012). Sleep patterns and fatigue of nursing students who work. *Revista da Escola de Enfermagem, 46*(5), 1178-1183. https://doi.org/https://dx.doi.org/10.1590/S0080-62342012000500020](https://doi.org/https:/dx.doi.org/10.1590/S0080-62342012000500020) | | Not written in English |
| [Nunes de Araújo, M. A., Pereira Fabi, F. W., Bortolon, R., Lunardi Filho, W. D., Ilha, S., & Martins Alvarenga, M. R. (2016). Sleep patterns in nursing students at Public State University. *Revista Cubana de Enfermeria, 32(*2), 162-170. https://www.scopus.com/inward/record.uri?eid=2-s2.0-84994230242&partnerID=40&md5=d37f4f63e46ae0f9a9a13204f852f156](https://www.scopus.com/inward/record.uri?eid=2-s2.0-84994230242&partnerID=40&md5=d37f4f63e46ae0f9a9a13204f852f156) | | Not written in English |
| [Furlani, R., & Ceolim, M. F. (2005). Sleep patterns of first-year nursing students. *Revista Brasileira de Enfermagem, 58*(3), 320-324. https://doi.org/10.1590/s0034-71672005000300013](https://doi.org/10.1590/s0034-71672005000300013) | | Not written in English |
| [Tribis-Arrospe, B., Ballesteros-Peña, S., Inchaurregui, L. C. A., Egea-Santaolalla, C., Guerra-Martin, L., & Álvarez Ruiz de Larrinaga, A. (2020). Sleep quality and adaptation to shift-work among ambulance staff in the basque country. *Anales del Sistema Sanitario de Navarra, 43*(2), 189-202. https://doi.org/10.23938/ASSN.0871](https://doi.org/10.23938/ASSN.0871) | | Not written in English |
| [Dos Santos, A. F., Mussi, F. C., da Silva Pires, C. G., de Souza Teles Santos, C. A., & Santos Paim, M. A. (2020). Sleep quality and associated factors in nursing undergraduates*. ACTA Paulista de Enfermagem, 33.* https://doi.org/10.37689/ACTA-APE/2020AO0144](https://doi.org/10.37689/ACTA-APE/2020AO0144) | | Not written in English |
| [Ahmadabadi, A., Tavousi, S. H., Sedaghat, A., Antighechi, F. S., & Rezaiyan, M. K. (2018). Sleep quality in nurses of burn ward compared to other clinical wards. *Iran Occupational Health, 15(*1), 26-33. https://www.scopus.com/inward/record.uri?eid=2-s2.0-85050275323&partnerID=40&md5=ed5e225fdc9a490d12aaf790d982ef9c](https://www.scopus.com/inward/record.uri?eid=2-s2.0-85050275323&partnerID=40&md5=ed5e225fdc9a490d12aaf790d982ef9c) | | Not written in English |
| [Satizábal Moreno, J. P., & Marín Ariza, D. A. (2018). Sleep Quality of Nurses Staff. *Revista Ciencias de la Salud, 16*, 75-86. https://doi.org/10.12804/revistas.urosario.edu.co/revsalud/a.6846](https://doi.org/10.12804/revistas.urosario.edu.co/revsalud/a.6846) | | Not written in English |
| [Renz Pretto, C., da Silva, R. M., Dorneles Lenz, F. C., Ester Müller, F., Flores Jacobi, L., & Nunes Frizzo, M. (2022). Sleep, Stress, Cardiovascular Risk and Ancillary Therapy in Nursing Professional from the Emergency Departmen*t. Revista Cubana de Enfermeria, 38*(3). https://www.scopus.com/inward/record.uri?eid=2-s2.0-85138305238&partnerID=40&md5=f20ab63e14f538c10df8da0dc63bc33e](https://www.scopus.com/inward/record.uri?eid=2-s2.0-85138305238&partnerID=40&md5=f20ab63e14f538c10df8da0dc63bc33e) | | Not written in English |
| [Da Rocha, M. C. P., & De Martino, M. M. F. (2009). Stress and sleep quality among registered nurses who use sleeping pills. *ACTA Paulista de Enfermagem, 22*(5), 658-665. https://doi.org/https://dx.doi.org/10.1590/s0103-21002009000500010](https://doi.org/https:/dx.doi.org/10.1590/s0103-21002009000500010) | | Not written in English |
| [Da Rocha, M. C. P., & de Martino, M. M. F. (2010). Stress and sleep quality of nurses working different hospital shifts. *Revista da Escola de Enfermagem, 44*(2), 280-286. https://doi.org/10.1590/S0080-62342010000200006](https://doi.org/10.1590/S0080-62342010000200006) | | Not written in English |
| [Jeong, J. Y., & Gu, M. O. (2018). Structural equation model for sleep quality of female shift work nurses. *Journal of Korean Academy of Nursing, 48*(5), 622-635. https://doi.org/10.4040/jkan.2018.48.5.622](https://doi.org/10.4040/jkan.2018.48.5.622) | | Not written in English |
| [Xu, L., Li, Y., & Fa, T. (2021). Study on the effect of circadian rhythm and sleep quality on job burnout among shift nurses in 3A-level hospitals. *Chinese Journal of Practical Nursing, 37*(1), 55-61. https://doi.org/10.3760/cma.j.cn211501-20200408-01721](https://doi.org/10.3760/cma.j.cn211501-20200408-01721) | | Not written in English |
| [De Martino, M. M. F. (2009). The architecture of day sleeping and the sleep-wake cycle in nurses in their working shifts. *Revista da Escola de Enfermagem, 43*(1), 186-191. https://doi.org/https://dx.doi.org/10.1590/S0080-62342009000100025](https://doi.org/https:/dx.doi.org/10.1590/S0080-62342009000100025) | | Not written in English |
| [Hosseini, S. K., Sayadi, A., Lotfabad, M. M., & Heidari, S. (2020). The effect of benson’s relaxation technique on sleep quality among shift-working nurses in hospitals. *HAYAT, 25*(4), 415-428. https://www.scopus.com/inward/record.uri?eid=2-s2.0-85081629943&partnerID=40&md5=bba39dd800cd80e073c9f352528b90d1](https://www.scopus.com/inward/record.uri?eid=2-s2.0-85081629943&partnerID=40&md5=bba39dd800cd80e073c9f352528b90d1) | | Not written in English |
| [Yang, S. Y., Wu, T. H., Wang, M. C., Chen, F. T., & Pan, Y. J. (2015). The effectiveness of lavender-based Aromatherapy on the sleep quality of female nurses. *Journal of Nursing and Healthcare Research, 11*(2), 99-108. https://doi.org/10.6225/JNHR.11.2.99](https://doi.org/10.6225/JNHR.11.2.99) | | Not written in English |
| [Kim, J. E., & Kim, S. S. (2017). The effects of mobile social networking service-based cognitive behavior therapy on insomnia in nurses. *Journal of Korean Academy of Nursing, 47*(4), 476-487. https://doi.org/10.4040/jkan.2017.47.4.476](https://doi.org/10.4040/jkan.2017.47.4.476) | | Not written in English |
| [Huang, K. H., Yin, H. F., Tang, Y. R., Liu, Y. X., Song, A. T., Zhang, Y. Y., & Zhang, Y. Q. (2022). The occupational fatigue among nurses with master degree. *Chinese Journal of Nursing Education, 19(*3), 267-272. https://doi.org/10.3761/j.issn.1672-9234.2022.03.014](https://doi.org/10.3761/j.issn.1672-9234.2022.03.014) | | Not written in English |
| [Kikuchi, Y., & Ishii, N. (2015). The relationship between self-awareness of fatigue symptoms and working conditions in female nurses. *Sangyō eiseigaku zasshi = Journal of occupational health, 57(*5), 230-240. https://doi.org/10.1539/sangyoeisei.E14005](https://doi.org/10.1539/sangyoeisei.E14005) | | Not written in English |
| [Bahri, N., Shamshri, M., Moshki, M., & Mogharab, M. (2014). The survey of sleep quality and its relationship to mental health of hospital nurses. *Iran Occupational Health, 11*(3), 96-104. https://www.scopus.com/inward/record.uri?eid=2-s2.0-84910636435&partnerID=40&md5=a3a8d901fbfb14f666fee41c80051091](https://www.scopus.com/inward/record.uri?eid=2-s2.0-84910636435&partnerID=40&md5=a3a8d901fbfb14f666fee41c80051091) | | Not written in English |
| [Shin, S., Lee, I., Kim, J., & Bae, S. H. (2019). Work-related characteristics and sleep quality of nurses in comprehensive nursing care units of small-medium sized hospitals. *Journal of the Korean Academy of Fundamentals of Nursing, 26*(4), 260-268. https://doi.org/10.7739/jkafn.2019.26.4.260](https://doi.org/10.7739/jkafn.2019.26.4.260) | | Not written in English |
| [Loew, M., Niel, K., Burlison, J. D., Russell, K. M., Karol, S. E., Talleur, A. C., Christy, L. A., Johnson, L.-M., & Crabtree, V. M. (2019). A quality improvement project to improve pediatric medical provider sleep and communication during night shifts. *International Journal for Quality in Health Care, 31*(8), 633-638. https://doi.org/https://dx.doi.org/10.1093/intqhc/mzy221](https://doi.org/https:/dx.doi.org/10.1093/intqhc/mzy221) | | Nurses not reported separately |
| [Korsiak, J., Tranmer, J., Leung, M., Borghese, M. M., & Aronson, K. J. (2018). Actigraph measures of sleep among female hospital employees working day or alternating day and night shifts. *Journal of Sleep Research, 27*(4), 1-8. https://doi.org/https://dx.doi.org/10.1111/jsr.12579](https://doi.org/https:/dx.doi.org/10.1111/jsr.12579) | | Nurses not reported separately |
| [Wyse, C. A., Celis Morales, C. A., Graham, N., Fan, Y., Ward, J., Curtis, A. M., Mackay, D., Smith, D. J., Bailey, M. E. S., Biello, S., Gill, J. M. R., & Pell, J. P. (2017). Adverse metabolic and mental health outcomes associated with shiftwork in a population-based study of 277,168 workers in UK biobank*. *Annals of Medicine, 49(*5), 411-420. https://doi.org/https://dx.doi.org/10.1080/07853890.2017.1292045](https://doi.org/https:/dx.doi.org/10.1080/07853890.2017.1292045) | | Nurses not reported separately |
| [Wasik, J., & Koweszko, T. (2022). Analysis of mental health and sleep problems among nursing and midwifery staff during the COVID-19 pandemic. *Psychiatria, 19*(2), 79-88. https://doi.org/10.5603/PSYCH.a2021.0046](https://doi.org/10.5603/PSYCH.a2021.0046) | | Nurses not reported separately |
| [Paudel, L., Sharma, P., Kadel, A. R., Lakhey, K., Singh, S., Khanal, P., Sharma, R., Chalise, P., Sharma, S. C., & Pradhan, S. N. (2021). Association Between Internet Addiction, Depression and Sleep Quality Among Undergraduate Students of Medical and Allied Sciences. *Journal of Nepal Health Research Council, 19*(3), 543-549. https://doi.org/https://dx.doi.org/10.33314/jnhrc.v19i3.3599](https://doi.org/https:/dx.doi.org/10.33314/jnhrc.v19i3.3599) | | Nurses not reported separately |
| [Alhusami, M., Jatan, N., Dsouza, S., & Sultan, M. A. (2024). Association between physical activity and sleep quality among healthcare students. *Frontiers in Sports and Active Living, 6.* https://doi.org/10.3389/fspor.2024.1357043](https://doi.org/10.3389/fspor.2024.1357043) | | Nurses not reported separately |
| [Palhares, V. C., Corrente, J. E., & Matsubara, B. B. (2014). Association between sleep quality and quality of life in nursing professionals working rotating shifts. *Revista de Saude Publica, 48*(4), 594-601. https://doi.org/https://dx.doi.org/10.1590/S0034-8910.2014048004939](https://doi.org/https:/dx.doi.org/10.1590/S0034-8910.2014048004939) | | Nurses not reported separately |
| [Lwiza, A. F., & Lugazia, E. R. (2023). Burnout and associated factors among healthcare workers in acute care settings at a tertiary teaching hospital in Tanzania: An analytical cross-sectional study. *Health Science Reports, 6*(5), e1256. https://doi.org/https://dx.doi.org/10.1002/hsr2.1256](https://doi.org/https:/dx.doi.org/10.1002/hsr2.1256) | | Nurses not reported separately |
| [Yella, T., & Dmello, M. K. (2022). Burnout and sleep quality among community health workers during the pandemic in selected city of Andhra Pradesh. *Clinical Epidemiology and Global Health, 16,* 101109. https://doi.org/https://dx.doi.org/10.1016/j.cegh.2022.101109](https://doi.org/https:/dx.doi.org/10.1016/j.cegh.2022.101109) | | Nurses not reported separately |
| [Lele, E. C. B., Ndongo, J. M., Ashu-Akoh, A. V., Ahmadou, Guyot, J., Ngalagou, P. T. M., Bongue, B., Tendongfor, N., Ayina, C. N. A., Tanga, M. Y. L., Mandengue, S. H., & Ndemba, P. B. A. (2023). Burnout syndrome among healthcare professionals in the Fako division, Cameroon: Impact of physical activity and sleep quality. *AIMS public health, 10*(4), 814-827. https://doi.org/https://dx.doi.org/10.3934/publichealth.2023054](https://doi.org/https:/dx.doi.org/10.3934/publichealth.2023054) | | Nurses not reported separately |
| [Guruswamy, A., Swamy, S., & Kurpad, K. P. (2022). Clinical Profile of Tension Type Headache in a Medical College with Special Emphasis on Triggering Factors. *Neurology India, 70*(5), 1958-1962. https://doi.org/https://dx.doi.org/10.4103/0028-3886.359261](https://doi.org/https:/dx.doi.org/10.4103/0028-3886.359261) | | Nurses not reported separately |
| [Kinoshita, T., Maruyama, K., Suyama, K., Nishijima, M., Akamatsu, K., Jogamoto, A., Katakami, K., & Saito, I. (2021). Consumption of OLL1073R-1 yogurt improves psychological quality of life in women healthcare workers: secondary analysis of a randomized controlled trial. BMC Gastroenterology, 21(1), 237. https://doi.org/https://dx.doi.org/10.1186/s12876-021-01793-7](https://doi.org/https:/dx.doi.org/10.1186/s12876-021-01793-7) | | Nurses not reported separately |
| [Wang, Q., Chu, H., Li, H., Li, C., Li, S., Fang, H., Liang, D., Deng, T., Li, J., & Liu, A. (2023). Deep neural network for prediction of diet quality among doctors and nurses in North China during the COVID-19 pandemic. *Frontiers in Public Health, 11,* 1196090. https://doi.org/https://dx.doi.org/10.3389/fpubh.2023.1196090](https://doi.org/https:/dx.doi.org/10.3389/fpubh.2023.1196090) | | Nurses not reported separately |
| [Arafa, A., Mohammed, Z., Mahmoud, O., Elshazley, M., & Ewis, A. (2021). Depressed, anxious, and stressed: What have healthcare workers on the frontlines in Egypt and Saudi Arabia experienced during the COVID-19 pandemic? *Journal of Affective Disorders, 278,* 365-371. https://doi.org/https://dx.doi.org/10.1016/j.jad.2020.09.080](https://doi.org/https:/dx.doi.org/10.1016/j.jad.2020.09.080) | | Nurses not reported separately |
| [Çelik, N., Ceylan, B., Ünsal, A., & Çağan, Ö. (2019). Depression in health college students: relationship factors and sleep quality. *Psychology, Health and Medicine, 24*(5), 625-630. https://doi.org/10.1080/13548506.2018.1546881](https://doi.org/10.1080/13548506.2018.1546881) | | Nurses not reported separately |
| [Yurtsever, C., & Yurtsever, B. A. (2021). Depression, Anxiety and Sleep Disturbance in Primary Health Care Workers During the COVID-19 Pandemic. *Eurasian Journal of Family Medicine, 10*(4), 193-202. https://doi.org/10.33880/ejfm.2021100405](https://doi.org/10.33880/ejfm.2021100405) | | Nurses not reported separately |
| [Zhao, P., & Ma, X. L. (2021). Developing mental health nursing strategies for the inbound quarantined population in China during the COVID-19 global pandemic. *European Review for Medical and Pharmacological Sciences, 25*(24), 7964-7970. https://doi.org/https://dx.doi.org/10.26355/eurrev_202112_27646](https://doi.org/https:/dx.doi.org/10.26355/eurrev_202112_27646) | | Nurses not reported separately |
| [Booker, L. A., Spong, J., Hodge, B., Deacon-Crouch, M., Bish, M., Mills, J., & Skinner, T. C. (2024). Differences in shift and work-related patterns between metropolitan and regional/rural healthcare shift workers and the occupational health and safety risks. *The Australian journal of rural health, 32*(1), 141-151. https://doi.org/https://dx.doi.org/10.1111/ajr.13075](https://doi.org/https:/dx.doi.org/10.1111/ajr.13075) | | Nurses not reported separately |
| [Munir, F., & Nielsen, K. (2009). Does self-efficacy mediate the relationship between transformational leadership behaviours and healthcare workers' sleep quality? *A longitudinal study. Journal of Advanced Nursing, 65*(9), 1833-1843. https://doi.org/https://dx.doi.org/10.1111/j.1365-2648.2009.05039.x](https://doi.org/https:/dx.doi.org/10.1111/j.1365-2648.2009.05039.x) | | Nurses not reported separately |
| [Melo, G. A. A., Neto, J. C. G. L., Silva, R. A., Martins, M. G., Pereira, F. G. F., & Caetano, J. A. (2020). Effectiveness of auriculoacupuncture on the sleep quality of working nursing professionals during the COVID-19 pandemic. *Texto e Contexto Enfermagem, 29*, 1-14. https://doi.org/https://dx.doi.org/10.1590/1980-265X-TCE-2020-0392](https://doi.org/https:/dx.doi.org/10.1590/1980-265X-TCE-2020-0392) | | Nurses not reported separately |
| [Chen, S., Wu, H., Sun, M., Wei, Q., & Zhang, Q. (2023). Effects of shift work schedules, compensatory sleep, and work-family conflict on fatigue of shift-working nurses in Chinese intensive care units. *Nursing in critical care, 28*(6), 948-956. https://doi.org/https://dx.doi.org/10.1111/nicc.12909](https://doi.org/https:/dx.doi.org/10.1111/nicc.12909) | | Nurses not reported separately |
| [Nazario, E. G., da Silva, R. M., Beck, C. L. C., Centenaro, A. P. F. C., de Oliveira Freitas, E., Miranda, F. M. D., & Nicoletti, G. S. (2023). Fatigue and sleep in intensive care nursing workers in the COVID-19 pandemic. *ACTA Paulista de Enfermagem, 36*, eAPE000881. https://doi.org/https://dx.doi.org/10.37689/acta-ape/2023AO000881](https://doi.org/https:/dx.doi.org/10.37689/acta-ape/2023AO000881) | | Nurses not reported separately |
| [Azizoddin, D. R., Kvaternik, N., Beck, M., Zhou, G., Hasdianda, M. A., Jones, N., Johnsky, L., Im, D., Chai, P. R., & Boyer, E. W. (2021). Heal the Healers: A pilot study evaluating the feasibility, acceptability, and exploratory efficacy of a Transcendental Meditation intervention for emergency clinicians during the coronavirus disease 2019 pandemic. *JACEP Open, 2*(6). https://doi.org/10.1002/emp2.12619](https://doi.org/10.1002/emp2.12619) | | Nurses not reported separately |
| [Abdelghani, M., Mahdy, R. S., & El-Gohari, H. M. (2021). Health anxiety to COVID-19 virus infection and its relationship to quality of life in a sample of health care workers in Egypt: A cross-sectional study. *Archives of Psychiatry and Psychotherapy, 23*(1), 19-28. https://doi.org/https://dx.doi.org/10.12740/APP/130304](https://doi.org/https:/dx.doi.org/10.12740/APP/130304) | | Nurses not reported separately |
| [Cena, H., Porri, D., De Giuseppe, R., Kalmpourtzidou, A., Salvatore, F. P., Ghoch, M. E., Itani, L., Kreidieh, D., Brytek-Matera, A., Pocol, C. B., Arteta, D. S. A., Utan, G., & Kolcic, I. (2021). How healthy are health-related behaviors in university students: The holistic study. *Nutrients, 13*(2), 1-16. https://doi.org/https://dx.doi.org/10.3390/nu13020675](https://doi.org/https:/dx.doi.org/10.3390/nu13020675) | | Nurses not reported separately |
| [Jafari, M. J., Mostafazadeh, P., Mojebi, M. R., Nemati-Vakilabad, R., & Mirzaei, A. (2024). Identifying predictors of patient safety competency based on sleep quality in student faculty of nursing and midwifery during the internship period: a multidisciplinary study. *BMC Nursing, 23*(1), 67. https://doi.org/https://dx.doi.org/10.1186/s12912-024-01725-2](https://doi.org/https:/dx.doi.org/10.1186/s12912-024-01725-2) | | Nurses not reported separately |
| [Ripoll, J., Chela, X., Briones-VozmedianoFiol-de Roque, E. M., Zamanillo-Campos, R., Ricci-Cabello, I., Llobera, J., Calafat-Villalonga, C., & Serrano-Ripoll, M. J. (2024). Impact of COVID-19 on mental health of health care workers in Spain: a mix-methods study. *BMC public health, 24(*1), 463. https://doi.org/https://dx.doi.org/10.1186/s12889-024-17979-z](https://doi.org/https:/dx.doi.org/10.1186/s12889-024-17979-z) | | Nurses not reported separately |
| [Gulati, P., Choudhry, D., Sharma, N., Dhankher, P., Manshi, & Kumari, A. (2023). Impact of Post-acute COVID-19 Syndrome on Mental Health of Healthcare Professionals: A Cross-sectional Study. *Journal of Clinical and Diagnostic Research, 17*(9), YC10-YC14. https://doi.org/https://dx.doi.org/10.7860/JCDR/2023/62723.18422](https://doi.org/https:/dx.doi.org/10.7860/JCDR/2023/62723.18422) | | Nurses not reported separately |
| [Kim, H. I., Jung, S.-A., Choi, J. Y., Kim, S.-E., Jung, H.-K., Shim, K.-N., & Yoo, K. (2013). Impact of shiftwork on irritable bowel syndrome and functional dyspepsia. *Journal of Korean medical science, 28*(3), 431-437. https://doi.org/https://dx.doi.org/10.3346/jkms.2013.28.3.431](https://doi.org/https:/dx.doi.org/10.3346/jkms.2013.28.3.431) | | Nurses not reported separately |
| [Garcia-Tudela, A., Simonelli-Munoz, A. J., Gallego-Gomez, J. I., & Rivera-Caravaca, J. M. (2023). Impact of the COVID-19 pandemic on stress and sleep in emergency room professionals. *Journal of Clinical Nursing, 32*(15-16), 5037-5045. https://doi.org/https://dx.doi.org/10.1111/jocn.16738](https://doi.org/https:/dx.doi.org/10.1111/jocn.16738) | | Nurses not reported separately |
| [Cabeza, G., Caballero, H., Castiblanco, F., Cabrera, D., Martinez, F., & Lara, G. (2022). Impact of the COVID-19 pandemic on the quality of sleep of mental health workers in Colombia. *Dreaming, 32*(2), 124-134. https://doi.org/https://dx.doi.org/10.1037/drm0000210](https://doi.org/https:/dx.doi.org/10.1037/drm0000210) | | Nurses not reported separately |
| [Shrimal, P. J., Maharana, S., Dave, A., Metri, K. G., Raghuram, N., & Shrimal, S. (2024). Impact of Yoga on anxiety, stress and sleep quality among health care professionals during public health crisis. *Work (Reading, Mass.)*. https://doi.org/https://dx.doi.org/10.3233/WOR-230061](https://doi.org/https:/dx.doi.org/10.3233/WOR-230061) | | Nurses not reported separately |
| [Varman, A., Nithiya, D. R., Meghna, C., Tipandjan, A., Thiagarajan, S., Saravanan, N., & Sivakumar, G. (2023). Influence of N95 Mask-related Hypoxemia on Headache, Stress, Anxiety, and Quality of Sleep during COVID-19 Patient Care among Frontline Health Care Professionals. *The Journal of the Association of Physicians of India, 71*(3), 11-12. https://doi.org/https://dx.doi.org/10.5005/japi-11001-0176](https://doi.org/https:/dx.doi.org/10.5005/japi-11001-0176) | | Nurses not reported separately |
| [Drager, L. F., Pachito, D. V., Moreno, C. R. C., Tavares, A. R., Conway, S. G., Assis, M., Sguillar, D. A., Moreira, G. A., Bacelar, A., & Genta, P. R. (2022). Insomnia episodes, new-onset pharmacological treatments, and other sleep disturbances during the COVID-19 pandemic: a nationwide cross-sectional study in Brazilian health care professionals. *Journal of Clinical Sleep Medicine, 18*(2), 373-382. https://doi.org/https://dx.doi.org/10.5664/jcsm.9570](https://doi.org/https:/dx.doi.org/10.5664/jcsm.9570) | | Nurses not reported separately |
| [Jin, L.-R., Zhou, S., & Yuan, H. (2022). Investigation on sleep quality in Chinese medical staff during the COVID-19 pandemic. *Psychology, health & medicine, 27*(2), 396-402. https://doi.org/https://dx.doi.org/10.1080/13548506.2021.1922722](https://doi.org/https:/dx.doi.org/10.1080/13548506.2021.1922722) | | Nurses not reported separately |
| [Luz, E. M. S., Marqueze, E., & Moreno, C. (2011). Job satisfaction and sleep quality in nursing professionals. *Sleep Science, 4*(2), 49-51. https://www.scopus.com/inward/record.uri?eid=2-s2.0-84892969620&partnerID=40&md5=a003761fa88719e5bbe3bc4c2f3bc8cc](https://www.scopus.com/inward/record.uri?eid=2-s2.0-84892969620&partnerID=40&md5=a003761fa88719e5bbe3bc4c2f3bc8cc) | | Nurses not reported separately |
| [Karhula, K., Harma, M., Sallinen, M., Hublin, C., Virkkala, J., Kivimaki, M., Vahtera, J., & Puttonen, S. (2013). Job strain, sleep and alertness in shift working health care professionals -- a field study. *Industrial Health, 51*(4), 406-416. https://doi.org/https://dx.doi.org/10.2486/indhealth.2013-0015](https://doi.org/https:/dx.doi.org/10.2486/indhealth.2013-0015) | | Nurses not reported separately |
| [Zhao, X., Zhang, T., Li, B., Yu, X., Ma, Z., Cao, L., Gu, Q., Dong, C., Jin, Y., Fan, J., & He, G. (2020). Job-related factors associated with changes in sleep quality among healthcare workers screening for 2019 novel coronavirus infection: a longitudinal study. *Sleep Medicine, 75,* 21-26. https://doi.org/https://dx.doi.org/10.1016/j.sleep.2020.07.027](https://doi.org/https:/dx.doi.org/10.1016/j.sleep.2020.07.027) | | Nurses not reported separately |
| [Wang, Q., Chu, H., Qu, P., Fang, H., Liang, D., Liu, S., Li, J., & Liu, A. (2023). Machine-learning prediction of BMI change among doctors and nurses in North China during the COVID-19 pandemic. *Frontiers in Nutrition, 10.* https://doi.org/10.3389/fnut.2023.1019827](https://doi.org/10.3389/fnut.2023.1019827) | | Nurses not reported separately |
| [Tang, X., Pei, Y., Wang, X., Jiang, L., Liu, P., Chen, Y., & Meng, Z. (2022). Mental health and fatigue status of the medical workforce during the COVID-19 outbreak in the Yangzhou city, China. *Frontiers in Psychiatry, 13,* 1018069. https://doi.org/https://dx.doi.org/10.3389/fpsyt.2022.1018069](https://doi.org/https:/dx.doi.org/10.3389/fpsyt.2022.1018069) | | Nurses not reported separately |
| [Bzeih, W., Kim, S., Yi, K., & Chung, S. (2023). No Association Between Insomnia Severity, and Discrepancy Between Desired Time in Bed and Desired Total Sleep Time Among Shift-Working Nursing Professionals. *Sleep Medicine Research, 14*(3), 155-160. https://doi.org/https://dx.doi.org/10.17241/smr.2023.01704](https://doi.org/https:/dx.doi.org/10.17241/smr.2023.01704) | | Nurses not reported separately |
| [Haseli, A., Egdampur, F., Qaderi, K., Kaffashian, M.R., & Delpisheh, A. (2023). Nurses and midwives' sleep quality and itsassociated factors during the early COVID-19 pandemic in Iran*. Heliyon, 9(*4). https://doi.org/10.1016/j.heliyon.2023.e15068](https://doi.org/10.1016/j.heliyon.2023.e15068) | | Nurses not reported separately |
| Karhula, K., Hakola, T., Koskinen, A., Ojajarvi, A., Kivimaki, M., & Harma, M. (2018). Permanent night workers' sleep and psychosocial factors in hospital work. A comparison to day and shift work. *Chronobiology International*, *35(*6), 785-794. https://doi.org/https://dx.doi.org/10.1080/07420528.2018.1466792 | | Nurses not reported separately |
| [Sato, T. D. O., de Faria, B. S. F., Albuquerque, B. B., Silva, F. L. D., Rohwedder, L. S., de Azevedo, R. T., Gonçalves, J. S., Vieira, L. M. S. M. D. A., Triches, M. I., de Sousa, R. A., Cardoso, V. D. F., & Mininel, V. A. (2022). Poor Health Conditions among Brazilian Healthcare Workers: The Study Design and Baseline Characteristics of the HEROES Cohort. *Healthcare (Switzerland), 10*(10). https://doi.org/10.3390/healthcare10102096](https://doi.org/10.3390/healthcare10102096) | | Nurses not reported separately |
| [Coelho, J., Lucas, G., Micoulaud-Franchi, J.-A., Philip, P., Boyer, L., & Fond, G. (2023). Poor sleep is associated with work environment among 10,087 French healthcare workers: Results from a nationwide survey. *Psychiatry research, 328,* 115448. https://doi.org/https://dx.doi.org/10.1016/j.psychres.2023.115448](https://doi.org/https:/dx.doi.org/10.1016/j.psychres.2023.115448) | | Nurses not reported separately |
| [Yang, Y., Shi, T., Song, Y., Zhou, Y., Zhang, Z., Guo, Y., Li, X., Liu, Y., Xu, G., Cheung, T., Xiang, Y. T., & Tang, Y. (2020). Prevalence and Demographic Correlates of Poor Sleep Quality Among Frontline Health Professionals in Liaoning Province, China During the COVID-19 Outbreak. *Frontiers in Psychiatry, 11,* 520. https://doi.org/https://dx.doi.org/10.3389/fpsyt.2020.00520](https://doi.org/https:/dx.doi.org/10.3389/fpsyt.2020.00520) | | Nurses not reported separately |
| [Al Mawaali, Z., Abdelrahman, H., Al Qadire, M., Ballad, C. A. C., Al Busafi, S., Al Busaidi, B., Al Mahari, F., Al Balushi, M., Al Rawahi, I., Al Omari, O., Almaawali, M., & Khalaf, A. (2024). Prevalence of Anxiety, Depression, and Sleep Disturbance Among Emergency Nurses in Oman. *Journal of emergency nursing.* https://doi.org/https://dx.doi.org/10.1016/j.jen.2024.02.004](https://doi.org/https:/dx.doi.org/10.1016/j.jen.2024.02.004) | | Nurses not reported separately |
| [Alghamdi, L. A., Alsubhi, L. S., Alghamdi, R. M., Aljahdaly, N. M., Barashid, M. M., Wazira, L. A., Batawi, G. A., Manzar, M. D., Alshumrani, R. A., Alhejaili, F. F., & Wali, S. O. (2024). Prevalence of poor sleep quality among physicians and nurses in a tertiary health care center. *Journal of Taibah University Medical Sciences, 19*(3), 473-481. https://doi.org/https://dx.doi.org/10.1016/j.jtumed.2024.03.003](https://doi.org/https:/dx.doi.org/10.1016/j.jtumed.2024.03.003) | | Nurses not reported separately |
| [Sahin, S. K., Arslan, E., Atalay, U. M., Demir, B., Elboga, G., & Altindag, A. (2022). Psychological impact of COVID-19 outbreak on health workers in a university hospital in Turkey. *Psychology, health & medicine, 2*7(1), 81-90. https://doi.org/https://dx.doi.org/10.1080/13548506.2021.1900578](https://doi.org/https:/dx.doi.org/10.1080/13548506.2021.1900578) | | Nurses not reported separately |
| [Lin, Y.-Q., Lin, Z.-X., Wu, Y.-X., Wang, L., Zeng, Z.-N., Chen, Q.-Y., Wang, L., Xie, X.-L., & Wei, S.-C. (2020). Reduced Sleep Duration and Sleep Efficiency Were Independently Associated With Frequent Nightmares in Chinese Frontline Medical Workers During the Coronavirus Disease 2019 Outbreak. *Frontiers in neuroscience, 14,* 631025. https://doi.org/https://dx.doi.org/10.3389/fnins.2020.631025](https://doi.org/https:/dx.doi.org/10.3389/fnins.2020.631025) | | Nurses not reported separately |
| [Jafari Roodbandi, A., Choobineh, A., & Daneshvar, S. (2015). Relationship between circadian rhythm amplitude and stability with sleep quality and sleepiness among shift nurses and health care workers. *International journal of occupational safety and ergonomics : JOSE, 21*(3), 312-317. https://doi.org/https://dx.doi.org/10.1080/10803548.2015.1081770](https://doi.org/https:/dx.doi.org/10.1080/10803548.2015.1081770) | | Nurses not reported separately |
| [Bukowska, A., Sobala, W., & Peplonska, B. (2015). Rotating night shift work, sleep quality, selected lifestyle factors and prolactin concentration in nurses and midwives. *Chronobiology International, 32*(3), 318-326. https://doi.org/https://dx.doi.org/10.3109/07420528.2014.975353](https://doi.org/https:/dx.doi.org/10.3109/07420528.2014.975353) | | Nurses not reported separately |
| [Layek, A. K., Ghosh, N., Biswas, J., & Bhaduri, S. (2021). Self-isolation of healthcare workers during covid-19 pandemic in a tertiary care center - association between their sleep quality, anxiety status and social capital. I*ndian Journal of Forensic Medicine and Toxicology, 15*(3), 129-136. https://doi.org/https://dx.doi.org/10.37506/ijfmt.v15i3.15292](https://doi.org/https:/dx.doi.org/10.37506/ijfmt.v15i3.15292) | | Nurses not reported separately |
| [Edell-Gustafsson, U. M., Kritz, E. I. K., & Bogren, I. K. (2002). Self-reported sleep quality, strain and health in relation to perceived working conditions in females. *Scandinavian Journal of Caring Sciences, 16*(2), 179-187. https://doi.org/https://dx.doi.org/10.1046/j.1471-6712.2002.00078.x](https://doi.org/https:/dx.doi.org/10.1046/j.1471-6712.2002.00078.x) | | Nurses not reported separately |
| [Harma, M., Karhula, K., Puttonen, S., Ropponen, A., Koskinen, A., Ojajarvi, A., & Kivimaki, M. (2019). Shift work with and without night work as a risk factor for fatigue and changes in sleep length: A cohort study with linkage to records on daily working hours. *Journal of Sleep Research, 28*(3), e12658. https://doi.org/https://dx.doi.org/10.1111/jsr.12658](https://doi.org/https:/dx.doi.org/10.1111/jsr.12658) | | Nurses not reported separately |
| [Paiva, T., Reis, C., Feliciano, A., Canas-Simiao, H., Machado, M. A., Gaspar, T., Tome, G., Branquinho, C., Silva, M. R., Ramiro, L., Gaspar, S., Bentes, C., Sampaio, F., Pinho, L., Pereira, C., Carreiro, A., Moreira, S., Luzeiro, I., Pimentel, J.,…Matos, M. G. (2021). Sleep and awakening quality during COVID-19 confinement: Complexity and relevance for health and behavior. *International Journal of Environmental Research and Public Health, 18*(7), 3506. https://doi.org/https://dx.doi.org/10.3390/ijerph18073506](https://doi.org/https:/dx.doi.org/10.3390/ijerph18073506) | | Nurses not reported separately |
| [Nugent, K., Raj, R., & Nugent, R. (2020). Sleep Patterns and Health Behaviors in Healthcare Students. *Southern Medical Journal, 113*(3), 104-110. https://doi.org/https://dx.doi.org/10.14423/SMJ.0000000000001077](https://doi.org/https:/dx.doi.org/10.14423/SMJ.0000000000001077) | | Nurses not reported separately |
| [Hassinger, A. B., Breuer, R. K., & Mishra, A. (2022). Sleep patterns of US healthcare workers during the first wave of the COVID-19 pandemic. *Sleep and Breathing, 26*(3), 1351-1361. https://doi.org/https://dx.doi.org/10.1007/s11325-021-02515-9](https://doi.org/https:/dx.doi.org/10.1007/s11325-021-02515-9) | | Nurses not reported separately |
| [Tmava-Berisha, A., Fellendorf, F. T., Ratzenhofer, M., Maget, A., Platzer, M., Bengesser, S. A., Birner, A., Queissner, R., Schonthaler, E., Dalkner, N., Lenger, M., & Reininghaus, E. Z. (2022). Sleep quality among workers in the health sector exposed to the COVID-19 pandemic. *PLoS ONE, 17*(12), e0268933. https://doi.org/https://dx.doi.org/10.1371/journal.pone.0268933](https://doi.org/https:/dx.doi.org/10.1371/journal.pone.0268933) | | Nurses not reported separately |
| [Zheng, Y., Wang, L., Feng, L., Ye, L., Zhang, A., & Fan, R. (2021). Sleep quality and mental health of medical workers during the coronavirus disease 2019 pandemic. *Sleep and Biological Rhythms, 19(*2), 173-180. https://doi.org/https://dx.doi.org/10.1007/s41105-020-00304-7](https://doi.org/https:/dx.doi.org/10.1007/s41105-020-00304-7) | | Nurses not reported separately |
| [Waich, A., Barahona-Correa, J. E., del Carmen Figueredo, M., Rondon-Sepulveda, M. A., Ruiz, A. J., Castellanos, J. C., & Hidalgo-Martinez, P. (2023). Sleep Quality, Insomnia, and Perceived Stress among Colombian Healthcare Workers during the COVID-19 Pandemic. *Sleep Science, 16*(1), 44-50. https://doi.org/https://dx.doi.org/10.1055/S-0043-1767756](https://doi.org/https:/dx.doi.org/10.1055/S-0043-1767756) | | Nurses not reported separately |
| [Coelho, J., Lucas, G., Micoulaud-Franchi, J.-A., Tran, B., Yon, D. K., Taillard, J., D'Incau, E., Philip, P., Boyer, L., & Fond, G. (2023). Sleep timing, workplace well-being and mental health in healthcare workers. *Sleep Medicine, 111,* 123-132. https://doi.org/https://dx.doi.org/10.1016/j.sleep.2023.09.013](https://doi.org/https:/dx.doi.org/10.1016/j.sleep.2023.09.013) | | Nurses not reported separately |
| [Martinez-Arriaga, R. J., Dominguez-Rodriguez, A., Herdoiza-Arroyo, P. E., Robles-Garcia, R., de la Rosa-Gomez, A., Figueroa Gonzalez, J. A., & Munoz Anacona, Y. A. (2023). Suicide risk and associated factors in healthcare workers seeking psychological support during COVID-19: a cross-sectional study. *Psychology, health & medicine, 28*(10), 3076-3090. https://doi.org/https://dx.doi.org/10.1080/13548506.2023.2216469](https://doi.org/https:/dx.doi.org/10.1080/13548506.2023.2216469) | | Nurses not reported separately |
| [Duru, H. (2022). The Continuing Effect of COVID-19 Pandemic on Physical Well-Being and Mental Health of ICU Healthcare Workers in Turkey: A Single-Centre Cross-Sectional Later-Phase Study. *Journal of Intensive Care Medicine, 37(*9), 1206-1214. https://doi.org/https://dx.doi.org/10.1177/08850666211070740](https://doi.org/https:/dx.doi.org/10.1177/08850666211070740) | | Nurses not reported separately |
| [Shamsipour, S., Sahebihagh, M. H., Ebrahimi, H., Mohammadpoorasl, A., & Areshtanab, H. N. (2018). The effect of aromatherapy with citrus aurantium on sleep quality among nursing students: A randomized clinical trial. *Acta Facultatis Medicae Naissensis, 35*(3), 236-243. https://doi.org/https://dx.doi.org/10.2478/afmnai-2018-0025](https://doi.org/https:/dx.doi.org/10.2478/afmnai-2018-0025) | | Nurses not reported separately |
| [Jahrami, H., BaHammam, A. S., AlGahtani, H., Ebrahim, A., Faris, M. A. I., AlEid, K., Saif, Z., Haji, E., Dhahi, A., Marzooq, H., Hubail, S., & Hasan, Z. (2021). The examination of sleep quality for frontline healthcare workers during the outbreak of COVID-19. *Sleep and Breathing, 25*(1), 503-511. https://doi.org/https://dx.doi.org/10.1007/s11325-020-02135-9](https://doi.org/https:/dx.doi.org/10.1007/s11325-020-02135-9) | | Nurses not reported separately |
| [Alhamed, A. A. (2023). The link among academic stress, sleep disturbances, depressive symptoms, academic performance, and the moderating role of resourcefulness in health professions students during COVID-19 pandemic. *Journal of professional nursing : official journal of the American Association of Colleges of Nursing, 46,* 83-91. https://doi.org/https://dx.doi.org/10.1016/j.profnurs.2023.02.010](https://doi.org/https:/dx.doi.org/10.1016/j.profnurs.2023.02.010) | | Nurses not reported separately |
| [Filipponi, C., Pizzoli, S. F. M., Masiero, M., Cutica, I., & Pravettoni, G. (2024). The Partial Mediator Role of Satisficing Decision-Making Style Between Trait Emotional Intelligence and Compassion Fatigue in Healthcare Professionals. *Psychological reports, 127(*2), 868-886. https://doi.org/https://dx.doi.org/10.1177/00332941221129127](https://doi.org/https:/dx.doi.org/10.1177/00332941221129127) | | Nurses not reported separately |
| [Nogueira, L. F. R., Pellegrino, P., Cipolla-Neto, J., Moreno, C. R. C., & Marqueze, E. C. (2021). Timing and Composition of Last Meal before Bedtime Affect Sleep Parameters of Night Workers. *Clocks and Sleep, 3*(4), 536-546. https://doi.org/10.3390/clockssleep3040038](https://doi.org/10.3390/clockssleep3040038) | | Nurses not reported separately |
| [Oneib, B., Filali, A. E., & Abda, N. (2023). Translation and Validation of the Pittsburgh Sleep Quality Index in the Moroccan Dialect. *Sleep Medicine Research, 14*(4), 213-219. https://doi.org/https://dx.doi.org/10.17241/smr.2023.01760](https://doi.org/https:/dx.doi.org/10.17241/smr.2023.01760) | | Nurses not reported separately |
| [Erbil, N., & Yucesoy, H. (2022). Relationship between premenstrual syndrome and sleep quality among nursing and medical students. *Perspectives in Psychiatric Care, 58(*2), 448-455. https://doi.org/https://dx.doi.org/10.1111/ppc.12628](https://doi.org/https:/dx.doi.org/10.1111/ppc.12628) | | Nurses not reported separately |
| Bukowska-Damska, A., Reszka, E., Kaluzny, P., Wieczorek, E., Przybek, M., Zienolddiny, S., & Peplonska, B. (2017). Sleep quality and methylation status of core circadian rhythm genes among nurses and midwives. *Chronobiology International, 34*(9), 1211–1223. https://doi.org/10.1080/07420528.2017.1358176 | | Nurses not reported separately |
| [Centofanti, S., Banks, S., Colella, A., Dingle, C., Devine, L., Galindo, H., Pantelios, S., Brkic, G., & Dorrian, J. (2018). Coping with shift work-related circadian disruption: A mixed-methods case study on napping and caffeine use in Australian nurses and midwives. *Chronobiology International, 35*(6), 853-864. https://doi.org/https://dx.doi.org/10.1080/07420528.2018.1466798](https://doi.org/https:/dx.doi.org/10.1080/07420528.2018.1466798) | | Nurses not reported separately |
| [Saynajakangas, P., Manttari, S., Selander, K., & Laitinen, J. (2024). Home Care Workers' Objective and Subjective Recovery From Work. *Journal of Occupational and Environmental Medicine, 66*(2), 161-165. https://doi.org/https://dx.doi.org/10.1097/JOM.0000000000003013](https://doi.org/https:/dx.doi.org/10.1097/JOM.0000000000003013) | | Nurses not reported separately |
| [Lobo, L. M. D. G. A., Evora, Y. D. M., Dos Santos, A. M. R., Gouveia, M. T. O., & Andrade, E. M. L. R. (2022). FACTORS ASSOCIATED WITH SMARTPHONE ADDICTION IN NURSING STUDENTS. *Texto e Contexto Enfermagem, 31,* e20210045. https://doi.org/https://dx.doi.org/10.1590/1980-265X-TCE-2021-0045](https://doi.org/https:/dx.doi.org/10.1590/1980-265X-TCE-2021-0045) | | Nurses not reported separately |
| Bukowska-Damska, A., Reszka, E., Kaluzny, P., Wieczorek, E., Przybek, M., Zienolddiny, S., & Peplonska, B. (2017). Sleep quality and methylation status of core circadian rhythm genes among nurses and midwives. *Chronobiology International*, *34*(9), 1211–1223. https://doi.org/10.1080/07420528.2017.1358176 | | Nurses not reported separately |
| [Huang, Y., & Zhao, N. (2020). Chinese mental health burden during the COVID-19 pandemic. *Asian Journal of Psychiatry, 51*, 102052. https://doi.org/https://dx.doi.org/10.1016/j.ajp.2020.102052](https://doi.org/https:/dx.doi.org/10.1016/j.ajp.2020.102052) | | Retracted |
| [Jeong, Y. M., & Min, A. (2021). Depression, Help-Seeking Attitude, Sleep Quality, and Missed Nursing Care Among Nurses in Korean Hospitals: A Cross-Sectional Study. *Journal of Nursing Scholarship.* https://doi.org/https://dx.doi.org/10.1111/jnu.12647](https://doi.org/https:/dx.doi.org/10.1111/jnu.12647) | | Retracted |
| [Liu, Y., Jiang, T. T., Shi, T. Y., Liu, Y. N., Liu, X. M., Xu, G. J., Li, F. L., Wang, Y. L., & Wu, X. Y. (2020). The effectiveness of diaphragmatic breathing relaxation training for improving sleep quality among nursing staff during the COVID-19 outbreak: a before and after study. *Sleep Medicine, 2,* 100026. https://doi.org/https://dx.doi.org/10.1016/j.sleepx.2020.100026](https://doi.org/https:/dx.doi.org/10.1016/j.sleepx.2020.100026) | | Retracted |
| [Chang, Y.-S., Chen, H.-L., Hsu, C.-Y., Su, S.-F., Liu, C.-K., & Hsu, C. (2013). Nurses working on fast rotating shifts overestimate cognitive function and the capacity of maintaining wakefulness during the daytime after a rotating shift. *Sleep Medicine, 14*(7), 605-613. https://doi.org/https://dx.doi.org/10.1016/j.sleep.2013.03.011](https://doi.org/https:/dx.doi.org/10.1016/j.sleep.2013.03.011) | | Sleep Duration collected via PSG |
| [Kasimis, I., Perdikaris, P., Boutopoulou, V., Tsanaka, A., Mantziou, T., Skodra, A., Effrosyni, V., & Matziou, V. (2023). Sleep Disturbance and Occupational Fatigue in Emergency Nurses of Public Hospitals in Greece: A Cross-Sectional Study. *Advanced emergency nursing journal, 45*(3), 230-239. https://doi.org/https://dx.doi.org/10.1097/TME.0000000000000473](https://doi.org/https:/dx.doi.org/10.1097/TME.0000000000000473) | | Sleep Duration measured TIB |
| [Boned-Galán, Á., López-Ibort, N., & Gascón-Catalán, A. (2022). Sleep disturbances in nurse managers during the early and late stages of the COVID-19 pandemic. *Frontiers in Psychology, 13.* https://doi.org/10.3389/fpsyg.2022.1070355](https://doi.org/10.3389/fpsyg.2022.1070355) | | Sleep Duration measured TIB |
| [Lim, S., Han, K., Cho, H., & Baek, H. (2019). Shift-work nurses' work environments and health-promoting behaviours in relation to sleep disturbance: A cross-sectional secondary data analysis. *Journal of clinical nursing, 28*(9-10), 1538-1545. https://doi.org/https://dx.doi.org/10.1111/jocn.14769](https://doi.org/https:/dx.doi.org/10.1111/jocn.14769) | | Sleep Duration measured TIB |
| [Ohida, T., Takemura, S., Nozaki, N., Kawahara, K., Sugie, T., & Uehata, T. (2001). The influence of lifestyle and night-shift work on sleep problems among female hospital nurses in Japan. *[Nippon kōshū eisei zasshi] Japanese journal of public health, 48*(8), 595-603. https://www.scopus.com/inward/record.uri?eid=2-s2.0-0035431466&partnerID=40&md5=47530ed5e345e7dd5576acd20838699d](https://www.scopus.com/inward/record.uri?eid=2-s2.0-0035431466&partnerID=40&md5=47530ed5e345e7dd5576acd20838699d) | | Sleep duration/ quality measured not reported |
| [Chen, X., Liu, P., Lei, G.-F., Tong, L., Wang, H., & Zhang, X.-Q. (2021). Sleep quality and the depression-anxiety-stress state of frontline nurses who perform nucleic acid sample collection during COVID-19: A cross-sectional study. *Psychology Research and Behavior Management, 14.* https://doi.org/https://dx.doi.org/10.2147/PRBM.S338495](https://doi.org/https:/dx.doi.org/10.2147/PRBM.S338495) | | Sleep duration/ quality measured not reported |
| [Dai, X., Zhao, Q., Li, J., & Pan, C. (2021). The prevalence and risk factors for anxiety in frontline nurses under COVID-19 pandemic based on a large cross-sectional study using the propensity score-matched method. *Revista de Psiquiatria Clinica, 48*(5), 221-228. https://doi.org/https://dx.doi.org/10.15761/0101-60830000000311](https://doi.org/https:/dx.doi.org/10.15761/0101-60830000000311) | | Sleep duration/ quality measured not reported |
| [Huang, Q., Tian, C., & Zeng, X.-T. (2021). Poor Sleep Quality in Nurses Working or Having Worked Night Shifts: A Cross-Sectional Study. *Frontiers in Neuroscience, 15,* 638973. https://doi.org/https://dx.doi.org/10.3389/fnins.2021.638973](https://doi.org/https:/dx.doi.org/10.3389/fnins.2021.638973) | | Sleep duration/ quality measured not reported |
| [Rizza, S., Luzi, A., Mavilio, M., Ballanti, M., Massimi, A., Porzio, O., Magrini, A., Hannemann, J., Menghini, R., Lehrke, M., Staels, B., Grant, P. J., Boger, R. H., Marx, N., & Federici, M. (2021). Alterations in Rev-ERBα/BMAL1 ratio and glycated hemoglobin in rotating shift workers: the EuRhythDia study. *Acta Diabetologica, 58*(8), 1111-1117. https://doi.org/10.1007/s00592-021-01676-z](https://doi.org/10.1007/s00592-021-01676-z) | | Sleep duration/ quality measured not reported |
| [Simonetti, V., Durante, A., Ambrosca, R., Arcadi, P., Graziano, G., Pucciarelli, G., Simeone, S., Vellone, E., Alvaro, R., & Cicolini, G. (2021). Anxiety, sleep disorders and self-efficacy among nurses during COVID-19 pandemic: A large cross-sectional study. *Journal of clinical nursing, 30*(9-10), 1360-1371. https://doi.org/https://dx.doi.org/10.1111/jocn.15685](https://doi.org/https:/dx.doi.org/10.1111/jocn.15685) | | Sleep duration/ quality measured not reported |
| [Vancampfort, D., & Mugisha, J. (2022). Mental health and lifestyle in mental health nurses: a cross-sectional, nation-wide study from Uganda during COVID-19 times. *Pan African Medical Journal, 42*(210). https://doi.org/https://dx.doi.org/10.11604/PAMJ.2022.42.210.33928](https://doi.org/https:/dx.doi.org/10.11604/PAMJ.2022.42.210.33928) | | Sleep duration/ quality measured not reported |
| [Vlahoyiannis, A., Karali, E., Giannaki, C. D., Karioti, A., Pappas, A., Lavdas, E., Karatzaferi, C., & Sakkas, G. K. (2022). The vicious circle between physical, psychological, and physiological characteristics of shift work in nurses: a multidimensional approach. *Sleep and Breathing, 26*(1), 149-156. https://doi.org/https://dx.doi.org/10.1007/s11325-021-02381-5](https://doi.org/https:/dx.doi.org/10.1007/s11325-021-02381-5) | | Sleep duration/ quality measured not reported |
| [Wang, H., Dai, X., Yao, Z., Zhu, X., Jiang, Y., Li, J., & Han, B. (2021). The prevalence and risk factors for depressive symptoms in frontline nurses under COVID-19 pandemic based on a large cross-sectional study using the propensity score-matched method. *BMC Psychiatry, 21*(1), 152. https://doi.org/https://dx.doi.org/10.1186/s12888-021-03143-z](https://doi.org/https:/dx.doi.org/10.1186/s12888-021-03143-z) | | Sleep duration/ quality measured not reported |
| [Wang, S., Fu, W., Jiang, Y., Deng, L., Zhou, S., & Yang, L. (2017). Association of plasma leptin levels with type 2 diabetes in rotating shift work women. *Biomedical Research (India), 28*(1), 295-298. http://www.biomedres.info/biomedical-research/association-of-plasma-leptin-levels-with-type-2-diabetes-in-rotating-shift-work-women.pdfhttp://ovidsp.ovid.com/ovidweb.cgi?T=JS&PAGE=reference&D=emed18&NEWS=N&AN=614096508](http://www.biomedres.info/biomedical-research/association-of-plasma-leptin-levels-with-type-2-diabetes-in-rotating-shift-work-women.pdfhttp:/ovidsp.ovid.com/ovidweb.cgi?T=JS&PAGE=reference&D=emed18&NEWS=N&AN=614096508) | | Sleep duration/ quality measured not reported |
| [Xiong, W., Huang, J., & Zhu, A. (2021). The relationship of sleep quality among internship nurses with clinical learning environment and mental stress: a cross-sectional survey. *Sleep Medicine, 83,* 151-158. https://doi.org/https://dx.doi.org/10.1016/j.sleep.2021.04.034](https://doi.org/https:/dx.doi.org/10.1016/j.sleep.2021.04.034) | | Sleep duration/ quality measured not reported |
| [Dehghan, H., Azmoon, H., Souri, S., & Akbari, J. (2014). The effects of state anxiety and thermal comfort on sleep quality and eye fatigue in shift work nurses. *Journal of Education and Health Promotion, 3,* 72. https://doi.org/https://dx.doi.org/10.4103/2277-9531.134870](https://doi.org/https:/dx.doi.org/10.4103/2277-9531.134870) | | Sleep duration/ quality measured not reported |
| [Ghasemi, F., Samavat, P., & Soleimani, F. (2019). The links among workload, sleep quality, and fatigue in nurses: a structural equation modeling approach. Fatigue: Biomedicine, *Health and Behavior, 7*(3), 141-152. https://doi.org/https://dx.doi.org/10.1080/21641846.2019.1652422](https://doi.org/https:/dx.doi.org/10.1080/21641846.2019.1652422) | | Sleep duration/ quality measured not reported |
| [Munjappa, H. B., Shinde, S. A., Parekh, M. K., & Ingale, S. S. (2022). A cross-sectional study of quality of sleep in medical healthcare workers from a tertiary healthcare center during COVID-19 pandemic. *National Journal of Physiology, Pharmacy and Pharmacology, 12*(7), 1038-1041. https://doi.org/https://dx.doi.org/10.5455/njppp.2022.12.05227202223052022](https://doi.org/https:/dx.doi.org/10.5455/njppp.2022.12.05227202223052022) | | Sleep duration/ quality measured not reported |
| [ang, J., Qiu, C., Xu, H., & You, G. (2013). A model for predicting acute and chronic fatigue in Chinese nurses. *Journal of Advanced Nursing, 69*(3), 546-558. https://doi.org/https://dx.doi.org/10.1111/j.1365-2648.2012.06029.x](https://doi.org/https:/dx.doi.org/10.1111/j.1365-2648.2012.06029.x) | | Sleep duration/ quality measured not reported |
| [Zhang, X., Lee, S.-Y., Luo, H., & Liu, H. (2019). A prediction model of sleep disturbances among female nurses by using the BP-ANN. *Journal of Nursing Management, 27*(6), 1123-1130. https://doi.org/https://dx.doi.org/10.1111/jonm.12782](https://doi.org/https:/dx.doi.org/10.1111/jonm.12782) | | Sleep duration/ quality measured not reported |
| [Liu, S., Li, X., Yin, X., & Wang, L. (2022). Analysis of the Effect of Rational Emotional Intervention Combined with Hierarchical Management Mode on Improving the Psychological Stress of Emergency Nurses and Trainee Nurses. *Emergency Medicine International, 2022,* 2038018. https://doi.org/https://dx.doi.org/10.1155/2022/2038018](https://doi.org/https:/dx.doi.org/10.1155/2022/2038018) | | Sleep duration/ quality measured not reported |
| [Ha, J., Mehta, R. S., Cao, Y., Huang, T., Staller, K., & Chan, A. T. (2023). Assessment of Gastroesophageal Reflux Symptoms and Sleep Quality among Women in the Nurses' Health Study II. *JAMA Network Open, 6*(7), E2324240. https://doi.org/https://dx.doi.org/10.1001/jamanetworkopen.2023.24240](https://doi.org/https:/dx.doi.org/10.1001/jamanetworkopen.2023.24240) | | Sleep duration/ quality measured not reported |
| [Alzoubi, M. M., Al-Momani, S., Al-Mugheed, K., Owiedat, I., Shosha, G. M. A., Alabdullah, A. A. S., Alsenany, S. A., & Abdelaliem, S. M. F. (2024). Assessment of the quality of nursing work life and its related factors among critical care nurses. *Frontiers in Public Health, 12,* 1305686. https://doi.org/https://dx.doi.org/10.3389/fpubh.2024.1305686](https://doi.org/https:/dx.doi.org/10.3389/fpubh.2024.1305686) | | Sleep duration/ quality measured not reported |
| [Setyowati, L., Kurnia, A. D., Lestari, W., & Karuncharernpanit, S. (2023). Association between social media addiction and sleep quality among undergraduate nursing students: A cross-sectional study. *Frontiers of Nursing, 10*(2), 233-239. https://doi.org/https://dx.doi.org/10.2478/fon-2023-0025](https://doi.org/https:/dx.doi.org/10.2478/fon-2023-0025) | | Sleep duration/ quality measured not reported |
| [Mao, F., Wan, J., Sun, Y., Yang, B., Wang, Y., & Cao, F. (2024). Association between transition patterns of sleep problems and suicidal ideation in Chinese female nurses: A prospective study. *Journal of clinical psychology, 80*(2), 279-290. https://doi.org/https://dx.doi.org/10.1002/jclp.23612](https://doi.org/https:/dx.doi.org/10.1002/jclp.23612) | | Sleep duration/ quality measured not reported |
| [Vancampfort, D., & Mugisha, J. (2022). Associations between compassion fatigue, burnout and secondary traumatic stress with lifestyle factors in mental health nurses: A multicenter study from Uganda. *Archives of psychiatric nursing, 41*, 221-226. https://doi.org/https://dx.doi.org/10.1016/j.apnu.2022.08.009](https://doi.org/https:/dx.doi.org/10.1016/j.apnu.2022.08.009) | | Sleep duration/ quality measured not reported |
| [Amaral, K. V., Galdino, M. J. Q., & Martins, J. T. (2021). Burnout, daytime sleepiness and sleep quality among technical-level Nursing students. *Revista latino-americana de enfermagem, 29*, e3487. https://doi.org/https://dx.doi.org/10.1590/1518-8345.5180.3487](https://doi.org/https:/dx.doi.org/10.1590/1518-8345.5180.3487) | | Sleep duration/ quality measured not reported |
| [Giorgi, F., Mattei, A., Notarnicola, I., Petrucci, C., & Lancia, L. (2018). Can sleep quality and burnout affect the job performance of shift-work nurses? A hospital cross-sectional study. *Journal of Advanced Nursing, 74*(3), 698-708. https://doi.org/https://dx.doi.org/10.1111/jan.13484](https://doi.org/https:/dx.doi.org/10.1111/jan.13484) | | Sleep duration/ quality measured not reported |
| [Djupedal, I. L. R., Pallesen, S., Harris, A., Waage, S., Bjorvatn, B., & Vedaa, O. (2022). Changes in the Work Schedule of Nurses Related to the COVID-19 Pandemic and Its Relationship with Sleep and Turnover Intention. *International Journal of Environmental Research and Public Health, 19*(14), 8682. https://doi.org/https://dx.doi.org/10.3390/ijerph19148682](https://doi.org/https:/dx.doi.org/10.3390/ijerph19148682) | | Sleep duration/ quality measured not reported |
| [Wang, Y., Xie, J., Yang, F., Wu, S., Wang, H., Zhang, X., Liu, H., Deng, X., Xie, W., & Yu, S. (2015). Comorbidity of poor sleep and primary headaches among nursing staff in north China. *Journal of Headache and Pain, 16*(1), 88. https://doi.org/https://dx.doi.org/10.1186/s10194-015-0571-z](https://doi.org/https:/dx.doi.org/10.1186/s10194-015-0571-z) | | Sleep duration/ quality measured not reported |
| [Delafosse, J. Y., Léger, D., Quera-Salva, M. A., Samson, O., & Adrien, J. (2000). Comparative study of actigraphy and ambulatory polysomnography in the assessment of adaptation to night shift work in nurses. *Revue Neurologique, 156*(6-7), 641-645. https://www.scopus.com/inward/record.uri?eid=2-s2.0-0343090867&partnerID=40&md5=43851da5d22786f555491aa54c187eae](https://www.scopus.com/inward/record.uri?eid=2-s2.0-0343090867&partnerID=40&md5=43851da5d22786f555491aa54c187eae) | | Sleep duration/ quality measured not reported |
| [Ling, Q., Liu, Q., Yao, B., Qin, Y., Huang, F., & Liu, X. (2023). Current status and influencing factors of psychological distress among dental nursing staff during the new crown pneumonia epidemic. *Revista de Psiquiatria Clinica, 50*(2), 177-183. https://doi.org/https://dx.doi.org/10.15761/0101-60830000000569](https://doi.org/https:/dx.doi.org/10.15761/0101-60830000000569) | | Sleep duration/ quality measured not reported |
| [Yao, J., Zhou, X., Xu, D., Liu, T., Gui, Y., & Huang, Y. (2024). Current Status and Influencing Factors of Secondary Traumatic Stress in Emergency and Intensive Care nurses:A Cross-Sectional Analysis. *Psychology Research and Behavior Management, 17,* 567-576. https://doi.org/https://dx.doi.org/10.2147/PRBM.S444205](https://doi.org/https:/dx.doi.org/10.2147/PRBM.S444205) | | Sleep duration/ quality measured not reported |
| [Suzuki, K., Ohida, T., Kaneita, Y., Yokoyama, E., & Uchiyama, M. (2005). Daytime sleepiness, sleep habits and occupational accidents among hospital nurses. *Journal of Advanced Nursing, 52*(4), 445-453. https://doi.org/https://dx.doi.org/10.1111/j.1365-2648.2005.03610.x](https://doi.org/https:/dx.doi.org/10.1111/j.1365-2648.2005.03610.x) | | Sleep duration/ quality measured not reported |
| [Che, Y., Tang, R., Zhang, H., Yang, M., Geng, R., Zhuo, L., Hu, X., Zhou, Y., Wang, P., Zhan, S., & Li, B. (2024). Development of a prediction model for predicting the prevalence of nonalcoholic fatty liver disease in Chinese nurses: the first-year follow data of a web-based ambispective cohort study. *BMC Gastroenterology, 24*(1), 72. https://doi.org/https://dx.doi.org/10.1186/s12876-024-03121-1](https://doi.org/https:/dx.doi.org/10.1186/s12876-024-03121-1) | | Sleep duration/ quality measured not reported |
| [Sukumaran, S., & Achambattu, S. (2023). Effect of COVID-19 pandemic on sleep quality of frontline healthcare workers. *National Journal of Physiology, Pharmacy and Pharmacology, 13*(5), 1055-1059. https://doi.org/https://dx.doi.org/10.5455/njppp.2023.13.03132202326032023](https://doi.org/https:/dx.doi.org/10.5455/njppp.2023.13.03132202326032023) | | Sleep duration/ quality measured not reported |
| [Boonyagate, K., & Taneepanichskul, N. (2017). Effect of sleep quality on blood pressure and heart rate among shift nurses in a public hospital in Bangkok, Thailand. *Journal of Health Research, 31,* S51-S56. https://doi.org/https://dx.doi.org/10.14456/jhr.2017.67](https://doi.org/https:/dx.doi.org/10.14456/jhr.2017.67) | | Sleep duration/ quality measured not reported |
| [Deng, X., Liu, X., & Fang, R. (2020). Evaluation of the correlation between job stress and sleep quality in community nurses. *Medicine (United States), 99*(4), e18822. https://doi.org/https://dx.doi.org/10.1097/MD.0000000000018822](https://doi.org/https:/dx.doi.org/10.1097/MD.0000000000018822) | | Sleep duration/ quality measured not reported |
| [Cattani, A. N., da Silva, R. M., Beck, C. L. C., Miranda, F. M. D., de Lima Dalmolin, G., & Camponogara, S. (2021). Evening work, sleep quality and illness of nursing workers. *ACTA Paulista de Enfermagem, 34,* eAPE00843. https://doi.org/https://dx.doi.org/10.37689/acta-ape/2021AO00843](https://doi.org/https:/dx.doi.org/10.37689/acta-ape/2021AO00843) | | Sleep duration/ quality measured not reported |
| [Alamer, M., Shdaifat, E., Alshowkan, A., Eldeen, A. G., & Jamama, A. (2020). Exploring associations between internet addiction, depressive symptoms, and sleep disturbance among saudi nursing students. *Open Nursing Journal, 14*(1), 29-36. https://doi.org/https://dx.doi.org/10.2174/1874434602014010029](https://doi.org/https:/dx.doi.org/10.2174/1874434602014010029) | | Sleep duration/ quality measured not reported |
| [Alfonsi, V., Scarpelli, S., Gorgoni, M., Couyoumdjian, A., Rosiello, F., Sandroni, C., Corsi, R., Pietrantonio, F., & De Gennaro, L. (2023). Healthcare Workers after Two Years of COVID-19: The Consequences of the Pandemic on Psychological Health and Sleep among Nurses and Physicians. *International Journal of Environmental Research and Public Health, 20*(2), 1410. https://doi.org/https://dx.doi.org/10.3390/ijerph20021410](https://doi.org/https:/dx.doi.org/10.3390/ijerph20021410) | | Sleep duration/ quality measured not reported |
| [Patel, R., Kademane, A., & Dadheech, S. (2023). Implications for nursing practice on the association between sleep and health outcomes. Salud, *Ciencia y Tecnologia, 3.* https://doi.org/10.56294/saludcyt2023449](https://doi.org/10.56294/saludcyt2023449) | | Sleep duration/ quality measured not reported |
| [Vigoureux, T. F. D., & Lee, S. (2021). Individual and joint associations of daily sleep and stress with daily well-being in hospital nurses: An ecological momentary assessment and actigraphy study. *Journal of Behavioral Medicine, 44(*3), 320-332. https://doi.org/https://dx.doi.org/10.1007/s10865-021-00207-z](https://doi.org/https:/dx.doi.org/10.1007/s10865-021-00207-z) | | Sleep duration/ quality measured not reported |
| [Yan, J., Wu, C., Liu, Y., Zhang, H., He, C., Lin, Y., Li, Y., Zhang, Y., & Lang, H. (2023). Influencing factors of quality of life among front-line nurses who collected nucleic acid samples during COVID-19: a path analysis. *Frontiers in Public Health, 11.* https://doi.org/10.3389/fpubh.2023.1154725](https://doi.org/10.3389/fpubh.2023.1154725) | | Sleep duration/ quality measured not reported |
| [Wang, W., Ji, X., Guo, H.-Y., Tao, M., Jin, L., Chen, M., Yuan, H., & Peng, H. (2023). Investigation on sleep-related cognition of Chinese health care workers during the first wave of COVID-19 pandemic. *Frontiers in Psychiatry, 14,* 1019837. https://doi.org/https://dx.doi.org/10.3389/fpsyt.2023.1019837](https://doi.org/https:/dx.doi.org/10.3389/fpsyt.2023.1019837) | | Sleep duration/ quality measured not reported |
| [Ibrahim, N. K., Al-Bloushy, R. I., Sait, S. H., Al-Azhary, H. W., Al Bar, N. H., & Mirdad, G. A. (2016). Irritable bowel syndrome among nurses working in King Abdulaziz University Hospital, Jeddah, Saudi Arabia. *The Libyan journal of medicine, 11,* 30866. https://doi.org/https://dx.doi.org/10.3402/ljm.v11.30866](https://doi.org/https:/dx.doi.org/10.3402/ljm.v11.30866) | | Sleep duration/ quality measured not reported |
| [Huang, Z.-P., Huang, F., Wang, M.-J., Tang, C.-Z., Huang, J.-P., Ling, J., Li, S.-E., Wei, S.-Q., Lei, H.-H., Li, J.-J., & Lan, X. (2022). Lifestyle and Social Factors Exacerbated on the Prevalence of Mood Disorders and Functional Dyspepsia Among Neonatal Nurses in China. *Frontiers in Psychiatry, 13,* 905401. https://doi.org/https://dx.doi.org/10.3389/fpsyt.2022.905401](https://doi.org/https:/dx.doi.org/10.3389/fpsyt.2022.905401) | | Sleep duration/ quality measured not reported |
| [Juchnowicz, D., Tulej, Z., Karakula, K., & Rog, J. (2022). Lifestyle, sleep quality, and overall mental health of nursing students. *Current Problems of Psychiatry, 23*(1), 13-23. https://doi.org/https://dx.doi.org/10.2478/cpp-2022-0002](https://doi.org/https:/dx.doi.org/10.2478/cpp-2022-0002) | | Sleep duration/ quality measured not reported |
| [Blome, M., Johnson, M., Beck, M., & Jones, M. (2022). Obligations and Behaviors That Impact Sleep in Prelicensure Baccalaureate Nursing Students. *Nurse educator, 47*(1), E12-E17. https://doi.org/https://dx.doi.org/10.1097/NNE.0000000000001128](https://doi.org/https:/dx.doi.org/10.1097/NNE.0000000000001128) | | Sleep duration/ quality measured not reported |
| [Harma, M. I., Ilmarinen, J., Knauth, P., Rutenfranz, J., & Hanninen, O. (1988). Physical training intervention in female shift workers: I. The effects of intervention of fitness, fatigue, sleep, and psychosomatic symptoms. *Ergonomics, 31(*1), 39-50. https://doi.org/https://dx.doi.org/10.1080/00140138808966647](https://doi.org/https:/dx.doi.org/10.1080/00140138808966647) | | Sleep duration/ quality measured not reported |
| [Kafle, B., Tiwari, S., Pokhrel, A., Shrestha, R., Bagale, Y., & Pahari, N. (2024). Poor Quality of Sleep among Healthcare Workers in a Tertiary Care Centre. *Journal of the Nepal Medical Association, 62*(270), 118-120. https://doi.org/https://dx.doi.org/10.31729/jnma.8435](https://doi.org/https:/dx.doi.org/10.31729/jnma.8435) | | Sleep duration/ quality measured not reported |
| [Yoshimoto, T., Oka, H., Ochiai, H., Ishikawa, S., Kokaze, A., Muranaga, S., & Matsudaira, K. (2020). Presenteeism and associated factors among nursing personnel with low back pain: A cross-sectional study. *Journal of Pain Research, 13*, 2979-2986. https://doi.org/https://dx.doi.org/10.2147/JPR.S269529](https://doi.org/https:/dx.doi.org/10.2147/JPR.S269529) | | Sleep duration/ quality measured not reported |
| [Lu, G., Xiao, S., He, J., Xie, W., Ge, W., Meng, F., Yang, Y., Yu, S., & Liu, R. (2023). Prevalence of depression and its correlation with anxiety, headache and sleep disorders among medical staff in the Hainan Province of China. *Frontiers in Public Health, 11,* 1122626. https://doi.org/https://dx.doi.org/10.3389/fpubh.2023.1122626](https://doi.org/https:/dx.doi.org/10.3389/fpubh.2023.1122626) | | Sleep duration/ quality measured not reported |
| [Wang, P., Shen, X., Jiang, Y., Wu, L., Shen, J., Nie, X., Chen, W., & Liu, J. (2022). Psychological and sleep disturbances among first-line, second-line, and at home medical staff during the COVID-19 pandemic in Shanghai, China. *Frontiers in Public Health, 10,* 1006610. https://doi.org/https://dx.doi.org/10.3389/fpubh.2022.1006610](https://doi.org/https:/dx.doi.org/10.3389/fpubh.2022.1006610) | | Sleep duration/ quality measured not reported |
| [Al Maqbali, M., & Al Khadhuri, J. (2021). Psychological impact of the coronavirus 2019 (COVID-19) pandemic on nurses. *Japan journal of nursing science : JJNS,* e12417. https://doi.org/https://dx.doi.org/10.1111/jjns.12417](https://doi.org/https:/dx.doi.org/10.1111/jjns.12417) | | Sleep duration/ quality measured not reported |
| [Kim, H. J., & Abraham, I. (2015). Psychometric comparison of single-item, short, and comprehensive depression screening measures in Korean young adults. *International Journal of Nursing Studies, 56*, 71-80. https://doi.org/10.1016/j.ijnurstu.2015.12.003](https://doi.org/10.1016/j.ijnurstu.2015.12.003) | | Sleep duration/ quality measured not reported |
| [Kim, H. J., & Abraham, I. (2015). Psychometric comparison of single-item, short, and comprehensive depression screening measures in Korean young adults. *International Journal of Nursing Studies, 56,* 71-80. https://doi.org/10.1016/j.ijnurstu.2015.12.003](https://doi.org/10.1016/j.ijnurstu.2015.12.003) | | Sleep duration/ quality measured not reported |
| [Lonnqvist, K., Sinervo, T., Kaihlanen, A.-M., Vehvilainen-Julkunen, K., & Elovainio, M. (2023). Psychosocial work characteristics and sleep quality among early career registered nurses: a cross-sectional latent profile analysis. *BMC health services research, 23*(1), 1020. https://doi.org/https://dx.doi.org/10.1186/s12913-023-09949-9](https://doi.org/https:/dx.doi.org/10.1186/s12913-023-09949-9) | | Sleep duration/ quality measured not reported |
| [Cecere, L., de Novellis, S., Gravante, A., Petrillo, G., Pisani, L., Terrenato, I., Ivziku, D., Latina, R., & Gravante, F. (2023). Quality of life of critical care nurses and impact on anxiety, depression, stress, burnout and sleep quality: A cross-sectional study. *Intensive & critical care nursing, 79*, 103494. https://doi.org/https://dx.doi.org/10.1016/j.iccn.2023.103494](https://doi.org/https:/dx.doi.org/10.1016/j.iccn.2023.103494) | | Sleep duration/ quality measured not reported |
| [Olawale, O., Taiwo, O., & Hesham, A. (2017). Quality of sleep and well-being of health workers in Najran, Saudi Arabia. *Indian Journal of Psychiatry, 59*(3), 347-351. https://doi.org/https://dx.doi.org/10.4103/psychiatry.IndianJPsychiatry_241_16](https://doi.org/https:/dx.doi.org/10.4103/psychiatry.IndianJPsychiatry_241_16) | | Sleep duration/ quality measured not reported |
| [Sutarti, T., Wati, D. R., Abdul, M., & Anwar, M. (2018). Relationship of sleep quality with student learning motivation in nursing academy 17 of karanganyar. *Indian Journal of Public Health Research and Development, 9*(12), 1410-1413. https://doi.org/https://dx.doi.org/10.5958/0976-5506.2018.02051.X](https://doi.org/https:/dx.doi.org/10.5958/0976-5506.2018.02051.X) | | Sleep duration/ quality measured not reported |
| [Cattani, A. N., da Silva, R. M., Beck, C. L. C., Miranda, F. M. D., Dalmolin, G. L., & Camponogara, S. (2022). REPERCUSSIONS OF NIGHT SHIFT WORK ON NURSING PROFESSIONALS' HEALTH AND SLEEP QUALITY. *Texto e Contexto Enfermagem, 31*, e20210346. https://doi.org/https://dx.doi.org/10.1590/1980-265X-TCE-2021-0346EN](https://doi.org/https:/dx.doi.org/10.1590/1980-265X-TCE-2021-0346EN) | | Sleep duration/ quality measured not reported |
| [Di Simone, E., Fabbian, F., Giannetta, N., Dionisi, S., Renzi, E., Cappadona, R., Di Muzio, M., & Manfredini, R. (2020). Risk of medication errors and nurses' quality of sleep: A national cross-sectional web survey study. *European Review for Medical and Pharmacological Sciences, 24*(12), 7058-7062. https://doi.org/https://dx.doi.org/10.26355/eurrev_202006_21699](https://doi.org/https:/dx.doi.org/10.26355/eurrev_202006_21699) | | Sleep duration/ quality measured not reported |
| [Bani-Issa, W., Radwan, H., Al Marzooq, F., Al Awar, S., Al-Shujairi, A. M., Samsudin, A. R., Khasawneh, W., & Albluwi, N. (2020). Salivary cortisol, subjective stress and quality of sleep among female healthcare professionals. *Journal of Multidisciplinary Healthcare, 13*, 125-140. https://doi.org/https://dx.doi.org/10.2147/JMDH.S229396](https://doi.org/https:/dx.doi.org/10.2147/JMDH.S229396) | | Sleep duration/ quality measured not reported |
| [Chung, M.-H., Liu, W.-I., Lee, H.-L., & Hsu, N. (2013). Selected neurophysiological, psychological, and behavioral influences on subjective sleep quality in nurses: a structure equation model. *PLoS ONE, 8*(11), e79529. https://doi.org/https://dx.doi.org/10.1371/journal.pone.0079529](https://doi.org/https:/dx.doi.org/10.1371/journal.pone.0079529) | | Sleep duration/ quality measured not reported |
| [Cheng, H. H., Chung, S. C., & Liao, W. C. (2011). Sleep and Dietary Patterns Among Nurses Working in different Shifts. *Journal of Nursing and Healthcare Research, 7*(4), 295-304. https://www.scopus.com/inward/record.uri?eid=2-s2.0-84865163323&partnerID=40&md5=828f95d326b7e7d95600b0d9f82a423d](https://www.scopus.com/inward/record.uri?eid=2-s2.0-84865163323&partnerID=40&md5=828f95d326b7e7d95600b0d9f82a423d) | | Sleep duration/ quality measured not reported |
| [Da Silva, R. M., Lenz, F. C. D., Schlotfeldt, N. F., de Morais, K. C. P., Beck, C. L. C., de Martino, M. M. F., Borges, E. M. D. N., & Zeitoune, R. C. G. (2022). SLEEP ASSESSMENT AND ASSOCIATED FACTORS IN HOSPITAL NURSING WORKERS. *Texto e Contexto Enfermagem, 31*, e20220277. https://doi.org/https://dx.doi.org/10.1590/1980-265X-TCE-2022-0277en](https://doi.org/https:/dx.doi.org/10.1590/1980-265X-TCE-2022-0277en) | | Sleep duration/ quality measured not reported |
| [Nag, K., Datta, A., Karmakar, N., Chakraborty, T., & Bhattacharjee, P. (2019). Sleep disturbance and its effect on work performance of staffs following shifting duties: A cross-sectional study in a medical college and hospital of Tripura. *Medical Journal of Dr. D.Y. Patil Vidyapeeth*, 211-216. https://doi.org/10.4103/mjdrdypu.mjdrdypu_116_18](https://doi.org/10.4103/mjdrdypu.mjdrdypu_116_18) | | Sleep duration/ quality measured not reported |
| [Katsifaraki, M., Nilsen, K. B., Christensen, J. O., Waersted, M., Knardahl, S., Bjorvatn, B., Harma, M., & Matre, D. (2019). Sleep duration mediates abdominal and lower-extremity pain after night work in nurses. *International Archives of Occupational and Environmental Health, 92(*3), 415-422. https://doi.org/https://dx.doi.org/10.1007/s00420-018-1373-9](https://doi.org/https:/dx.doi.org/10.1007/s00420-018-1373-9) | | Sleep duration/ quality measured not reported |
| [Kolo, E. S., Ahmed, A. O., Hamisu, A., Ajiya, A., & Akhiwu, B. I. (2017). Sleep health of healthcare workers in Kano, Nigeria. *Nigerian journal of clinical practice, 20*(4), 479-483. https://doi.org/https://dx.doi.org/10.4103/1119-3077.204378](https://doi.org/https:/dx.doi.org/10.4103/1119-3077.204378) | | Sleep duration/ quality measured not reported |
| [Forthun, I., Waage, S., Pallesen, S., Moen, B. E., & Bjorvatn, B. (2022). Sleep medication and melatonin use among Norwegian nurses - A cross-sectional study. *Nursing open, 9*(1), 233-244. https://doi.org/https://dx.doi.org/10.1002/nop2.1057](https://doi.org/https:/dx.doi.org/10.1002/nop2.1057) | | Sleep duration/ quality measured not reported |
| [Ghalichi, L., Pournik, O., Ghaffari, M., & Vingard, E. (2013). Sleep quality among health care workers. *Archives of Iranian Medicine, 16*(2), 100-103. http://www.aimjournal.ir/pdffiles/34_2013162_0010.pdfhttp://ovidsp.ovid.com/ovidweb.cgi?T=JS&PAGE=reference&D=emed14&NEWS=N&AN=368417921](http://www.aimjournal.ir/pdffiles/34_2013162_0010.pdfhttp:/ovidsp.ovid.com/ovidweb.cgi?T=JS&PAGE=reference&D=emed14&NEWS=N&AN=368417921) | | Sleep duration/ quality measured not reported |
| [Ravi, A., Sivapriya, K. R. S., George, N., Britto, R., Parthiban, A., & Anukruthi, N. (2022). Sleep Quality among Healthcare Professionals in a Tertiary Care Hospital. *National Journal of Community Medicine, 13*(4), 213-218. https://doi.org/https://dx.doi.org/10.55489/njcm.1342022434](https://doi.org/https:/dx.doi.org/10.55489/njcm.1342022434) | | Sleep duration/ quality measured not reported |
| [Yazdi, Z., Sadeghniiat-Haghighi, K., Javadi, A. R., & Rikhtegar, G. (2014). Sleep quality and insomnia in nurses with different circadian chronotypes: morningness and eveningness orientation. *Work (Reading, Mass.), 47(*4), 561-567. https://doi.org/https://dx.doi.org/10.3233/WOR-131664](https://doi.org/https:/dx.doi.org/10.3233/WOR-131664) | | Sleep duration/ quality measured not reported |
| [Chung, M.-H., Chang, F.-M., Yang, C. C. H., Kuo, T. B. J., & Hsu, N. (2009). Sleep quality and morningness-eveningness of shift nurses. *Journal of Clinical Nursing, 18(*2), 279-284. https://doi.org/https://dx.doi.org/10.1111/j.1365-2702.2007.02160.x](https://doi.org/https:/dx.doi.org/10.1111/j.1365-2702.2007.02160.x) | | Sleep duration/ quality measured not reported |
| [Yilmaz, M., Kirac, Y., & Sahin, M. K. (2021). Sleep quality and related factors in a sample of Turkish healthcare workers during the COVID-19 pandemic: A cross-sectional study. *International Journal of Clinical Practice, 75*(11), e14813. https://doi.org/https://dx.doi.org/10.1111/ijcp.14813](https://doi.org/https:/dx.doi.org/10.1111/ijcp.14813) | | Sleep duration/ quality measured not reported |
| [Azizollah, A., Kord, F., & Hossein, A. (2015). Sleep quality in nurses working in different shifts. *Der Pharmacia Lettre, 7*(9), 305-309. http://scholarsresearchlibrary.com/dpl-vol7-iss9/DPL-2015-7-9-305-309.pdfhttp://ovidsp.ovid.com/ovidweb.cgi?T=JS&PAGE=reference&D=emed16&NEWS=N&AN=606476002](http://scholarsresearchlibrary.com/dpl-vol7-iss9/DPL-2015-7-9-305-309.pdfhttp:/ovidsp.ovid.com/ovidweb.cgi?T=JS&PAGE=reference&D=emed16&NEWS=N&AN=606476002) | | Sleep duration/ quality measured not reported |
| [Cheng, S.-Y., Lin, P.-C., Chang, Y.-K., Lin, Y.-K., Lee, P.-H., & Chen, S.-R. (2019). Sleep quality mediates the relationship between work-family conflicts and the self-perceived health status among hospital nurses. *Journal of Nursing Management, 27*(2), 381-387. https://doi.org/https://dx.doi.org/10.1111/jonm.12694](https://doi.org/https:/dx.doi.org/10.1111/jonm.12694) | | Sleep duration/ quality measured not reported |
| [Guerra, P. C., Oliveira, N. F., Terreri, M. T., & Len, C. A. (2016). Sleep, quality of life and mood of nursing professionals of pediatric intensive care units. *Revista da Escola de Enfermagem da U S P, 50*(2), 279-285. https://doi.org/https://dx.doi.org/10.1590/S0080-623420160000200014](https://doi.org/https:/dx.doi.org/10.1590/S0080-623420160000200014) | | Sleep duration/ quality measured not reported |
| [Geiger-Brown, J., Rogers, V. E., Trinkoff, A. M., Kane, R. L., Bausell, R. B., & Scharf, S. M. (2012). Sleep, sleepiness, fatigue, and performance of 12-hour-shift nurses. *Chronobiology International, 29*(2), 211-219. https://doi.org/https://dx.doi.org/10.3109/07420528.2011.645752](https://doi.org/https:/dx.doi.org/10.3109/07420528.2011.645752) | | Sleep duration/ quality measured not reported |
| [Kalal, N., Vel, N. S., Angmo, S., Choyal, S., Bishnoi, S., Dhaka, S., Rulaniya, S., & Banswal, S. (2023). Smartphone addiction and its impact on quality of sleep and academic performance among nursing students. Institutional based cross-sectional study in Western Rajasthan (India). *Investigacion y Educacion en Enfermeria, 41*(2), e11. https://doi.org/https://dx.doi.org/10.17533/udea.iee.v41n2e11](https://doi.org/https:/dx.doi.org/10.17533/udea.iee.v41n2e11) | | Sleep duration/ quality measured not reported |
| [da Silva, K. K. M., de Martino, M. M. F., Bezerra, C. M. B., de Souza, Â. M. L., da Silva, D. M., & Nunes, J. T. (2020). Stress and quality of sleep in undergraduate nursing students. *Revista Brasileira de Enfermagem, 73.* https://doi.org/10.1590/0034-7167-2018-0227](https://doi.org/10.1590/0034-7167-2018-0227) | | Sleep duration/ quality measured not reported |
| [Prabhavathi, K., Kumar, G. T., Hemamalini, R. V., Poornima, K. N., & Saravanan, A. (2018). Study of psychological predictors and sleep quality in different grades of premenstrual syndrome. *National Journal of Physiology, Pharmacy and Pharmacology, 8*(3), 353-357. https://doi.org/https://dx.doi.org/10.5455/njppp.2017.7.0935427102017](https://doi.org/https:/dx.doi.org/10.5455/njppp.2017.7.0935427102017) | | Sleep duration/ quality measured not reported |
| [Wang, J., Zhang, X., Yang, B., Li, J., Li, Y., Chen, Q., Wu, L., & Cao, F. (2020). Suicidal ideation among nurses: Unique and cumulative effects of different subtypes of sleep problems. *Journal of Affective Disorders, 276,* 600-607. https://doi.org/https://dx.doi.org/10.1016/j.jad.2020.07.095](https://doi.org/https:/dx.doi.org/10.1016/j.jad.2020.07.095) | | Sleep duration/ quality measured not reported |
| [Bjorvatn, B., Axelsson, J., Pallesen, S., Waage, S., Vedaa, Ø., Blytt, K. M., Buchvold, H. V., Moen, B. E., & Thun, E. (2020). The Association Between Shift Work and Immunological Biomarkers in Nurses. *Frontiers in Public Health, 8*. https://doi.org/10.3389/fpubh.2020.00415](https://doi.org/10.3389/fpubh.2020.00415) | | Sleep duration/ quality measured not reported |
| [Katsifaraki, M., Nilsen, K. B., Waersted, M., Knardahl, S., Lie, J.-A. S., Bjorvatn, B., Harma, M., & Matre, D. (2018). The association of sleepiness, insomnia, sleep disturbance and pain: A study amongst shiftworking nurses. *Sleep and Biological Rhythms, 16(*1), 133-140. https://doi.org/https://dx.doi.org/10.1007/s41105-017-0135-5](https://doi.org/https:/dx.doi.org/10.1007/s41105-017-0135-5) | | Sleep duration/ quality measured not reported |
| [Kurumatani, N., Koda, S., Nakagiri, S., Hisashige, A., Sakai, K., Saito, Y., Aoyama, H., Dejima, M., & Moriyama, T. (1994). The effects of frequently rotating shiftwork on sleep and the family life of hospital nurses. *Ergonomics, 37*(6), 995-1007. http://ovidsp.ovid.com/ovidweb.cgi?T=JS&PAGE=reference&D=emed5&NEWS=N&AN=24169544](http://ovidsp.ovid.com/ovidweb.cgi?T=JS&PAGE=reference&D=emed5&NEWS=N&AN=24169544) | | Sleep duration/ quality measured not reported |
| [Uekata, S., Kato, C., Nagaura, Y., Eto, H., & Kondo, H. (2019). The impact of rotating work schedules, chronotype, and restless legs syndrome/Willis-Ekbom disease on sleep quality among female hospital nurses and midwives: A cross-sectional survey. *International Journal of Nursing Studies, 95*, 103-112. https://doi.org/https://dx.doi.org/10.1016/j.ijnurstu.2019.04.013](https://doi.org/https:/dx.doi.org/10.1016/j.ijnurstu.2019.04.013) | | Sleep duration/ quality measured not reported |
| [Ljevak, I., Vasilj, I., Curlin, M., Saravanja, N., Mestrovi, T., Simi, J., & Neuberg, M. (2020). The impact of shift work on psychosocial functioning and quality of life among hospital-employed nurses: A cross-sectional comparative study. *Psychiatria Danubina, 32,* 262-268. https://doi.org/https://dx.doi.org/10.24869/psyd.2020.262](https://doi.org/https:/dx.doi.org/10.24869/psyd.2020.262) | | Sleep duration/ quality measured not reported |
| [Najafzadeh, M., Amini, K., Sadeghniiat-Haghighi, K., & Kamali, K. (2022). The impact of work schedules, workplace bullying and some demographic characteristics on nurses' sleep quality in Iran. *Sleep Science, 15*(1), 62-67. https://doi.org/https://dx.doi.org/10.5935/1984-0063.20210029](https://doi.org/https:/dx.doi.org/10.5935/1984-0063.20210029) | | Sleep duration/ quality measured not reported |
| [Yun, M., & Beehr, T. (2024). When experiencing nice interactions at work: Good sleep quality via well-being. *Stress and Health*. https://doi.org/https://dx.doi.org/10.1002/smi.3390](https://doi.org/https:/dx.doi.org/10.1002/smi.3390) | | Sleep duration/ quality measured not reported |
| [Zhang, Y., El Ghaziri, M., Dugan, A. G., & Castro, M. E. (2019). Work and Health Correlates of Sleep Quantity and Quality Among Correctional Nurses. *Journal of forensic nursing, 15(*1), 42-51. https://doi.org/https://dx.doi.org/10.1097/JFN.0000000000000229](https://doi.org/https:/dx.doi.org/10.1097/JFN.0000000000000229) | | Sleep duration/ quality measured not reported |
| [Rabei, S., Mourad, G., & Hamed, A. E. D. (2020). Work stress and sleep disturbances among internship nursing students. *Middle East Current Psychiatry, 27(*1). https://doi.org/10.1186/s43045-020-00032-1](https://doi.org/10.1186/s43045-020-00032-1) | | Sleep duration/ quality measured not reported |
| [Alabdullatif, E. A., Alameri, R. A., & Al-Dossary, R. N. M. (2024). Relationship Between Quality of Sleep and Psychological Well-being Among Frontline Nurses During the COVID-19 Pandemic in Saudi Arabia. *Medical archives (Sarajevo, Bosnia and Herzegovina), 78*(1), 22-28. https://doi.org/https://dx.doi.org/10.5455/medarh.2024.78.22-28](https://doi.org/https:/dx.doi.org/10.5455/medarh.2024.78.22-28) | | Sleep duration/ quality measured not reported |
| [Surani, S. R., Majid, H., Booparaju, S., Surani, A., Guntupalli, B., & Subramanian, S. (2009). To assess sleep and vigilance among nurses in an intensive care unit setting. *Chest, 136*(4). http://meeting.chestpubs.org/cgi/content/abstract/136/4/58S-fhttp://ovidsp.ovid.com/ovidweb.cgi?T=JS&PAGE=reference&D=emed11&NEWS=N&AN=70203967](http://meeting.chestpubs.org/cgi/content/abstract/136/4/58S-fhttp:/ovidsp.ovid.com/ovidweb.cgi?T=JS&PAGE=reference&D=emed11&NEWS=N&AN=70203967) | | Sleep duration/ quality measured not reported |
| [Tian, Y., Yue, Y., Liao, X., Wang, J., Ye, M., Liu, Y., Li, Y., & Zhou, J. (2021). Mental health service use and its associated factors among nurses in China: A cross-sectional survey. *PeerJ, 9,* e11181. https://doi.org/https://dx.doi.org/10.7717/peerj.11181](https://doi.org/https:/dx.doi.org/10.7717/peerj.11181) | | Sleep duration/ quality measured not reported |
| [Dietch, J. R., Taylor, D. J., Pruiksma, K., Wardle-Pinkston, S., Slavish, D. C., Messman, B., Estevez, R., Ruggero, C. J., & Kelly, K. (2021). The Nightmare Disorder Index: Development and initial validation in a sample of nurses. *Sleep, 44*(5), zsaa254. https://doi.org/https://dx.doi.org/10.1093/sleep/zsaa254](https://doi.org/https:/dx.doi.org/10.1093/sleep/zsaa254) | | Sleep duration/ quality measured not reported |
| West, S. H., Ahern, M., Byrnes, M., & Kwanten, L. (2007). New graduate nurses adaptation to shift work: can we help? *Collegian (Royal College of Nursing, Australia), 14*(1), 23-30. https://doi.org/10.1016/S1322-7696(08)60544-2 | | Sleep duration/ quality measured not reported |
| [Yeom, J., & Yeom, I. (2023). Mediating effects of fatigue on the relationship between sleep quality and the quality of life of shift-working nurses. *Chronobiology International, 40*(4), 450-457. https://doi.org/https://dx.doi.org/10.1080/07420528.2023.2186130](https://doi.org/https:/dx.doi.org/10.1080/07420528.2023.2186130) | | Sleep duration/ quality measured not reported |
| [Uchiyama, M. (2020). A composite measure of sleep health is associated with symptoms of depression among Japanese female hospital nurses. *Comprehensive Psychiatry, 97*, 152151. https://doi.org/https://dx.doi.org/10.1016/j.comppsych.2019.152151](https://doi.org/https:/dx.doi.org/10.1016/j.comppsych.2019.152151) | | Sleep duration/ quality not reported |
| [Wu, C., Ge, Y., Xu, C., Zhang, X., & Lang, H. (2020). A correlation study of emergency department nurses' fatigue, perceived stress, social support and self-efficacy in grade III A hospitals of Xi'an. *Medicine (United States)*, 99(32), E21052. https://doi.org/https://dx.doi.org/10.1097/MD.0000000000021052](https://doi.org/https:/dx.doi.org/10.1097/MD.0000000000021052) | | Sleep duration/ quality not reported |
| [Kobayashi, K., & Tadaka, E. (2022). A Cross-Sectional Study of Mental Health, Lifestyle, and Anxiety in Japanese Nursing Students by Year of College. *Healthcare (Switzerland), 10*(10). https://doi.org/10.3390/healthcare10101870](https://doi.org/10.3390/healthcare10101870) | | Sleep duration/ quality not reported |
| [Vedaa, O., Harris, A., Waage, S., Bjorvatn, B., Thun, E., Buchvold, H. V., Djupedal, I. L. R., & Pallesen, S. (2020). A longitudinal study on the association between quick returns and occupational accidents. *Scandinavian Journal of Work, Environment & Health, 46*(6), 645-649. https://doi.org/https://dx.doi.org/10.5271/sjweh.3906](https://doi.org/https:/dx.doi.org/10.5271/sjweh.3906) | | Sleep duration/ quality not reported |
| [Van Woerkom, M. (2021). A quasi-experimental study into the effects of naps and therapy glasses on fatigue and well-being. *Journal of Nursing Management, 29*(3), 562-571. https://doi.org/https://dx.doi.org/10.1111/jonm.13172](https://doi.org/https:/dx.doi.org/10.1111/jonm.13172) | | Sleep duration/ quality not reported |
| [Rutledge, T., Stucky, E., Dollarhide, A., Shively, M., Jain, S., Wolfson, T., Weinger, M. B., & Dresselhaus, T. (2009). A Real-Time Assessment of Work Stress in Physicians and Nurses. *Health Psychology, 28*(2), 194-200. https://doi.org/https://dx.doi.org/10.1037/a0013145](https://doi.org/https:/dx.doi.org/10.1037/a0013145) | | Sleep duration/ quality not reported |
| [Tsai, S. M., Wong, R. H., Hwang, S. L., Yang, H. C., Lin, Y. C., & Liao, W. C. (2014). A study relating shift work to the parameters of metabolic syndrome among nurses in a medical center. *Taiwan Journal of Public Health, 33*(2), 119-130. https://doi.org/10.6288/TJPH201433102110](https://doi.org/10.6288/TJPH201433102110) | | Sleep duration/ quality not reported |
| [Vigoureux, T. F. D., Mu, C. X., Mason, T. M., Gonzalez, B. D., & Lee, S. (2022). Adapting Research to a Global Pandemic: A Fully Remote Actigraphy and Ecological Momentary Assessment Study. *Nursing research, 71*(2), 119-127. https://doi.org/https://dx.doi.org/10.1097/NNR.0000000000000561](https://doi.org/https:/dx.doi.org/10.1097/NNR.0000000000000561) | | Sleep duration/ quality not reported |
| [Abbas Ahadiat, S. A., Hosseinian, Z., & Kouchaki, E. (2022). An epidemiological study of migraine headaches among the staff of Shahid Beheshti Hospital in Kashan, Iran, and related factors. *medRxiv.* https://doi.org/https://dx.doi.org/10.1101/2022.07.21.22277660](https://doi.org/https:/dx.doi.org/10.1101/2022.07.21.22277660) | | Sleep duration/ quality not reported |
| [Roshangar, M., Mollazehi, K., & Poorkahkhaei, M. (2016). Analyzing the sleep quality of nurses employed in special care units of Chabahar's Imam Ali teaching hospital in 2016. *International Journal of Pharmacy and Technology, 8*(3), 18796-18801. http://www.ijptonline.com/wp-content/uploads/2016/10/18796-18801.pdfhttp://ovidsp.ovid.com/ovidweb.cgi?T=JS&PAGE=reference&D=emed17&NEWS=N&AN=612952805](http://www.ijptonline.com/wp-content/uploads/2016/10/18796-18801.pdfhttp:/ovidsp.ovid.com/ovidweb.cgi?T=JS&PAGE=reference&D=emed17&NEWS=N&AN=612952805) | | Sleep duration/ quality not reported |
| [Lijuan, Q., Lin, Y., Juntao, L., Kun, Y., & Hongjuan, C. (2023). Anxiety, depression, and stress among nurses under the stress of flooding and the COVID-19 pandemic. *International nursing review, 70*(4), 535-543. https://doi.org/https://dx.doi.org/10.1111/inr.12874](https://doi.org/https:/dx.doi.org/10.1111/inr.12874) | | Sleep duration/ quality not reported |
| [Kruthika, K., Kotabal, R., Vedavati, B. I., & Jayashree, K. S. (2023). Assessment of Sleep Quality and Sleep Hygiene among Nursing officers during COVID pandemic in a Tertiary Care Hospital in a Southern District of Karnataka, India. *Indian Journal of Public Health Research and Development, 14*(2), 119-126. https://doi.org/https://dx.doi.org/10.37506/ijphrd.v14i2.19082](https://doi.org/https:/dx.doi.org/10.37506/ijphrd.v14i2.19082) | | Sleep duration/ quality not reported |
| [Augner, C. (2011). Associations of subjective sleep quality with depression score, anxiety, physical symptoms and sleep onset latency in students. *Central European journal of public health, 19*(2), 115-117. http://ovidsp.ovid.com/ovidweb.cgi?T=JS&PAGE=reference&D=emed12&NEWS=N&AN=362272845](http://ovidsp.ovid.com/ovidweb.cgi?T=JS&PAGE=reference&D=emed12&NEWS=N&AN=362272845) | | Sleep duration/ quality not reported |
| [Alameri, F., Aldaheri, N., Almesmari, S., Basaloum, M., Albeshr, N. A., Simsekler, M. C. E., Ugwuoke, N. V., Dalkilinc, M., Al Qubaisi, M., Campos, L. A., Almahmeed, W., Alefishat, E., Al Tunaiji, H., & Baltatu, O. C. (2022). Burnout and Cardiovascular Risk in Healthcare Professionals During the COVID-19 Pandemic. *Frontiers in Psychiatry, 13,* 867233. https://doi.org/https://dx.doi.org/10.3389/fpsyt.2022.867233](https://doi.org/https:/dx.doi.org/10.3389/fpsyt.2022.867233) | | Sleep duration/ quality not reported |
| [Sawyer, A. M., Saconi, B., Lyons, M. M., Lang-Gallagher, R., Renz, S. M., Watach, A. J., McPhillips, M. V., & Rosen, I. M. (2022). Case-based, asynchronous sleep education outcomes among primary care nurse practitioner students. *Journal of Clinical Sleep Medicine, 18*(10), 2367-2376. https://doi.org/https://dx.doi.org/10.5664/jcsm.10112](https://doi.org/https:/dx.doi.org/10.5664/jcsm.10112) | | Sleep duration/ quality not reported |
| [Geiger-Brown, J., Sagherian, K., Zhu, S., Wieroniey, M. A., Blair, L., Warren, J., Hinds, P. S., & Szeles, R. (2016). CE: Original Research: Napping on the Night Shift: A Two-Hospital Implementation Project. *The American journal of nursing, 116*(5), 26-33. https://doi.org/https://dx.doi.org/10.1097/01.NAJ.0000482953.88608.80](https://doi.org/https:/dx.doi.org/10.1097/01.NAJ.0000482953.88608.80) | | Sleep duration/ quality not reported |
| [Wrobel-Knybel, P., Flis, M., Rog, J., Jalal, B., Wolkowski, L., & Karakula-Juchnowicz, H. (2022). Characteristics of Sleep Paralysis and Its Association with Anxiety Symptoms, Perceived Stress, PTSD, and Other Variables Related to Lifestyle in Selected High Stress Exposed Professions. *International Journal of Environmental Research and Public Health, 19*(13), 7821. https://doi.org/https://dx.doi.org/10.3390/ijerph19137821](https://doi.org/https:/dx.doi.org/10.3390/ijerph19137821) | | Sleep duration/ quality not reported |
| [Yamaguchi, S., Winwood, P. C., & Yano, R. (2023). Chronic fatigue and recovery among nurses working two-shift and three-shift rotations. *Collegian, 30*(6), 786-794. https://doi.org/https://dx.doi.org/10.1016/j.colegn.2023.06.001](https://doi.org/https:/dx.doi.org/10.1016/j.colegn.2023.06.001) | | Sleep duration/ quality not reported |
| [Boivin, D. B., & James, F. O. (2002). Circadian adaptation to night-shift work by judicious light and darkness exposure*. Journal of Biological Rhythms, 17(*6), 556-567. https://doi.org/https://dx.doi.org/10.1177/0748730402238238](https://doi.org/https:/dx.doi.org/10.1177/0748730402238238) | | Sleep duration/ quality not reported |
| [Kant, R., Yadav, P., Kishore, S., Kumar, R., & Bairwa, M. (2020). Circadian dysynchrony among nurses performing shift work at a tertiary care teaching hospital: A preliminary study. International J*ournal of Physiology, Pathophysiology and Pharmacology, 12*(6), 166-172. http://www.ijppp.org/files/ijppp0117939.pdfhttp://ovidsp.ovid.com/ovidweb.cgi?T=JS&PAGE=reference&D=emed21&NEWS=N&AN=2006050498](http://www.ijppp.org/files/ijppp0117939.pdfhttp:/ovidsp.ovid.com/ovidweb.cgi?T=JS&PAGE=reference&D=emed21&NEWS=N&AN=2006050498) | | Sleep duration/ quality not reported |
| [Celik, A. S., & Sonmez, T. (2021). COMPARISON of the FEAR LEVELS and SLEEP PROBLEMS of NURSES WORKING in WARDS WHERE PATIENTS with and without COVID-19 ARE HOSPITALIZED: A STUDY from Turkey. *Psychiatria Danubina, 33,* 357-363. https://www.psychiatria-danubina.com/UserDocsImages/pdf/dnb_vol33_noSuppl%2013/dnb_vol33_noSuppl%2013_357.pdfhttp://ovidsp.ovid.com/ovidweb.cgi?T=JS&PAGE=reference&D=emed23&NEWS=N&AN=2016942215](https://www.psychiatria-danubina.com/UserDocsImages/pdf/dnb_vol33_noSuppl%2013/dnb_vol33_noSuppl%2013_357.pdfhttp:/ovidsp.ovid.com/ovidweb.cgi?T=JS&PAGE=reference&D=emed23&NEWS=N&AN=2016942215) | | Sleep duration/ quality not reported |
| [Zhang, Y., Punnett, L., McEnany, G. P., & Gore, R. (2016). Contributing influences of work environment on sleep quantity and quality of nursing assistants in long-term care facilities: A cross-sectional study. *Geriatric nursing (New York, N.Y.), 37*(1), 13-18. https://doi.org/https://dx.doi.org/10.1016/j.gerinurse.2015.08.010](https://doi.org/https:/dx.doi.org/10.1016/j.gerinurse.2015.08.010) | | Sleep duration/ quality not reported |
| [Zhang, S.-e., Yang, L.-b., Zhao, C.-x., Shi, Y., Wang, H.-n., Zhao, X., Wang, X.-h., Sun, T., & Cao, D.-p. (2021). Contribution of character strengths to psychology stress, sleep quality, and subjective health status in a sample of Chinese nurses. *Frontiers in Psychology, 12.* https://doi.org/https://dx.doi.org/10.3389/fpsyg.2021.631459](https://doi.org/https:/dx.doi.org/10.3389/fpsyg.2021.631459) | | Sleep duration/ quality not reported |
| [Ciray, N., Adaici, M., & Ozcan, A. (2023). COVID-19 Pandemisinde Hemsirelik Ogrencilerinde Kronotip, Sosyal Jetlag, Dikkat Kontrolu ve Akademik Basari Iliskisi. *Journal of Turkish Sleep Medicine, 10*(3), 229-234. https://doi.org/https://dx.doi.org/10.4274/tjsm.galenos.2023.37980](https://doi.org/https:/dx.doi.org/10.4274/tjsm.galenos.2023.37980) | | Sleep duration/ quality not reported |
| [Reicherts, P., Zerbini, G., Halms, T., Strasser, M., Papazova, I., Hasan, A., & Kunz, M. (2022). COVID-19 related psychological burden and potential benefits of vaccination - Data from a repeated cross-sectional survey in healthcare workers. *Psychiatry Research Communications, 2*(3), 100054. https://doi.org/https://dx.doi.org/10.1016/j.psycom.2022.100054](https://doi.org/https:/dx.doi.org/10.1016/j.psycom.2022.100054) | | Sleep duration/ quality not reported |
| [Kumar, M., Kumari, A., Rohilla, K. K., Dhawan, S., Singh, A., Sharma, N., Kriplani, N., Barari, N., & Soni, R. K. (2022). COVID-somnia: Sleep disturbance among Indian nurses during COVID-19 pandemic. *Journal of family medicine and primary care, 11*(6), 3167-3173. https://doi.org/https://dx.doi.org/10.4103/jfmpc.jfmpc_2113_21](https://doi.org/https:/dx.doi.org/10.4103/jfmpc.jfmpc_2113_21) | | Sleep duration/ quality not reported |
| [Harris, T. P., Vigoureux, T. F. D., & Lee, S. (2022). Daily associations between sleep and stressors in nurses with and without children. *Journal of Sleep Research, 31*(3), e13505. https://doi.org/https://dx.doi.org/10.1111/jsr.13505](https://doi.org/https:/dx.doi.org/10.1111/jsr.13505) | | Sleep duration/ quality not reported |
| [Gangwisch, J. E., Rexrode, K., Forman, J. P., Mukamal, K., Malaspina, D., & Feskanich, D. (2014). Daytime sleepiness and risk of coronary heart disease and stroke: Results from the Nurses' Health Study II. *Sleep Medicine, 15*(7), 782-788. https://doi.org/https://dx.doi.org/10.1016/j.sleep.2014.04.001](https://doi.org/https:/dx.doi.org/10.1016/j.sleep.2014.04.001) | | Sleep duration/ quality not reported |
| [Hasson, D., & Gustavsson, P. (2010). Declining sleep quality among nurses: A population- based four-year longitudinal study on the transition from nursing education to working life. *PLoS ONE, 5*(12), e14265. https://doi.org/https://dx.doi.org/10.1371/journal.pone.0014265](https://doi.org/https:/dx.doi.org/10.1371/journal.pone.0014265) | | Sleep duration/ quality not reported |
| [Junsatitanon, P., & Pongpirul, K. (2021). Depression and sleep quality among nurses in a tertiary hospital in Bangkok. *Chulalongkorn Medical Journal, 65*(2), 147-153. https://doi.org/10.14456/clmj.2021.20](https://doi.org/10.14456/clmj.2021.20) | | Sleep duration/ quality not reported |
| [Kim, O., Kim, S., Jeon, H. O., Kim, A., Cha, C., & Kim, B. (2022). Depressive symptoms and menstrual distress according to the menstrual phase in nurses: the Korea Nurses' Health Study. *Journal of Psychosomatic Obstetrics and Gynecology, 4*3(4), 541-549. https://doi.org/https://dx.doi.org/10.1080/0167482X.2022.2095999](https://doi.org/https:/dx.doi.org/10.1080/0167482X.2022.2095999) | | Sleep duration/ quality not reported |
| [Hruby, A., Manson, J. E., Qi, L., Malik, V. S., Rimm, E. B., Sun, Q., Willett, W. C., & Hu, F. B. (2016). Determinants and Consequences of Obesity. *American journal of public health, 106*(9), 1656-1662. https://doi.org/https://dx.doi.org/10.2105/AJPH.2016.303326](https://doi.org/https:/dx.doi.org/10.2105/AJPH.2016.303326) | | Sleep duration/ quality not reported |
| [Winwood, P. C., & Lushington, K. (2006). Disentangling the effects of psychological and physical work demands on sleep, recovery and maladaptive chronic stress outcomes within a large sample of Australian nurses. *Journal of Advanced Nursing, 56*(6), 679-689. https://doi.org/https://dx.doi.org/10.1111/j.1365-2648.2006.04055.x](https://doi.org/https:/dx.doi.org/10.1111/j.1365-2648.2006.04055.x) | | Sleep duration/ quality not reported |
| [Niu, J., An, Y., Xu, M., Zhang, L., Liu, J., Feng, X., Li, L., Song, X., & Niu, W. (2023). Do sleep and psychological factors influence musculoskeletal pain among nurses? *Work (Reading, Mass.), 75*(4), 1455-1465. https://doi.org/https://dx.doi.org/10.3233/WOR-211113](https://doi.org/https:/dx.doi.org/10.3233/WOR-211113) | | Sleep duration/ quality not reported |
| [Watanabe, M., Akamatsu, Y., Furui, H., Tomita, T., Watanabe, T., & Kobayashi, F. (2004). Effects of Changing Shift Schedules from A Full-day to A Half-day Shift before A Night Shift on Physical Activities and Sleep Patterns of Single Nurses and Married Nurses with Children. *Industrial Health, 42(*1), 34-40. https://doi.org/https://dx.doi.org/10.2486/indhealth.42.34](https://doi.org/https:/dx.doi.org/10.2486/indhealth.42.34) | | Sleep duration/ quality not reported |
| [i, Y., Wang, Y., Lv, X., Li, R., Guan, X., Li, L., Li, J., & Cao, Y. (2022). Effects of Factors Related to Shift Work on Depression and Anxiety in Nurses. *Frontiers in Public Health, 10,* 926988. https://doi.org/https://dx.doi.org/10.3389/fpubh.2022.926988](https://doi.org/https:/dx.doi.org/10.3389/fpubh.2022.926988) | | Sleep duration/ quality not reported |
| [Niedhammer, I., Lert, F., & Marne, M. J. (1994). Effects of shift work on sleep among French nurses: A longitudinal study. *Journal of Occupational Medicine, 36*(6), 667-674. http://ovidsp.ovid.com/ovidweb.cgi?T=JS&PAGE=reference&D=emed5&NEWS=N&AN=24177359](http://ovidsp.ovid.com/ovidweb.cgi?T=JS&PAGE=reference&D=emed5&NEWS=N&AN=24177359) | | Sleep duration/ quality not reported |
| [EscribÀ‐AgÜIr, V., PÉRez‐Hoyos, S., & Bolumar, F. (1993). Effects of work organization on the mental health of nursing staff. *Journal of Nursing Management, 1*(1), 3-8. https://doi.org/10.1111/j.1365-2834.1993.tb00176.x](https://doi.org/10.1111/j.1365-2834.1993.tb00176.x) | | Sleep duration/ quality not reported |
| [Perez-Fuentes, M. C., Jurado, M. M. M., Marquez, M. M. S., Martin, A. B. B., & Linares, J. J. G. (2019). Emotional effects of the duration, efficiency and subjective quality of sleep in healthcare personnel. *International Journal of Environmental Research and Public Health, 16*(19), 3512. https://doi.org/https://dx.doi.org/10.3390/ijerph16193512](https://doi.org/https:/dx.doi.org/10.3390/ijerph16193512) | | Sleep duration/ quality not reported |
| [Dyer, N. L., Baldwin, A. L., Pharo, R., & Gray, F. (2023). Evaluation of a Distance Reiki Program for Frontline Healthcare Workers' Health-Related Quality of Life During the COVID-19 Pandemic. *Global Advances in Integrative Medicine and Health, 12*. https://doi.org/https://dx.doi.org/10.1177/27536130231187368](https://doi.org/https:/dx.doi.org/10.1177/27536130231187368) | | Sleep duration/ quality not reported |
| [An, M., Heo, S., Hwang, Y. Y., Kim, J. S., & Lee, Y. (2022). Factors Affecting Turnover Intention among New Graduate Nurses: Focusing on Job Stress and Sleep Disturbance. *Healthcare (Switzerland), 10*(6). https://doi.org/10.3390/healthcare10061122](https://doi.org/10.3390/healthcare10061122) | | Sleep duration/ quality not reported |
| [Higgins, J. T., Okoli, C., Otachi, J., Lawrence, J., Bryant, E. D., Lykins, A., & Seng, S. (2020). Factors Associated With Burnout in Trauma Nurses. *Journal of trauma nursing : the official journal of the Society of Trauma Nurses, 27*(6), 319-326. https://doi.org/https://dx.doi.org/10.1097/JTN.0000000000000538](https://doi.org/https:/dx.doi.org/10.1097/JTN.0000000000000538) | | Sleep duration/ quality not reported |
| [Wang, J., Okoli, C. T. C., He, H., Feng, F., Li, J., Zhuang, L., & Lin, M. (2020). Factors associated with compassion satisfaction, burnout, and secondary traumatic stress among Chinese nurses in tertiary hospitals: A cross-sectional study. *International Journal of Nursing Studies, 102,* 103472. https://doi.org/https://dx.doi.org/10.1016/j.ijnurstu.2019.103472](https://doi.org/https:/dx.doi.org/10.1016/j.ijnurstu.2019.103472) | | Sleep duration/ quality not reported |
| [Chan, M. F. (2009). Factors associated with perceived sleep quality of nurses working on rotating shifts. *Journal of Clinical Nursing, 18*(2), 285-293. https://doi.org/https://dx.doi.org/10.1111/j.1365-2702.2008.02583.x](https://doi.org/https:/dx.doi.org/10.1111/j.1365-2702.2008.02583.x) | | Sleep duration/ quality not reported |
| [Kunzweiler, K., Voigt, K., Kugler, J., Hirsch, K., Bergmann, A., & Riemenschneider, H. (2016). Factors influencing sleep quality among nursing staff: Results of a cross sectional study. *Applied nursing research : ANR, 32*, 241-244. https://doi.org/https://dx.doi.org/10.1016/j.apnr.2016.08.007](https://doi.org/https:/dx.doi.org/10.1016/j.apnr.2016.08.007) | | Sleep duration/ quality not reported |
| [Ramadhani, M., Muis, M., Thamrin, Y., Furqaan Naiem, M., Indar, I., & Wahiduddn, W. (2021). Factors that affecting female nurse performance with work stress as an intervening variable in the hospital of dr. Tadjuddin chalid makassar. *Open Access Macedonian Journal of Medical Sciences, 9*, 835-842. https://doi.org/https://dx.doi.org/10.3889/oamjms.2021.6763](https://doi.org/https:/dx.doi.org/10.3889/oamjms.2021.6763) | | Sleep duration/ quality not reported |
| [Celik, S., Tasdemir, N., Kurt, A., Ilgezdi, E., & Kubalas, O. (2017). Fatigue in intensive care nurses and related factors. *International Journal of Occupational and Environmental Medicine, 8*(4), 199-206. https://doi.org/https://dx.doi.org/10.15171/ijoem.2017.1137](https://doi.org/https:/dx.doi.org/10.15171/ijoem.2017.1137) | | Sleep duration/ quality not reported |
| [De Los Santos, J. A. A., Labrague, L. J., & Falguera, C. C. (2022). Fear of COVID-19, poor quality of sleep, irritability, and intention to quit school among nursing students: A cross-sectional study. P*erspectives in Psychiatric Care, 5*8(1), 71-78. https://doi.org/https://dx.doi.org/10.1111/ppc.12781](https://doi.org/https:/dx.doi.org/10.1111/ppc.12781) | | Sleep duration/ quality not reported |
| [da Silva, R. M., Costa, A. L. S., Mussi, F. C., Lopes, V. C., de Melo Batista, K., & dos Santos, O. P. (2019). Health alterations in nursing students after a year from admission to the undergraduate course. *Revista da Escola de Enfermagem, 53*, e03450. https://doi.org/https://dx.doi.org/10.1590/S1980-220X2018008103450](https://doi.org/https:/dx.doi.org/10.1590/S1980-220X2018008103450) | | Sleep duration/ quality not reported |
| [Iskera-golec, I., Folkard, S., Marek, T., & Noworol, C. (1996). Health, well-being and burnout of ICU nurses on 12- and 8-h shifts. *Work and Stress, 10*(3), 251-256. https://doi.org/10.1080/02678379608256804](https://doi.org/10.1080/02678379608256804) | | Sleep duration/ quality not reported |
| [Kang, J., & Lee, Y. (2022). Health-Related Factors Influencing Nurse Turnover by Clinical Career: A Secondary Data Analysis of Clinical Nurses in South Korea. *International Journal of Environmental Research and Public Health, 19(*22), 15222. https://doi.org/https://dx.doi.org/10.3390/ijerph192215222](https://doi.org/https:/dx.doi.org/10.3390/ijerph192215222) | | Sleep duration/ quality not reported |
| [Nadir, Y., Saharov, G., Hoffman, R., Keren-Politansky, A., Tzoran, I., Brenner, B., & Shochat, T. (2015). Heparanase procoagulant activity, factor Xa, and plasminogen activator inhibitor 1 are increased in shift work female nurses. *Annals of Hematolog*y. https://doi.org/https://dx.doi.org/10.1007/s00277-015-2345-8](https://doi.org/https:/dx.doi.org/10.1007/s00277-015-2345-8) | | Sleep duration/ quality not reported |
| [Zhang, H., Zhou, Z. E., Zhang, L., Liu, Y., & Shi, Y. (2023). How customer mistreatment hinders employee sleep quality and next-morning vigor: The effects of affective rumination and mindfulness. *Applied Psychology.* https://doi.org/https://dx.doi.org/10.1111/apps.12507](https://doi.org/https:/dx.doi.org/10.1111/apps.12507) | | Sleep duration/ quality not reported |
| [Benjamin, L. S., Pasay-An, E., Pangket, P., Alqarni, A. S., Gonzales, F., Sacgaca, L., Mahmoud, D. A., Mohsen, M. M., Ali Hamdi, Y. S., & Shanmugam, S. R. (2024). Impact of Sleep and Psychological Well-Being on the Academic and Clinical Performance of Nursing Students in Saudi Arabia. P*sychology Research and Behavior Management, 17,* 1355-1364. https://doi.org/https://dx.doi.org/10.2147/PRBM.S453685](https://doi.org/https:/dx.doi.org/10.2147/PRBM.S453685) | | Sleep duration/ quality not reported |
| [Tanveer, H., Munir, I., Osama, M., Zia, M. R., Chattha, I. R., & Zaffar, S. (2023). Impact of Sleep Deprivation on Academic Performance of Nursing Students at CMH, Lahore. *Pakistan Journal of Medical and Health Sciences, 17*(1), 289-291. https://doi.org/https://dx.doi.org/10.53350/pjmhs2023171289](https://doi.org/https:/dx.doi.org/10.53350/pjmhs2023171289) | | Sleep duration/ quality not reported |
| [Rolland, F., Frajerman, A., Falissard, B., Bertschy, G., Diquet, B., & Marra, D. (2023). *Impact of the first wave of the COVID-19 pandemic on French Health students. Encephale, 49(*3), 219-226. https://doi.org/https://dx.doi.org/10.1016/j.encep.2021.12.004](https://doi.org/https:/dx.doi.org/10.1016/j.encep.2021.12.004) | | Sleep duration/ quality not reported |
| [Zhang, S.-E., Liu, W., Wang, J., Shi, Y., Xie, F., Cang, S., Sun, T., & Fan, L. (2018). Impact of workplace violence and compassionate behaviour in hospitals on stress, sleep quality and subjective health status among Chinese nurses: a cross-sectional survey. *BMJ Open, 8(*10), e019373. https://doi.org/https://dx.doi.org/10.1136/bmjopen-2017-019373](https://doi.org/https:/dx.doi.org/10.1136/bmjopen-2017-019373) | | Sleep duration/ quality not reported |
| [Fagundo-Rivera, J., Allande-Cussó, R., Ortega-Moreno, M., García-Iglesias, J. J., Romero, A., Ruiz-Frutos, C., & Gómez-Salgado, J. (2021). Implications of lifestyle and occupational factors on the risk of breast cancer in shiftwork nurses. *Healthcare (Switzerland), 9*(6). https://doi.org/10.3390/healthcare9060649](https://doi.org/10.3390/healthcare9060649) | | Sleep duration/ quality not reported |
| [Schiavo, D., & de Martino, M. M. F. (2010). Incidence of weight gain and obesity in night shift workers and the relationship of these factors with sleep. *Sleep Science, 3*(4), 125-130. https://www.scopus.com/inward/record.uri?eid=2-s2.0-84893004834&partnerID=40&md5=5cff8763976b77b3894e33eed1065e65](https://www.scopus.com/inward/record.uri?eid=2-s2.0-84893004834&partnerID=40&md5=5cff8763976b77b3894e33eed1065e65) | | Sleep duration/ quality not reported |
| [Chang, W.-P. (2018). Influence of shift type on sleep quality of female nurses working monthly rotating shifts with cortisol awakening response as mediating variable. *Chronobiology International, 35(*11), 1503-1512. https://doi.org/https://dx.doi.org/10.1080/07420528.2018.1493491](https://doi.org/https:/dx.doi.org/10.1080/07420528.2018.1493491) | | Sleep duration/ quality not reported |
| [Chang, W.-P., & Wang, C.-H. (2022). Influence of sleep fragmentation and fatigue on turnover of female nurses working rotating shifts. J*ournal of Clinical Nursing, 31*(23-24), 3573-3583. https://doi.org/https://dx.doi.org/10.1111/jocn.16184](https://doi.org/https:/dx.doi.org/10.1111/jocn.16184) | | Sleep duration/ quality not reported |
| [Zhang, L., Sun, D.-M., Li, C.-B., & Tao, M.-F. (2016). Influencing Factors for Sleep Quality Among Shift-working Nurses: A Cross-Sectional Study in China Using 3-factor Pittsburgh Sleep Quality Index. *Asian nursing research, 10*(4), 277-282. https://doi.org/https://dx.doi.org/10.1016/j.anr.2016.09.002](https://doi.org/https:/dx.doi.org/10.1016/j.anr.2016.09.002) | | Sleep duration/ quality not reported |
| [Lai, H.-L., Lin, Y.-P., Chang, H.-K., Wang, S.-C., Liu, Y.-L., Lee, H.-C., Peng, T.-C., & Chang, F.-M. (2008). Intensive care unit staff nurses: Predicting factors for career decisions. *Journal of Clinical Nursing, 17*(14), 1886-1896. https://doi.org/https://dx.doi.org/10.1111/j.1365-2702.2007.02180.x](https://doi.org/https:/dx.doi.org/10.1111/j.1365-2702.2007.02180.x) | | Sleep duration/ quality not reported |
| [Jordan, D. G., Slavish, D. C., Dietch, J., Messman, B., Ruggero, C., Kelly, K., & Taylor, D. J. (2023). Investigating sleep, stress, and mood dynamics via temporal network analysis. *Sleep Medicine, 103*, 1-11. https://doi.org/https://dx.doi.org/10.1016/j.sleep.2023.01.007](https://doi.org/https:/dx.doi.org/10.1016/j.sleep.2023.01.007) | | Sleep duration/ quality not reported |
| [Park, L. S., Martinez, L., & Xu, S. (2022). Job experience as a buffer against incivility: a daily diary study. *Journal of Managerial Psychology, 37*(3), 243-263. https://doi.org/10.1108/JMP-03-2021-0194](https://doi.org/10.1108/JMP-03-2021-0194) | | Sleep duration/ quality not reported |
| [Yao, L., Chen, J., Zhao, Q., Bai, D., Li, Y., Xiao, M., Du, Y., & Liu, L. (2023). Level and influencing factors of transition shock among new nurses in China: A multicenter cross-sectional study. *Health Science Reports, 6*(12), e1758. https://doi.org/https://dx.doi.org/10.1002/hsr2.1758](https://doi.org/https:/dx.doi.org/10.1002/hsr2.1758) | | Sleep duration/ quality not reported |
| [Ching, S. M., Cheong, A. T., Yee, A., Thurasamy, R., Lim, P. Y., Ismail, I. Z., Mohammad, N. M., Ho, B. K., Ng, J. Y., Tan, S. F., Rashid, Z. Z. Z., Jogulu, S. R., Lee, K. W., & Taher, S. W. (2023). Level of fear towards COVID-19 and its determinants among healthcare providers in Malaysia: A cross-sectional study. *Malaysian Family Physician, 18*, 56. https://doi.org/https://dx.doi.org/10.51866/oa.238](https://doi.org/https:/dx.doi.org/10.51866/oa.238) | | Sleep duration/ quality not reported |
| [Correa Pinto, R. N., Da Silva, M. C., Caputo, E. L., & Domingues, M. R. (2021). Low back pain prevalence and associated factors in nurses from Brazilian primary health units. *Work, 70*(1), 279-285. https://doi.org/https://dx.doi.org/10.3233/WOR-213572](https://doi.org/https:/dx.doi.org/10.3233/WOR-213572) | | Sleep duration/ quality not reported |
| [Geiger Brown, J., Wieroney, M., Blair, L., Zhu, S., Warren, J., Scharf, S. M., & Hinds, P. S. (2014). Measuring subjective sleepiness at work in hospital nurses: validation of a modified delivery format of the Karolinska Sleepiness Scale. *Sleep and Breathing, 18*(4), 731-739. https://doi.org/10.1007/s11325-013-0935-z](https://doi.org/10.1007/s11325-013-0935-z) | | Sleep duration/ quality not reported |
| [Gao, J., Wang, F., Guo, S., & Hu, F. (2021). Mental Health of Nursing Students amid Coronavirus Disease 2019 Pandemic. *Frontiers in Psychology, 12.* https://doi.org/10.3389/fpsyg.2021.699558](https://doi.org/10.3389/fpsyg.2021.699558) | | Sleep duration/ quality not reported |
| [El Fouhil, A. F., Aldakheel, Y. F., Alnamlah, I. S., Alsqabi, H. A., & Alfaifi, A. H. (2023). Mental Illnesses and Quality of Sleep Among Nurses Working at a Tertiary Hospital in Riyadh During the COVID-19 Pandemic. *Cureus, 15*(10), e47394. https://doi.org/https://dx.doi.org/10.7759/cureus.47394](https://doi.org/https:/dx.doi.org/10.7759/cureus.47394) | | Sleep duration/ quality not reported |
| [Bani Issa, W., Abdul Rahman, H., Albluwi, N., Samsudin, A. B. R., Abraham, S., Saqan, R., & Naing, L. (2020). Morning and evening salivary melatonin, sleepiness and chronotype: A comparative study of nurses on fixed day and rotating night shifts. *Journal of Advanced Nursing, 76*(12), 3372-3384. https://doi.org/https://dx.doi.org/10.1111/jan.14530](https://doi.org/https:/dx.doi.org/10.1111/jan.14530) | | Sleep duration/ quality not reported |
| [Demir Zencirci, A., & Arslan, S. (2011). Morning-evening type and burnout level as factors influencing sleep quality of shift nurses: a questionnaire study. *Croatian medical journal, 52(*4), 527-537. https://doi.org/https://dx.doi.org/10.3325/cmj.2011.52.527](https://doi.org/https:/dx.doi.org/10.3325/cmj.2011.52.527) | | Sleep duration/ quality not reported |
| [Ohida, T., Kamal, A. M. M., Sone, T., Ishii, T., Uchiyama, M., Minowa, M., & Nozaki, S. (2001). Night-shift work related problems in young female nurses in Japan. *Journal of Occupational Health, 43*(3), 150-156. https://doi.org/http://dx.doi.org/10.1539/joh.43.150](https://doi.org/http:/dx.doi.org/10.1539/joh.43.150) | | Sleep duration/ quality not reported |
| [Liao, T., Liu, Y., Luo, W., Duan, Z., Zhan, K., Lu, H., & Chen, X. (2024). Non-linear association of years of experience and burnout among nursing staff: a restricted cubic spline analysis*. Frontiers in Public Health, 12,* 1343293. https://doi.org/https://dx.doi.org/10.3389/fpubh.2024.1343293](https://doi.org/https:/dx.doi.org/10.3389/fpubh.2024.1343293) | | Sleep duration/ quality not reported |
| [Hittle, B. M., Caruso, C. C., Jones, H. J., Bhattacharya, A., Lambert, J., & Gillespie, G. L. (2020). Nurse Health: The Influence of Chronotype and Shift Timing. *Western journal of nursing research, 42*(12), 1031-1041. https://doi.org/https://dx.doi.org/10.1177/0193945920916802](https://doi.org/https:/dx.doi.org/10.1177/0193945920916802) | | Sleep duration/ quality not reported |
| [Higbee, M. R., Chilton, J. M., El-Saidi, M., Duke, G., & Haas, B. K. (2020). Nurses Consuming Energy Drinks Report Poorer Sleep and Higher Stress. *Western journal of nursing research, 42*(1), 24-31. https://doi.org/https://dx.doi.org/10.1177/0193945919840991](https://doi.org/https:/dx.doi.org/10.1177/0193945919840991) | | Sleep duration/ quality not reported |
| [Tung, C.-Y., Chang, C.-C., Ming, J.-L., & Chao, K.-P. (2014). Occupational hazards education for nursing staff through web-based learning. *International Journal of Environmental Research and Public Health, 11*(12), 13035-13046. https://doi.org/https://dx.doi.org/10.3390/ijerph111213035](https://doi.org/https:/dx.doi.org/10.3390/ijerph111213035) | | Sleep duration/ quality not reported |
| [Orji, E. O., Fasubaa, O. B., Onwudiegwu, U., Dare, F. O., & Ogunniyi, S. O. (2002). Occupational health hazards among health care workers in an obstetrics and gynaecology unit of a Nigerian teaching hospital. *Journal of Obstetrics and Gynaecology, 22*(1), 75-78. https://doi.org/10.1080/01443610120101781](https://doi.org/10.1080/01443610120101781) | | Sleep duration/ quality not reported |
| [Labrague, L. J. (2021). Pandemic fatigue and clinical nurses' mental health, sleep quality and job contentment during the covid-19 pandemic: The mediating role of resilience. *Journal of Nursing Management, 29*(7), 1992-2001. https://doi.org/https://dx.doi.org/10.1111/jonm.13383](https://doi.org/https:/dx.doi.org/10.1111/jonm.13383) | | Sleep duration/ quality not reported |
| [Albikawi, Z. F. (2023). Perceived Stress, Physical Activity, and Insomnia of Female Nursing University Students in Saudi Arabia: A Cross-Sectional Study. *Universal Journal of Public Health, 11*(1), 57-67. https://doi.org/10.13189/ujph.2023.110107](https://doi.org/10.13189/ujph.2023.110107) | | Sleep duration/ quality not reported |
| [Abebe, G. K., Melkie, K. A., Alamaw, A. W., Zemariam, A. B., & Workneh, B. S. (2023). Perceived work-ability and its associated factors among nurses working in the Northwest of Amhara regional state Referral Hospitals, Northwest Ethiopia, 2022. *BMC public health, 23*(1), 1460. https://doi.org/https://dx.doi.org/10.1186/s12889-023-16386-0](https://doi.org/https:/dx.doi.org/10.1186/s12889-023-16386-0) | | Sleep duration/ quality not reported |
| [Yu, F., Cavadino, A., Mackay, L., Ward, K., King, A., & Smith, M. (2020). Physical activity and personal factors associated with nurse resilience in intensive care units. *Journal of Clinical Nursing, 29*(17-18), 3246-3262. https://doi.org/https://dx.doi.org/10.1111/jocn.15338](https://doi.org/https:/dx.doi.org/10.1111/jocn.15338) | | Sleep duration/ quality not reported |
| [Trinkoff, A. M., Storr, C. L., & Lipscomb, J. A. (2001). Physically demanding work and inadequate sleep, pain medication use, and absenteeism in registered nurses. *Journal of Occupational and Environmental Medicine, 43*(4), 355-363. https://doi.org/https://dx.doi.org/10.1097/00043764-200104000-00012](https://doi.org/https:/dx.doi.org/10.1097/00043764-200104000-00012) | | Sleep duration/ quality not reported |
| [Vinstrup, J., Jakobsen, M. D., & Andersen, L. L. (2020). Poor sleep is a risk factor for low-back pain among healthcare workers: Prospective cohort study. *International Journal of Environmental Research and Public Health, 17*(3), 996. https://doi.org/https://dx.doi.org/10.3390/ijerph17030996](https://doi.org/https:/dx.doi.org/10.3390/ijerph17030996) | | Sleep duration/ quality not reported |
| [Nilsson, A., Lindberg, P., & Denison, E. (2010). Predicting of pain, disability, and sick leave regarding a non-clinical sample among Swedish nurses. *Scandinavian Journal of Pain, 1*(3), 160-166. https://doi.org/https://dx.doi.org/10.1016/j.sjpain.2010.05.029](https://doi.org/https:/dx.doi.org/10.1016/j.sjpain.2010.05.029) | | Sleep duration/ quality not reported |
| [Kim, O., & Jung, H. (2021). Prediction model for abnormal eating behaviour among hospital nurses: A structural equation modelling approach. *International journal of nursing practice, 27*(5), e13006. https://doi.org/https://dx.doi.org/10.1111/ijn.13006](https://doi.org/https:/dx.doi.org/10.1111/ijn.13006) | | Sleep duration/ quality not reported |
| [Pang, Y., Fang, H., Li, L., Chen, M., & Chen, Y. (2021). Predictive factors of anxiety and depression among nurses fighting coronavirus disease 2019 in China. *International Journal of Mental Health Nursing, 30*(2), 524-532. https://doi.org/10.1111/inm.12817](https://doi.org/10.1111/inm.12817) | | Sleep duration/ quality not reported |
| [Chen, D., Jiang, M., Shi, X., Geng, F., Qi, H., Zhang, Y., Lai, Y., & Fan, F. (2020). Predictors of the initiation of shift work disorder among Chinese intern nurses: a prospective study. *Sleep Medicine, 68,* 199-206. https://doi.org/10.1016/j.sleep.2019.11.1263](https://doi.org/10.1016/j.sleep.2019.11.1263) | | Sleep duration/ quality not reported |
| [Rajčáni, J., Vytykačová, S., Botíkov, V., & Šuhajdová, B. (2022). PREDICTORS OF TRAUMATIC STRESS IN NURSES DURING THE COVID-19 PANDEMIC. *Ceskoslovenska Psychologie, 66*(1), 17-29. https://doi.org/10.51561/cspsych.66.1.17](https://doi.org/10.51561/cspsych.66.1.17) | | Sleep duration/ quality not reported |
| [Han, K., Kim, Y.-H., Lee, H. Y., & Lim, S. (2019). Pre-employment health lifestyle profiles and actual turnover among newly graduated nurses: A descriptive and prospective longitudinal study. *International Journal of Nursing Studies, 98*, 1-8. https://doi.org/https://dx.doi.org/10.1016/j.ijnurstu.2019.05.014](https://doi.org/https:/dx.doi.org/10.1016/j.ijnurstu.2019.05.014) | | Sleep duration/ quality not reported |
| [Silver, S. R., Li, J., Marsh, S. M., & Carbone, E. G. (2022). Prepandemic Mental Health and Well-being: Differences Within the Health Care Workforce and the Need for Targeted Resources. *Journal of Occupational and Environmental Medicine, 64*(12), 1025-1035. https://doi.org/https://dx.doi.org/10.1097/JOM.0000000000002630](https://doi.org/https:/dx.doi.org/10.1097/JOM.0000000000002630) | | Sleep duration/ quality not reported |
| [Qiao, Z., Chen, L., Chen, M., Guan, X., Wang, L., Jiao, Y., Yang, J., Tang, Q., Yang, X., Qiu, X., Han, D., Ma, J., Yang, Y., & Zhai, X. (2016). Prevalence and factors associated with occupational burnout among HIV/AIDS healthcare workers in China: a cross-sectional study. *BMC public health, 16,* 335. https://doi.org/https://dx.doi.org/10.1186/s12889-016-2890-7](https://doi.org/https:/dx.doi.org/10.1186/s12889-016-2890-7) | | Sleep duration/ quality not reported |
| [Nazatul, S. M., Saimy, I., Moy, F. M., & Nabila, A. S. (2008). Prevalence of sleep disturbance among nurses in a malaysian government Hospital and its association with work characteristics. *Journal of Health and Translational Medicine, 11(*2), 66-71. http://jummec.um.edu.my/currenthttp://ovidsp.ovid.com/ovidweb.cgi?T=JS&PAGE=reference&D=emed10&NEWS=N&AN=354781749](http://jummec.um.edu.my/currenthttp:/ovidsp.ovid.com/ovidweb.cgi?T=JS&PAGE=reference&D=emed10&NEWS=N&AN=354781749) | | Sleep duration/ quality not reported |
| [Oster, K., Tucker, P., Soderstrom, M., & Dahlgren, A. (2023). Pros and cons of quick returns-a cross-sectional survey among Swedish nurses and nurse assistants. *Industrial Health, 61*(5), 379-392. https://doi.org/https://dx.doi.org/10.2486/indhealth.2022-0033](https://doi.org/https:/dx.doi.org/10.2486/indhealth.2022-0033) | | Sleep duration/ quality not reported |
| [Buccione, E., Santella, B., Fiani, M. E., Maffeo, M., Tedesco, B., D'Errico, A., Della Pelle, C., Bambi, S., & Rasero, L. (2022). Quality of Life of Pediatric Nurses During the COVID-19 Pandemic: A Cross-sectional Study. *Dimensions of critical care nursing : DCCN, 41*(5), 246-255. https://doi.org/https://dx.doi.org/10.1097/DCC.0000000000000537](https://doi.org/https:/dx.doi.org/10.1097/DCC.0000000000000537) | | Sleep duration/ quality not reported |
| [Dahlgren, A., Tucker, P., Gustavsson, P., & Rudman, A. (2016). Quick returns and night work as predictors of sleep quality, fatigue, work-family balance and satisfaction with work hours. *Chronobiology International, 33*(6), 759-767. https://doi.org/https://dx.doi.org/10.3109/07420528.2016.1167725](https://doi.org/https:/dx.doi.org/10.3109/07420528.2016.1167725) | | Sleep duration/ quality not reported |
| [Ding, J., Gehrman, P. R., Liu, S., Yang, F., Ma, R., Jia, Y., & Yang, X. (2020). Recovery experience as the mediating factor in the relationship between sleep disturbance and depressive symptoms among female nurses in chinese public hospitals: A structural equation modeling analysis. *Psychology Research and Behavior Management, 13*, 303-311. https://doi.org/https://dx.doi.org/10.2147/PRBM.S243864](https://doi.org/https:/dx.doi.org/10.2147/PRBM.S243864) | | Sleep duration/ quality not reported |
| [Ce, E., Doria, C., Roveda, E., Montaruli, A., Galasso, L., Castelli, L., Mule, A., Longo, S., Coratella, G., D'Aloia, P., Banfi, G., & Esposito, F. (2020). Reduced Neuromuscular Performance in Night Shift Orthopedic Nurses: New Insights From a Combined Electromyographic and Force Signals Approach. *Frontiers in Physiology, 11,* 693. https://doi.org/https://dx.doi.org/10.3389/fphys.2020.00693](https://doi.org/https:/dx.doi.org/10.3389/fphys.2020.00693) | | Sleep duration/ quality not reported |
| [Silva-Costa, A., Fischer, F. M., Griep, R. H., & Rotenberg, L. (2013). Relationship between napping during night shift work and household obligations of female nursing personnel. *International Journal of General Medicine, 6,* 227-231. https://doi.org/https://dx.doi.org/10.2147/IJGM.S41200](https://doi.org/https:/dx.doi.org/10.2147/IJGM.S41200) | | Sleep duration/ quality not reported |
| [Baek, J., Ki, J., Ryu, J., & Smi, C.-K. (2022). Relationship between occupational stress, sleep disturbance, and presenteeism of shiftwork nurses. *Journal of Nursing Scholarship, 54*(5), 631-638. https://doi.org/https://dx.doi.org/10.1111/jnu.12766](https://doi.org/https:/dx.doi.org/10.1111/jnu.12766) | | Sleep duration/ quality not reported |
| [Tang, H. Y., Ko, W. S., Yan, Y. H., Yu, S. C., & Chiou, Y. L. (2022). Relationship between serum 25 hydroxyvitamin D (25(OH)D) levels and mental health in shift female nurses. *Scientific Reports, 12*(1). https://doi.org/10.1038/s41598-022-18721-8](https://doi.org/10.1038/s41598-022-18721-8) | | Sleep duration/ quality not reported |
| [Tsipkalo, A., Kozak, K., & Marushchak, M. (2024). Relationship between sleep quality and blood pressure level in nurses performing shift work. *Frontiers of Nursing, 11(*1), 77-84. https://doi.org/https://dx.doi.org/10.2478/FON-2024-0008](https://doi.org/https:/dx.doi.org/10.2478/FON-2024-0008) | | Sleep duration/ quality not reported |
| [Silva-Costa, A., Rotenberg, L., Griep, R. H., & Fischer, F. M. (2011). Relationship between sleeping on the night shift and recovery from work among nursing workers - the influence of domestic work. *Journal of Advanced Nursing, 67*(5), 972-981. https://doi.org/https://dx.doi.org/10.1111/j.1365-2648.2010.05552.x](https://doi.org/https:/dx.doi.org/10.1111/j.1365-2648.2010.05552.x) | | Sleep duration/ quality not reported |
| [Ngo, T. P., Antisdel, J. A. L., Xing, K., & Reising, D. L. (2023). Relationships Between Remote Learning Modalities and Nursing Students' Perceptions of Their Sleep Quality During the COVID-19 Pandemic. *Nurse educator, 48*(2), E41-E46. https://doi.org/https://dx.doi.org/10.1097/NNE.0000000000001320](https://doi.org/https:/dx.doi.org/10.1097/NNE.0000000000001320) | | Sleep duration/ quality not reported |
| [Belingheri, M., Pellegrini, A., Facchetti, R., de Vito, G., Cesana, G., & Riva, M. A. (2021). Self-reported prevalence of sleep disorders among medical and nursing students. *Occupational Medicine, 70*(2), 127-130. https://doi.org/https://dx.doi.org/10.1093/OCCMED/KQAA011](https://doi.org/https:/dx.doi.org/10.1093/OCCMED/KQAA011) | | Sleep duration/ quality not reported |
| [Jensen, H. I., Markvart, J., Holst, R., Thomsen, T. D., Larsen, J. W., Eg, D. M., & Nielsen, L. S. (2016). Shift work and quality of sleep: effect of working in designed dynamic light. *International Archives of Occupational and Environmental Health, 89*(1), 49-61. https://doi.org/https://dx.doi.org/10.1007/s00420-015-1051-0](https://doi.org/https:/dx.doi.org/10.1007/s00420-015-1051-0) | | Sleep duration/ quality not reported |
| [Escriba, V., Perez-Hoyos, S., & Bolumar, F. (1992). Shiftwork: its impact on the length and quality of sleep among nurses of the Valencian region in Spain. *International Archives of Occupational and Environmental Health, 64*(2), 125-129. http://ovidsp.ovid.com/ovidweb.cgi?T=JS&PAGE=reference&D=emed4&NEWS=N&AN=22275953](http://ovidsp.ovid.com/ovidweb.cgi?T=JS&PAGE=reference&D=emed4&NEWS=N&AN=22275953) | | Sleep duration/ quality not reported |
| [Zhang, L., Zhang, R., Shen, Y., Qiao, S., Hui, Z., & Chen, J. (2020). Shimian granules improve sleep, mood and performance of shift nurses in association changes in melatonin and cytokine biomarkers: a randomized, double-blind, placebo-controlled pilot study. *Chronobiology International, 37*(4), 592-605. https://doi.org/https://dx.doi.org/10.1080/07420528.2020.1730880](https://doi.org/https:/dx.doi.org/10.1080/07420528.2020.1730880) | | Sleep duration/ quality not reported |
| [Dorrian, J., Tolley, C., Lamond, N., van den Heuvel, C., Pincombe, J., Rogers, A. E., & Drew, D. (2008). Sleep and errors in a group of Australian hospital nurses at work and during the commute. *Applied Ergonomics, 39*(5), 605-613. https://doi.org/https://dx.doi.org/10.1016/j.apergo.2008.01.012](https://doi.org/https:/dx.doi.org/10.1016/j.apergo.2008.01.012) | | Sleep duration/ quality not reported |
| [Puerta, Y., García, M., Heras, E., López-Herce, J., Fernández, S. N., & Mencía, S. (2017). Sleep characteristics of the staffworking in a pediatric intensive care unit based on a survey. *Frontiers in Pediatrics, 5*. https://doi.org/10.3389/fped.2017.00288](https://doi.org/10.3389/fped.2017.00288) | | Sleep duration/ quality not reported |
| [Infante-Rivard, C., Dumont, M., & Montplaisir, J. (1989). Sleep disorder symptoms among nurses and nursing aides. *International Archives of Occupational and Environmental Health, 61*(5), 353-358. https://doi.org/https://dx.doi.org/10.1007/BF00409392](https://doi.org/https:/dx.doi.org/10.1007/BF00409392) | | Sleep duration/ quality not reported |
| [Arif, I., Ali, S., Khan, A., Khan, K., Ullah, M., & Zeb, H. (2020). Sleep disorders among rotating shift and day-working nurses in public and private sector hospitals of Peshawar. *Journal of the Dow University of Health Sciences, 14(*3), 103-113. https://doi.org/10.36570/jduhs.2020.3.994](https://doi.org/10.36570/jduhs.2020.3.994) | | Sleep duration/ quality not reported |
| [Belingheri, M., Luciani, M., Ausili, D., Paladino, M. E., Di Mauro, S., De Vito, G., & Riva, M. A. (2022). Sleep disorders and night-shift work in nursing students: a cross-sectional study. *La Medicina del lavoro, 113(*1), e2022003. https://doi.org/https://dx.doi.org/10.23749/mdl.v113i1.12150](https://doi.org/https:/dx.doi.org/10.23749/mdl.v113i1.12150) | | Sleep duration/ quality not reported |
| [Korompeli, A., Chara, T., Chrysoula, L., & Sourtzi, P. (2013). Sleep Disturbance in Nursing Personnel Working Shifts. *Nursing Forum, 48(*1), 45-53. https://doi.org/https://dx.doi.org/10.1111/nuf.12005](https://doi.org/https:/dx.doi.org/10.1111/nuf.12005) | | Sleep duration/ quality not reported |
| [Chaiard, J., Deeluea, J., Suksatit, B., Songkham, W., Inta, N., & Stone, T. E. (2019). Sleep disturbances and related factors among nurses. *Nursing & health sciences, 21*(4), 470-478. https://doi.org/https://dx.doi.org/10.1111/nhs.12626](https://doi.org/https:/dx.doi.org/10.1111/nhs.12626) | | Sleep duration/ quality not reported |
| [Devore, E. E., Grodstein, F., Duffy, J. F., Stampfer, M. J., Czeisler, C. A., & Schernhammer, E. S. (2014). Sleep duration in midlife and later life in relation to cognition. *Journal of the American Geriatrics Society, 62*(6), 1073-1081. https://doi.org/https://dx.doi.org/10.1111/jgs.12790](https://doi.org/https:/dx.doi.org/10.1111/jgs.12790) | | Sleep duration/ quality not reported |
| [Díaz-Ramiro, E. M., Rubio-Valdehita, S., López-Núñez, M. I., & Aparicio-García, M. E. (2020). Sleep habits as predictors of psychological health in healthcare pro-fessionals. *Anales de Psicologia, 36*(2), 242-246. https://doi.org/10.6018/analesps.350301](https://doi.org/10.6018/analesps.350301) | | Sleep duration/ quality not reported |
| [Cavalheiri, J. C., Pascotto, C. R., Tonini, N. S., Vieira, A. P., Ferreto, L. E. D., & Follador, F. A. C. (2021). Sleep quality and common mental disorder in the hospital Nursing team. *Revista latino-americana de enfermagem, 29,* e3444. https://doi.org/https://dx.doi.org/10.1590/1518-8345.4280.3444](https://doi.org/https:/dx.doi.org/10.1590/1518-8345.4280.3444) | | Sleep duration/ quality not reported |
| [Menon, B., Karishma, H. P., & Mamatha, I. V. (2015). Sleep quality and health complaints among nursing students. *Annals of Indian Academy of Neurology, 18*(3), 363-364. https://doi.org/https://dx.doi.org/10.4103/0972-2327.157252](https://doi.org/https:/dx.doi.org/10.4103/0972-2327.157252) | | Sleep duration/ quality not reported |
| [Olana, D. D., Ayana, A. M., & Abebe, S. T. (2020). Sleep quality and its associated factors among nurses in jimma zone public hospitals, southwest Ethiopia, 2018. *Sleep and Hypnosis, 21(*4), 271-280. https://doi.org/https://dx.doi.org/10.37133/Sleep.Hypn.2019.21.0197](https://doi.org/https:/dx.doi.org/10.37133/Sleep.Hypn.2019.21.0197) | | Sleep duration/ quality not reported |
| [Kabbadj, K., Amekran, Y., Damoun, N., Taiek, N., & El Hangouche, A. J. (2023). Sleep quality and shift work among Moroccan nurses: A cross-sectional study. *Journal of education and health promotion, 12,* 453. https://doi.org/https://dx.doi.org/10.4103/jehp.jehp_664_23](https://doi.org/https:/dx.doi.org/10.4103/jehp.jehp_664_23) | | Sleep duration/ quality not reported |
| [Durkin, A. C., & Richards, A. J. (2024). Sleep quality and the intention to modify sleep behaviors among night-shift nurses. *Nursing, 54(*3), 54-58. https://doi.org/https://dx.doi.org/10.1097/01.NURSE.0001006268.77409.dc](https://doi.org/https:/dx.doi.org/10.1097/01.NURSE.0001006268.77409.dc) | | Sleep duration/ quality not reported |
| [Song, Y., Yang, F., Sznajder, K., & Yang, X. (2020). Sleep Quality as a Mediator in the Relationship Between Perceived Stress and Job Burnout Among Chinese Nurses: A Structural Equation Modeling Analysis. *Frontiers in Psychiatry, 11,* 566196. https://doi.org/https://dx.doi.org/10.3389/fpsyt.2020.566196](https://doi.org/https:/dx.doi.org/10.3389/fpsyt.2020.566196) | | Sleep duration/ quality not reported |
| [Petrov, M. E., Clark, C. B., Molzof, H. E., Johnson, R. L., Cropsey, K. L., & Gamble, K. L. (2014). Sleep strategies of night-shift nurses on days off: Which ones are most adaptive? *Frontiers in Neurology, 5*, 277. https://doi.org/https://dx.doi.org/10.3389/fneur.2014.00277](https://doi.org/https:/dx.doi.org/10.3389/fneur.2014.00277) | | Sleep duration/ quality not reported |
| [Moreno-Casbas, M. T., Ruzafa-Martinez, M., Rol, M. A., Madrid, J. A., Serrano Pinto, A., Gonzalez-Maria, E., & Fuentelsaz-Gallego, C. (2014). Sleepiness in Spanish nursing staff - Influence of chronotype and care unit in circadian rhythm impairment: Research protocol. *Journal of Advanced Nursing, 70*(1), 211-219. https://doi.org/https://dx.doi.org/10.1111/jan.12200](https://doi.org/https:/dx.doi.org/10.1111/jan.12200) | | Sleep duration/ quality not reported |
| [Luo, A., Kong, W., He, H., Li, Y., & Xie, W. (2022). Status and Influencing Factors of Social Media Addiction in Chinese Medical Care Professionals: A Cross-Sectional Survey. *Frontiers in Psychology, 13.* https://doi.org/10.3389/fpsyg.2022.888714](https://doi.org/10.3389/fpsyg.2022.888714) | | Sleep duration/ quality not reported |
| [Costa, G., Anelli, M. M., Castellini, G., Fustinoni, S., & Neri, L. (2014). Stress and sleep in nurses employed in "3x8" and "2x12" fast rotating shift schedules. *Chronobiology International, 31*(10), 1169-1178. https://doi.org/https://dx.doi.org/10.3109/07420528.2014.957309](https://doi.org/https:/dx.doi.org/10.3109/07420528.2014.957309) | | Sleep duration/ quality not reported |
| [De Carvalho, A. E. L., da Silva Frazão, I., da Silva, D. M. R., Andrade, M. S., Vasconcelos, S. C., & de Aquino, J. M. (2020). Stress of nursing professionals working in pre-hospital care. *Revista Brasileira de Enfermagem, 73*(2). https://doi.org/10.1590/0034-7167-2018-0660](https://doi.org/10.1590/0034-7167-2018-0660) | | Sleep duration/ quality not reported |
| [Bernburg, M., Hetzmann, M. S., Mojtahedzadeh, N., Neumann, F. A., Augustin, M., Harth, V., Groneberg, D. A., Zyriax, B.-C., & Mache, S. (2021). Stress Perception, Sleep Quality and Work Engagement of German Outpatient Nurses during the COVID-19 Pandemic. *International Journal of Environmental Research and Public Health, 19*(1). https://doi.org/https://dx.doi.org/10.3390/ijerph19010313](https://doi.org/https:/dx.doi.org/10.3390/ijerph19010313) | | Sleep duration/ quality not reported |
| [Sigursteinsdottir, H., Skuladottir, H., Agnarsdottir, T., & Halldorsdottir, S. (2020). Stressful factors in the working environment, lack of adequate sleep, and musculoskeletal pain among nursing unit managers. *International Journal of Environmental Research and Public Health, 17*(2), 673. https://doi.org/https://dx.doi.org/10.3390/ijerph17020673](https://doi.org/https:/dx.doi.org/10.3390/ijerph17020673) | | Sleep duration/ quality not reported |
| [Liu, H., Fan, J., Tao, X., Zhan, Y., Huang, L., & Wang, G. (2021). Suboptimal health status of nurses in Wuhan, China during the COVID-19 outbreak. *Revista da Associacao Medica Brasileira, 67*(7), 1010-1014. https://doi.org/https://dx.doi.org/10.1590/1806-9282.20210449](https://doi.org/https:/dx.doi.org/10.1590/1806-9282.20210449) | | Sleep duration/ quality not reported |
| [Taguchi, T., Hayashi, A., & Nakamori, M. (2011). Survey of actual sleeping conditions in medical university students: Comparison of nursing students with those of other faculties. *Sleep and Biological Rhythms, 9*(4), 404-405. https://doi.org/https://dx.doi.org/10.1111/j.1479-8425.2011.00518.x](https://doi.org/https:/dx.doi.org/10.1111/j.1479-8425.2011.00518.x) | | Sleep duration/ quality not reported |
| [Alyahya, M. S., Hijazi, H. H., Alolayyan, M. N., Ajayneh, F. J., Khader, Y. S., & Al-Sheyab, N. A. (2021). The association between cognitive medical errors and their contributing organizational and individual factors. *Risk Management and Healthcare Policy, 14,* 415-430. https://doi.org/https://dx.doi.org/10.2147/RMHP.S293110](https://doi.org/https:/dx.doi.org/10.2147/RMHP.S293110) | | Sleep duration/ quality not reported |
| [Alreshidi, S. M., & Rayani, A. M. (2023). The Correlation Between Night Shift Work Schedules, Sleep Quality, and Depression Symptoms. *Neuropsychiatric Disease and Treatment, 19,* 1565-1571. https://doi.org/https://dx.doi.org/10.2147/NDT.S421092](https://doi.org/https:/dx.doi.org/10.2147/NDT.S421092) | | Sleep duration/ quality not reported |
| [Samrkhanova, I. I., Kadyrov, R. F., & Efremov, I. S. (2022). The Correlation between Psychopathological Syndromes and Insomnia of Medical Students Working as Nurses. *Psychiatry (Moscow), 20*(2-3), 137-138. https://www.scopus.com/inward/record.uri?eid=2-s2.0-85141806513&partnerID=40&md5=b5547dc08891e5e10a59b8dd059e6060](https://www.scopus.com/inward/record.uri?eid=2-s2.0-85141806513&partnerID=40&md5=b5547dc08891e5e10a59b8dd059e6060) | | Sleep duration/ quality not reported |
| [Ruiz-Zaldibar, C., Gal-Iglesias, B., Azpeleta-Noriega, C., Ruiz-Lopez, M., & Perez-Manchon, D. (2022). The Effect of a Sleep Intervention on Sleep Quality in Nursing Students: Study Protocol for a Randomized Controlled Trial. *International Journal of Environmental Research and Public Health, 19*(21), 13886. https://doi.org/https://dx.doi.org/10.3390/ijerph192113886](https://doi.org/https:/dx.doi.org/10.3390/ijerph192113886) | | Sleep duration/ quality not reported |
| [Karimi, N., Hasanvand, S., Beiranvand, A., Gholami, M., & Birjandi, M. (2024). The effect of Aromatherapy with Pelargonium graveolens (P. graveolens) on the fatigue and sleep quality of critical care nurses during the Covid-19 pandemic: A randomized controlled trial. *Explore, 20*(1), 82-88. https://doi.org/https://dx.doi.org/10.1016/j.explore.2023.06.006](https://doi.org/https:/dx.doi.org/10.1016/j.explore.2023.06.006) | | Sleep duration/ quality not reported |
| [Curuk, G. N., Ozgul, E., & Karadag, S. (2023). The effect of COVID-19 on fear, anxiety, and sleep in nursing students. *Irish Journal of Medical Science, 192*(6), 3125-3131. https://doi.org/https://dx.doi.org/10.1007/s11845-023-03308-7](https://doi.org/https:/dx.doi.org/10.1007/s11845-023-03308-7) | | Sleep duration/ quality not reported |
| [Seo, M. S., & Park, D. (2017). The effect of inhaling aromas on the quality of sleep and fatigue of three shift working nurses. *Journal of Engineering and Applied Sciences, 12(*14), 3612-3615. https://doi.org/10.3923/jeasci.2017.3612.3615](https://doi.org/10.3923/jeasci.2017.3612.3615) | | Sleep duration/ quality not reported |
| [Karabulut, N., Gurcayir, D., Yaman Aktas, Y., Kara, A., Kiziloglu, B., Arslan, B., & Bolukbas, N. (2021). The effect of perceived stress on anxiety and sleep quality among healthcare professionals in intensive care units during the coronavirus pandemic. *Psychology, health & medicine, 26(*1), 119-130. https://doi.org/https://dx.doi.org/10.1080/13548506.2020.1856897](https://doi.org/https:/dx.doi.org/10.1080/13548506.2020.1856897) | | Sleep duration/ quality not reported |
| [Guyette, F. X., Morley, J. L., Weaver, M. D., Patterson, P. D., & Hostler, D. (2013). The effect of shift length on fatigue and cognitive performance in air medical providers. P*rehospital emergency care : official journal of the National Association of EMS Physicians and the National Association of State EMS Directors, 17(*1), 23-28. https://doi.org/http://dx.doi.org/10.3109/10903127.2012.710719](https://doi.org/http:/dx.doi.org/10.3109/10903127.2012.710719) | | Sleep duration/ quality not reported |
| [Han, S., & Kwak, S. (2022). The effect of sleep disturbance on the association between work-family conflict and burnout in nurses: a cross-sectional study from South Korea. *BMC Nursing, 21*(1), 354. https://doi.org/https://dx.doi.org/10.1186/s12912-022-01114-7](https://doi.org/https:/dx.doi.org/10.1186/s12912-022-01114-7) | | Sleep duration/ quality not reported |
| [Quronfulah, B. S., Aboalshamat, K. T., Badri, H. M., Mahmoud, M. A., Rajeh, M. T., Badawoud, A. M., Alzhrani, A. M., & Abdouh, I. M. (2023). The Effect of Sleep Quality on Psychological Distress Among Saudi Healthcare Students and Professionals. *International Journal of Pharmaceutical Research and Allied Sciences, 12*(3), 52-60. https://doi.org/https://dx.doi.org/10.51847/V7W6UQEKye](https://doi.org/https:/dx.doi.org/10.51847/V7W6UQEKye) | | Sleep duration/ quality not reported |
| [Gillet, N., Huyghebaert-Zouaghi, T., Reveillere, C., Colombat, P., & Fouquereau, E. (2020). The effects of job demands on nurses' burnout and presenteeism through sleep quality and relaxation. *Journal of Clinical Nursing, 29(*3-4), 583-592. https://doi.org/https://dx.doi.org/10.1111/jocn.15116](https://doi.org/https:/dx.doi.org/10.1111/jocn.15116) | | Sleep duration/ quality not reported |
| [Jung, M., Yim, S. Y., & Ryu, Y. M. (2024). The Impact of COVID-19 Stress, Interpersonal Relations, and Information Literacy on the Adaptation of Nursing Students to College Life. *Journal of Korean Academy of Psychiatric and Mental Health Nursing, 33(*1), 9-17. https://doi.org/10.12934/jkpmhn.2024.33.1.9](https://doi.org/10.12934/jkpmhn.2024.33.1.9) | | Sleep duration/ quality not reported |
| [Tuckett, A., & Henwood, T. (2015). The impact of five lifestyle factors on nurses' and midwives' health: The Australian and New Zealand nurses' and midwives' e-cohort study. *International Journal of Health Promotion and Education, 53*(3), 156-168. https://doi.org/https://dx.doi.org/10.1080/14635240.2014.978949](https://doi.org/https:/dx.doi.org/10.1080/14635240.2014.978949) | | Sleep duration/ quality not reported |
| [Ljevak, I., Vasilj, I., Lesko, J., Neuberg, M., Peric, O., & Curlin, M. (2021). THE IMPACT OF SHIFT WORK ON THE METABOLISM AND CIRCADIAN RHYTHM IN NURSES AND MEDICAL TECHNICIANS. *Acta Clinica Croatica, 60*(3), 476-482. https://doi.org/https://dx.doi.org/10.20471/acc.2021.60.03.19](https://doi.org/https:/dx.doi.org/10.20471/acc.2021.60.03.19) | | Sleep duration/ quality not reported |
| [Ferri, P., Guadi, M., Marcheselli, L., Balduzzi, S., Magnani, D., & Di Lorenzo, R. (2016). The impact of shift work on the psychological and physical health of nurses in a general hospital: A comparison between rotating night shifts and day shifts. *Risk Management and Healthcare Policy, 9*, 203-211. https://doi.org/https://dx.doi.org/10.2147/RMHP.S115326](https://doi.org/https:/dx.doi.org/10.2147/RMHP.S115326) | | Sleep duration/ quality not reported |
| [Geiger-Brown, J., Trinkoff, A., & Rogers, V. E. (2011). The impact of work schedules, home, and work demands on self-reported sleep in registered nurses. *Journal of Occupational and Environmental Medicine, 53*(3), 303-307. https://doi.org/https://dx.doi.org/10.1097/JOM.0b013e31820c3f87](https://doi.org/https:/dx.doi.org/10.1097/JOM.0b013e31820c3f87) | | Sleep duration/ quality not reported |
| [Khamis, F., Al Mahyijari, N., Al Lawati, F., & Badahdah, A. M. (2020). The Mental Health of Female Physicians and Nurses in Oman during the COVID-19 Pandemic. *Oman medical journal, 35*(6), e203. https://doi.org/https://dx.doi.org/10.5001/omj.2020.103](https://doi.org/https:/dx.doi.org/10.5001/omj.2020.103) | | Sleep duration/ quality not reported |
| [Haun, V. C., & Baethge, A. (2020). The Moderating Role of Work-Related Rumination in Nurses' Sleep Quality Trajectory during Morning Shift Work. *Journal of Personnel Psychology, 19*(4), 151-163. https://doi.org/10.1027/1866-5888/a000247](https://doi.org/10.1027/1866-5888/a000247) | | Sleep duration/ quality not reported |
| [Biddle, C., & Aker, J. (2011). The national study of sleep-related behaviors of nurse anesthetists: Personal and professional implications. *AANA Journal, 79(*4), 324-331. http://www.aana.com/WorkArea/DownloadAsset.aspx?id=31434http://ovidsp.ovid.com/ovidweb.cgi?T=JS&PAGE=reference&D=emed12&NEWS=N&AN=362366397](http://www.aana.com/WorkArea/DownloadAsset.aspx?id=31434http://ovidsp.ovid.com/ovidweb.cgi?T=JS&PAGE=reference&D=emed12&NEWS=N&AN=362366397) | | Sleep duration/ quality not reported |
| [Chen, C. J., Chen, Y. C., Sung, H. C., Hsieh, T. C., Lee, M. S., & Chang, C. Y. (2015). The prevalence and related factors of depressive symptoms among junior college nursing students: a cross-sectional study. *Journal of psychiatric and mental health nursing, 22*(8), 590-598. https://doi.org/https://dx.doi.org/10.1111/jpm.12252](https://doi.org/https:/dx.doi.org/10.1111/jpm.12252) | | Sleep duration/ quality not reported |
| [Blundell, A. R., Font, C. M., Figueredo, L., Gordon, A., Casas, C., Colon, P., Gutierrez, A., Perez, J., & Colon-Diaz, M. (2023). The Prevalence of Depression Symptoms and Frequency of Risk Factors in Medical and Nursing Students: A Descriptive Cross-sectional Study. *Puerto Rico health sciences journal, 42*(1), 70-76. http://ovidsp.ovid.com/ovidweb.cgi?T=JS&PAGE=reference&D=emexb&NEWS=N&AN=640833588](http://ovidsp.ovid.com/ovidweb.cgi?T=JS&PAGE=reference&D=emexb&NEWS=N&AN=640833588) | | Sleep duration/ quality not reported |
| [An, F.-R., Qi, Y.-K., Zeng, J.-Y., Ding, Y.-M., Chiu, H. F. K., Ungvari, G. S., Newhouse, R., Yu, D. S. F., Lai, K. Y. C., & Xiang, Y.-T. (2016). The prevalence of insomnia, its demographic correlates, and treatment in nurses working in Chinese psychiatric and general hospitals. *Perspectives in Psychiatric Care, 52*(2), 88-94. https://doi.org/https://dx.doi.org/10.1111/ppc.12103](https://doi.org/https:/dx.doi.org/10.1111/ppc.12103) | | Sleep duration/ quality not reported |
| [Scott, L. D., Hwang, W.-T., Rogers, A. E., Nysse, T., Dean, G. E., & Dinges, D. F. (2007). The relationship between nurse work schedules, sleep duration, and drowsy driving. *Sleep, 30*(12), 1801-1807. https://doi.org/https://dx.doi.org/10.1093/sleep/30.12.1801](https://doi.org/https:/dx.doi.org/10.1093/sleep/30.12.1801) | | Sleep duration/ quality not reported |
| [Hatukay, A. L., Shochat, T., Zion, N., Baruch, H., Cohen, R., Azriel, Y., & Srulovici, E. (2024). The relationship between quick return shift schedules and burnout among nurses: A prospective repeated measures multi-source study. *International Journal of Nursing Studies, 151,* 104677. https://doi.org/https://dx.doi.org/10.1016/j.ijnurstu.2023.104677](https://doi.org/https:/dx.doi.org/10.1016/j.ijnurstu.2023.104677) | | Sleep duration/ quality not reported |
| [Hou, X., He, Y., Chen, F., Li, Y., Wu, M., Chen, K., & Zhou, Z. (2024). The relationship between the frequency of headaches associated with the personal protective equipment and its influencing factors is mediated by depression: A cross-sectional study. *Heliyon, 10*(3). https://doi.org/10.1016/j.heliyon.2024.e24744](https://doi.org/10.1016/j.heliyon.2024.e24744) | | Sleep duration/ quality not reported |
| [Azmoon, H., Dehghan, H., Akbari, J., & Souri, S. (2013). The relationship between thermal comfort and light intensity with sleep quality and eye tiredness in shift work nurses. *Journal of environmental and public health, 2013*, 639184. http://ovidsp.ovid.com/ovidweb.cgi?T=JS&PAGE=reference&D=emed14&NEWS=N&AN=369361270](http://ovidsp.ovid.com/ovidweb.cgi?T=JS&PAGE=reference&D=emed14&NEWS=N&AN=369361270) | | Sleep duration/ quality not reported |
| [Asci, O., Gokdemir, F., Kahyaoglu Sut, H., & Payam, F. (2015). The Relationship of Premenstrual Syndrome Symptoms with Menstrual Attitude and Sleep Quality in Turkish Nursing Student. *Journal of caring sciences, 4(*3), 179-187. https://doi.org/https://dx.doi.org/10.15171/jcs.2015.018](https://doi.org/https:/dx.doi.org/10.15171/jcs.2015.018) | | Sleep duration/ quality not reported |
| [Zhou, Y., Wang, S., Liu, M., Gan, G., Qin, N., Luo, X., Zhang, C., Xie, J., Wang, K., & Cheng, A. S. (2023). The role of sleep quality and perceived stress on depressive symptoms among tertiary hospital nurses: a cross-sectional study. *BMC Psychiatry, 23*(1), 416. https://doi.org/https://dx.doi.org/10.1186/s12888-023-04936-0](https://doi.org/https:/dx.doi.org/10.1186/s12888-023-04936-0) | | Sleep duration/ quality not reported |
| [Tang, C., Zhou, S., Liu, C., Min, R., Cai, R., & Lin, T. (2023). Turnover intention of nurses in public hospitals and its association with quality of working life: a cross-sectional survey in six provinces in China. *Frontiers in Public Health, 11*, 1305620. https://doi.org/https://dx.doi.org/10.3389/fpubh.2023.1305620](https://doi.org/https:/dx.doi.org/10.3389/fpubh.2023.1305620) | | Sleep duration/ quality not reported |
| Caboral-Stevens, M., Raymond, D., & Evangelista, L. S. (2023). Well-Being, Occupational Fatigue, and Sleep Quality among Filipino Nurses working during COVID-19. *Asian journal of nursing education and research, 13*(1), 67-72. https://doi.org/https://dx.doi.org/10.52711/2349-2996.2023.00016 | | Sleep duration/ quality not reported |
| [Strout, K., Schwartz-Mette, R., McNamara, J., Parsons, K., Walsh, D., Bonnet, J., O'Brien, L. M., Robinson, K., Sibley, S., Smith, A., Sapp, M., Sprague, L., Sabegh, N. S., & Henderson, A. (2023). Wellness in Nursing Education to Promote Resilience and Reduce Burnout: Protocol for a Holistic Multidimensional Wellness Intervention and Longitudinal Research Study Design in Nursing Education. *JMIR Research Protocols, 12.* https://doi.org/10.2196/49020](https://doi.org/10.2196/49020) | | Sleep duration/ quality not reported |
| [Choi, Y. J., Um, Y. J., & Cho, D. H. (2023). What influenced frontline nurses' mental health during the early phase of the COVID-19 pandemic*. International nursing review, 70*(4), 589-595. https://doi.org/https://dx.doi.org/10.1111/inr.12895](https://doi.org/https:/dx.doi.org/10.1111/inr.12895) | | Sleep duration/ quality not reported |
| [Wangsan, K., Chaiear, N., Sawanyawisuth, K., Klainin-Yobas, P., Simajareuk, K., & Boonsawat, W. (2022). Which Shiftwork Pattern Is the Strongest Predictor for Poor Sleep Quality in Nurses? *International Journal of Environmental Research and Public Health, 19(*21), 13986. https://doi.org/https://dx.doi.org/10.3390/ijerph192113986](https://doi.org/https:/dx.doi.org/10.3390/ijerph192113986) | | Sleep duration/ quality not reported |
| [Li, Y., Fang, J., & Zhou, C. (2019). Work-Related Predictors of Sleep Quality in Chinese Nurses: Testing a Path Analysis Model. *The journal of nursing research : JNR, 27(*5), e44. https://doi.org/https://dx.doi.org/10.1097/jnr.0000000000000319](https://doi.org/https:/dx.doi.org/10.1097/jnr.0000000000000319) | | Sleep duration/ quality not reported |
| [Crincoli, S., de Cordova, P., Thomas-Hawkins, C., Flynn, L., Zha, P., & Sagherian, K. (2024). The Effects of Organizational Characteristics, Individual Nurse Characteristics, and Occupational Fatigue on Missed Care at Night. *Nursing Research, 73*(2), 101-108. https://doi.org/https://dx.doi.org/10.1097/NNR.0000000000000696](https://doi.org/https:/dx.doi.org/10.1097/NNR.0000000000000696) | | Sleep duration/ quality not reported |
| [Chiang, H. J., Tsai, L. L., & Weng, C. Y. (2009). Subjective sleep quality an D sleep problems in Taiwan nurses working on rotating shifts. *Sleep and Biological Rhythms, 7(*4), A22. https://doi.org/https://dx.doi.org/10.1111/j.1479-8425.2009.00426.x](https://doi.org/https:/dx.doi.org/10.1111/j.1479-8425.2009.00426.x) | | Sleep duration/ quality not reported |
| [Vevodova, S., Petrasova, B., Vevoda, J., Borikova, A., & Nakladalova, M. (2016). The impact of shift work on sleep and anxiety in general nurses. *Pracovni Lekarstvi, 68*(3), 103-109. http://www.prolekare.cz/pracovni-lekarstvi-archiv-ciselhttp://ovidsp.ovid.com/ovidweb.cgi?T=JS&PAGE=reference&D=emca3&NEWS=N&AN=614031348](http://www.prolekare.cz/pracovni-lekarstvi-archiv-ciselhttp:/ovidsp.ovid.com/ovidweb.cgi?T=JS&PAGE=reference&D=emca3&NEWS=N&AN=614031348) | | Sleep duration/ quality not reported |
| [Abbas, A., Ali, A., Bahgat, S., & Shouman, W. (2019). Prevalence, associated factors, and consequences of burnout among ICU healthcare workers: An Egyptian experience. *Egyptian Journal of Chest Diseases and Tuberculosis, 68*(4), 514-525. https://doi.org/https://dx.doi.org/10.4103/ejcdt.ejcdt_188_18](https://doi.org/https:/dx.doi.org/10.4103/ejcdt.ejcdt_188_18) | | Sleep duration/ quality not reported |
| [Kang, J., Noh, W., & Lee, Y. (2020). Sleep quality among shift-work nurses: A systematic review and meta-analysis. *Applied nursing research : ANR, 52*, 151227. https://doi.org/https://dx.doi.org/10.1016/j.apnr.2019.151227](https://doi.org/https:/dx.doi.org/10.1016/j.apnr.2019.151227) | | Not a primary study (systematic review and meta-analysis) |
| [Gerace, A., & Rigney, G. (2020). Considering the relationship between sleep and empathy and compassion in mental health nurses: It's time. *International Journal of Mental Health Nursing, 29*(5), 1002-1010. https://doi.org/https://dx.doi.org/10.1111/inm.12734](https://doi.org/https:/dx.doi.org/10.1111/inm.12734) | | Not a primary study (literature commentary) |
| [Bell, T., Sprajcer, M., Flenady, T., & Sahay, A. (2023). Fatigue in nurses and medication administration errors: A scoping review. *Journal of Clinical Nursing, 32*(17-18), 5445-5460. https://doi.org/https://dx.doi.org/10.1111/jocn.16620](https://doi.org/https:/dx.doi.org/10.1111/jocn.16620) | | Not a primary study (scoping review) |
| [Booker, L. A., Magee, M., Rajaratnam, S. M. W., Sletten, T. L., & Howard, M. E. (2018). Individual vulnerability to insomnia, excessive sleepiness and shift work disorder amongst healthcare shift workers. A systematic review. *Sleep Medicine Reviews, 41*, 220-233. https://doi.org/https://dx.doi.org/10.1016/j.smrv.2018.03.005](https://doi.org/https:/dx.doi.org/10.1016/j.smrv.2018.03.005) | | Not a primary study (systematic review) |
| [Chang, W.-P., & Peng, Y.-X. (2021). Influence of rotating shifts and fixed night shifts on sleep quality of nurses of different ages: A systematic literature review and meta-analysis. *Chronobiology International, 38*(10), 1384-1396. https://doi.org/https://dx.doi.org/10.1080/07420528.2021.1931273](https://doi.org/https:/dx.doi.org/10.1080/07420528.2021.1931273) | | Not a primary study (systematic literature review and meta-analysis) |
| [Moitra, M., Rahman, M., Collins, P. Y., Gohar, F., Weaver, M., Kinuthia, J., Rossler, W., Petersen, S., Unutzer, J., Saxena, S., Huang, K. Y., Lai, J., & Kumar, M. (2021). Mental Health Consequences for Healthcare Workers During the COVID-19 Pandemic: A Scoping Review to Draw Lessons for LMICs. *Frontiers in Psychiatry, 12,* 602614. https://doi.org/https://dx.doi.org/10.3389/fpsyt.2021.602614](https://doi.org/https:/dx.doi.org/10.3389/fpsyt.2021.602614) | | Not a primary study (scoping review) |
| [Zeng, L.-N., Yang, Y., Wang, C., Li, X.-H., Xiang, Y.-F., Hall, B. J., Ungvari, G. S., Li, C.-Y., Chen, C., Chen, L.-G., Cui, X.-L., An, F.-R., & Xiang, Y.-T. (2020). Prevalence of poor sleep quality in nursing staff: A meta-analysis of observational studies. *Behavioral Sleep Medicine, 18*(6), 746-759. https://doi.org/https://dx.doi.org/10.1080/15402002.2019.1677233](https://doi.org/https:/dx.doi.org/10.1080/15402002.2019.1677233) | | Not a primary study (systematic literature review and meta-analysis) |
| [Al Maqbali, M., Al Sinani, M., & Al-Lenjawi, B. (2021). Prevalence of stress, depression, anxiety and sleep disturbance among nurses during the COVID-19 pandemic: A systematic review and meta-analysis. *Journal of Psychosomatic Research, 141.* https://doi.org/10.1016/j.jpsychores.2020.110343](https://doi.org/10.1016/j.jpsychores.2020.110343) | | Not a primary study (systematic literature review and meta-analysis) |
| [Membrive-Jiménez, M. J., Gómez-Urquiza, J. L., Suleiman-Martos, N., Velando-Soriano, A., Ariza, T., De la Fuente-Solana, E. I., & Cañadas-De la Fuente, G. A. (2022). Relation between Burnout and Sleep Problems in Nurses: A Systematic Review with Meta-Analysis. H*ealthcare (Switzerland), 10*(5). https://doi.org/10.3390/healthcare10050954](https://doi.org/10.3390/healthcare10050954) | | Not a primary study (systematic literature review and meta-analysis) |
| [Alanazi, T. N. M., McKenna, L., Buck, M., & Alharbi, R. J. (2022). Reported effects of the COVID-19 pandemic on the psychological status of emergency healthcare workers: A scoping review. *Australasian emergency care, 25*(3), 197-212. https://doi.org/https://dx.doi.org/10.1016/j.auec.2021.10.002](https://doi.org/https:/dx.doi.org/10.1016/j.auec.2021.10.002) | | Not a primary study (scoping review) |
| [Martin, C. V., Joyce-McCoach, J., Peddle, M., & East, C. E. (2024). Sleep deprivation and medication administration errors in registered nurses-A scoping review. *Journal of Clinical Nursing, 33*(3), 859-873. https://doi.org/https://dx.doi.org/10.1111/jocn.16912](https://doi.org/https:/dx.doi.org/10.1111/jocn.16912) | | Not a primary study (scoping review) |
| [Sayehmiri, K., & Mohammadi, E. (2018). Sleep disturbances among nurses in Iran: A meta-analysis. *Iranian Red Crescent Medical Journal, 20*(11), e62089. https://doi.org/https://dx.doi.org/10.5812/ircmj.62089](https://doi.org/https:/dx.doi.org/10.5812/ircmj.62089) | | Not a primary study (systematic literature review and meta-analysis) |
| [Ribeiro, Í. A. P., de Oliveira, A. L. C. B., Feitosa, C. D. A., Pillon, S. C., Marziale, M. H. P., & Fernandes, M. A. (2023). Sleep quality of nurses who worked in coping with COVID-19: an integrative review. *Revista Brasileira de Enfermagem, 76*(6). https://doi.org/10.1590/0034-7167-2023-0007](https://doi.org/10.1590/0034-7167-2023-0007) | | Not a primary study (integrative review) |
| [Aemmi, S. Z., Mohammadi, E., Heidarian-Miri, H., Fereidooni-Moghadam, M., Boostani, H., & Zarea, K. (2020). The effectiveness of bright light exposure in shift-worker nurses: A systematic review and meta-analysis. *Sleep Science, 13*(2), 145-151. https://doi.org/https://dx.doi.org/10.5935/1984-0063.20190137](https://doi.org/https:/dx.doi.org/10.5935/1984-0063.20190137) | | Not a primary study (systematic literature review and meta-analysis) |
| [Oamen, B. R., Jordan, P., & Iwu-Jaja, C. (2023). The impact of COVID-19 on the mental health of healthcare workers in Africa: a scoping review. *Pan African Medical Journal One Health, 12.* https://doi.org/10.11604/pamj-oh.2023.12.7.40107](https://doi.org/10.11604/pamj-oh.2023.12.7.40107) | | Not a primary study (scoping review) |
| [Salari, N., Khazaie, H., Hosseinian-Far, A., Ghasemi, H., Mohammadi, M., Shohaimi, S., Daneshkhah, A., Khaledi-Paveh, B., & Hosseinian-Far, M. (2020). The prevalence of sleep disturbances among physicians and nurses facing the COVID-19 patients: A systematic review and meta-analysis. *Globalization and Health, 16*(1), 92. https://doi.org/https://dx.doi.org/10.1186/s12992-020-00620-0](https://doi.org/https:/dx.doi.org/10.1186/s12992-020-00620-0) | | Not a primary study (systematic literature review and meta-analysis) |
| [Stanojevic, C., Simic, S., & Milutinovic, D. (2016). HEALTH EFFECTS OF SLEEP DEPRIVATION ON NURSES WORKING SHIFTS. *Medicinski pregled, 69*(5-6), 183-188. https://doi.org/https://dx.doi.org/10.2298/MPNS1606183S](https://doi.org/https:/dx.doi.org/10.2298/MPNS1606183S) | | Not a primary study (narrative review) |
| [Zion, N., Drach-Zahavy, A., & Shochat, T. (2018). Who is sleepier on the night shift? The influence of bio-psycho-social factors on subjective sleepiness of female nurses during the night shift. *Ergonomics, 61*(7), 1004-1014. https://doi.org/https://dx.doi.org/10.1080/00140139.2017.1418027](https://doi.org/https:/dx.doi.org/10.1080/00140139.2017.1418027) | | Duplicate of 278 |
| [Cannici, C., Liptrott, S. J., Serra, N., Samarani, E., De Cecco, V., Caime, A., Galgano, L., Rostagno, E., Orlando, L., Gargiulo, G., Cioce, M., Lupo, R., Capuano, A., Rea, T., & Botti, S. (2023). Predictive factors of sleep quality and burnout in nurses working in transplant units during the COVID-19 lockdown - A cross-sectional study. *Contemporary nurse, 59(*4-5), 362-376. https://doi.org/https://dx.doi.org/10.1080/10376178.2023.2244090](https://doi.org/https:/dx.doi.org/10.1080/10376178.2023.2244090) | | Duplicate of 28 |
| [Dong, H., Zhang, Q., Sun, Z., Sang, F., & Xu, Y. (2017). Sleep disturbances among Chinese clinical nurses in general hospitals and its influencing factors. *BMC Psychiatry, 17*(1), 241. https://doi.org/https://dx.doi.org/10.1186/s12888-017-1402-3](https://doi.org/https:/dx.doi.org/10.1186/s12888-017-1402-3) | | Duplicate of 63 |
| [Zhang, Y., Peters, A., & Bradstreet, J. (2018). Relationships among sleep quality, coping styles, and depressive symptoms among college nursing students: A multiple mediator model. *Journal of professional nursing : official journal of the American Association of Colleges of Nursing, 34*(4), 320-325. https://doi.org/https://dx.doi.org/10.1016/j.profnurs.2017.12.004](https://doi.org/https:/dx.doi.org/10.1016/j.profnurs.2017.12.004) | | Duplicate of 253 |
| [Cox, S. D., Benoit, J. S., Brohard, C. L., & McIntyre, T. M. (2022). Evaluation of sleep quality among nursing faculty: Application of the Pittsburgh Sleep Quality Index—A descriptive correlational study. *Nursing Open, 9*(1), 339-348. https://doi.org/10.1002/nop2.1067](https://doi.org/10.1002/nop2.1067) | | Duplicate of 50 |
| [Chang, W.-P., & Li, H.-B. (2022). Influence of shift work on rest-activity rhythms, sleep quality, and fatigue of female nurses. *Chronobiology International, 39*(4), 557-568. https://doi.org/https://dx.doi.org/10.1080/07420528.2021.2005082](https://doi.org/https:/dx.doi.org/10.1080/07420528.2021.2005082) | | Duplicate of 35 |
| [Almhdawi, K. A., Alrabbaie, H., Kanaan, S. F., Oteir, A. O., Jaber, A. F., Ismael, N. T., & Obaidat, D. S. (2020). Predictors and prevalence of lower quadrant work-related musculoskeletal disorders among hospital-based nurses: A cross-sectional study. *Journal of Back and Musculoskeletal Rehabilitation, 33*(6), 885-896. https://doi.org/https://dx.doi.org/10.3233/BMR-191815](https://doi.org/https:/dx.doi.org/10.3233/BMR-191815) | | Duplicate of 11 |
| [Bazrafshan, M., Moravveji, F., Soleymaninejad, N., Zare, F., Rahimpoor, R., & Zolghadr, R. (2018). Prevalence and effects of sleep disorders among shift-working nurse. *Annals of Tropical Medicine and Public Health, 11*(1), 13-18. http://www.atmph.orghttp://ovidsp.ovid.com/ovidweb.cgi?T=JS&PAGE=reference&D=emed19&NEWS=N&AN=630312195](about:blank) | | Duplicate of 21 |
| [Wang, B., Lu, Q., Sun, F., & Zhang, R. (2021). The relationship between sleep quality and psychological distress and job burnout among chinese psychiatric nurses. *Industrial Health, 59*(6), 427-435. https://doi.org/10.2486/indhealth.2020-0249](https://doi.org/10.2486/indhealth.2020-0249) | | Duplicate of 254 |
| [Perez-Fuentes, M. C., Molero Jurado, M. M., Martin, A. B. B., Martinez, A. M., & Linares, J. J. G. (2019). Association with the quality of sleep and the mediating role of eating on self-esteem in healthcare personnel. *Nutrients, 11*(2), 321. https://doi.org/https://dx.doi.org/10.3390/nu11020321](https://doi.org/https:/dx.doi.org/10.3390/nu11020321) | | Duplicate of 189 |
| [Niu, S.-F., Chu, H., Chung, M.-H., Lin, C.-C., Chang, Y.-S., & Chou, K.-R. (2013). Sleep quality in nurses: a randomized clinical trial of day and night shift workers. *Biological Research for Nursing, 15*(3), 273-279. https://doi.org/https://dx.doi.org/10.1177/1099800412439459](https://doi.org/https:/dx.doi.org/10.1177/1099800412439459) | | Duplicate of 179 |
| [Niu, S.-F., Lin, C.-J., Chen, P.-Y., Fan, Y.-C., Huang, H.-C., & Chiu, H.-Y. (2021). Immediate and lasting effects of aerobic exercise on the actigraphic sleep parameters of female nurses: A randomized controlled trial. *Research in nursing & health, 44(*3), 449-457. https://doi.org/https://dx.doi.org/10.1002/nur.22126](https://doi.org/https:/dx.doi.org/10.1002/nur.22126) | | Duplicate of 179 |
| Farag, A., Scott, L. D., Perkhounkova, Y., Saeidzadeh, S., & Hein, M. (2022). A human factors approach to evaluate predicators of acute care nurse occupational fatigue. *Applied Ergonomics*, *100*, 103647. https://doi.org/10.1016/j.apergo.2021.103647 | | Duplicate of 81 |
| [Mu, C. X., & Lee, S. (2024). The moderating role of trait and state mindfulness between daily sleep and physical pain symptoms: an ecological momentary assessment and actigraphy study. *Psychology & health, 39*(1), 91-108. https://doi.org/https://dx.doi.org/10.1080/08870446.2022.2069245](https://doi.org/https:/dx.doi.org/10.1080/08870446.2022.2069245) | | Duplicate of 248 |
| [Slavish, D. C., Dietch, J. R., Kane, H. S., Messman, B. A., Garcia, O., Wiley, J. F., Yap, Y., Kelly, K., Ruggero, C., & Taylor, D. J. (2022). Daily stress and sleep associations vary by work schedule: A between- and within-person analysis in nurses. *Journal of Sleep Research, 31*(3), e13506. https://doi.org/https://dx.doi.org/10.1111/jsr.13506](https://doi.org/https:/dx.doi.org/10.1111/jsr.13506) | | Duplicate of 266 |
| [Garde, A. H., Hansen, A. M., & Hansen, J. (2009). Sleep length and quality, sleepiness and urinary melatonin among healthy Danish nurses with shift work during work and leisure time. *International Archives of Occupational and Environmental Health, 82*(10), 1219-1228. https://doi.org/https://dx.doi.org/10.1007/s00420-009-0419-4](https://doi.org/https:/dx.doi.org/10.1007/s00420-009-0419-4) | | Duplicate of 94 |
| [Alaradi, F. A., Priya, G., & Al-Darazi, F. (2022). Sleep Quality and Work Performance Among Shift Work Critical Care Nurses: A Cross-Sectional Study. *Bahrain Medical Bulletin, 44*(4), 1181-1188. http://www.bahrainmedicalbulletin.com/http://ovidsp.ovid.com/ovidweb.cgi?T=JS&PAGE=reference&D=emed25&NEWS=N&AN=2019043954](http://www.bahrainmedicalbulletin.com/http:/ovidsp.ovid.com/ovidweb.cgi?T=JS&PAGE=reference&D=emed25&NEWS=N&AN=2019043954) | | Duplicate of 258 |
| [Kunert, K., King, L., & Kolkhorst, F. W. (2007). Fatigue and sleep quality in nurses. *Journal of Psychosocial Nursing and Mental Health Services, 45*(8), 30-37. http://ovidsp.ovid.com/ovidweb.cgi?T=JS&PAGE=reference&D=emed10&NEWS=N&AN=47473353](http://ovidsp.ovid.com/ovidweb.cgi?T=JS&PAGE=reference&D=emed10&NEWS=N&AN=47473353) | | Duplicate of 140 |
| [Chen, H., Schernhammer, E., Schwarzschild, M. A., & Ascherio, A. (2006). A prospective study of night shift work, sleep duration, and risk of Parkinson's disease. *American Journal of Epidemiology, 163*(8), 726-730. https://doi.org/https://dx.doi.org/10.1093/aje/kwj096](https://doi.org/https:/dx.doi.org/10.1093/aje/kwj096) | | Duplicate of 108 |
| [Pan, A., Malik, V. S., Hao, T., Willett, W. C., Mozaffarian, D., & Hu, F. B. (2013). Changes in water and beverage intake and long-term weight changes: Results from three prospective cohort studies. *International Journal of Obesity, 37*(10), 1378-1385. https://doi.org/https://dx.doi.org/10.1038/ijo.2012.225](https://doi.org/https:/dx.doi.org/10.1038/ijo.2012.225) | | Duplicate of 108 |
