# Supplementary material for: Trends in Nurses’ Sleep: A Meta‐Regression Involving 219,245 Nurses From 45 Countries
Source: J Nurs Manag. 2026 Aug 27;2026:9051680. doi: 10.1155/jonm/9051680 (PMC13522381; doi:10.1155/jonm/9051680)
Supplement: Supplementary file 3 [file JONM-2026-9051680-s003.docx]

**Supplementary File 2: Data extraction protocol**

Data extraction involved viewing each of the included studies for review and recording the relevant data in an Excel spreadsheet. The first step of this process was to determine whether the full texts of the included studies were suitable and had relevant data that could be extracted. All studies required a yes/no response. If the answer was ‘no’, then a reason was provided. For example, if the study sample size was below 30, then the comment would be ‘no- the sample size was insufficient’.  An explanation of the required data and definitions is provided below.

***Bibliographic Information***

Individual columns were created to record the bibliographic information including;

*Authors:* All the authors of the study were reported

*Abstract:* The abstract of the study was reported

*Year of publication:* The year of publication was reported.

*Year of Measurement:* The year of measurement is expressed as overall annual year reported. If the measurement period was collected over different annual periods, the midpoint of the measurement period was taken (e.g., 2000 was taken as the midpoint measurement year for data collected between 1999 and 2001). If data were not provided, then the year of measurement was estimated as 2 years prior to the publication date, as this was the mean gap between measurement and publication dates for included studies.

*DOI:* The DOI of the study was reported.

*Reference:* The reference of the study was reported

*Covidence number:* The Covidence number attached to the study was reported.

***Dataset***

Three columns were created to identify duplicate datasets which are explained below;

*Duplicate:* Identify studies that share the same dataset. A yes/no was required, and for those with a yes, it was required to document the Covidence number of the duplicate study.

*Larger dataset:* Identify data that stems from a larger dataset. A simple yes/no was required in this column.

*Name of dataset:* If a larger dataset was being used, the name was reported within this column (e.g., Nurses' Health Study)

***Country***

Three columns were created to record where the study was conducted.

*Country reported:* This column reported whether the country was reported. It required a simple yes/no response. Yes, meant the country was documented. No, meant the country was assumed and based on the first author’s affiliation. Both yes and no responses were not to be capitalized.

*Country:* This column was to be used to document the country in which the study was conducted. The country's name was capitalized, and the United States was reported as USA and the United Kingdom was reported as UK.

*Continent:* Documented the continent on which the study was conducted. The continent was capitalized.

*Geographical region*: Guided by the World Health Organisation, the continental region documented included: the African Region. (AFR), Region of the Americas (AMR), Eastern Mediterranean Region (EMR), European Region (EUR), South-East Asia Region (SEAR) and Western Pacific Region (WPR). The applicable acronym was documented for each study.

***Study Design***

A column was created to record the study's design. The designs included primary studies including observational (e.g., cross-sectional, case-control, and cohort studies), longitudinal, and intervention (e.g., experimental, and quasi-experimental). For cross-sectional and cohort studies, only baseline data and refreshment sample data were extracted. In the case of experimental and longitudinal studies, only baseline data were extracted. The response was not to be capitalized.

***Nursing Characteristics***

*Nurse type:* The nurse descriptor was developed following review of several papers to determine how studies described samples of nurses. A list was developed and exclusively included nurse, retiree, and manager.

*Nurse specialization number:* Where available, the number of nurse specialties was reported. For instance, if a study examined nurses working within two settings this would be recorded as ‘2’. If the number exceeds 4, ‘4+’ was documented.

*Nurse specialization:* Where available, the nurse specialties were reported (e.g., ICU or oncology). If the types of nurse specialization were more than one, then ‘multiple’ was documented.

*Shift Work:* If shift work was documented, the type of shift was to be described in this column. Shift types were collapsed into two categories. (1) Works night hours (i.e., participants who worked night-time hours, whether as part of a fixed, rotating, or shift work schedule). (2) Works non-night hours (i.e., participants who did not work night-time hours, which included daytime and evening shifts). When a study provided a mixed sample, the majority category was used for reporting (e.g., if 80% of the sample worked non-night shifts and 20% worked night shifts, the study would be reported as involving ‘works non-night hours’)

*Years of nursing experience (mean):* Where available, the mean years of nursing experience were to be recorded.

*Years of nursing experience (standard deviation):* Where available, the standard deviation

of the years of nursing experience were recorded.

*Years of nursing experience (other):* If the mean and standard deviation were not reported but other summary statistics were used, then these details were documented within this separate column (e.g., if 50% of participants had <15 years of experience and 50% had >15 year of experience, it was reported as ‘50% <15 yrs; 50% >15 yrs’)

All worded responses were not capitalized.

***Sample Characteristics***

Septate columns were created to record the information listed below;

*Sample size:* The sample size of each study was recorded. If the sample size included non-nurse personnel, only the nursing sample was to be reported.

*Age(mean):* Reported the mean age of the participants.

*Age (SD):* Reported the standard deviation of the participant's age.

*Age (range):* Reported the age range of the participants (e.g., 25 – 40)

*Age (other):* If the mean age was not reported but other summary statistics were used, such as 50% of participants were aged < 30, then these details were recorded (e.g., 50% <30 yrs)

*Sex:* The percentage of the sample who were female was recorded (e.g., if 75% of the sample were female, then 75 would be reported).

***Sample Groups***

If a study measured sleep for separate groups (e.g., night shift and day shift workers), these were recorded and entered as individual study entries. If the separated groups had a sample size <30, then they were to be reported together considering the groups combined made a sample >30. (e.g., night shift workers n=20, day shift workers n=31. In this example, the sample size of one of the groups was below 30, but the combined sample size is greater than 30. Therefore, only the total sample would be reported). Alongside the individual groups, the total sample was reported for all studies, and information would be repeated from the previous section.  For each entry, the sample characteristics below were reported;

*Grouping:* A description of the separate samples was recorded (e.g., if a study looked at sleep duration for night shift workers and non-night shift workers, the groups would be titled ‘night shift workers’ and ‘non-night shift workers’). If no separate groupings were documented/used, the total group was to be documented and labelled as 'total’.

*Sample size (n(group)):* The sample size of each separate group was recorded. If a separate sample size was below 30, then only the studies ‘total’ group was to be reported.

*Age (mean):* The mean age of the participants within the separate samples was recorded.

*Sex:* The percentage of females within each separate sample was recorded.

*Year the study was conducted:* If documented, the year the data was conducted and collected was recorded (e.g., if the data was collected in 2018, then ‘2018’ would be reported). If a study did not report the data collection year, 2 years were to be taken from the publication data (e.g., publication data 2018, then ‘2016’ was to be reported). The 2-year estimate was based on averaging the total time from the data collection date to the publication date across the other studies.

*10-year Increments:* Studies were divided into 4 subgroups with 10-year increments for the year the study was conducted. The breakdown is as follows; 1= 1985- 1999, 2 = 2000 – 2009, 3 = 2010 – 2019 and 4 = 2020 +.

***Sleep Parameter Assessed***

A column for the sleep parameter assessed in the studies was created. The options within this analysis were sleep quality and/or sleep duration. If a study measured sleep quality, then ‘quality’ would be reported. If duration was measured, then ‘duration’ would be reported. If both were measured, then ‘both’ would be reported.

***Sleep Duration***

*Measure:* A column for the tool used to measure sleep duration was included. A list was created including sleep reports (SR) (e.g., questionnaires and logbooks) and actigraphy (ACT).

*Period:* The period over which the data was collected was reported (e.g., if the data was collected over a one-week period, '1 week’ was reported. If the sleep duration was averaged, then ‘average’ would be reported).

*Statistics:* Only continuous statistics of sleep duration operationalised as total sleep time (TST) (e.g., time in bed minus latency or responses to questions such as ‘how long do you sleep on average’) are to be reported. If not explicitly reported, assumptions can be made based on the information provided.

*Sleep duration (mean):* The mean (hours) sleep duration was reported.

*Sleep duration (standard deviation):* The standard deviation (SD, hours) of sleep duration was reported. If the standard deviation was not reported, the estimated weighted SD score of 0.159 was to be multiplied by the provided mean (e.g., provided mean is 7.4. Therefore, 7.4 x 0.159 = 1.18. The SD reported is 1.18).

*Sleep duration notes:* Additional notes were reported (e.g., sleep duration median).

***Sleep Quality***

*Measure:* A column for the tool used to measure sleep quality was included. Although the Pittsburgh Sleep Quality (PSQI) was the only tool included, it has been translated into different languages, which needed to be recorded. For instance, if a study used the Chinese PSQI, it would be recorded as ‘PSQI-Chinese’.

*Sleep quality (mean):* The global mean PSQI score was reported.

*Sleep quality (standard deviation):* The standard deviation of the global PSQI score was reported.  If the standard deviation was not reported, the estimated weighted SD score of 0.441 was to be multiplied by the provided mean (e.g., provided mean is 7.4. Therefore, 7.4 x 0.441 = 3.26. The SD reported is 3.26).

*Cut of score:* The cut of score used to indicate poor sleep quality was recorded. If a study had a PSQI cut off score of ‘>5’, then it would be reported within the column as such.

*Sleep quality notes:* Additional notes were reported (e.g., the median scores for the global PSQI score)

For all columns, for information that is not reported (NR), NR was to be documented
