# Supplementary material for: Trends in Nurses’ Sleep: A Meta‐Regression Involving 219,245 Nurses From 45 Countries
Source: J Nurs Manag. 2026 Aug 27;2026:9051680. doi: 10.1155/jonm/9051680 (PMC13522381; doi:10.1155/jonm/9051680)
Supplement: Supplementary file 4 [file JONM-2026-9051680-s004.docx]

| **Supplementary File 5: Extracted data of all included studies** | | | | | | | | | | | | |
| --- | --- | --- | --- | --- | --- | --- | --- | --- | --- | --- | --- | --- |
| **No** | **In-text reference** | **Year Study was Conducted** | **Study Type** | **Geographical Region** | **Shift Type** | **Total Sample Size*** | **Mean Age (SD)** | **Proportion of Female (%)** | **Sleep Duration Method** | **Sleep Duration Mean (SD)** | **PSQI Translation** | **PSQI Mean (SD)** |
| 1 | (Tosun et al., 2022) | 2021 | observational | EUR | night | 142 | 27.25 (6.25) | 78.2 |  |  | PSQI - Turkish | 7.53(3.94) |
| 2 | (Al-Hammouri & Rababah, 2024) | 2022 | intervention | EMR | night | 123 | 30.9 (4.5) | 74.8 | SR | 6.89(1.6) | PSQI | 10.51(3.64) |
| 3 | (Yu et al., 2022) | 2019 | observational | WPR | night | 93 | 33.9 (9.6) | 73.1 | ACT | 8.51(2.35) |  |  |
| 4 | (Alward & Monk, 1990) | 1990 | observational | AMR | night | 60 | 33 (NR) | 100 | SR | 5.338(1.55) | PSQI | 9.45(4.745) |
| 5 | (Güneysu et al., 2024) | 2022 | observational | EUR | non night | 114 | 30.76 (6.64) | 76.3 |  |  | PSQI - Turkish | 12.32(3.3) |
| 6 | (Bharadwaj, 2023) | 2021 | observational | SEAR | NR | 90 | 29.5(NR) | 62.22 |  |  | PSQI | 4.47(1.96) |
| 7 | (Kishore & Shekhar, 2023) | 2021 | observational | SEAR | night | 100 | 24.34 (1.6) | 45 |  |  | PSQI | 5.18(2.32) |
| 8 | (Hittle et al., 2023) | 2020 | observational | AMR | non night | 63 | 44.3 (10.1) | 100 | SR | 6.2(1.3) | PSQI | 10(3.1) |
| 9 | (Chatha et al., 2022) | 2020 | observational | EMR | NR | 60 | 29.21 (6.053) | 79 |  |  | PSQI | 8.25(3.7) |
| 10 | (T. Feng et al., 2021) | 2021 | observational | AMR | non night | 113 | 37.8 (8.6) | 72.6 | ACT | 6.61(0.159) | PSQI | 7.7(2.25) |
| 11 | (Seabra et al., 2019) | 2017 | observational | EUR | night | 1264 | 38.22 (9.57) | 83.2 | SR | 6.43(0.98) |  |  |
| 12 | (Saadat et al., 2018) | 2016 | observational | EMR | non night | 47 | 30.05 (4.75) | 100 | SR | 7.65(4.6) |  |  |
| 13 | (Fang & Li, 2015) | 2013 | intervention | WPR | night | 105 | 35.59(10.445) | 100 |  |  | PSQI - Chinese | 10.11(2.12) |
| 14 | (Cyr et al., 2023) | 2021 | intervention | AMR | night | 57 | 31.15(7.60) | 84.5 | SR | 5.04(2.49) |  |  |
| 15 | (Scott et al., 2010) | 2010 | intervention | AMR | night | 47 | 37.74(11.7) | 96.8 | SR | 7.8(0.95) | PSQI | 8.35(2.70) |
| 16 | (Perez-Fuentes et al., 2019) | 2019 | observational | EUR | NR | 1094 | 32.4(7.27) | 85.1 |  |  | PSQI | 6.44(2.90) |
| 17 | (Tamrakar et al., 2023) | 2020 | observational | SEAR | night | 96 | 27.41(3.37) | 98.9 | SR | 6.76(1.21) |  |  |
| 18 | (X. Liu et al., 2022) | 2020 | observational | WPR | NR | 118 | 32(5.14) | 89 |  |  | PSQI | 7.38(4.04) |
| 19 | (Zarzour et al., 2022) | 2020 | observational | EMR | NR | 503 | 35.67(NR) | 79.1 |  |  | PSQI | 5.93(3.67) |
| 20 | (Sayik et al., 2022) | 2020 | observational | EUR | night | 118 | 32.62(7.36) | 85.5 |  |  | PSQI | 14.29(3.1) |
| 21 | (Norful et al., 2023) | 2020 | observational | AMR | NR | 423 | 42(12.34) | 87.5 |  |  | PSQI | 9.27(4.14) |
| 22 | (van de Langenberg et al., 2023) | 2012 | observational | WPR | night | 59,947 | 46.7(11.3) | 100 | SR | 7.15(1) |  |  |
| 23 | (Togo et al., 2017) | 2010 | observational | WPR | night | 3032 | 40.85(10.05) | 96.35 | SR | 6.795(1.27) |  |  |
| 24 | (H.-l. Feng et al., 2021) | 2019 | observational | WPR | night | 3206 | 33.11(6.35) | 95.98 |  |  | PSQI - Chinese | 4.64(3.67) |
| 25 | (Li et al., 2022) | 2020 | observational | WPR | NR | 357 | 31.15(5.57) | 86 |  |  | PSQI - Chinese | 9.29(4.73) |
| 26 | (Kagamiyama et al., 2019) | 2017 | observational | WPR | night | 61 | 32.04(NR) | 96.7 | SR | 8.3(1.4) |  |  |
| 27 | (Park et al., 2018) | 2014 | observational | SEAR | night | 188 | 30.84(7.08) | 96.8 |  |  | PSQI | 7.18(2.98) |
| 28 | (Ichiba et al., 2018) | 2015 | observational | WPR | night | 35 | 36.9(10) | 88.57 |  |  | PSQI - Japanese | 5.95(1.6) |
| 29 | (Tada et al., 2014) | 2010 | observational | WPR | night | 2758 | 41.6(10.65) | 100 | SR | 6.725(1.325) |  |  |
| 30 | (Dong et al., 2022) | 2020 | observational | WPR | night | 4815 | 34.2(7.9) | 98.1 |  |  | PSQI - Chinese | 9.2(4.5) |
| 31 | (Yan & Xie, 2022) | 2020 | observational | WPR | night | 1344 | 46.12(10.47) | 100 |  |  | PSQI | 6.02(3.35) |
| 32 | (Wang et al., 2024) | 2023 | observational | WPR | night | 1022 | 29(NR) | 90.9 |  |  | PSQI | 10.51(2.23) |
| 33 | (Lin et al., 2012) | 2005 | observational | WPR | night | 1360 | 29.9(NR) | 100 |  |  | PSQI | 7.85(3.44) |
| 34 | (Qiao et al., 2020) | 2016 | observational | WPR | night | 651 | 29.43(4.93) | 98.16 |  |  | PSQI | 6.45(2.44) |
| 35 | (Tsou et al., 2021) | 2019 | observational | WPR | night | 1758 | 35.2(11.02) | 95.85 | SR | 6.61(1.1) |  |  |
| 36 | (Aydin Sayilan et al., 2021) | 2020 | observational | EUR | NR | 267 | 28.03(5.99) | 75.3 |  |  | PSQI | 10.07(3.08) |
| 37 | (Makowiec-Dąbrowska et al., 2000) | 1998 | observational | EUR | non night | 698 | 40.19(8.24) | 97.7 | SR | 8.1(3.01) |  |  |
| 38 | (Han et al., 2019) | 2014 | longitudinal | SEAR | night | 493 | 24(HR) | 95 | SR | 7.2(1.145) |  |  |
| 39 | (Debbia et al., 2021) | 2018 | observational | EUR | night | 92 | 49.27(9.93) | 80.40 |  |  | PSQI - Spanish | 7.01(3.52) |
| 40 | (Kang et al., 2015) | 2015 | observational | WPR | non night | 35 | 25.9(3.19) | 96.6 | SR | 6.74(1.49) | PSQI | 8.77(2.75) |
| 41 | (Zion & Shochat, 2018) | 2015 | observational | EUR | night | 109 | 39.29(9.06) | 100 | ACT | 7.45(2.21) | PSQI | 5.49(2.87) |
| 42 | (Di Muzio et al., 2021) | 2020 | observational | EUR | night | 144 | 41.3(0.8) | 63.9 |  |  | PSQI - Italian | 6.38(0.355) |
| 43 | (Fang et al., 2019) | 2016 | observational | WPR | night | 172 | 32.22(7.36) | 100 |  |  | PSQI - Chinese | 5.48(1.39) |
| 44 | (Bamonti et al., 2019) | 2019 | observational | AMR | non night | 56 | 33.52(11.35) | 89.3 | SR | 6(1.6) |  |  |
| 45 | (Ruggiero, 2003) | 2003 | observational | AMR | night | 142 | 44.9(8.3) | 100 |  |  | PSQI | 7.06(3.42) |
| 46 | (Minelli et al., 2021) | 2021 | observational | EUR | night | 30 | 39.6(1.57) | 70 |  |  | PSQI | 4.97(0.51) |
| 47 | (Yoo et al., 2023) | 2020 | longitudinal | AMR | non night | 392 | 39.54(11.15) | 91.9 | ACT | 6.7(0.84) |  |  |
| 48 | (Alimoglu & Donmez, 2005) | 2003 | observational | EUR | night | 141 | 29.7(6) | 100 | SR | 6.85(1.35) |  |  |
| 49 | (Molu et al., 2023) | 2022 | observational | EUR | night | 134 | 28.94(8.25) | 72.4 |  |  | PSQI | 8.95(2.25) |
| 50 | (Karahan et al., 2020) | 2018 | observational | EUR | night | 285 | 27.69(7.76) | 91.2 |  |  | PSQI | 7.87(3.82) |
| 51 | (Olson et al., 2020) | 2018 | observational | AMR | night | 33 | 32.73(8.59) | 76 | SR | 5.16(3.06) |  |  |
| 52 | (Chang et al., 2015) | 2013 | observational | WPR | night | 63 | 25.7(2.5) | 92.5 | SR | 5.4(2.33) |  |  |
| 53 | (Beebe et al., 2017) | 2016 | observational | AMR | night | 103 | 38.8(13.5) | 98 |  |  | PSQI | 6.3(4.2) |
| 54 | (Roveda et al., 2021) | 2019 | observational | EUR | night | 37 | 41.7(9.1) | 83.76 | SR | 6.05(0.71) |  |  |
| 55 | (JÄRvelin-Pasanen et al., 2013) | 2011 | observational | EUR | night | 51 | 42(11) | 100 | SR | 7.5(1.4) |  |  |
| 56 | (Chang & Li, 2019) | 2019 | observational | WPR | night | 191 | 26.62(5) | 100 |  |  | PSQI | 8.043(2.32) |
| 57 | (Hansen et al., 2006) | 2007 | observational | EUR | non night | 170 | 42(10) | 100 | SR | 7.365(1.17) |  |  |
| 58 | (Ulusoy et al., 2021) | 2019 | observational | EUR | night | 44 | 24.5(5.3) | 72.7 | SR | 7.25(1.45) |  |  |
| 59 | (Garrido et al., 2021) | 2018 | observational | AMR | night | 36 | 39.4(1) | 100 | SR | 5.76(0.92) |  |  |
| 60 | (Balamadhuwanthi et al., 2023) | 2021 | observational | EMR | NR | 60 | 23.45(0.965) | 83.6 |  |  | PSQI | 7.26(2.1) |
| 61 | (Mousavi et al., 2020) | 2018 | intervention | EMR | NR | 539 | 35.6(8.5) | 86.5 |  |  | PSQI | 6.4(3.9) |
| 62 | (Parajuli et al., 2021) | 2019 | intervention | SEAR | NR | 33 | 40.6(10.26) | 100 |  |  | PSQI | 5.09(3.48) |
| 63 | (Donmezdil & Arac, 2021) | 2020 | observational | EUR | night | 72 | 29(3.95) | 41.7 |  |  | PSQI | 9.3(4.9) |
| 64 | (Seong et al., 2022) | 2021 | observational | SEAR | night | 60 | 27.08(2.81) | 100 |  |  | PSQI - Korean | 7.55(2.4) |
| 65 | (Ha et al., 2022) | 2021 | intervention | SEAR | night | 57 | 27.33(3.37) | 100 |  |  | PSQI - Korean | 8.97(3.15) |
| 66 | (Wang, Lin, Xiong, et al., 2023) | 2021 | intervention | WPR | NR | 401 | 29.3(6.49) | 93.4 |  |  | PSQI - Chinese | 9.44(3.37) |
| 67 | (Watanabe et al., 2022) | 2022 | longitudinal | WPR | night | 49 | 28.8(4.2) | 93.9 | SR | 6.3(0.9) |  |  |
| 68 | (Chen et al., 2006) | 2006 | longitudinal | WPR | NR | 116 | 31(10.8) | 98.3 |  |  | PSQI | 12(3.83) |
| 69 | (Spelten et al., 1995) | 1993 | observational | EUR | night | 572 | 33.26(8.99) | 100 | SR | 7.88(1.27) |  |  |
| 70 | (Shiffer et al., 2018) | 2016 | observational | EUR | night | 100 | 30(5.5) | 100 | SR | 6.525(1.555) |  |  |
| 71 | (Tahvilian et al., 2022) | 2020 | observational | EMR | night | 302 | 36.9(7.6) | 50.6 |  |  | PSQI | 9.97(2.56) |
| 72 | (Kikuchi et al., 2018) | 2016 | observational | WPR | night | 31 | 49.8(6.3) | 100 | SR | 5.65(0.87) |  |  |
| 73 | (Tsai et al., 2019) | 2018 | observational | WPR | non night | 61 | 26.72(4.56) | 100 | ACT | 6.03(1.13) |  |  |
| 74 | (Guofang et al., 2024) | 2022 | intervention | WPR | night | 1037 | 34(10.4) | 90.3 |  |  | PSQI | 7.19(3.65) |
| 75 | (Kurebayashi, 2020) | 2018 | observational | WPR | night | 404 | 40.2(11.6) | 57.7 |  |  | PSQI - Japanese | 5.9(3.1) |
| 76 | (Labyak et al., 2002) | 2000 | observational | AMR | night | 68 | 29.95(0.85) | 100 | SR | 6.84(0.287) |  |  |
| 77 | (Chappel et al., 2020) | 2018 | observational | WPR | night | 47 | 33(17) | 90 |  |  | PSQI | 8(3.3) |
| 78 | (Helvaci et al., 2020) | 2018 | observational | EUR | non night | 298 | 29.9(6.9) | 92.6 |  |  | PSQI - Turkish | 7.2(3.5) |
| 79 | (Cox et al., 2021) | 2019 | observational | AMR | non night | 105 | 55.44(NR) | 97 |  |  | PSQI | 8.14(3.81) |
| 80 | (Deng et al., 2021) | 2018 | observational | WPR | non night | 223 | 36.07(11.31) | 96.6 |  |  | PSQI - Chinese | 8.19(3.24) |
| 81 | (Hui-Ren et al., 2023) | 2021 | observational | WPR | night | 1060 | 31.85(7.15) | 92.08 |  |  | PSQI | 7.37(3.54) |
| 82 | (Mokros et al., 2018) | 2016 | observational | EUR | night | 31 | 51.97(8.57) | 100 |  |  | PSQI | 6.77(2.86) |
| 83 | (Weaver et al., 2024) | 2022 | observational | AMR | night | 1657 | 38(12.5) | 85.5 |  |  | PSQI | 8.36(3.69) |
| 84 | (Hsu, Lee, et al., 2021) | 2014 | observational | WPR | night | 393 | 31.48(7.41) | 100 |  |  | PSQI | 10.2(2.85) |
| 85 | (Li et al., 2023) | 2023 | observational | WPR | night | 1426 | 30.2(7.6) | 98.2 |  |  | PSQI - Chinese | 7.9(3.67) |
| 86 | (Y. Liu et al., 2022) | 2021 | observational | WPR | night | 1004 | 32.19(5.81) | 95.4 |  |  | PSQI | 10.545(3.39) |
| 87 | (Colak & Esin, 2024) | 2022 | observational | EUR | night | 83 | 25.71(4.47) | 69.9 |  |  | PSQI | 8.9(3.16) |
| 88 | (Hwang et al., 2022) | 2020 | observational | SEAR | night | 125 | 28(3.1) | 100 |  |  | PSQI - Korean | 8.5(3.6) |
| 89 | (Suleiman, Hijazi, et al., 2020) | 2018 | observational | EMR | night | 186 | 30.53(6.46) | 51 |  |  | PSQI | 8.76(3.18) |
| 90 | (Chen et al., 2022) | 2020 | observational | WPR | NR | 327 | 42.27(NR) | 99.4 |  |  | PSQI - Chinese | 6.74(2.895) |
| 91 | (Fang et al., 2008) | 2006 | observational | WPR | night | 581 | 29.49(6.73) | 100 |  |  | PSQI | 7.84(3.86) |
| 92 | (Kim-Godwin et al., 2021) | 2020 | observational | AMR | non night | 215 | 36.07(10.24) | 100 |  |  | PSQI | 9.85(2.72) |
| 93 | (Kunert et al., 2007) | 2005 | observational | AMR | non night | 190 | 36.3(7.55) | 85 |  |  | PSQI | 6.75(2.65) |
| 94 | (Hausswirth et al., 2022) | 2020 | intervention | EUR | NR | 45 | 44.63(10.76) | 77.8 | ACT | 6.04(0.613) |  |  |
| 95 | (Zhou et al., 2017) | 2010 | observational | WPR | night | 402 | 31.4(8.4) | 98 |  |  | PSQI | 5.105(2.84) |
| 96 | (Huang et al., 2017) | 2013 | observational | AMR | night | 209 | 60.8(3.95) | 100 |  |  | PSQI | 6.5(3.3) |
| 97 | (Ruggiero, 2005) | 2003 | observational | AMR | night | 247 | 43.2(8.6) | 100 |  |  | PSQI | 7.1(3.5) |
| 98 | (Y. Zhang et al., 2023) | 2021 | observational | WPR | night | 52 | 30.81(4.927) | 96.2 |  |  | PSQI | 8.69(4.346) |
| 99 | (Faraut et al., 2022) | 2018 | observational | EUR | night | 191 | 39.27(10.63) | 95.00 | SR | 7.295(1.32) |  |  |
| 100 | (Bayoumi et al., 2020) | 2019 | observational | EMR | night | 40 | 26.2(4.05) | 70.00 |  |  | PSQI | 17.27(1.66) |
| 101 | (Chiang et al., 2022) | 2019 | observational | WPR | night | 329 | 32.8(8.60) | 95.40 | SR | 7.7(1.7) |  |  |
| 102 | (Yamasaki et al., 1998) | 1996 | observational | AMR | night | 128 | 40.7(NR) | 100 | SR | 6.2(1.525) |  |  |
| 103 | (Sirinara et al., 2019) | 2016 | observational | SEAR | non night | 2765 | 41.1(13.5) | 94.6 |  |  | PSQI | 5.4(2.6) |
| 104 | (Kim et al., 2013) | 2011 | observational | SEAR | night | 207 | 32.3(7.80) | 79 | SR | 6.255(1.375) | PSQI - Korean | 6.61(3.11) |
| 105 | (X. Zhang et al., 2023) | 2021 | observational | WPR | night | 152 | 31.81(6.09) | 100 | ACT | 6.83 (1.81) |  |  |
| 106 | (Ding et al., 2023) | 2022 | observational | WPR | NR | 1774 | 32(7.99) | 93.9 |  |  | PSQI - Chinese | 6.33(2.44) |
| 107 | (Saleh et al., 2014) | 2012 | observational | EMR | night | 52 | 23.48(3.20) | 98.10 | SR | 6.59(1.82) |  |  |
| 108 | (Nishimura et al., 2023) | 2020 | observational | WPR | night | 30 | 28.2(5.90) | 100 | SR | 7.4(2.5) |  |  |
| 109 | (Majumdar et al., 2023) | 2021 | observational | SEAR | night | 80 | 28.42(10.65) | 100 | SR | 6.3(1.0525) | PSQI | 5.8(2.27) |
| 110 | (Duan et al., 2017) | 2014 | observational | SEAR | night | 60 | 30.18(7.15) | 92 |  |  | PSQI | 6.26(3.41) |
| 111 | (Vijaykumar et al., 2018) | 2016 | observational | WPR | NR | 150 | 32.47(4.9) | 100 |  |  | PSQI | 5.59(1.29) |
| 112 | (Roman et al., 2023) | 2019 | observational | EUR | night | 380 | 24.8(6.9) | 92.9 |  |  | PSQI | 9.6(4.4) |
| 113 | (Kim & Lee, 2022) | 2020 | observational | SEAR | night | 88 | 23.25(1.26) | 71 |  |  | PSQI | 7.47(3.34) |
| 114 | (Chang & Yang, 2020) | 2018 | observational | WPR | night | 145 | 27(NR) | 100 |  |  | PSQI | 7.05(3.07) |
| 115 | (Zamanian et al., 2016) | 2014 | observational | EMR | night | 1456 | 30.81(7.8) | 89.04 | SR | 6.66(1.3) |  |  |
| 116 | (Cho et al., 2023) | 2021 | observational | SEAR | night | 198 | 38.3(10.9) | 96 |  |  | PSQI | 7.35(3.1) |
| 117 | (Khatony et al., 2020) | 2020 | observational | EMR | night | 540 | 32.02(5.63) | 66.30 |  |  | PSQI | 6.81(3.18) |
| 118 | (Polydoropoulou et al., 2023) | 2021 | observational | EUR | night | 1710 | 42.3(9.1) | 77.2 |  |  | PSQI - Greek | 8.64(3.1) |
| 119 | (Yuan & Fang, 2024) | 2020 | observational | WPR | night | 9719 | 28.76(NR) | 97.84 |  |  | PSQI | 13.345(1.95) |
| 120 | (Zion & Shochat, 2019) | 2014 | observational | EUR | night | 119 | 39(9.1) | 100 |  |  | PSQI - Hebrew | 5.6(2.8) |
| 121 | (Grundy et al., 2009) | 2006 | observational | AMR | night | 61 | 42.62(7.57) | 100 | SR | 6.47(2.29) |  |  |
| 122 | (R. T. Lin et al., 2021) | 2017 | observational | WPR | NR | 973 | 31(7.5) | 95.5 | SR | 6.8(1.1) |  |  |
| 123 | (S. Wu et al., 2023) | 2019 | observational | WPR | night | 379 | 29.64(5.49) | 95.78 |  |  | PSQI - Chinese | 6.75(3.12) |
| 124 | (Zarei & Fooladvand, 2022) | 2021 | observational | EMR | NR | 250 | 37.62(5.13) | 74 |  |  | PSQI | 8.93(2.09) |
| 125 | (Wang, Lin, Liang, et al., 2023) | 2022 | observational | WPR | NR | 496 | 28.8(NR) | 93.8 |  |  | PSQI | 11.45(3.33) |
| 126 | (Holanda et al., 2018) | 2016 | observational | AMR | night | 60 | 39.8(10.5) | 100 | SR | 6.9(0.5) |  |  |
| 127 | (Molzof et al., 2019) | 2017 | observational | AMR | NR | 30 | 31.2(NR) | 100 | ACT | 6.475(0.522) |  |  |
| 128 | (Hajaghazadeh et al., 2019) | 2018 | observational | EMR | night | 120 | 33.09(6.89) | 97 |  |  | PSQI - Persian | 6.88(2.18) |
| 129 | (Apellido, 2018) | 2017 | observational | AMR | night | 67 | 45.126 | 100 |  |  | PSQI | 4.42(2.22) |
| 130 | (Peplonska et al., 2012) | 2010 | observational | EUR | non night | 724 | 49.25(5.25) | 100 |  |  | PSQI | 6.65(3.3) |
| 131 | (Matre et al., 2017) | 2015 | intervention | EUR | night | 53 | 31.6(9) | 77 | SR | 7(1.4) |  |  |
| 132 | (Haseli et al., 2023) | 2020 | observational | EMR | NR | 340 | 32.12(4.01) | 81.2 | SR | 7.12(4.63) | PSQI | 8.4(4.46) |
| 133 | (Di Muzio et al., 2020) | 2017 | observational | EUR | night | 86 | 40(1.535) | 63.95 |  |  | PSQI | 6.44(0.49) |
| 134 | (Botti et al., 2022) | 2020 | observational | EUR | night | 308 | 42.2(10.5) | 82.5 |  |  | PSQI | 7.8(4.5) |
| 135 | (Petrosino et al., 2024) | 2023 | observational | EUR | night | 160 | 38.06(10.73) | 54.4 |  |  | PSQI | 7.59(3.5) |
| 136 | (Huang et al., 2022) | 2020 | observational | WPR | NR | 966 | 31.92(5.89) | 91.3 |  |  | PSQI | 6.58(3.7) |
| 137 | (Gomez-Garcia et al., 2016) | 2014 | observational | EUR | night | 635 | 41.1(10.03) | 87.2 |  |  | PSQI | 6.8(3.39) |
| 138 | (Stimpfel et al., 2020) | 2016 | observational | AMR | night | 1558 | 31.7(8.7) | 88.4 | SR | 7.6(1.55) |  |  |
| 139 | (Kumar et al., 2022) | 2020 | observational | SEAR | night | 60 | 24.32(1.6) | 36.67 |  |  | PSQI | 5.13(2.33) |
| 140 | (Assadi et al., 2019) | 2017 | observational | EMR | night | 205 | 31(NR) | 79.51 | SR | 7.352(1.595) |  |  |
| 141 | (Haldar & Sahu, 2015) | 2014 | observational | SEAR | night | 122 | 30.56(0.37) | 100 | SR | 5.85(0.16) |  |  |
| 142 | (Zhang et al., 2021) | 2020 | observational | WPR | NR | 323 | 32.11(6.75) | 91.6 |  |  | PSQI - Chinese | 8.73(3.49) |
| 143 | (Katsifaraki et al., 2020) | 2020 | observational | EUR | night | 679 | 41(11.1) | 90.6 | SR | 5.59(0.89) |  |  |
| 144 | (Rahman et al., 2022) | 2020 | observational | WPR | night | 130 | 36.2(7.8) | 77.3 |  |  | PSQI | 9.4(2.2) |
| 145 | (Hajo et al., 2020) | 2018 | observational | AMR | night | 342 | 43(12) | 94 | ACT | 8.65(1.08) |  |  |
| 146 | (Almhdawi et al., 2020) | 2018 | observational | EMR | non night | 597 | 32.1(5.7) | 52.8 |  |  | PSQI - Greek | 7.75(3.7) |
| 147 | (Winyuchakrit & Taneepanichskul, 2017) | 2017 | observational | SEAR | night | 209 | 31.38(5.35) | 100 | SR | 6.23(0.99) |  |  |
| 148 | (Kayaroganam et al., 2022) | 2020 | observational | SEAR | night | 1217 | 36.6(8.6) | 77.5 |  |  | PSQI | 4.49(2.98) |
| 149 | (Bazrafshan et al., 2018) | 2017 | observational | EMR | night | 100 | 30.4(7.5) | 49 |  |  | PSQI | 6.52(4.23) |
| 150 | (Su et al., 2007) | 2005 | longitudinal | WPR | NR | 102 | 29.85(5.66) | 100 |  |  | PSQI | 5.475(2.45) |
| 151 | (J. Wang et al., 2022) | 2020 | observational | WPR | night | 812 | 32.69(8.07) | 74.4 |  |  | PSQI | 5.77(3.08) |
| 152 | (Chueh et al., 2021) | 2014 | observational | WPR | non night | 119 | 31.6(7.6) | 100 | SR | 6.4(1.5) | PSQI | 7.2(3.1) |
| 153 | (Samaha et al., 2007) | 2006 | observational | WPR | night | 111 | 37.5(12.5) | 88.3 |  |  | PSQI | 6.7(3.2) |
| 154 | (Koh et al., 2014) | 2013 | observational | SEAR | night | 301 | 28.12(4.88) | 96.1 | SR | 8.03(1.93) | PSQI | 9.83(2.78) |
| 155 | (Farmer et al., 2022) | 2018 | observational | AMR | night | 377 | 39.46(11.18) | 91.78 | ACT | 6.75(0.95) |  |  |
| 156 | (Quera-Salva et al., 1997) | 1995 | observational | EUR | NR | 40 | 35.5(7) | 80 | ACT | 5.9(1.125) |  |  |
| 157 | (Totterdell et al., 1995) | 1995 | observational | EUR | night | 61 | 33.55(8.25) | 100 | SR | 7.34(1.17) |  |  |
| 158 | (Hsieh et al., 2021) | 2018 | observational | WPR | night | 248 | 32.98(8.25) | 100 |  |  | PSQI - Chinese | 7.04(3.19) |
| 159 | (Pelin et al., 2023) | 2021 | observational | EUR | night | 221 | 29.83(7.79) | 81 |  |  | PSQI | 6.81(3.3) |
| 160 | (Miguez-Torres et al., 2021) | 2020 | observational | EUR | NR | 62 | 33.177(NR) | 77 |  |  | PSQI - Spanish | 7.44(3.76) |
| 161 | (Chang & Chang, 2019) | 2017 | observational | WPR | non night | 178 | 26.35(4.26) | 100 |  |  | PSQI | 6.76(1.98) |
| 162 | (Shi et al., 2020) | 2019 | observational | WPR | night | 521 | 25.86(NR) | 96.6 |  |  | PSQI | 7.44(3.285) |
| 163 | (Sajedi et al., 2022) | 2021 | observational | EMR | night | 200 | 32.784(NR) | 83.5 |  |  | PSQI | 8.81(3.62) |
| 164 | (da Silva et al., 2017) | 2015 | observational | AMR | non night | 104 | 34.5(NR) | 90.38 | SR | 6.47(1.03) |  |  |
| 165 | (Mohamed et al., 2022) | 2020 | observational | EMR | night | 139 | 28.46(6.28) | 73.4 |  |  | PSQI | 8.14(3.1) |
| 166 | (Liu et al., 2020) | 2019 | observational | WPR | night | 2372 | 31.18(7.2) | 97.4 |  |  | PSQI | 7.56(3.62) |
| 167 | (L. Wang et al., 2022) | 2020 | observational | WPR | night | 414 | 32.37(NR) | 73.7 |  |  | PSQI | 7.07(3.7) |
| 168 | (Smart & Wilson, 2013) | 2008 | observational | AMR | non night | 131 | 44.4(11.3) | 84 | SR | 7.5(0.86) |  |  |
| 169 | (L. C. Lin et al., 2021) | 2020 | observational | AMR | night | 120 | 42.69(11.71) | 85 |  |  | PSQI | 7.53(3.93) |
| 170 | (Y. Wu et al., 2023) | 2020 | observational | WPR | night | 1286 | 30(8.3) | 96.6 |  |  | PSQI | 6.52(3.69) |
| 171 | (Shin & Kim, 2021) | 2019 | intervention | SEAR | NR | 62 | 26.77(2.5) | 82.8 | ACT | 5.71(1.26) |  |  |
| 172 | (Bracci et al., 2014) | 2012 | observational | EUR | night | 116 | 39.75(5.25) | 100 |  |  | PSQI | 5.5(2.9) |
| 173 | (Vivarelli et al., 2023) | 2021 | observational | EUR | night | 102 | 46.17 | 53.9 |  |  | PSQI | 4.15(2.84) |
| 174 | (Sveinsdottir, 2006) | 2002 | observational | EUR | non night | 394 | 43.77(8.87) | 100 | SR | 7.37(0.93) |  |  |
| 175 | (Morimoto et al., 2016) | 2014 | intervention | WPR | night | 84 | 34.4(12.9) | 94.1 |  |  | PSQI - Japanese | 6.2(2.9) |
| 176 | (Bjorvatn et al., 2012) | 2006 | observational | EUR | night | 150 | 39.4(9.1) | 90.7 |  |  | PSQI | 7.5(3) |
| 177 | (Lee, 1992) | 1990 | observational | AMR | non night | 759 | 35.23(9.175) | 100 | SR | 8.55(1.51) |  |  |
| 178 | (Shehata et al., 2021) | 1990 | observational | EMR | night | 73 | 37.32(10.21) | 72.6 |  |  | PSQI | 8.085(3.49) |
| 179 | (Alaradi et al., 2023) | 2021 | observational | EMR | night | 200 | 38.2(8.2) | 72.5 |  |  | PSQI | 13.2(5.2) |
| 180 | (Kim & Jang, 2021) | 2021 | observational | SEAR | night | 194 | 31.59(5.93) | 100 | SR | 7.09(1.02) |  |  |
| 181 | (Taylor et al., 2022) | 2020 | observational | AMR | night | 454 | 38.9(11.1) | 91 | SR | 6.696(0.8497) |  |  |
| 182 | (Nascimento et al., 2019) | 2017 | observational | AMR | night | 231 | 39.6(NR) | 82.7 | SR | 6.3(1.5) |  |  |
| 183 | (Huth et al., 2013) | 2011 | observational | AMR | night | 378 | 38.71(11.42) | 96 |  |  | PSQI | 11.45(2.07) |
| 184 | (Chin et al., 2021) | 2019 | observational | WPR | night | 1602 | 31(7.2) | 100 | SR | 6.7(1) |  |  |
| 185 | (Chaiard et al., 2018) | 2016 | observational | SEAR | night | 220 | 38.45(NR) | 92.3 | SR | 6.2(1.2) |  |  |
| 186 | (Thun et al., 2021) | 2019 | observational | EUR | night | 1272 | 40.5(8.1) | 90 | SR | 6.78(0.817) |  |  |
| 187 | (Chin et al., 2015) | 2013 | observational | WPR | night | 1384 | 31.9(8) | 100 | SR | 6.6(0.9) |  |  |
| 188 | (Juan-Garcia et al., 2021) | 2020 | observational | EUR | night | 70 | 35(2.31) | 81.4 |  |  | PSQI - Spanish | 7.4(2.09) |
| 189 | (Garcia-Tudela et al., 2023) | 2020 | observational | EUR | night | 154 | 40.4(11.1) | 84 |  |  | PSQI | 8.06(4.23) |
| 190 | (Johnson et al., 2014) | 2012 | observational | AMR | night | 255 | 39(9.8) | 82.33 | SR | 5.34(2.07) |  |  |
| 191 | (Vedaa et al., 2017) | 2015 | observational | EUR | night | 67 | 47.7(9.6) | 94 | SR | 6.375(1.01) |  |  |
| 192 | (Baek et al., 2020) | 2018 | observational | SEAR | night | 94 | 28.09(4.28) | 100 | SR | 7.005(0.77) |  |  |
| 193 | (Hsieh et al., 2011) | 2009 | observational | WPR | night | 661 | 31.86(8.09) | 95.5 |  |  | PSQI | 7.01(3.72) |
| 194 | (Han et al., 2016) | 2015 | observational | WPR | night | 2033 | 27(7) | 94.1 |  |  | PSQI | 7.26(3.56) |
| 195 | (Al Maqbali, 2021) | 2020 | observational | EMR | NR | 987 | 36.16(NR) | 90.7 | SR | 7.04(1.59) |  |  |
| 196 | (Park et al., 2020) | 2018 | observational | SEAR | NR | 39 | 29.7(3.6) | 100 |  |  | PSQI | 5.75(1.8) |
| 197 | (Barak et al., 1995) | 1993 | observational | EUR | night | 175 | 36.9(9.8) | 100 | SR | 70.5(1.9) |  |  |
| 198 | (Boned-Galán et al., 2022) | 2020 | observational | EUR | non night | 87 | 33.68(NR) | 86.2 |  |  | PSQI | 8.45(4.29) |
| 199 | (Katsifaraki et al., 2019) | 2017 | observational | EUR | non night | 679 | 40.7(11.3) | 95.6 | SR | 6.71(2) |  |  |
| 200 | (James et al., 2020) | 2018 | intervention | AMR | night | 165 | 36.26(9.68) | 92.2 | SR | 6.66(1.03) |  |  |
| 201 | (Lee et al., 2021) | 2019 | observational | AMR | night | 61 | 35.39(11.73) | 91.8 | SR | 6.95(0.96) |  |  |
| 202 | (Khastar et al., 2020) | 2018 | intervention | EMR | night | 113 | 33.785(6.575) | 100 | 33.785 |  | PSQI | 7.61(1.715) |
| 203 | (Ruggiero & Avi-Itzhak, 2016) | 2011 | observational | AMR | non night | 35 | 40.69(10.86) | 85.7 | ACT | 7.108(2.14) |  |  |
| 204 | (Dong et al., 2017) | 2015 | observational | WPR | night | 4951 | 28(6) | 94 |  |  | PSQI - Chinese | 7.32(3.24) |
| 205 | (Chien et al., 2013) | 2009 | observational | WPR | non night | 156 | 34.6(8.1) | 100 |  |  | PSQI - Chinese | 7.34(2.94) |
| 206 | (Akbarzadeh et al., 2014) | 2014 | observational | EMR | night | 135 | 31.64(NR) | 91.85 |  |  | PSQI | 7.17(2.08) |
| 207 | (Alharbi et al., 2021) | 2020 | observational | EMR | night | 238 | 34.058(NR) | 84 |  |  | PSQI | 6.98(3.4) |
| 208 | (Christina & Konjengbam, 2019) | 2018 | observational | SEAR | night | 457 | 39.12(8.591) | 98.9 |  |  | PSQI | 4.98(3.12) |
| 209 | (Aliyu et al., 2017) | 2017 | observational | AFR | NR | 100 | 31.4(8.6) | 77 |  |  | PSQI | 5.7(2.70) |
| 210 | (Segon et al., 2022) | 2021 | observational | AFR | night | 510 | 29.26(3.95) | 43.3 |  |  | PSQI | 7.21(1.42) |
| 211 | (Dagcan & Arslan, 2021) | 2019 | observational | EUR | night | 583 | 38.19(NR) | 85.1 |  |  | PSQI | 7.45(3.35) |
| 212 | (Alameri et al., 2024) | 2022 | observational | EMR | night | 173 | 35.22(NR) | 91.9 |  |  | PSQI | 8.16(3.46) |
| 213 | (Karagozoglu & Bingol, 2008) | 2005 | observational | EUR | night | 418 | 27.4(3.99) | 85.4 |  |  | PSQI | 7.28(3.56) |
| 214 | (Tu et al., 2020) | 2020 | observational | WPR | NR | 100 | 34.44(5.85) | 100 |  |  | PSQI - Chinese | 8.48(3.63) |
| 215 | (Shao et al., 2010) | 2008 | observational | WPR | night | 435 | 29.5(5.43) | 100 |  |  | PSQI | 5.33(2.8) |
| 216 | (Hesselbacher et al., 2015) | 2012 | observational | AMR | non night | 67 | 36.55(10.85) | 83.58 |  |  | PSQI | 5.82(2.63) |
| 217 | (Niu et al., 2017) | 2016 | intervention | WPR | non night | 62 | 27.76(4.31 | 100 | ACT | 6.095(1.1675) |  |  |
| 218 | (Sampaio et al., 2023) | 2020 | observational | EUR | NR | 629 | 41.3(9.56) | 83.79 | SR | 6.33(1.74) |  |  |
| 219 | (Sungur et al., 2022) | 2020 | observational | EUR | night | 234 | 30.43(NR) | 81.2 |  |  | PSQI - Turkish | 10.76(3.23) |
| 220 | (Dong et al., 2020) | 2019 | observational | WPR | night | 4730 | 35.3(6.8) | 88.48 |  |  | PSQI - Chinese | 9.57(5.85) |
| 221 | (Xi et al., 2022) | 2021 | observational | EUR | NR | 442 | 40.46(NR) | 96.61 |  |  | PSQI | 7.07(2.14) |
| 222 | (Topal Kilincarslan et al., 2024) | 2019 | observational | EUR | night | 133 | 34.5(NR) | 85.5 |  |  | PSQI | 8.37(4.47) |
| 223 | (Silva et al., 2022) | 2019 | observational | AMR | night | 42 | 40.2(8.9) | 73.8 |  |  | PSQI - Brazilian | 9.6(3.73) |
| 224 | (Lyu et al., 2022) | 2021 | observational | WPR | night | 950 | 31.93(8.152) | 84.5 |  |  | PSQI | 7(3.59) |
| 225 | (Min et al., 2021) | 2020 | observational | SEAR | night | 82 | 26.79(3.05) | 97.56 | ACT | 6.572(0.897) |  |  |
| 226 | (Arimura et al., 2010) | 2006 | observational | WPR | night | 454 | 36.4(10.8) | 92.51 |  |  | PSQI | 6.8(2.9) |
| 227 | (Geiger-Brown et al., 2012) | 2011 | observational | AMR | night | 80 | 37.2(10.4) | 100 | ACT | 7.5(1.5) |  |  |
| 228 | (Dorrian et al., 2011) | 2009 | observational | WPR | non night | 41 | 36.3(11.3) | 82.93 | SR | 8.3(2.55) |  |  |
| 229 | (Mulhall et al., 2019) | 2017 | observational | WPR | night | 33 | 34.06(11.37) | 79.79 | SR | 6.81(0.18) |  |  |
| 230 | (Kato et al., 2012) | 2007 | observational | WPR | non night | 51 | 49.2(8.9) | 100 | ACT | 6.01(0.8) |  |  |
| 231 | (Lee & Jang, 2022) | 2018 | observational | SEAR | night | 190 | 26.41(2.6) | 100 | SR | 6.83(1.45) | PSQI - Korean | 12.79(8.145) |
| 232 | (Rajcani et al., 2021) | 2020 | observational | EUR | NR | 67 | 39.22(9.93) | 100 |  |  | PSQI | 8.91(4.16) |
| 233 | (Yu et al., 2024) | 2022 | observational | WPR | night | 1384 | 29.39(NR) | 97 |  |  | PSQI - Chinese | 5.97(3.42) |
| 234 | (Bilgic et al., 2021) | 2020 | observational | EUR | night | 338 | 32.24(8.41) | 84.9 |  |  | PSQI - Turkish | 7.36(3.35) |
| 235 | (Khade et al., 2018) | 2016 | observational | SEAR | night | 190 | 25.65(NR) | 77.8 |  |  | PSQI | 4.9(1.75) |
| 236 | (Veal et al., 2021) | 2019 | observational | AMR | night | 60 | 35.4(11.8) | 93.3 | ACT | 7.41(1.82) |  |  |
| 237 | (Verhaegen et al., 1987) | 1985 | observational | EUR | night | 167 | 34.52(8.25) | 85 | SR | 7.31(1.29) |  |  |
| 238 | (Korkmaz et al., 2020) | 2019 | observational | EUR | NR | 70 | 30.9(5.9) | 57 |  |  | PSQI | 6.1(4.07) |
| 239 | (Ghorbani et al., 2023) | 2021 | observational | EMR | night | 173 | 33.12(6.74) | 85 |  |  | PSQI | 5.71(2.5) |
| 240 | (Fard et al., 2020) | 2018 | observational | EMR | night | 150 | 29.68(5.69) | 62.7 |  |  | PSQI | 7.16(3.06) |
| 241 | (Gu et al., 2019) | 2017 | observational | WPR | night | 2889 | 31.2(6.72) | 96.7 |  |  | PSQI | 8.67(3.723) |
| 242 | (Stavas et al., 2024) | 2015 | observational | EUR | night | 684 | 40.9(11.1) | 90 | SR | 6.7(1) |  |  |
| 243 | (Cho et al., 2019) | 2017 | observational | SEAR | night | 118 | 33.5(4.8) | 100 |  |  | PSQI | 7.6(1.43) |
| 244 | (Kwak et al., 2024) | 2022 | observational | SEAR | night | 195 | 32.51(7.67) | 89.2 |  |  | PSQI - Korean | 9.51(3.44) |
| 245 | (Ramadan & Al-Saleh, 2014) | 2012 | observational | EMR | night | 138 | 33.9(11.47) | 56.5 | SR | 5.335(1.8) |  |  |
| 246 | (Hayajneh et al., 2024) | 2022 | intervention | EMR | night | 100 | 29.76(3.83) | 69 |  |  | PSQI | 8.5(3.97) |
| 247 | (Celik & Yarali, 2023) | 2021 | intervention | EUR | night | 90 | 28.83(6.43) | 86.6 |  |  | PSQI - Turkish | 17.03(2.77) |
| 248 | (Dai et al., 2019) | 2017 | observational | WPR | night | 865 | 30.41(8.06) | 98 |  |  | PSQI | 8.33(3.54) |
| 249 | (Suleiman, Al-Khateeb, et al., 2020) | 2019 | observational | EMR | night | 179 | 30.44(8.16) | 46.9 |  |  | PSQI - Arabic | 7.87(3.86) |
| 250 | (Zadeh et al., 2018) | 2015 | intervention | EMR | night | 36 | 34.16(11.34) | 61.1 |  |  | PSQI | 11.97(1.997) |
| 251 | (Abasi et al., 2022) | 2020 | intervention | EMR | night | 80 | 28.615(3.705) | 61.25 |  |  | PSQI | 7.23(2.77) |
| 252 | (Chang et al., 2017) | 2015 | intervention | WPR | night | 50 | 29.455(5.325) | 100 |  |  | PSQI - Brazilian | 8.49(2.38) |
| 253 | (Nourian et al., 2021) | 2020 | intervention | EMR | NR | 41 | 35.6(8.21) | 59.6 |  |  | PSQI | 9.65(2.39) |
| 254 | (Scott et al., 2006) | 2004 | observational | AMR | non night | 393 | 44.8(8.8) | 92 | SR | 7.42(2.09) |  |  |
| 255 | (Lin et al., 2014) | 2010 | observational | WPR | night | 266 | 30.77(7.38) | 100 |  |  | PSQI - Chinese | 8.82(3.62) |
| 256 | (McDowall et al., 2017) | 2016 | observational | EUR | night | 738 | 41.46(NR) | 90.23 |  |  | PSQI | 7.51(3.31) |
| 257 | (Panwar et al., 2024) | 2022 | observational | SEAR | NR | 38 | 29.2(6) | 100 |  |  | PSQI | 5(3.55) |
| 258 | (Wang et al., 2020) | 2018 | observational | WPR | night | 263 | 30.19(5.71) | 85.2 |  |  | PSQI | 7.53(2.88) |
| 259 | (Setyowati et al., 2023) | 2018 | observational | SEAR | night | 115 | 32.2(8.8) | 54.8 | SR | 5.46(1.11) | PSQI | 7.68(2.73) |
| 260 | (Kang et al., 2020) | 2018 | observational | SEAR | night | 120 | 26.23(3.39) | 98.3 | SR | 6.88(1.72) | PSQI - Korean | 8.31(3.04) |
| 261 | (Magnavita et al., 2022) | 2021 | observational | EUR | NR | 302 | 49.5(8.6) | 80.8 |  |  | PSQI - Italian | 6.8(3.7) |
| 262 | (Hwang & Lee, 2023) | 2022 | observational | SEAR | night | 131 | 28.71(5.49) | 90.1 |  |  | PSQI - Korean | 5.29(2.56) |
| 263 | (Malak et al., 2023) | 2020 | observational | EMR | night | 450 | 28.97(5.08) | 42.9 |  |  | PSQI | 9.64(2.60) |
| 264 | (Yin et al., 2022) | 2017 | observational | WPR | night | 664 | 29.258(NR) | 79.22 |  |  | PSQI | 6.87(3.59) |
| 265 | (Dai et al., 2023) | 2022 | observational | WPR | NR | 2143 | 30.15(7.7) | 96.8 |  |  | PSQI | 7.59(3.53) |
| 266 | (Hong & Lee, 2020) | 2018 | observational | SEAR | NR | 1874 | 28.8(NR) | 95.3 |  |  | PSQI - Korean | 9.37(3.31) |
| 267 | (Hemmati-Maslakpak et al., 2021) | 2019 | observational | EMR | night | 327 | 33(NR) | 79.2 |  |  | PSQI - Persian | 7.77(3.535) |
| 268 | (Liu et al., 2021) | 2017 | observational | WPR | night | 27575 | 32.01(NR) | 97.5 | SR | 6.67(0.97) |  |  |
| 269 | (Saracoglu et al., 2020) | 2020 | observational | EUR | NR | 208 | 29(7.748) | 27.9 |  |  | PSQI | 6.18(4.356) |
| 270 | (Aldemir & Tasdemir Yigitoglu, 2024) | 2020 | observational | EUR | night | 340 | 37.24(8.18) | 83.5 |  |  | PSQI | 8.26(2.88) |
| 271 | (Jung & Oh, 2020) | 2020 | observational | SEAR | night | 279 | 36.9(9.9) | 100 |  |  | PSQI - Korean | 6.1(3.7) |
| 272 | (Demir & Karadag, 2024) | 2021 | observational | EUR | night | 378 | 29.27(7.79) | 81.7 |  |  | PSQI | 8.25(4.81) |
| 273 | (De Martino et al., 2013) | 2011 | observational | EUR | night | 60 | 31.76(NR) | 88.33 |  |  | PSQI | 7.02(2.87) |
| 274 | (Ebrahimian et al., 2023) | 2020 | observational | EMR | night | 98 | 33.66(5.74) | 100 |  |  | PSQI - Persian | 8.46(4.43) |
| 275 | (Lin et al., 2023) | 2021 | observational | WPR | night | 171 | 22.96(2.61) | 100 |  |  | PSQI | 5.6(2.82) |
| 276 | (Lee et al., 2015) | 2013 | observational | WPR | night | 398 | 30.42(NR) | 97.5 |  |  | PSQI | 7.3(3.1) |
| 277 | (Farag et al., 2021) | 2017 | observational | AMR | night | 1137 | 34.9(11.7) | 93.8 | SR | 6.35(1.1) | PSQI | 8.25(3.55) |
| 278 | (Andreassen et al., 2018) | 2012 | observational | EUR | night | 1781 | 36.1(8.8) | 90 | SR | 6.9(1.6) |  |  |
| 279 | (Berkman et al., 2015) | 2013 | observational | AMR | night | 1524 | 37.55(11.1) | 92.2 | ACT | 7.3(1) |  |  |
| 280 | (Guardiano et al., 2022) | 2020 | observational | AMR | non night | 109 | 42.79(11.11) | 89.73 | SR | 6.455(1.26) |  |  |

*Note.* NR = not reported; *SD* = standard deviation; Geographical Region: AFR= African Region; AMR= Region of the Americas; EMR= Eastern Mediterranean Region; EUR= European Region; SEAR= South-East Asia Region; WPR= Western Pacific Region; Shift Type: night = participants who worked night-time hours, whether as part of a fixed, rotating, or shift work schedule; non night = participants who did not work night-time hours, which included daytime and evening shifts; Sleep Duration Method: SR= Self-report; ACT= Actigraphy

* Please note that the sample size reported for each of the studies did not always correspond to the sample used for analysis.
