# Supplementary material for: Trends in Nurses’ Sleep: A Meta‐Regression Involving 219,245 Nurses From 45 Countries
Source: J Nurs Manag. 2026 Aug 27;2026:9051680. doi: 10.1155/jonm/9051680 (PMC13522381; doi:10.1155/jonm/9051680)
Supplement: Supplementary file 5 [file JONM-2026-9051680-s005.docx]

**Supplementary File 1: The search strategies formulated for each database***.*

| Database | Search syntax |
| --- | --- |
| MEDLINE | exp sleep deprivation/ or exp sleep duration/ or exp sleep quality/ or (sleep* quality or sleep duration or total sleep time or sleep loss or lack of sleep or time in bed or time spent sleeping or time asleep or sleep length or quality of sleep or length of sleep or bedtime or wake time or rise time or sleep period or Pittsburgh Sleep Quality Index or PSQI or sleep disturbances or sleep deprivation or insufficient sleep or sleep insufficiency).ti,ab,kf)  AND  exp Nurses/ or exp Nursing/ or exp Nursing staff/ or exp Student, nursing/ or ((nurse or nurses or nurse staffing or nursing staff or nurse-staffing or nursing-staff or nursing student* or student nurs* or nurs* intern or nurs* interns).ti,ab,kf.) or ((nurs* adj2 (new* employ* or new* recruit* or new* train* or train* or recruit* or registered or enrolled or licensed or consultant or senior or junior)).ti,ab,kf.) or ((nurs* adj3 (shift* or shift work or shift-work or day shift* or night shift* or night-shift* or rotat* shift* or shift schedul* or work schedul* or hospital or tertiary or primary care).ti,ab,kf.) or ((nurs* adj3 (surg* or emergency or triage or community or hospital or palliative care or geriatrics or pediatric or paediatric or ICU or intensive care or psych* or critical care or public health or oncolog* or correctional or clinical)).ti,ab,kf.) or ((nurs* adj3 (undergrad* or student* or register*or prelicensure or graduat* or transition* or personnel or practitioner*)).ti,ab,kf.)  AND  Limit to English language |
| Embase | exp sleep deprivation/ or exp night sleep/ or exp sleep debt/ or exp sleep quality/ or exp sleep time/ or ((sleep* quality or sleep duration or total sleep time or sleep loss or lack of sleep or time in bed or time spent sleeping or time asleep or sleep length or quality of sleep or length of sleep or bedtime or wake time or rise time or sleep period or Pittsburgh Sleep Quality Index or PSQI or sleep disturbances or sleep deprivation or insufficient sleep or sleep insufficiency).ti,ab,kf.)  AND  exp nurse/ or exp nursing staff/ or exp nursing student/ or exp nursing/ or (nurse or nurses or nurse staffing or nursing staff or nurse-staffing or nursing-staff or nursing student* or student nurs* or nurs* intern or nurs* interns).ti,ab,kf.) or ((nurs* adj2 (new* employ* or new* recruit* or new* train* or train* or recruit* or registered or enrolled or licensed or consultant or senior or junior)).ti,ab,kf.) or ((nurs* adj3 (shift* or shift work or shift-work or day shift* or night shift* or night-shift* or rotat* shift* or shift schedul* or work schedul* or hospital or tertiary or primary care)).ti,ab,kf.) or ((nurs* adj3 (surg* or emergency or triage or community or hospital or palliative care or geriatrics or pediatric or paediatric or ICU or intensive care or psych* or critical care or public health or oncolog* or correctional or clinical)).ti,ab,kf.) or ((nurs* adj3 (undergrad* or student* or register*or prelicensure or graduat* or transition* or personnel or practitioner*)).ti,ab,kf.)  AND    Limit to English language |
| Emcare | exp night sleep/ or exp sleep debt/ or exp sleep deprivation/ or exp sleep quality/ or exp sleep time/ or ((sleep* quality or sleep duration or total sleep time or sleep loss or lack of sleep or time in bed or time spent sleeping or time asleep or sleep length or quality of sleep or length of sleep or bedtime or wake time or rise time or sleep period or Pittsburgh Sleep Quality Index or PSQI or sleep disturbances or sleep deprivation or insufficient sleep or sleep insufficiency).ti,ab,kf.)  AND  exp nurse/ or exp nursing staff/ or exp nursing student/ or exp nursing/ or ((nurse or nurses or nurse staffing or nursing staff or nurse-staffing or nursing-staff or nursing student* or student nurs* or nurs* intern or nurs* interns).ti,ab,kf.) or ((nurs* adj2 (new* employ* or new* recruit* or new* train* or train* or recruit* or registered or enrolled or licensed or consultant or senior or junior)).ti,ab,kf.) or ((nurs* adj3 (shift* or shift work or shift-work or day shift* or night shift* or night-shift* or rotat* shift* or shift schedul* or work schedul* or hospital or tertiary or primary care)).ti,ab,kf.) or ((nurs* adj3 (surg* or emergency or triage or community or hospital or palliative care or geriatrics or pediatric or paediatric or ICU or intensive care or psych* or critical care or public health or oncolog* or correctional or clinical)).ti,ab,kf.) or ((nurs* adj3 (undergrad* or student* or register*or prelicensure or graduat* or transition* or personnel or practitioner*)).ti,ab,kf.)    Limit to English language |
| PsychInfo | exp sleep deprivation/ or exp sleep quality/ or exp sleepiness/ or exp sleep onset/ or ((sleep* quality or sleep duration or total sleep time or sleep loss or lack of sleep or time in bed or time spent sleeping or time asleep or sleep length or quality of sleep or length of sleep or bedtime or wake time or rise time or sleep period or Pittsburgh Sleep Quality Index or PSQI or sleep disturbances or sleep deprivation or insufficient sleep or sleep insufficiency).tw.)  AND  exp nurses/ or exp nursing/ or exp nursing students/ or ((nurse or nurses or nurse staffing or nursing staff or nurse-staffing or nursing-staff or nursing student* or student nurs* or nurs* intern or nurs* interns).tw.) or ((nurs* adj2 (new* employ* or new* recruit* or new* train* or train* or recruit* or registered or enrolled or licensed or consultant or senior or junior)).tw.) or ((nurs* adj3 (surg* or emergency or triage or community or hospital or palliative care or geriatrics or pediatric or paediatric or ICU or intensive care or psych* or critical care or public health or oncolog* or correctional or clinical)).tw.) or ((nurs* adj3 (undergrad* or student* or register*or prelicensure or graduat* or transition* or personnel or practitioner*)).tw.)  AND    Limit to English language |
| Scopus | (TITLE-ABS-KEY ("sleep quality" or "sleep duration" or "total sleep time" or "time spent sleeping" or "time asleep" or "sleep length" or bedtime or "wake time" or "sleep length" or "rise time" or "sleep period" or "Pittsburgh Sleep Quality Index" or psqi or "sleep disturbances"))  AND  ((TITLE-ABS-KEY (nurs* W/2 ("new* employ*" or "new* recruit*" or "new* train*" or train* or recruit*))) or (TITLE-ABS-KEY (nurs* W/3 (shift or "shift work" or shift-work or "day shift" or "night shift" or night-shift or "rotat* shift" or full-time or "full time" or "shift system" or "shift schedul*" or "work schedul*"))) or (TITLE-ABS-KEY (nurs* W/3 (surg* or emergency or triage or community or hospital or "palliative care" or geriatrics or pediatric or icu or "intensive care" or psych* or "critical care" or "public health" or "oncolog*" or correctional or clinical))) or (TITLE-ABS-KEY (nurs* W/4 (hospital or tertiary or "primary care"))) or (TITLE-ABS-KEY ( nurs* W/3 (undergrad* or student* or register* or prelicensure or graduat* or transition* or personnel or practitioner*)))) AND Limit to English language  AND    Limit to English language |
